# Identification of Symmetrical RNA Editing Events in the Mitochondria of *Salvia miltiorrhiza* by Strand-specific RNA Sequencing

**DOI:** 10.1038/srep42250

**Published:** 2017-02-10

**Authors:** Bin Wu, Haimei Chen, Junjie Shao, Hui Zhang, Kai Wu, Chang Liu

**Affiliations:** 1 Key Laboratory of Bioactive Substances and Resources Utilization of Chinese Herbal Medicine from Ministry of Education, Institute of Medicinal Plant Development, Chinese Academy of Medical Sciences and Peking Union Medical College, Beijing, 100193, China

## Abstract

*Salvia miltiorrhiza* is one of the most widely-used medicinal plants. Here, we systematically analyzed the RNA editing events in its mitochondria. We developed a pipeline using REDItools to predict RNA editing events from stand-specific RNA-Seq data. The predictions were validated using reverse transcription, RT-PCR amplification and Sanger sequencing experiments. Putative sequences motifs were characterized. Comparative analyses were carried out between *S. miltiorrhiza, Arabidopsis thaliana* and *Oryza sativa*. We discovered 1123 editing sites, including 225 “C to U” sites in the protein-coding regions. Fourteen of sixteen (87.5%) sites were validated. Three putative DNA motifs were identified around the predicted sites. The nucleotides on both strands at 115 of the 225 sites had undergone RNA editing, which we called symmetrical RNA editing (SRE). Four of six these SRE sites (66.7%) were experimentally confirmed. Re-examination of strand-specific RNA-Seq data from *A. thaliana* and *O. sativa* identified 327 and 369 SRE sites respectively. 78, 20 and 13 SRE sites were found to be conserved among *A. thaliana, O. sativa* and *S. miltiorrhiza* respectively. This study provides a comprehensive picture of RNA editing events in the mitochondrial genome of *S. miltiorrhiza*. We identified SREs for the first time, which may represent a universal phenomenon.

RNA editing is an important mechanism to increase the diversities of transcriptomes and proteomes in eukaryotic organisms through post-transcriptionally modifications of mRNA sequences^1^,^2^. The phenomenon has been observed in the nuclei of higher eukaryotes^1^,^3^, plant mitochondria^4^,^5^,^6^,^7^,^8^ and plastids^9^. The modifications of mRNA sequences include the insertion, deletion, and substitution of nucleotides; among them, base substitution is the most frequently observed^3^. RNA editing can occur in the coding and non-coding regions. Most RNA editing were observed at the first or second position of a codon, thereby directly altering the coded amino acid^10^. Particularly, RNA editing can create a start or stop codon^11^,^12^ or remove a stop codon, thereby producing proteins having different sizes^11^,^13^. In contrast, RNA editing in the introns and untranslated regions have been found to regulate the stability of particular mRNA molecules^14^.

Mitochondria are membrane-bound organelles found in most eukaryotic cells and play important roles in energy conversion, the storage of calcium ions, and other metabolic tasks, such as regulation of the membrane potential, steroid synthesis and etc^15^. The mitochondria of flowering plants usually have 300–500 sites that are subjected to RNA editing^16^. Although all kinds of nucleotide substitutions have been reported, the “C to U” substitution is the most frequently observed type^10^ and has been identified in the mitochondria of various plants, such as thale cress (*Arabidopsis thaliana*)^4^, rice (*Oryza sativa*)^7^, rapeseed (*Brassica napus*)^5^, red beetroot (*Beta vulgaris*)^6^, grape (*Vitis vinifera*)^8^, and tobacco (*Nicotiana tabacum*)^17^. Disruption of the normal RNA editing process could lead to severe consequences. For example, the loss of mitochondrial *atp6* RNA editing in sorghum causes male sterility, which is also known as the CMS phenotype^18^. The loss of function of a mitochondrial pentatricopeptide repeat (PPR) protein abolished the “C to U” RNA editing of *rpl16* and affected seed development in maize^16^. Rice *OGR1* also encodes a PPR protein that is essential for RNA editing in the mitochondria and required for normal growth and development^19^.

*Salvia miltiorrhiza* (Danshen) is one of the most economically important medicinal plants, and its products have been widely used for centuries to treat various human diseases, such as cardiovascular disease, dysmenorrhea, and amenorrhea^20^. Understanding the biogenesis of its secondary metabolites, as well as their regulation, has been a subject of extensive research. To date, two draft genome sequences of *S. miltiorrhiza* have been reported^21^,^22^. A dozen of analyses on the *S. miltiorrhiza* transcriptome have been carried out^23^,^24^,^25^,^26^,^27^,^28^. Furthermore, microRNAs^29^ and long non-coding RNAs^30^ have been systematically examined. Many genes or gene families from *S. miltiorrhiza* have also been studied in details. These include genes involved in the biosynthesis of terpenoid and phenolic acid^31^,^32^, genes implicated in the RNA silencing pathway, such as Argonaute gene^33^ and RNA-dependent RNA polymerase gene^34^, and gene families for phenylalanine ammonia-lyase^35^, transcription factors such as SPL^36^, the R2R3-MYB^37^ and WRKY^38^. Together, these information has painted a comprehensive picture of *S. miltiorrhiza*’s biology. Nevertheless, as an important functional diversification mechanism, RNA editing has not been investigated in *S. miltiorrhiza* so far.

With the rapid development of DNA sequencing technologies, RNA sequencing (RNA-Seq) has become an effective method for the discovery of RNA editing sites because of its massive throughput, low cost, and superior sensitivity^39^. In the present study, we systematically identified the RNA editing sites in the mitochondria of *S. miltiorrhiza* based on strand-specific RNA-Seq data previously generated by our group^23^. We have identified a total of 1123 editing sites, among them, 225 were “C to U” editing sites in the protein-coding sequences (CDS). This information has laid the foundation to illustrate how RNA editing functions in *S. mitiorrhiza*’s biology. Most interestingly, we identified 115 sites, at which the nucleotides on both strands were edited. This so-called symmetrical RNA editing (SRE) phenomenon was further identified in the *A. thaliana* and *O. sativa* mitochondria, suggesting the universal presence of SRE in plant mitochondria.

## Results

### Validation of the assembly of the mitochondrial genome sequence of *S. miltiorrhiza*

Because the reference mitochondrial genome (mitogenome) sequence of *S. miltiorrhiza* has not been published by the time this study was initiated, we examined the quality of the genome assembly using three methods. Firstly, we mapped short reads generated from the same species and sequenced using the illumina platform to the reference genome. The entire assembly was covered with short reads at an average coverage of 1390× (Fig. S1). Secondly, we mapped long reads generated from the same species and sequenced with the PacBio RS II platform to the reference genome^40^. The average coverage was 30× and only 0.72% of the reference genome had zero coverage (Supplementary Method 1 and Fig. S2). Last, we extracted the predicted CDS sequences from the reference genome and translated them to the protein sequences. The protein sequences were then used to search the public databases to identify their homologous sequences, which was then subjected to multiple sequence alignment (Supplementary Method 2). The alignments of the mitochondrial proteins of *S. mitiorrhiza* and their homologs were shown in Fig. S3.1–S3.41. No significant discrepancy was observed. Results from these three lines of analyses suggest that the reference genome is of high quality, particular for the regions encoding proteins.

### Discovery of RNA editing sites

We developed a computational pipeline (Fig. S4) to identify RNA editing sites in the mitogenome of *S. miltiorrhiza*. Briefly, clean reads were obtained from the leaf (53,849,024), flower (25,345,740), and root (26,731,733) samples, these reads were mapped to the mitogenome with mismatch = 7. The results showed that 569,489 reads from the leaf, 221,727 reads from the flower, and 52,485 reads from the root were successfully mapped. These mapped reads were grouped based on the strands to which they were mapped by samtools. Consequently, 285,146 reads from the leaf, 111,155 reads from the flower, and 26,387 reads from the root were mapped to the forward strand. By contrast, 284,343 leaf, 110,572 flower, and 26,098 root reads were mapped to the reverse strand.

We first carried out the analysis using data combined from the three tissue types. RNA editing sites from each strand were identified by REDItools. A total of 1123 sites were found to have undergone RNA editing, these sites will be called RNA editing site for simplicity. Among them, 575 sites were found on the forward strand and 548 were found on the reverse strand (Table S1).

As some of the observed nucleotide changes in mRNAs might result from the polymorphism at these sites in the genome DNA; thus, we need to identify possible SNP sites in the genome and exclude them from the steps for the prediction of RNA editing sites. We conducted a low-coverage sequencing (~40×) with whole genomic DNA and identified putative SNP sites in the mitogenome. A total of 63 SNPs and 32 indels were identified (Table S2); 24 of these sites overlapped with the predicted RNA editing sites (Table S1) and 7 sites overlapped with SRE sites. These sites were excluded from the following analyses. Among the 1123 putative editing sites, 24 sites were excluded because they overlapped with the predicted high-quality SNP sites.

**Figure 1:**
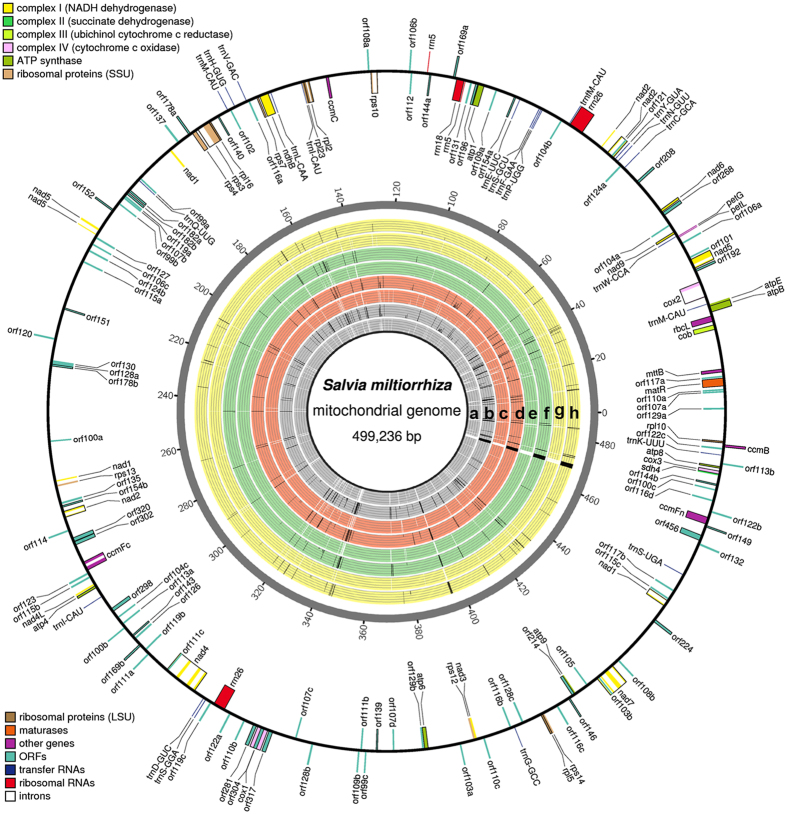
An Ideogram showing the location, types and frequencies of RNA editing on the mitogenome of *S. miltiorrhiza*. The predicted genes are shown on the outermost circle. Genes outside the circle are on the positive strand and oriented clockwise. Genes inside the circle are on the negative strand and oriented anticlockwise. The eight rings correspond to: all RNA editing events found on the positive (**a**) and negative strands (**b**), events found in the flower sample on the positive (**c**) and negative strands (**d**), events found in the leaf sample on the positive (**e**) and negative strands (**f**), and events found in the root sample on the positive (**g**) and negative strands (**h**). The height of the bar represents the relative frequency.

The schematic representation of all predicted RNA editing sites is shown in Fig. 1. As shown, the outermost circle showed the location of the annotated genes on the *S. miltiorrhiza* mitogenome as constructed with OrganellarGenomeDRAW^41^. The inner circles were drawn by custom Perl scripts. The following information are shown in Fig. 1: the predicted RNA editing sites on the positive and negative strands; the types of RNA editing sites; the editing frequency of each site; and the 225 “C to U” predicted editing events in the CDS regions.

We then predicted RNA editing sites using data of each tissue. All RNA editing sites found in each tissue were shown in Table S3. The tissue distribution of these RNA editing sites across the three tissue types were shown in the Venn diagram (Fig. S5). The RNA editing sites found on the positive and negative strands were summarized in Fig. S5A and S5B respectively. The numbers indicate the RNA editing sites found in different categories. 89 and 97 RNA editing sites on the positive and negative strands were found across all three tissue types. And 126 and 124 RNA editing sites on the positive and negative strands were only found in the flower tissue, more than unique RNA editing sites found in the leaf and root tissues, suggesting that RNA editing events might occur more frequently in the flower tissue than in the other two tissues.

A RNA editing site that were found missing in a particular type of tissue might be due to two reasons. Firstly, no reads were found in the region containing the RNA editing sites. Alternatively, there were reads found in the regions containing the RNA editing sites. However, the coverage and/or the editing frequency did not pass our threshold. To show how many RNA editing sites were not predicted due to each of the two reasons, we summarized the tissue distribution patterns for all RNA editing sites in Table S4. In Table S4A, all RNA editing sites were classified into two categories, the ones that passed the threshold and those did not. In Table S4B, all RNA editing sites were classified into three categories, the ones that passed the threshold, the ones that failed to pass the threshold but there were reads covering the regions, and the ones had no read coverage. Comparison of results from Table S4A and S4B showed that many RNA editing sites failed to be predicted could result from either the failure to pass the threshold or the missing of read coverage. These results highlight the impact of read coverage on the prediction of RNA editing sites.

### Characterization of predicted RNA editing events

**Figure 2:**
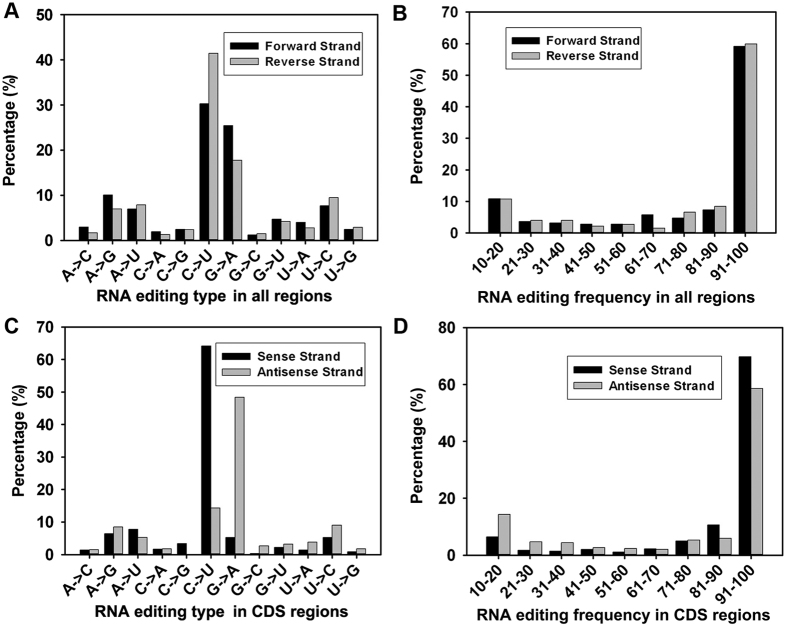
Distribution of RNA editing events on the mitogenome of *S. miltiorrhiza*. The types and frequencies of RNA editing sites across the entire genome are shown in Panels A and B, respectively. The types and frequencies of RNA editing sites in the CDS regions are shown in Panels C and D, respectively.

In our analysis, we filtered out the sites that have undergone more than one type of RNA editing. As a result, the sites reported here are associated with only one type of RNA editing. We found 12 types of RNA editing events (Fig. 2); these events included all possible intra-base transitions: “A to C”, “A to G”, “A to U”, “C to A”, “C to G”, “C to U”, “G to A”, “G to C”, “G to U”, “U to A”, “U to C”, and “U to G”. Overall, the “C to U” transition is the most abundant type, which represents 30.26% and 41.42% of all RNA editing sites on the forward and reverse strands, respectively (Fig. 2a). The complementary “G to A” is the second most abundant type, which represents 25.39% and 17.70% of all RNA editing sites on the forward and reverse strands, respectively. In terms of the editing frequency, the majority of the sites have frequencies between 91%-100%, including 59.13% on the forward strand and 9.85% on the reverse strand (Fig. 2b).

In the CDS regions, the “C to U” transition is also the most abundant type, which represents 64.17% and 14.29% of all events on the forward and reverse strands, respectively (Fig. 2c). Similarly, the complementary “G to A” is the second most abundant type, which represents 5.28% and 48.40% of all RNA editing sites on the forward and reverse strands, respectively. In terms of the editing frequency, the majority of sites have frequencies between 91%-100%, including 69.72% on the forward strand and 58.60% on the reverse strand (Fig. 2d), Interestingly, an obvious bias of “C to U” sites exists on the strands, such that more “C to U” sites are on the forward strand; this trend probably reflects the fact that more genes are coded on the forward strand.

**Figure 3:**
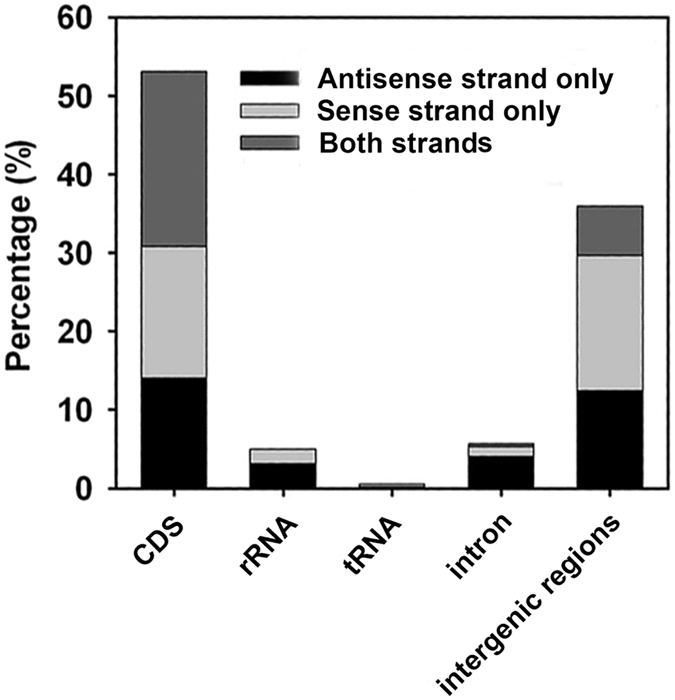
Distribution of all types of RNA editing sites across various regions of the mitogenome. The X axis shows different types of genomic regions. The Y axis shows the frequencies of RNA editing sites on the sense and antisense strands. For the intergenic regions, the sense and antisense strands correspond to the forward and reverse strands respectively.

We also examined the distribution of RNA editing sites across the CDS, intron, rRNA, tRNA, and intergenic regions (Fig. 3). The sites are grouped based on whether the RNA editing events are on the sense strand, antisense strand, or both strands, except for those in the intergenic regions. Sites in the intergenic regions are grouped based on whether they are on the forward strand, reverse strand, or both strands. The RNA editing sites are mostly located in the CDS regions, followed by the intergenic regions. In the CDS regions, 16.76%, 14.01%, and 22.27% of the sites are on the sense, antisense, and both strands. By contrast, 17.22%, 12.40%, and 6.31% of the sites are on the forward, reverse, and both strands in the intergenic regions. More editing sites are on the sense strand than on the antisense strand in the CDS regions. Overall, the RNA editing sites can be located on either strand. Interestingly, the complementary nucleotides at the same genomic location can be edited. These sites are described in the following subsections.

### Characterization of 225 “C to U” RNA editing sites in the CDS regions

The 225 “C to U” RNA editing sites are unevenly distributed among the 26 protein-coding genes (Table S5). Overall, the RNA editing sites were more frequently found in the regions encoding ribosomal proteins or proteins belonging to Complexes I and IV. The highest number of RNA editing sites was found in the regions coding complex I proteins. By contrast, RNA editing sites were rarely found in the regions encoding proteins belonging to Complexes III and V; these trends are consistent with those observed in *A. thaliana*^4^. The percentage of RNA editing sites found at the first and the second positions of codons are 33.3% and 57.8%, respectively (Table S5 and Fig. S6a). A total of 202 out of the 225 RNA editing events lead to the change of amino acid. The RNA editing also increased the overall hydrophobicity of mitochondrial proteins. The percentage of hydrophobic amino acids increased from 41.8% before RNA editing to 73.3% after RNA editing (Fig. S6b). Notably, rps10–2 created a translation start codon (Table S5).

### Validation of predicted RNA editing events

**Figure 4:**
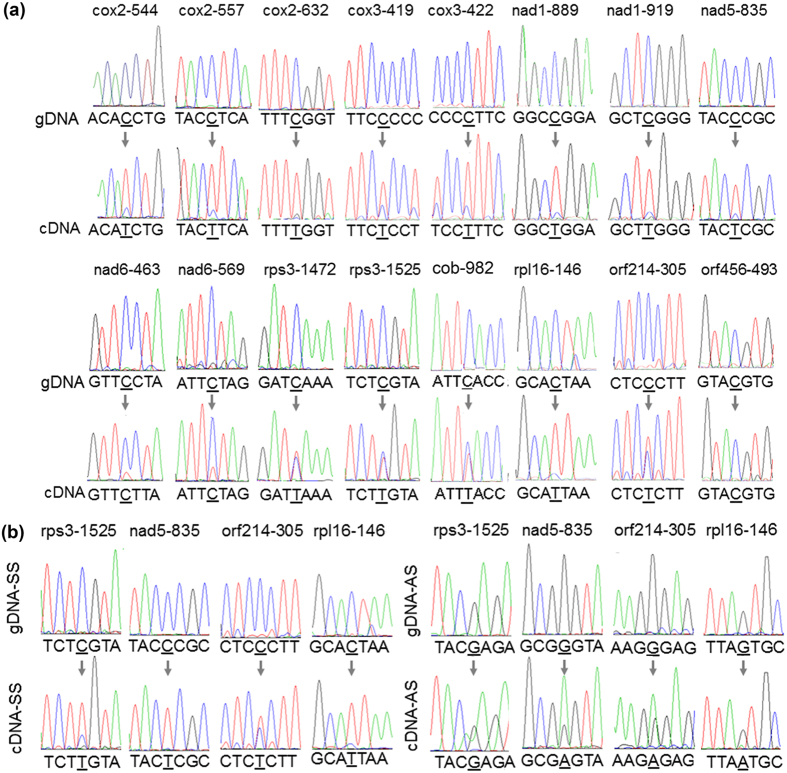
Validation of selected RNA editing sites. Panel A shows sixteen RNA editing sites that were validated with non-strand-specific RT-PCR and DNA sequencing experiments. The names of the RNA editing sites are shown on top of the chromatograms. The edited bases are indicated by arrows. Panel B shows four SRE sites that were validated with strand-specific RT-PCR and DNA sequencing experiments. The names of the sites are shown on top of the chromatograms. The edited bases are indicated by arrows. “gDNA” indicates that the template was genomic DNA; “cDNA” indicates that the template was cDNA. “SS”: sense strand; “AS”: antisense strand.

We randomly selected sixteen predicted RNA editing sites for experimental validation. The results for the sixteen RNA editing sites: cox2-544, cox2-557, cox2-632, cox3-419, cox3-422, nad1-889, nad1-919, nad5-835, nad6-463, nad6-569, rps3-1471, rps-1525, rpl12-196, rpl16-146, orf214-305 and orf456-493 are shown in Fig. 4a. Compared with those observed for the products amplified from the control (gDNA), the predicted “C to U” conversions are visible in the chromatograms at the predicted RNA editing sites for the products amplified from the corresponding cDNA samples, confirming the RNA editing of these sites. For sites such as rps3-1471 and rps3-1525, two overlapping peaks were seen at the predicted RNA editing sites; these peaks might result from the incomplete RNA editing at these sites for all mRNA molecules.

### Possible effects of mitochondrial DNA copies (NUMTs) in the nuclear genome on the predicted mitochondrial RNA editing sites

Due to the technical difficulty in isolating mitochondria specific RNAs, we used total RNAs for the RNA-Seq experiments. It is well known that mitochondrial DNA sequences might be present in the nuclear genome and some RNA editing sites predicted above might actually derived from these NUMTs. To determine the possible effects of NUMTs on the prediction of mitochondrial RNA editing sites, we first identified NUMTs (Supplementary Method 4). A total of 468 NUMTs were identified (Table S6). These NUMTs were used as reference sequences and subjected to RNA editing site prediction with the exact same procedure as that for *S. miltiorrhiza* mitogenome. A total of 2899 and 2784 putative RNA editing sites were predicted on the positive and negative strands of NUMT sequences (data not shown). Comparing the set of RNA editing sites identified on NUMTs with those on the mitogenome revealed eleven possible identical RNA editing sites (Table S7).

### Identification of SREs in the mitochondria of *S. miltiorrhiza*

The use of strand-specific RNA-Seq data allowed us to distinguish RNA editing events observed on particular strands. Carefully examining the identified RNA editing sites in the protein-coding regions revealed that the nucleotides on both strands at 115 sites were edited (Table S8). We designated these RNA editing events as symmetrical RNA editing (SRE). To validate these predicted strand-specific editing events, the cDNA was reverse transcribed with forward and reverse primers specific for four predicted SRE sites: rps3-1525, nad5-835, orf214-305, and rpl16-146. The transcribed products were then subjected to PCR amplification and Sanger DNA sequencing as described. The results are shown in Fig. 4b. Compared with those amplified from gDNA, the “C to U” editing events on the sense strand (the strand coding for proteins) and “G to A” editing events on the antisense strand were simultaneously observed, agreeing with our predictions.

### Presence of conserved sequence motifs around the RNA editing sites

**Figure 5:**
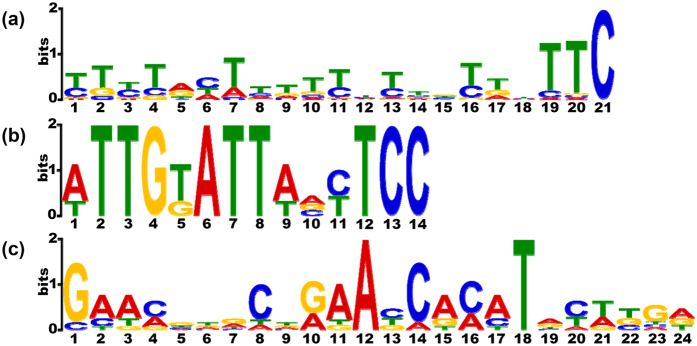
Putative RNA editing related sequence motif identified by MEME. The horizontal axis represents the base position in the corresponding motif. The vertical axis shows the bit score for each base. Panels A, B and C represent the three different motifs, details are described in the text.

Previous studies suggest that RNA editing events are mediated by RNA-binding proteins that recognize specific sequence motifs around the RNA editing sites. Generally, the nucleotides 20–25 bp upstream of the RNA editing site are sufficient for recognition by RNA machinery^42^. To determine if there are any conserved sequence motifs around these predicted RNA editing sites, the neighboring sequences (−25 to 0) of the 225 “C to U” RNA editing sites in the CDS regions were extracted and analyzed with the MEME software. Three motifs were identified. Motif 1 was 21 bp-long and found in the upstream regions of 52 RNA editing sites; the overall E-value cutoff was 9.1e-034 (Fig. 5a). Motif 2 was 14 bp-long and found in the upstream regions of 7 RNA editing sites; the overall E-value cutoff was 8.1e-003 (Fig. 5b). Motif 3 was 24 bp-long and found in the upstream regions of 9 RNA editing sites; the overall E-value cutoff was 5.2e-002 (Fig. 5c). The functional validation of these conserved motifs shall be the subject of future study.

### Potential effects of RNA editing

To determine the potential functional effects of these RNA editing events, the RNA and protein sequences resulting from the 225 “C to U” type of RNA editing were subjected to structure prediction. Some significant changes in the RNA and protein tertiary structures were observed. For example, the RNA secondary structure of the *Rps3* gene was changed after editing; the large loop (highlighted with the arrowhead) was divided into two smaller loops (Fig. S7a). The tertiary structure of the Rps3 protein was also altered (Fig. S7b); part of the domain was separated from the others, thereby suggesting the creation of a protein product with novel functions.

### Identification of SREs in *A. thaliana* and *O. sativa* cv. *japonica*

To eliminate the possibility that the observed SRE events resulted from our particular experimental procedure, we set out to determine whether SREs were also present in other plants. We mapped a set of strand-specific RNA-Seq data from *A. thaliana* and *O. sativa* cv. *japonica* to their mitogenome sequences respectively, and predicted the RNA editing events using the same procedure as that for *S. miltiorrhiza*. Considering the high false discovery rate usually associated with RNA editing site prediction, we only focus on those found in the CDS regions. Particularly, when comparing RNA editing sites identified from the three species, we only compared those sites found in CDS having homologs in all three species. The comparison results of conserved RNA editing sites found in the homologous CDS among the three species were shown in Table S9, in which the genomic position, RNA editing site name, coverage, substitution type, editing frequency, P value, strand for the CDS and strand for the sites were shown. The distribution of RNA editing sites among the three species were shown in the Venn diagram (Fig. S8). Fifty-five RNA editing sites on the sense strand were found to be conserved across all three species (Fig. S8a). In contrast, 33 RNA editing site on the negative strand were found to be conserved across the three species (Fig. S8b). As a whole, there were more conserved RNA editing sites found between *A. thaliana* and *O. sativa* than those found between *S. miltiorrhiza* and either of them.

We then identified SRE from *A. thaliana* and *O. sativa*. For *A. thaliana*, a total of 327 SRE sites were identified (Table S10). In contrast, for *O. sativa*, a total of SRE 369 sites were identified (Table S11). Conserved SRE sites were identified and are listed in Table S12. The distributions of conserved SRE sites among 26 clusters of homologs from the three species were shown in the Venn diagram (Fig. S9). As shown, 19 SRE sites were found to be conserved among all three species, These 19 SRE sites include 4 in cox3, 4 in nad1, 1 in nad4, 3 in nad5, 1 in nad6, 2 in nad7, 1 in rpl16 and 3 in rps12 genes (Table S12). In addition, 78, 20 and 13 SRE sites were found to be conserved between *A. thaliana* and *O. sativa, A. thaliana* and *S. miltiorrhiza, O. sativa* and *S. miltiorrhiza* respectively (Fig. S9). The identification of these SRE sites suggests that SRE be a universal phenomenon. To our knowledge, these SREs have not been reported before and their underlying functional roles have not been elucidated.

## Discussion

### RNA editing events in the mitochondria of *S. miltiorrhiza*

In this study, we first developed a computational pipeline for the identification of RNA editing sites based on strand-specific RNA-Seq data. Particularly, we established a method to optimize the parameters critical for the identification of RNA editing sites. Using this pipeline, we found 1123 putative editing sites in the mitochondria of *S. miltiorrhiza*. The number of “C to U” editing sites in the *S. miltiorrhiza* coding regions is 225, which is less than those reported for *A. thaliana* [441]^4^, *O. sativa* [427]^7^, *B. napus* [491]^5^, *B. vulgaris* [357]^6^, and grape [401]^8^. Several possible reasons may account for these discrepancies.

Firstly, RNA editing could be tissue-specific or development-related^43^, as well as stress- and hormone- induced^44^. It is possible that not all RNA editing events are represented in our data set. Secondly, most of the previous studies did not use strand-specific RNA-Seq data. Some of the “G to A” editing events in the antisense transcripts might be mistreated as the “C to U” editing events, thereby causing the potential overestimation of the number of the “C to U” RNA editing events in the previous studies. Certainly this possibility needs to be examined carefully. Thirdly, multiple type of RNA editing events can happen at the same sites. In our current analysis pipeline, only sites undergone one type of RNA editing were selected for detailed characterization. Sites undergone multiple RNA editing were left out due to concerns that these sites might contain more errors. As a result, some of the true RNA editing events might have been left out. Lastly, some RNA editing sites are very close to each other and the corresponding reads were failed to be mapped to the reference genome due to large number of mismatches. Although we have carried out a systematic optimization of the mismatch numbers, it is still possible that certain numbers of RNA editing events were missed.

### Technical limitations

There are several technical issues might influence the results. Firstly, mitochondrial DNA fragments can be transferred to the nuclear genomes^45^ and the nuclear genome might harbor sequences similar to those found in the mitogenome. Our RNA-Seq data were obtained from total cellular RNAs; thus, it could not be eliminated the possibility that some of the predicted RNA editing events may have actually occurred in the nucleus. As the majority of the “C to U” alterations occur in the mitochondria of flowering plants^46^, our study focused more on this type of RNA editing event to minimize the possible mis-assignment of nuclear RNA editing events to the mitochondria. Secondly, in our analyses, we focus on the sites associated with only one type of RNA editing event. As a result, we might have missed some of the functionally important RNA editing types at particular sites as these sites are associated with multiple types of RNA editing. Thirdly, our study focused mostly on the RNA editing sites in the protein-coding regions. Those locate in the non-protein coding regions might still have significant biological functions. Lastly, we have optimized the mismatch numbers in our computational pipeline. However, because the density of RNA editing sites is likely to vary across different regions, it is conceivable that our pipeline might still over- or under-estimate the numbers of RNA editing events. Additional experiments are needed to identify additional RNA editing events.

### Discovery of the SRE events

A unique finding of this study is the 115 SRE sites (Table S4). When these SRE events were first observed, we suspected that they resulted from errors in the strand-specific RNA sequencing experiments. However, the strand-specific RT-PCR experiments confirmed the presence of these SRE events. The identification of SRE sites in *A. thaliana* (Table S10) and *O. sativa* (Table S11), and particularly, the identification of conserved sites among the species further support their actual existence. With the availability of strand-specific RNA-Seq dataset for many other organisms, it would be straightforward to examine these additional organisms for the present of SRE. SRE might represent a novel mechanism by which the cells increase their mRNA and protein diversities.

### Possible molecular mechanisms for the genesis of the SRE events

The next question is what functions SRE might have. On one hand, the simultaneous editing on both strands may have important functional consequences. On the other hand, the editing of the antisense strand could be a by-product of the editing of the sense strand. Another interesting question is how SRE is implemented. To answer this question, factors involved in RNA editing have to be considered.

Previous research showed that several RNA modification reactions required a *cis*-acting or a *trans*-acting antisense RNA, which participate in RNA duplex formation^47^. The *cis*-element was recognized by a *trans*-factor that recruited the RNA editosome to the site. The most well-studied *trans*-factor is the pentatricopeptide repeat protein (PPR), which contain repeats with a ~35-amino acid sequence motif^42^. The PPR proteins belonging to the E and DYW classes were found to be the main *trans*-factors involved in RNA editing^48^,^49^. By contrast, members of the P family were found to participate in splicing^42^. The structure and function relationship of the PPR proteins have also been studied. For example, PPR10 forms an anti-parallel homodimer in its crystal form, which recognizes the single-stranded RNA^50^. By contrast, THA8 forms an asymmetric dimmer with the target at the dimeric surface^51^. In addition to these PPR proteins, multiple-site organellar RNA editing factors can interact with the PPR proteins and may be directly or indirectly involved in the RNA editing process. Other than the PPR proteins, the edited antisense transcripts (*cis-*NATs) might also be involved in the process to generate SRE. They can perfectly pair with the edited sense coding transcripts and may form double-stranded RNA *in vivo* in some cases^52^.

Taken together, we propose two possible mechanisms for the generation of SRE. First, the sequence around the RNA editing sites on the sense and antisense strands contain motifs that are recognized by the proteins involved in RNA editing. Consequently, the complementing sites on both strands were edited. Second, the double-strand RNA structure might be involved in the RNA editing process. The two sites on both strands were not distinguishable in certain contexts, thereby leading to the editing of the sites on both strands. In the future, data from other organisms need to be examined to further examine the universality and prevalence of the SRE events. Additional functional studies are needed to illustrate the mechanism for the genesis of SRE.

## Materials and Methods

### Plant materials, DNA and RNA extraction

The *S. miltiorrhiza* strain 99-3 was grown in the experimental field of the Institute of Medicinal Plant Development (Beijing, China). The genomic DNA was extracted by the CTAB method^53^. Total RNA was extracted from fresh leaf, flower, and root samples of three *S. miltiorrhiza* plants with the RNAprep Pure Plant Kit (Tiangen, China) according to the manufacturers’ recommendations. The total RNA samples were treated with RNase-free DNase I (Promega) to remove genomic DNA contamination.

### The mapping-based method for the identification of RNA editing sites in *S. miltiorrhiza* mitochondria

The flowchart for the identification of RNA editing sites is shown in Fig. S4. Briefly, the sequence for the mitogenome of *S. miltiorrhiza* was downloaded from REFSeq (NC_023209). The RNA-Seq data of the flower, leaf, and root samples (SRR1043988, SRR1045051, and SRR1020591) were generated in our previous study^23^ and can be downloaded from GenBank SRA database (http://www.be-md.ncbi.nlm.nih.gov/sra). The cleaned reads from the three tissues were mapped to the mitogenome by bowtie2 (version 2.2.1)^54^ with mismatch = 7. The optimization of this parameter was described in Supplementary method 3 and Fig. S10. These mapped reads were extracted by samtools^55^ with the parameter ‘flag = 16’ to separate those mapped to the forward and reverse strands. Finally, RNA editing sites from both strands were separately called by REDItools^56^ with the following cutoffs: coverage ≥ 5, frequency ≥ 0.1, and P ≤ 0.05. We named this approach the “mapping-based method”.

### Parameter optimization for the mapping-based discovery of RNA editing sites

Given this mapping-based method, a critical parameter is the mismatch number used to map the reads to the reference genome. Our preliminary analysis showed that the use of a small mismatch number might lead to the significant underestimation of the RNA editing sites because several genes have RNA editing sites that are very closely located. For example, in the mitogenome of *A. thaliana*, 455 RNA editing sites are distributed in the CDS regions of 34 genes. An average of 3.4 RNA editing sites can be found on every 100 bp of sequence. The *ccb206* gene has 40 RNA editing sites; the average distance between two RNA editing sites is only 14.5 bp. Consequently, the default parameters, such as mismatch number of 2, will exclude the mapping of edited reads to these regions. On the other hand, a large mismatch number for the reads mapping might lead to a high false-positive rate (results not shown).

To determine the “true” number of RNA editing sites, we then developed an “assembly-based” method to predict the RNA editing sites. In this method, the clean reads obtained for the three tissue types were separately assembled by trinity^57^ with the ‘-SS_lib_type FR’ option and other default parameters. No mapping to the reference genome sequences was involved in this process; thus, the effects of mismatch numbers are minimal. These strand-specific transcripts were compared with the mitogenome sequence by BLAT^58^ with the default parameters. The differences between the genome sequences and transcript sequences were considered RNA editing sites. These sites are likely to represent the dominant RNA editing sites at these positions and are considered to be the “true” RNA editing sites. The limitation of this assembly-based method is that it would miss the RNA editing sites that are less frequent.

To find the optimal mismatch numbers, the mapping-based method (method 1) was used to identify RNA editing sites with the mismatch numbers ranging from 2 to 10. The identified RNA editing sites were compared with those identified by the assembly-based method (method 2). The sensitivity and specificity were calculated for the results obtained with different mismatch numbers by the following formulas: Sensitivity = (RNA editing sites discovered by both methods 1 and 2)/(RNA editing sites discovered by method 2) and Specificity = (RNA editing sites discovered by both methods 1 and method 2)/(RNA editing sites discovered by method 1). Overall, the best combination of sensitivity and specificity was observed with mismatch number = 7 (Fig. S10). This number was used in the subsequent analyses.

### Detection of single nucleotide polymorphism (SNP) sites in the mitogenome

The whole genomic DNA samples were subjected to next-generation sequencing (NGS) with Hiseq2000 (Illumina) sequencing platform. A total of 93,695,535 high-quality reads with 100 bp were obtained. These high-quality reads were mapped to the mitogenome by BWA (version 0.7.12)^22^ with default parameters. Then, samtools was used to convert the SAM file into the BAM file, sort the BAM file, remove the duplicates with the rmdup command, and generate the BCF file with the mpileup command and the following parameters: “-g usDf -C 50”. The SNPs were called with bcftools^23^. The SNPs with read coverage ≥ 10 and variant frequency ≥ 0.1 were selected as high-quality SNPs. These sites were excluded in the steps to identify the RNA editing sites.

### Validation of predicted RNA editing sites

Total genomic DNA and RNA were extracted and processed as described above. Two types of primers (Table S13) were used for non-specific and strand-specific reverse transcription. For the non-specific reverse transcription, random primers were used to transcribe 1 μg of total RNA extracted from the leaf sample with 200 U SuperScript III Reverse Transcriptase (Invitrogen) in a 20 μl reaction volume. For strand-specific reverse transcription, the gene-specific forward and reverse primers were used to reverse transcribe the target regions from 200 ng of the respective pooled RNA samples from the leaf, flower, and root tissues. Gene-specific primers were designed around the editing sites by DNAMAN (version 6.0). The reactions were performed with 60 U SuperScript III Reverse Transcriptase (Invitrogen) in a 10 μl reaction volume. The PCR amplifications were performed with 50 ng of cDNA or gDNA as the template under the following conditions: 95 °C for 3 min, followed by 35 cycles of 95 °C for 30 s, 58 °C for 30 s, and 72 °C for 30 s, with a final extension at 72 °C for 10 min. Subsequently, PCR products were detected on a 1.5% agarose gels and purified with the AXYGEN Gel Extraction Kit (AXYGEN, USA) according to the manufacturer’s instructions. The purified PCR products were sequenced by the Sanger method (Beijing Sunbiotech Corp., Beijing). The sequence chromatograms were examined by Lasergen (version 9, DNASTAR, Madison, Wisconsin) to identify RNA editing sites.

### Contextual analysis of RNA editing sites

The sequence context of the RNA editing sites was analyzed to discover potential motifs that may determine its interaction with the RNA editing enzyme complexes. The regions covering the upstream 25 bp around the RNA editing sites^42^ were extracted and subjected to pattern analyses by the MEME software (http://meme-suite.org/tools/meme) 59.

### Prediction of RNA and protein structures after RNA editing

The secondary structures of RNA were predicted by RNAfold (version 2.1.6) with the default parameters^60^. Protein structures were predicted by RaptorX web server (http://raptorx.uchicago.edu/) with the default parameters.

### Discovery of symmetrical RNA editing in *A. thaliana* and *O. sativa* mitochondria

The genome sequences of *A. thaliana* (NC_001284.2) and *O. sativa* (NC_011033) mitochondria were downloaded from GenBank. The strand-specific RNA-Seq data of *A. thaliana* (SRR1004790) and *O. sativa* (SRR1618549) were downloaded from the GenBank SRA database. The SRE sites on the mitochondrial genomes of *A. thaliana* and *O. sativa* were identified by the mapping-based method with the same parameters as those described for *S. miltiorrhiza*.

To identify the conserved SRE sites, we first extracted all CDS sequences from each of the three genomes. Only CDS having homologs across the three species were subjected to the following analysis. Secondly, for each cluster of homologous CDS, multiple sequence alignment was conducted. Thirdly, the consensus sequence for each cluster was obtained. The conserved RNA editing sites were those that were aligned to the same consensus position and had undergone the same type of editing.

## Additional Information

**How to cite this article**: Wu, B. *et al*. Identification of Symmetrical RNA Editing Events in the Mitochondria of *Salvia miltiorrhiza* by Strand-specific RNA Sequencing. *Sci. Rep.* **7**, 42250; doi: 10.1038/srep42250 (2017).

**Publisher's note:** Springer Nature remains neutral with regard to jurisdictional claims in published maps and institutional affiliations.

## Supplementary Material

Supplementary Information

Supplementary Dataset 1

Supplementary Dataset 2

Supplementary Dataset 3

Supplementary Dataset 4
