# Supplementary material for: Identification of Symmetrical RNA Editing Events in the Mitochondria of Salvia miltiorrhiza by Strand-specific RNA Sequencing
Source: Sci Rep. 2017 Feb 10;7:42250. doi: 10.1038/srep42250 (PMC5301482; doi:10.1038/srep42250)
Supplement: Supplementary Information [file srep42250-s1.pdf]

Supplementary information

Identification of Symmetrical RNA Editing Events in the Mitochondria of *Salvia miltiorrhiza* by Strand-specific RNA Sequencing

Bin Wu[☯], Haimei Chen[☯], Junjie Shao, Hui Zhang, Kai Wu, Chang Liu*

Key Laboratory of Bioactive Substances and Resources Utilization of Chinese Herbal Medicine from Ministry of Education, Institute of Medicinal Plant Development, Chinese Academy of Medical Sciences and Peking Union Medical College, Beijing, 100193, China

☯ These authors contributed equally to this work.

* To whom correspondence should be addressed. Tel: +86-10-57833111; Fax: +86-10-62899715; Email: cliu6688@yahoo.com.

Supplement methods

1 Mapping of Illumina short reads and PacBio Long reads to the mitochondrial genome (mitogenome) of *S. miltiorrhiza*

To validate the accuracy of the mitogenome assembly of *S. miltiorrhiza* (NC_023209), the short and long reads derived from the Illumina and Pacbio sequencing platforms, respectively, were mapped to the mitogenome.

To create the short Illumina reads, the genomic DNA of *S. miltiorrhiza* Bunge *cv* 99-3 was sheared to yield approximately 500 bp long fragments for paired-end library construction according to the manufacturer's instructions (Illumina Inc., San Diego, CA). The library was sequenced on Illumina HiSeq 2000 (Illumina Inc.). In total, 187,403,838 paired-end reads (2 × 100 bp) were obtained. The raw sequence reads with high quality were mapped to the mitogenome of *S. miltiorrhiza* (NC_023209) using BWA (v0.7.12) program with default settings. The statistics of the coverage of the entire mitogenome of *S. miltiorrhiza* was performed using Qualimap v.2.2 (Fig. S1)..

The PacBio data had been described in our previous paper (Chen H, Zhang J, Yuan G, Liu C. Complex Interplay among DNA Modification, Noncoding RNA Expression and Protein-Coding RNA Expression in *Salvia miltiorrhiza* Chloroplast Genome. Margis R, ed. PLoS ONE. 2014;9(6):e99314. doi:10.1371/journal.pone.0099314.). Briefly, the gemomic DNA of *S. miltiorrhiza* Bunge *cv* 99-3 was used to construct two libraries with the insert sizes of 1 kb and 10 kb, which were subsequently subjected to DNA sequencing use the PacBio RS platform following the standard protocol provided by the manufacturer. A total of 575,643,513bp long reads derived from the PacBio sequencing platform were mapped to the mitogenome of *S. miltiorrhiza* (NC_023209) using the program RS_BridgeMapper with default parameters. The average length was 3,274bp. Genome coverage of 99.26% was achieved with a mean coverage of 32.07X. The observed coverage across the mitogenome of *S. miltiorrhiza* was showed in the Fig. S2.

2 Alignment of mitochondrial proteins of *S. miltiorrhiza* and their homologous

The protein-coding sequences annotated in the mitogenome of *S. miltiorrhiza* (NC_023209) were searched against the RefSeq database using the BLASTP algorithm, with an e-value cutoff of 1e-5. The ten best-hit homologous sequences found in RefSeq database were selected and subjected to multiple sequence alignment using MegAlign (DNASTAR, WI) (Fig. S3.1-3.41).

3 Identification of Nuclear Mitochondrial DNA (NUMT) in *S. miltiorrhiza.*

The mitogenome sequences were compared with the genomic assembly reported previous (Xu, H. *et al.* Analysis of the genome sequence of the medicinal plant *Salvia miltiorrhiza*. *Mol Plant*, doi: 10.1016/j.molp.2016.1003.1010 (2016).
). Using a threshold of 1e-4 as described previously (Hazkanicovo E, Zeller R M, Martin W. Molecular Poltergeists: Mitochondrial DNA Copies (NUMTs) in Sequenced Nuclear Genomes[J]. Plos Genetics, 2010, 6(2):217-217.), we identified all NUMTs including those possible mitochondrial genome fragments. In the sequenced genome, approximately 2.8 SNP were identified in every 1kb sequences. This gives a degree of genome heterozygosity of 0.28%. As a result, any fragments identified above with a percentage identity greater than 99.72% were likely to represent the fragments of mitogenome. Those fragments with percentage identify less than 99.72% were selected as candidate NUMTs (Table S6).

4 Optimization of the mismatch number used for RNA-editing site identification.

To optimize the mismatch numbers, we selected strand-specific RNA-Seq data set for *A. thaliana* (SRR1004790), *Oryza sativa* (SRR1618549) and *S. miltiorrhiza* and run them through our RNA-editing identification pipeline using various mismatch numbers. The RNA-editing sites for *A. thaliana* and *O. sativa* were well retrieved from the GenBank records. The sensitivities and specificities obtained for various mismatch numbers were plotted in Fig. S10. As shown in Fig. S10a and S10b, the sensitivity and specificity have both reached optimal levels when the mismatch number is >=6. Similar trends were observed for the sensitivities and specifies obtained for *S. miltiorrhiza* (Fig. S10c). And results obtained with mismatch number 7 were used in the following analysis for *S. miltiorrhiza*.

Supplementary Figure

Figure S1 Coverage of Illumina reads across the mitogenome of *S. miltiorrhiza*.

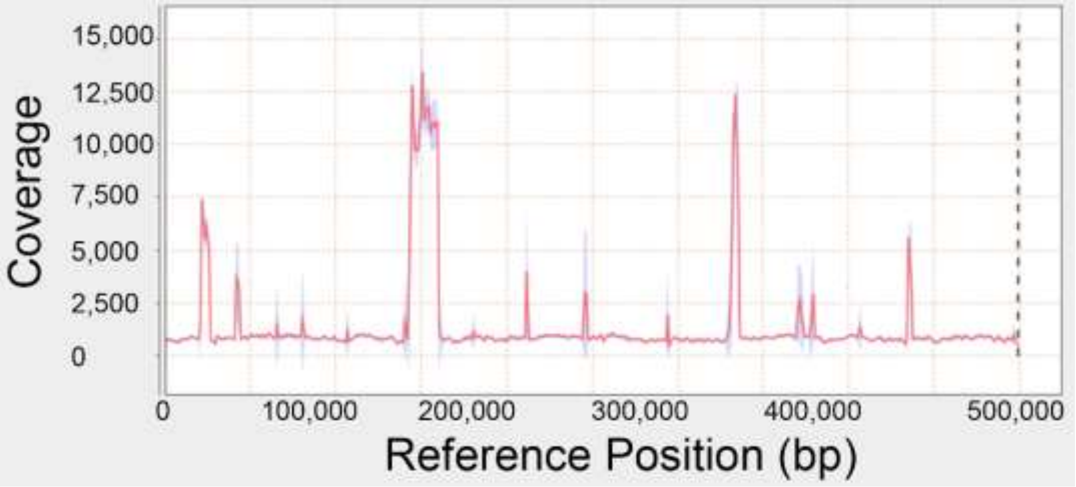

Figure S2 Coverage of PacBio reads across the mitogenome of *S. miltiorrhiza*.

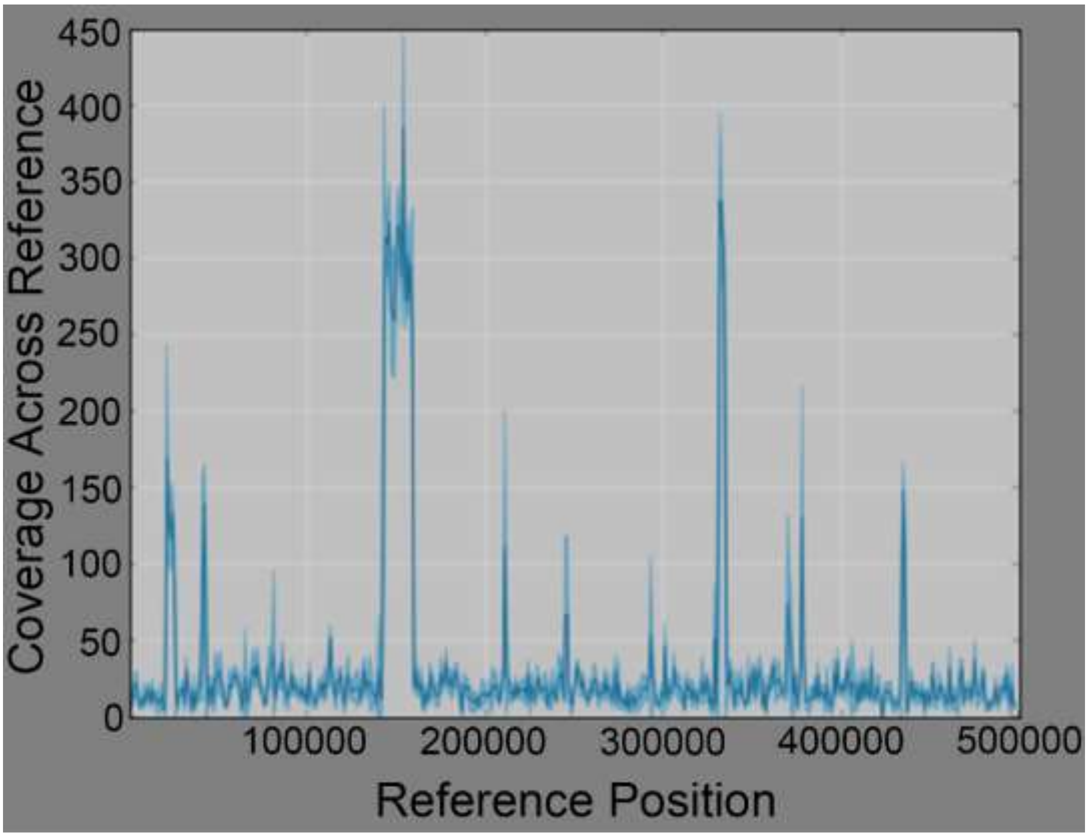

Figure S3.1 The amino acid sequence allignment of ATP1 in mitogenome of *S. miltiorrhiza* and the corresponding homologs in other species

```
                         *        20         *        40         *        60         *        80         *       100
YP 009243651 : MEFSPRAAELTTLLESRISNFYTNFQVDEIGRVVSVGDGIARVYGLNEIQAGEMVEFASGVKGIALNLENENVGIVVFGSDTAIKEGDLVKRTGSIVDVP : 100
YP 009237616 : MEFSVRAAELTTLLESRITNFYTNFQVDEIGRVVSVGDGIARVYGLNEIQAGELVEFASGVKGIALNLENENVGIVVFGSDTSIKEGDLVKRTGSIVDVP : 100
XP 011101782 : MELSPRAAELTTLLESRISNFYTNFQVDEIGRVVSVGDGIARVYGLNEIQAGEMVEFSSGVKGIALNLENENVEIVVFGSDIAIKEGDLVKRTGSIVDVP : 100
ATP1 SAMI    : MELSPRAAELTTLLESRISNFYTNFQVDEIGRVVSVGDGIARVYGLNEIQAGEMVEFSSGVKGIALNLENENVGIVVFGSDTAIKEGDLVKRTGSIVDVP : 100
YP 008964126 : MTLSPKAAELTTLLESRISHFYTNFQVDEIGRVVSVGDGIARVYGLNEIQAGEMVEFSSGVKGIALNLENENVGIVVFGSDTAIKEGDLVKRTGSIVDVP : 100
YP 007516887 : MEFSVRAAELTTLLESRITNFYTNFQVDEIGRVVSVGDGIARVYGLNEIQAGEMVEFASGVKGIALNLENENVGIVVFGSDTAIKEGDLVKRTGSIVDVP : 100
YP 006460147 : MELSPRAAELTTLLESRISNFYTNFQVDEIGRVVSVGDGIARVYGLNEIQAGEMVEFSSGVKGIALNLENENVGIVVFGSDTAIKEGDLVKRTGSIVDVP : 100
YP 005090418 : MELSPRAAELTTLLENRISNFYTNFKVDEIGRVVSVGDGIARVYGLNEIQAGEMVEFASGVKGIALNLENENVGIVVFGSDTAIKEGDLVKRTGSIVDVP : 100
YP 005090487 : MEFSLRAAELTTLLESRITNFYTNFQVDEIGRVVSVGDGIARVYGLNEIQAGEMVEFASGVKGIALNLENENVGIVVFGSDTAIKEGDLVKRTGSIVDVP : 100
YP 003875497 : MEFSPRAAELTTLLESRISNFYTNFQVDEIGRVVSVGDGIARVYGLNEIQAGEMVEFASGVKGIALNLENENVGIVVFGSDTAIKEGDLVKRTGSIVDVP : 100
NP 064105    : MEFSPRAAELTNLLESRITNFYTNFQVDEIGRVVSVGDGIARVYGLNEIQAGEMVEFASGVKGIALNLENENVGIVVFGSDTAIKEGDLVKRTGSIVDVP : 100
               Me S 4AAELTtLLEsRI3nFYTNFqVDEIGRVVSVGDGIARVYGLNEIQAGE6VEF SGVKGIALNLENENVgIVVFGSDtaIKEGDLVKRTGSIVDVP

                         *       120         *       140         *       160         *       180         *       200
YP 009243651 : AGKAMLGRVVDALGVPIDGRGALSDHERRRVEVKAPGIIERKSVHEPMQTGLKAVDSLVPIGRGQRELIIGDRQTGKTAIAIDTILNQKQMNSRATSESE : 200
YP 009237616 : AGKAMLGRVVDALGVPIDGRGALSDHERRRVEVKAPGIIERKSVHEPMQTGLKAVDSLVPIGRGQRELIIGDRQTGKTAIAIDTILNQKQMNSRATSESE : 200
XP 011101782 : AGKAMLGRVVDALGVPIDGRGALSAHERRRVEVKAPGIIKRKSMHEPMQTGLKAVDSLVPIGRGQRELIIGDRQTGKTTIAIDTILNQKQLNSKAKGESE : 200
ATP1 SAMI    : AGKAMLGRVVDALGVPIDGRGALSAHERRRVEVKAPGIIERKSVHEPMQTGLKAVDSLVPIGRGQRELIIGDRQTGKTAIAIDTILNQKQLNSKATSESE : 200
YP 008964126 : AGKAMLGRVVDALGLPIDGRGALSAHERRRVEVKAPGIIERKSVHEPMQTGLKAVDSLVPIGRGQRELIIGDRQTGKTAIAIDTILNQKQLNSKATSESE : 200
YP 007516887 : AGKAMLGRVVDALGVPIDGRGALSDHERRRVEVKAPGIIERKSVHEPMQTGLKAVDSLVPIGRGQRELIIGDRQTGKTAIAIDTILNQKQMNSRATSESE : 200
YP 006460147 : AGKAMLGRVVDALGVPIDGRGALSAHERRRVEVKAPGIIERKSVHEPMQTGLKAVDSLVPIGRGQRELIIGDRQTGKTAIAIDTILNQKQLNSKATSESE : 200
YP 005090418 : AGKAMLGRVVDALGVPIDGRGALSDHERRRVEVKAPGIIERKSVHEPMQTGLKAVDSLVPIGRGQRELIIGDRQTGKTAIAIDTILNQKQLNLKATSESE : 200
YP 005090487 : AGKAMLGRVVDALGVPIDGRGALSDHERRRVEVKAPGIIERKSVHEPMQTGLKAVDSLVPIGRGQRELIIGDRQTGKTAIAIDTILNQKQMNSRATSESE : 200
YP 003875497 : AGKAMLGRVVDALGVPIDGRGALSDHERRRVEVKAPGIIERKSVHEPMQTGLKAVDSLVPIGRGQRELIIGDRQTGKTAIAIDTILNQKQLNSKATSESE : 200
NP 064105    : AGKAMLGRVVDALGVPIDGRGALSDHERRRVEVKAPGIIERKSVHEPMQTGLKAVDSLVPIGRGQRELIIGDRQTGKTAIAIDTILNQKQLNSKATSESE : 200
               AGKAMLGRVVDALG6PIDGRGALS HERRRVEVKAPGIIeRKS6HEPMQTGLKAVDSLVPIGRGQRELIIGDRQTGKTaIAIDTILNQKQ6Ns4AtsESE

                         *       220         *       240         *       260         *       280         *       300
YP 009243651 : TLYCVYVAIGQKRSTVAQLVQILSEANALEYSILVAATASDPAPLQFLAPYSGCAMGEYFRDNGMHALIIYDDLSKQAVAYRQMSLLLRRPPGREAFPGD : 300
YP 009237616 : TLYCVYVAIGQKRSTVAQLVQILSEANALEYSILVAATASDPAPLQFLAPYSGCAMGEYFRDNGMHALIIYDDLSKQAVAYRQMSLLLRRPPGREAFPGD : 300
XP 011101782 : TLYCVYVAIGQKRSTVAQLVQILSEANALEYSILVAATASDPTPLQFLTPYSGCAMGEYFRNNGMHALIIYDDLSKQAVAYQQMSLLLRRPPGREAFPGD : 300
ATP1 SAMI    : TLYCVYVAIGQKRSTVAQLVQILSEANALEYSILVAATASDPAPLQFLAPYSGCAMGEYFRDNGMHALIIYDDLSKQAVAYRQMSLLLRRPPGREAFPGD : 300
YP 008964126 : TLYCVYVAIGQKRSTVAQLVQILSEANALEYSILVAATASDPAPLQFLAPYSGCAMGEYFRDNGMHALIIYDDLSKQAVAYRQMSLLLRRPPGREAFPGD : 300
YP 007516887 : TLYCVYVAIGQKRSTVAQLVQILSEANALEYSILVAATASDPAPLQFLAPYSGCAMGEYFRDNGMHALIIYDDLSKQAVAYRQMSLLLRRPPGREAFPGD : 300
YP 006460147 : TLYCVYVAIGQKRSTVAQLVQILSEANALEYSILVAATASDPAPLQFLAPYSGCAMGEYFRDNGMHALIIYDDLSKQAVAYRQMSLLLRRPPGREAFPGD : 300
YP 005090418 : ILYCVYVAIGQKRSTVAQLVQILSEANALEYSILVAATASDPAPLQFLAPYSGCAMGEYFRDNGMHALIIYDDLSKQAVAYRQMSLLLRRPPGREAFPGD : 300
YP 005090487 : TLYCVYVAIGQKRSTVAQLVQILSEANALEYSILVAATASDPAPLQFLAPYSGCAMGEYFRDNGMHALIIYDDLSKQAVAYRQMSLLLRRPPGREAFPGD : 300
YP 003875497 : TLYCVYVAIGQKRSTVAQLVQILSEANALEYSILVAATASDPAPLQFLAPYSGCAMGEYFRDNGMHALIIYDDLSKQAVAYRQMSLLLRRPPGREAFPGD : 300
NP 064105    : TLYCVYVAVGQKRSTVAQLVQILSEANALEYSILVAATASDPAPLQFLAPYSGCAMGEYFRDNGMHALIIYDDLSKQAVAYRQMSLLLRRPPGREAFPGD : 300
               tLYCVYVA6GQKRSTVAQLVQILSEANALEYSILVAATASDPaPLQFLaPYSGCAMGEYFR1NGMHALIIYDDLSKQAVAYrQMSLLLRRPPGREAFPGD
```

```
                       *       320         *       340         *       360         *       380         *       400
YP 009243651 : VFYLHSRLLERAAKRSDQTGAGSLTALPVIETQAGDVSAYIPTNVISITDGQICLETELFYRGIRPAINVGLSVSRVGSAAQLKAMKQVCGSLKLELAQY : 400
YP 009237616 : VFYLHSRLLERAAKRSDQTGAGSLTALPVIETQAGDVSAYIPTNVISITDGQICLETELFYRGIRPAINVGLSVSRVGSAAQLKAMKQVCGSLKLELAQY : 400
XP 011101782 : VFYLQSRLLERAAKRSDQTGAGSLTALPVIQTQAGDVSAYIPTNVISITDGQICLETELFYRGIRPAINVGLSVSRVGSAAQLKAMKQVCGSLKLELAQY : 400
ATP1 SAMI     : VFYLHSRLLERAAKRSDQTGAGSLTALPVIETQAGDVSAYIPTNVISITDGQICLETELFYRGIRPAINVGLSVSRVGSAAQLKAMKQVCGSLKLELAQY : 400
YP 008964126 : VFYLHSRLLERAAKRSDQTGAGSLTALPVIETQAGDVSAYIPTNVISITDGQICLETELFYRGIRPAINVGLSVSRVGSAAQLKAMKQVCGSLKLELAQY : 400
YP 007516887 : VFYLHSRLLERAAKRSDQTGAGSLTALPVIETQAGDVSAYIPTNVISITDGQICLETELFYRGIRPAINVGLSVSRVGSAAQLKAMKQVCGSLKLELAQY : 400
YP 006460147 : VFYLHSRLLERAAKRSDQTGAGSLTALPVIETQAGDVSAYIPTNVISITDGQICLETELFYRGIRPAINVGLSVSRVGSAAQLKAMKQVCGSLKLELAQY : 400
YP 005090418 : VFYLHSRLLERAAKRSDQTGAGSLTALPVIETQAGDVSAYIPTNVISITDGQICLETELFYRGIRPAINVGLSVSRVGSAAQLKAMKQVCGSLKLELAQY : 400
YP 005090487 : VFYLHSRLLERAAKRSDQTGAGSLTALPVIETQAGDVSAYIPTNVISITDGQICLETELFYRGIRPAINVGLSVSRVGSAAQLKAMKQVCGSLKLELAQY : 400
YP 003875497 : VFYLHSRLLERAAKRSDQTGAGSLTALPVIETQAGDVSAYIPTNVISITDGQICLETELFYRGIRPAINVGLSVSRVGSAAQLKAMKQVCGSLKLELAQY : 400
NP 064105     : VFYLHSRLLERAAKRSDQTGAGSLTALPVIETQAGDVSAYIPTNVISITDGQICLETELFYRGIRPAINVGLSVSRVGSAAQLKAMKQVCGSLKLELAQY : 400
               VFYLhSRLLERAAKRSDQTGAGSLTALPVI2TQAGDVSAYIPTNVISITDGQICLETELFYRGIRPAINVGLSVSRVGSAAQLKAMKQVCGSLKLELAQY

                       *       420         *       440         *       460         *       480         *       500
YP 009243651 : REVAAFAQFGSDLDAATQALLNRGARLTEVLKQPQYAPLPIEKQILVIYAAVNGFCDRMPLDRISQYEKAILSSVKPELLQSLLEKGGLTNERKMELDAF : 500
YP 009237616 : REVAAFAQFGSDLDAATQALLNRGARLTEVLKQPQYAPLPIEKQILVIYAAVNGFCDRMPLDKIAQYERDILSTIKPELLESL--KGGLTGERKIEPDAF : 498
XP 011101782 : REVAAFAQFGSDLDAATQALLNRGARLTEILKQPQYTPLPIEKEILVIYAAVNGFCDRMPLDKIAQYEKEILSTVKPELLESL--KGSLNKEKKLEIDTF : 498
ATP1 SAMI     : REVAAFAQFGSDLDAATQALLNRGARLTEILKQPQYTPLPIEKQILVIYAAVNGFCDRMPLDKIAQYEKILLSTVKPELLEAL--KGQLTKEKK------ : 492
YP 008964126 : REVAAFAQFGSDLDAATQALLNRGARLTEILKQPQYTPLPIEKQILVIYAAVNGFCDRMPLDKIAQYEKRLLSTVKADLLEDL--KEPLTKEKKRELDTF : 498
YP 007516887 : REVAAFAQFGSDLDAATQALLNRGARLTEVLKQPQYAPLPIEKQILVIYAAVNGFCDRMPLDKIPQYERDILTTIKPELLQSL--KGGLTSERKIELEKF : 498
YP 006460147 : REVAAFAQFGSDLDAATQALLNRGARLTEILKQPQYTPLPIEKQILVIYAAVNGFCDRMPLDKIAQFEKMLLSTVKPELLESL--KGNLTKEKKLEIDTL : 498
YP 005090418 : REVAAFAQFGSDLDAATQALLNRGARLTEILKQPQYTPLPIEKQILVIYAAVNGFCDRMPLDKISQYEKMLLSTVKPELLESL--KGSLTKEKKLEIDTF : 498
YP 005090487 : REVAAFAQFGSDLDAATQALLNRGARLTEVLKQPQYAPLPIEKQILVIYAAVNGFCDRMPLDKIAQYERDILSTIKPELLQSL--KGILTSERKIELDAF : 498
YP 003875497 : REVAAFAQFGSDLDAATQALLNRGARLTEVLKQQQYAPLPIEKQIIVIYAAVNGFCDRMPLDKISQYERTILNSVNPEFLQSL--KGGLTNEKKMELDSF : 498
NP 064105     : REVAAFAQFGSDLDAATQALLNRGARLTEVLKQPQYAPLPIEKQILVIYAAVNGFCDRMPLDKISQYERTILNSVKPELLQSL--KGGLTNEKKMELDSF : 498
               REVAAFAQFGSDLDAATQALLNRGARLTE6LKQpQY PLPIEK2I6VIYAAVNGFCDRMPLD4I Q5E4 6L 36kpelL2sL  Kg Lt E4K e d f

                       *
YP 009243651 : LKENALPYL------ : 509
YP 009237616 : LKEKALTN------- : 506
XP 011101782 : LKECALSI------- : 506
ATP1 SAMI     : --------------- :   -
YP 008964126 : LKEAEKGI------- : 506
YP 007516887 : LKEKGGTYYI----- : 508
YP 006460147 : LKECALSLK------ : 507
YP 005090418 : LKECALALA------ : 507
YP 005090487 : LKEKANS-------- : 505
YP 003875497 : LKECALNYLKNKSEV : 513
NP 064105     : LKECALNY------- : 506
               lke
```

Figure S3.2 The amino acid sequence allignment of ATP4 in mitogenome of *S. miltiorrhiza* and the corresponding homologs in other species

```
                        *        20         *        40         *        60         *        80         *
XP 002535062 : MDPIKYFTFSMIISILGIRGILLNRRNIPIMSMPIESMLLAVNLNFLVFSVSSDDMMGQSFASLVPTVAAAESAIGLAIFVITFRVRGTI :  90
YP 009041153 : -------------------------------------------------------------------------------------MR--- :   2
ATP4 SAMI    : -------------------------------------------------------------------------------------MR--- :   2
YP 006460176 : -------------------------------------------------------------------------------------MR--- :   2
YP 006291844 : -------------------------------------------------------------------------------------MR--- :   2
YP 005090408 : -------------------------------------------------------------------------------------MR--- :   2
YP 004935350 : -------------------------------------------------------------------------------------MR--- :   2
YP 004849325 : -------------------------------------------------------------------------------------MR--- :   2
YP 004842186 : -------------------------------------------------------------------------------------MR--- :   2
YP 004237263 : -------------------------------------------------------------------------------------MR--- :   2
YP 002608365 : -------------------------------------------------------------------------------------MR--- :   2
                                                                                                    6R

                       100         *       120         *       140         *       160         *       180
XP 002535062 : AVEFINSIQGSGPFSLGELSSTNMQARKMLFAAILSICALSSKKISIYNEEMIVARCFIGFIIFSRKSLGNTFKVTLDGRIQAIQEESQQ : 180
YP 009041153 : ------------------LSSTNMQARKMLFAAILSICASSSKKISIYNEEMIVAACFIGFIIFSRKSLGNTLKVTLDGRIQAIQEESQQ :  74
ATP4 SAMI    : ------------------SCSTNMQARKMLFAAILSICASSSKKISIYNEEMIVARCFIGFIIFSRKSLGKTFKMTLDGRIQAIQEESQQ :  74
YP 006460176 : --------------------STNMQARKMLFAAILSICASSSKKISIYNEEMIVARCFIGFILFSRKSLGKTFKMTLDGRIQAIQEESQQ :  72
YP 006291844 : ------------------LISTNMQARKMLFAAIPSICASSSKKISIYNEEMIVARCFIGFIIFSRKSLGKTFKVTLDGRIQAIQEESQQ :  74
YP 005090408 : ------------------LSSTNMQARKMLFAAILSICASSSKKISIYNEEIIVARCFIGFIIFSRKSLGNTFKVILDGRIQAIQEELQQ :  74
YP 004935350 : ------------------KSSTKMQARKMLFAAILSICASSSKKISIYNEEMIVARCFIGFIIFSRKSLGKTFKVTLDERIQAIQEESQQ :  74
YP 004849325 : ------------------WSSTNMQARKMLFAAILSICALSSKKISIYNEEMIVASSFIGFIILSRKSLGKTFKATLDGRIQAIQEESQQ :  74
YP 004842186 : ------------------KSSTKMQARKMLFAAILSICASSSKKISIYNEEMIVARCFIGFIIFSRKSLGNTFKVTLDERIQAIQEESQQ :  74
YP 004237263 : ------------------LSSTNMQARKMLFAAILSICALSSKKISIYNEEMIVARCFIGFIIFSRKSLGNTFKVTLDGRIQAIQEESQQ :  74
YP 002608365 : ------------------LSSTNMQARKMLFAAIPSICASSSKKISIYNEEMIVARCFIGFIILSRKSLGKTLKVTLDGRIQAIQEESQQ :  74
                                   STnMQARKMLFAAIlSICA SSKKISIYNEE6IVArcFIGFI6fSRKSLG TfK tLDgRIQAIQEEsQQ

                        *       200         *       220         *       240         *       260         *
XP 002535062 : FLNPNEVVPPESNEQQRLLRISLRICGTVVESLPMARCAPKCEKTVQALLCRNLNVKSATLSNATSSRRIRLQDDLVTGFHFSVSERFFP : 270
YP 009041153 : FLNPNEVVLPESNEQQRLLRISLRICGTVVESLPMARCAPKCEKTVQALLCRNLNVKSATLPNATSSRRIRLQDDLVTGFHFSVSERFEP : 164
ATP4 SAMI    : FPNPNEVVPPESNEQQRLLRISLRICGTVVESLPMARCAPKCEKTVQAFLCRNLNVKSATLPNATSSRRIRLQDDLVTAFHLSVSERFVP : 164
YP 006460176 : FPNPNEVVPPESNEQQRLLRISLQICGTVVESLPMARCAPKCEKTVQAFLCRNLNVKSATLPNATSSRRIRLQDDLVTGFHFSVSERFVP : 162
YP 006291844 : FPNPNEVVPPESNEQQRLLRISLRICGTVVESLPMARCAPKCEKTVQALLCRNLNVKSATLPNATSSRRIRLQDDLVTGFHFSVSERFVP : 164
YP 005090408 : FLNPNEVVLLESNEQQRLLRISLRICGTVVESLPMARCAPKCEKTVQAFLCRNLNVKSATLPNATSSRRIRLQDDLVTGFHFSVSERFVP : 164
YP 004935350 : FPNPNEVVPPESNEQQRLLRISLRICGTVVESLPMARCAPKCEKTVQALLCRNLNVKSATLPNATSSRRTRLQDDLVTGFHFSVSERFVP : 164
YP 004849325 : FPNPNEVVPEESNEEQRLLRISLRICGTVVESLPMARYAPKCEKTVQAFLCRNLNVKSATLPNATSSRRIRLQDDLVTGFHFSVSERFVP : 164
YP 004842186 : FPNPNEVVPPESNEQQRLLRVSLRICGTVVESLPMARCAPKCEKTVQALLCRNLNVKSATLPNATSSRRTRLQDDLVTGFHFSVSERFVP : 164
YP 004237263 : FLNPNEVVPPESNEQQRLLRISLRICGTVVESLPMARCAPKCEKTVQALLCRNLNVKSATLSNATSSRRIRLQDDLVTGFHFSVSERFFP : 164
YP 002608365 : FPNPNEVVPPESNEQQRLLRISLRICGTVVESLPMARCAPKCEKTVQALLCRNLNVKSATLPNATSSRRIRLQDDLVTGFHFSVSERFVP : 164
               F NPNEVVppESNE2QRLLR6SLrICGTVVESLPMARcAPKCEKTVQA LCRNLNVKSATLpNATSSRRiRLQDDLVTgFHfSVSERF P
```

```
                          280         *       300
XP 002535062 : GCTLKAS--IVELIREGLVVLRMVRVGGSLKNKEDE : 304
YP 009041153 : GSTLKAS--IVELIREGLVVLRMVRVGGSLKNKEDE : 198
ATP4 SAMI    : GSTLKVS--IVELIREGLGVLKMVRVGGSLKNKENE : 198
YP 006460176 : GSTLKVS--IVELIREGLGVLKMVRVGGSLNNKENE : 196
YP 006291844 : GSTLKAS--IVELIREGLVVLRMVRVGGFS------ : 192
YP 005090408 : GTTLRASIAIVELIREGLGVLKMVRVGGSLKNKEDE : 200
YP 004935350 : GSTLKAS--IVELIREGLAVLRMVRVGGSLKNKEDK : 198
YP 004849325 : GSTLKAS--IVELIREGLVVLRMVRVGGSLKNKSDK : 198
YP 004842186 : GSTLKAS--IVELIREGLAVLRMVRVGGSLKNKEDK : 198
YP 004237263 : GCTLKAS--IVELIREGLVVLRMVRVGGSLKNKEDE : 198
YP 002608365 : GCTLKAS--IVELIREGLAVLRMVRVGGSLKNKEDE : 198
               G TL4aS  IVELIREGL VL4MVRVGGslknke
```

Figure S3.3 The amino acid sequence allignment of ATP6 in mitogenome of *S. miltiorrhiz*a and the corresponding homologs in other species

```
                        *        20         *        40         *        60         *        80         *
YP 009270664 : ------------------------------------------------------------------------------------------ :   -
YP 009045813 : -----------------------------------------------MKSSASSADTSG------IYEDHPGLNPNDER----------- :  26
YP 008999577 : --------------------------------------------------MRVVKDYY---------VTIERTVTDN--------AIIFD :  23
ATP6 SAMI    : ----------------------------------------MAENAAEIPPQEAFKDFVDE-----EREALDKNQADDRP------IERDE :  39
YP 006460168 : ----------------------------------------------MFNVFKTIGNWIGS-----VFNNFRRIFRKDENSLHSSSSDTTS :  39
YP 005090404 : MKRRFSETNLNSSSDTFSRIPNPNTTTLTESNPLLSGTQVAGNDTYNNHDFDEVNDYLRSEIFENVKKTFKRNCPDENKLL----SIAED :  86
YP 004935355 : -------------------------------------------------------------------MDYIATDLDTNG----------- :  12
YP 004237270 : ------------------------------------------------------------------------------------------ :   -
YP 004222295 : ------------------------------------------------------------------------------------------ :   -
YP 003875490 : ------------------------------------------------MAEASHAESQNI-----EFMDYIATDLDTNG----------- :  26
NP 064053    : ------------------------------------------------------------------------------------------ :   -

                      100         *       120         *       140         *       160         *       180
YP 009270664 : ----------------------------MIT-----------------PNSPLEQFAIIPLIPMNIGNLYFSFTNSSLFMLLTLSLVLLL :  45
YP 009045813 : -VVELQCTIRENFE----------ALVQNAQNGVSGEALQEVLD-Q--VPSPLEQFAILPLIPMKIGNLYFSFTNPSLFMLLTLSLVLLL : 102
YP 008999577 : YHHYHNNYYYYNLS----------AFAEMQTR--------TQ------APSPLEQFSILPLIPMNIGNLYFSFTNSSLFMLLTLSLVLLL :  89
ATP6 SAMI    : REIEFLNQVAQDLRR--RG-----PLSVYITQILGGG------------RSPLDQFAIVPFYPLQIGKLYFSFTNASFFLLLILSFVLFL : 110
YP 006460168 : TLSNTNATVNESLK----------DLSNLQENGIGGEAYKDVLDIAEIVPSPLEQFSILPLIPMKIGNLYFSFTNPSLFMLLTLSLVLLF : 119
YP 005090404 : LHNYENDFLKEILEDLKNKEGEHYDFAQQQLNKILGGT-PPT------ITSPLDQFEIISWIPIEIGDLYFSFTNSSLFMLLTLSLVLLL : 169
YP 004935355 : ---VASESYREALA----------LIGEVVPN------------------SPLDQFAILPLIPMKLGNLYFSFTNPSLFMLLTLSLVLLL :  71
YP 004237270 : ---------------------------------------MDALDLAGLVPSPLEQFAILPLIPMKIGNLYFSFTNSSLFMLLTLSLVLLL :  51
YP 004222295 : ----------------------------MIT-----------------PNSPLEQFSILPLIPMKIGNLYFSFTNSSLFMLLTLSLVLLL :  45
YP 003875490 : ---VASESYREALA----------LIGEVVPN------------------SPLDQFAILPLIPMKLGNLYFSFTNSSLFMLLTLSLVLLL :  85
NP 064053    : -------MITPN--------------------------------------SPLEQFSILPLIPMKIGNLYFSFTNPSLFMLLTLSLVLLL :  45
                                                                 SPL QF I6pliP6 6G LYFSFTN SlF6LLtLSlVLll

                        *       200         *       220         *       240         *       260         *
YP 009270664 : VHFVTKNGGGNLVPNAWQSLVELIYDFVLNLVNEQIGGLSGNVKQKFFPCILVTFTFLLFCNLQGMIPYSFTVTSHFLITLGLSFSIFIG : 135
YP 009045813 : IHFVTKNGGGKSVPNAWQSLVELIYDFVPNPVNEQIGGLSGNVKQKFFPRISVTFTFSLFRNLQGMIPYSFTVTSHFLITLGLSFSIFIG : 192
YP 008999577 : IHFVTKKGGGNLVPNAWQSLVELIYDFVLNLVNEQIGGLSGNVKQKFFPCILVTFTFLLFCNLQGMIPYSFTVTSHFLITLGLSFSIFIG : 179
ATP6 SAMI    : FHLVTQKGGGKLVGNACQSVLELIYDFVPNLVNEQIGGLSGNVKQKYFPCISVTFTFSLFRNLQGMIPYSFTVTSHFLVTLGLSFSLFIG : 200
YP 006460168 : VHFVTKKGGGNSVPNAWQSLVELIYDFVPNLVNEQIGGLSGNVKQKFFPCISVTFTFSLFRNLQGMIPYSFTVTSHFLITLGLSFSLFIG : 209
YP 005090404 : FYFVTKKGGGKLVPNAWQSLVEILYDFVLNLVNEQIGGLSGNVKQKYFPCISVTFTFSLFRNLQGMIPYSFTVTSHFLITLGLSFSLFIG : 259
YP 004935355 : LHFVTKKGGGNSVPNVWQSLVELIYDFVLNLVNEQIGGLSGNVKQKFFPCILVTFTFLLFRNLQGMIPYSFTVTSHFLITLGLSFSIFIG : 161
YP 004237270 : VHFVTKKGGGNLVPNACQSLVELIYDFVLNLVNEQIGGLSGNVKQKFFPCILVTFTFLLFCNLQGMIPYSFTVTSHFLITLGLSFSIFIG : 141
YP 004222295 : LHFVTKKGGGNLVPNVWQSLVELIYDFVLNLVNEQIGGLSGNVKQKFFPCILVTFTFLLFCNLQGMIPYSFTVTSHFLITLGLSFSIFIG : 135
YP 003875490 : LHFVTKKGGGNLVPNVWQSLVELIYDFVLNLVNEQIGGLSGNVKQKFFPCILVTFTFLLFCNLQGMIPYSFTVTSHFLITLGLSFSIFIG : 175
NP 064053    : LHFVTKKGGGNSVPNVWQSLVELIYDFVLNLVNEQIGGLSGNVKQKFFPCILVTFTFLLFRNLQGMIPYSFTVTSHFLITLGLSFSIFIG : 135
                hfVTkkGGG  VpN wQS66E66YDFV NlVNEQIGGLSGNVKQK5FPcI VTFTF LF NLQGMIPYSFTVTSHFL6TLGLSFS6FIG
```

```
                        280         *        300         *        320         *        340         *        360
YP 009270664 : ITIVGFQRNGLHFLSLLLPAGVPLPLAPFLVLLELISYCFRALSLGIRLFANMMAGHSLVKILSGFAWTMLCMNDILYFIGDLGPLFIVL : 225
YP 009045813 : ITIVGFQKNGLHFLSFLLPAGVPLPLAPFLVLLELIPHCFRALSSGIRLFANMMAGHSSVKILSGSAWTMLCMNDLLYFIGDLGPLFIVL : 282
YP 008999577 : ITIVGFQRNGLHFLSFLLPAGVPLPLAPFLVLLELISYCFRALSLGIRLFANMMAGHSLVKILSGFAWTMLCMNDLLYFIGDLGPLFIVL : 269
ATP6 SAMI     : ITIVGFAKNGLHFLSFSLPAGVPLPLAPFLVLLELIPHCFRALSLGIRLFANMMAGHSLVKILSGFAWTMLCMNDLFYFIGSTGPFFIVL : 290
YP 006460168 : ITIVGFQKNGLHFLSFSLPAGVPLPLAPFLVLLELIPHCFRALSLGIRLFANMMAGHSSVKILSGSAWTMLCMNDLFYFIGDPGPLFIVL : 299
YP 005090404 : ITIVGFQRNGLHFLSFSLPAGVPLPLAPFLVLLELISYCFRALSLGIRLFANMMAGHSLVKILSGFAWTMLCMNNLFYFIGDLGPLFIVL : 349
YP 004935355 : ITIVGFQRNGLHFLSFSLPAGVPLPLAPFLVLLELIPHCFRALSLGIRLFANMMAGHSSVKILSGFAWTMLCMNDLLYFIGDLGPLFIVL : 251
YP 004237270 : ITIVGFQKNGLHFLSFSLPAGVPLPLAPFLVLLELIPHCFRALSSGIRLFANMMAGHSSVKILSGFAWTMLCMNDLFYFIGDPGPLFIVL : 231
YP 004222295 : ITIVGFQRNGLHFLSFLLPAGVPLPLAPFLVLLELISYCFRALSLGIRLFANMMAGHSLVKILSGFAWTMLCMNDLLYFIGDLGPLFIVL : 225
YP 003875490 : ITIVGFQRNGLHFLSFLLPAGVPLPLAPFLVLLELISYCFRALSLGIRLFANMMAGHSLVKILSGFAWTMLCMNDLLYFIGDLGPLFIVL : 265
NP 064053     : ITIVGFQRNGLHFLSFSLPAGVPLPLAPFLVLLELIPHCFRALSSGIRLFANMMAGHSSVKILSGFAWTMLCMNDLLYFIGDLGPLFIVL : 225
               ITIVGFq4NGLHFLSf LPAGVPLPLAPFLVLLELI  CFRALS GIRLFANMMAGHS VKILSGfAWTMLCMN16 YFIGd GPlFIVL

                       *        380         *        400         *        420
YP 009270664 : ALTGLELGVAILQAYVFTILICIYLNDAINLH----------------------------------- : 257
YP 009045813 : ALTGLELGVAISQAYVSTILICIYLNDATNLHQ---------------------------------- : 315
YP 008999577 : ALTGLELGVAILQAYVFTILICIYLNDAINLH----------------------------------- : 301
ATP6 SAMI     : ALTGLELGVAILQAYVFTILICIYLNDAINLH----------------------------------- : 322
YP 006460168 : ALTGLELGVAISQAHVSTISICIYLNDATNLH----------------------------------- : 331
YP 005090404 : ALTGLELGVAISQAHVSTISICIYLNDATNLHQSYLFIIEQKRGTKFTKFTRTGSKKSREKKSILE : 415
YP 004935355 : ALTGLELGVAILQAHVFTILICIYLNDATNLH----------------------------------- : 283
YP 004237270 : ALTGLELGVAILQAHVSTISICIYLNDATNLH----------------------------------- : 263
YP 004222295 : ALTGLELGVAILQAYVFTILICIYLNDAINLH----------------------------------- : 257
YP 003875490 : ALTGLELGVAILQAYVFTILICIYLNDAINLH----------------------------------- : 297
NP 064053     : ALTGLELGVAILQAHVFTILICIYLNDATNLH----------------------------------- : 257
               ALTGLELGVAI QA V TI ICIYLNDA NLH
```

Figure S3.4 The amino acid sequence allignment of ATP8 in mitogenome of *S. miltiorrhiza* and the corresponding homologs in other species

```
                         *        20         *        40         *        60         *        80         *
YP 009270684 : -----------------------------------------MPQLDKFTYFTQFFWLCLLFFTFYISICNDGDGVLGISRILKLRNQLVSHRGN :  53
YP 009121974 : -----------------------------------------MPQLDKFTYFTQFFWSCLFLFTFYIPICNDGDGVLGISRILKLRNQLVSHREN :  53
XP 011101780 : MLSVGQQPTAALVSFFTTRTDLTELSCLEGIIGSQSIMPQLDKFTYFTQFFWSCLFLFTFYIAICNNGDGVLGISRILKLWNXLVSHRET :  90
YP 009049738 : -----------------------------------------MPQLDKFTYFTQFFWSCLFLFTFYIPICNDGDGVLGISRILKLRNQLVSHREN :  53
YP 008999597 : -----------------------------------------MPQLDKFTYFTQFFWSCLFLFTFYIPICNDGDGVLGISRILKLRNQLVSHREN :  53
YP 008999555 : -----------------------------------------MPQLDKFTYFTQFFWSCLFLFTFYIAICNDGDGLLGISRILKLRNQLLSHRTN :  53
ATP8 SAMI     : -----------------------------------------MPQLDKFTYFTQFFWSCLFLFTFYIAICNDGDGVLGISRILKLRNQLVSHRET :  53
YP 006460155 : -----------------------------------------MPQLDKFTYFTQFFWSCLFLFTFYIAICNDGDGVLGISRILKLRNQLVSHRET :  53
YP 005090417 : -----------------------------------------MPQLDKFTYFTQFFWSCLFLFTFYIAICNDGDGVLGISRILKLRNQLVSHREN :  53
YP 002608379 : -----------------------------------------MPQLDKFTYFTQFFWSCLFLFTFYIPICNDGDGVLGISRILKLRNQLVSHRGN :  53
YP 173455     : -----------------------------------------MPQLDKFTYFTQFFWSCLFLFTFYIPICNDGDGVLGISRILKLRNQLVSHREN :  53
                                                         MPQLDKFTYFTQFFWsCLflFTFYI ICN1GDG6LGISRILKLrNqL6SHR

                        100         *       120         *       140         *       160         *       180
YP 009270684 : NIRSNDPNSLEDILRKGFSTGVSYMYSSLFEVSQWCNAVDLLGKRRKITLISCFGEISGSRGMERNIFYLISKSSYSTSSNPGWGITCRN : 143
YP 009121974 : KIRSNDPNSLEDILRKGFSTGVSYMYSSLFEVSQWCNAVDFLGKRRKMTLISCFGEISGSRGMERNILYLISKS---VHSNPGWVITCRN : 140
XP 011101780 : NIRSNDSKSLEDILRKGFSTGVSYMYSSLFEVSQWCNAVDLLGKRKKITLISCFGELSGSRGMERNIFYLISKSSYSTSSNPGWGITCRN : 180
YP 009049738 : KIRSNDPNSLEDILRKGFSTGVSYMYSSLFEVSQWCNAVDFLGKRRKMTLISCFGEISGSRGMERNILYLISKS---VHSNPGWVITCRN : 140
YP 008999597 : NMRSNDPKSLENILKKGFSTGVSYMYSSLFEVSQWCNAVDLLGKKRKITLISCFGEISGSRGMERNIFYLISKSSYSTSSNPEWGITCRN : 143
YP 008999555 : NIRSKDPNSLEDILRKGFSTGLSYMYSSLFEVSQWCKAVDLLGKRRKITLISCFGEISGSRGMERNIFYLISKSSYSTSSNPGWGITCRN : 143
ATP8 SAMI     : NIRSNDPKSLEDILRKGFSTGVSYMYSSLFEVSQWCNAVDLLGKRKKITLISCFGELSGSRGMERNIFYLISKSSYSTSSNPGWGITCRN : 143
YP 006460155 : DIRSNDPKSLEDILRKGFSTGVSYMYSSLFEVSQWCNAVDLLGKRKKITLISCFGELSGSRGMERNIFYLISKSSYSTSFNPGWGITCRN : 143
YP 005090417 : NIRRNDPNSLEDILRKGFSTGVSYMYSSLFEVSQWCNAVDLLGKSKKITLISCFGELSGSRGMERNIFYLISKSSYSTSSNPGWGITCRN : 143
YP 002608379 : NIRSNDPNSLEDILRKGFSTGVSYMYSSLFEVSQWCNAVDLLGKRSQITLISCFGEISGSRGMERNIFYLISKSSYSTSSNPGWGITCRN : 143
YP 173455     : KIRSNDPNCLEDILRKGFSTGVSYMYSSLFEVSQWCNAVDFLGKRRKMTLISCFGEISGSRGMERNILYLISKS---VHSNPGWVITCRN : 140
                6RsnDp sLE1IL4KGFSTG6SYMYSSLFEVSQWCnAVD LGK  k6TLISCFGE6SGSRGMERNI YLISKS     sNPgW ITCRN

                         *
YP 009270684 : DIMLIHVLHGQGSIVF : 159
YP 009121974 : DIMLIHVPHGQGGIGF : 156
XP 011101780 : DANPMHVLHGQGSIGF : 196
YP 009049738 : DIMLIHVPHGQGSIGF : 156
YP 008999597 : DIMLIHVPHGQGSIVF : 159
YP 008999555 : DIMLIHVPHGQGSIGF : 159
ATP8 SAMI     : DIMLMHVLHSQGSIGF : 159
YP 006460155 : DIMLMHVLHGQGSIGF : 159
YP 005090417 : DIMLIHVLHGQGSIGF : 159
YP 002608379 : DIMLIHVPHGQGSIVF : 159
YP 173455     : DIMLIHVPHGQGSIGF : 156
                Diml6HV HgQGsI F
```

Figure S3.5 The amino acid sequence allignment of ATP9 in mitogenome of *S. miltiorrhiza* and the corresponding homologs in other species

```
                        *        20         *        40         *        60         *        80         *
XP 016207714 : MSNLSKSVCQVSKFKRNENKHGMTRMEKFEMLEGAKSMGAGAATIASVGAAVGIGNVFSSLIHSVARNPSLAKQLFGYAILGFALTEAIA :  90
YP 009047529 : ------------------------------MLEGAKLIGAGAATIALAGAAVGIGNVFSSSIHSVARNPSLAKQLFGYAILGFALTEAIA :  60
WP 028130702 : ------------------------------MLEGAKLIGAGAATIALAGAAIGIGNVFSSLIHSVARNPSLAKQLFGYAILGFALTEAIA :  60
YP 008816086 : ------------------------------MLEGAKLIGAGCATIALAGAAVGIGNVFSSLITSVARNPSLAKQLFGYAILGFALTEAIA :  60
YP 005090413 : ------------------------------MLEGAKSMGAGAATIALAGAAVGIGNVFSSLIHSVARNPSLAKQLFGYAILGFALTEAIA :  60
YP 004935338 : ------------------------------MLEGAKLIGAGAATIASAGAAIGIGNVFSSLIHSVARNPSLAKQLFGYAILGFALTEAIA :  60
YP 004927713 : ------------------------------MLEGAKLIGAGAATIALAGAAVGIGNVFSSLIHSVARNPSLAKQLFGYAILGFALTEAIA :  60
YP 588368     : ------------------------------MLEGAKLIGAGAATIALAGAAVGIGNVFSSLIHSVARNPSLAKQLFGYAILGFALTEAIA :  60
NP 943666     : ----------------------------MAMLEGAKLIGAGCATIALAGAAVGIGNVFSSLIHSVARNPSLAKQLFGYAILGFALTEAIA :  62
NP 054462     : ------------------------------MLEGAKLIGAGAATIALAGAAVGIGNVFSSLINSVARNPSLAKQLFGYAILGFALTEAIA :  60
ATP9 SAMI     : ------------------------------MLEGAKLIGAGAATIALAGAAVGIGNVFSSLIHSVARNPSLAKQLFGYAILGFALTEAIA :  60
                                             MLEGAKl6GAGaATIAlaGAA6GIGNVFSSlIhSVARNPSLAKQLFGYAILGFALTEAIA

                      100
XP 016207714 : LFALMMAILILFVF : 104
YP 009047529 : LFALMMAFLILFVF :  74
WP 028130702 : LFALMMAFLILFVF :  74
YP 008816086 : LFALMMAFLILFVF :  74
YP 005090413 : LFALMMAFLISFVF :  74
YP 004935338 : LFALMMAFLISFVF :  74
YP 004927713 : LFALMMAFLISFVF :  74
YP 588368     : LFALMMAFLILFVF :  74
NP 943666     : LFALMMAFLILFVF :  76
NP 054462     : LFALMMAFLILFVF :  74
ATP9 SAMI     : LFALMMAFLISFVF :  74
               LFALMMAfLI FVF
```

Figure S3.6 The amino acid sequence allignment of ATPB in mitogenome of *S. miltiorrhiza* and the corresponding homologs in other species

```
                       *        20         *        40         *        60         *        80         *       100
ATPB SAMI    : MRMNPTTSGSGVSTLEKKNLGRIIQIIGPVLDVAFPPGQMPNIYNALVVKGRDTVGQPINVTCEVQQLLGNNRVRAVAMSATDGLMRGMEVIDTGAPLSV : 100
YP 009232049 : MRMNPTTSGSGVSTLEKKNLGRIIQIIGPVLDAAFPPGQMPNIYNALVVKGRDTVGQPINVTCEVQQLLGNNRVRAVAMSATDGLMRGMEVIDTGAPLSV : 100
YP 009144522 : MRTNPTTSGSGVSALEKKNLGRIIQIIGPVLDVAFPPGQMPNIYNALVVKGQDTVGQPINVTCEVQQLLGNNRVRAVAMSATDGLMRGMEVIDTGAPLSV : 100
YP 007353922 : MRINPTTSGSGVSTLEKKNLGRIIQIIGPVLDVAFPPGKMPNIYNALVVKGRDTAGQPINVTCEVQQLLGNNRVRAVAMSATDGLMRGMEVIDTGAPLSV : 100
YP 009117228 : MRVNPTTSGSGVSTLEKKNLGRIIQIIGPVLDVAFPPGKMPNIYNALIVKGRDTVGQPINVTCEVQQLLGNNRVRAVAMSATDGLMRGMEVIDTGAPLSV : 100
YP 009270919 : MIINPTTSGSGVSTLEKKNLGRIIQIIGPVLDVAFPPGQMPNIYNALVVKGRDTVGQTINVTCEVQQLLGNNRVRAVAMSATDGLMRGMEVIDTGAPLSV : 100
YP 004935673 : MRINPTTSGSGVSTLEKKNPGRIVQIIGPVLDVAFPPGKMPNIYNALVVKGRDTVGQPINVTCEVQQLLGNNRVRAVAMSATDGLMRGMEVIDTGAPLSV : 100
YP 008964041 : MIINPTTSGSGVSTLEKKNLGRIIQIIGPVLDVAFPPGKMPNIYNALVVKGRDTVGQPINVTCEVQQLLGNNRVRAVAMSATDGLTRGMEVIDTGAPLSV : 100
YP 009254222 : MRINPTTSGSGVSTLEKKNLGRIVQIIGPVLDVAFSPGKMPNIYNALVVKGRDTAGQPINVTCEVQQLLGNNRVRAVAMSATDGLTRGMEVIDTGAPLSV : 100
YP 009164582 : MRINPTTSGPGVSTLEKKNLGRVVQIIGPVLDVAFPPGKMPNIYNALVVKGRDTVGQPINVTCEVQQLLGNNQVRAVAMSATDGLMRGMEVIDTGAPLSV : 100
YP 009110609 : MRINPTTSGSGVSTLEKQNLGRIVQIIGPVLDVAFPPGKMPNIYNALVVKGRDTVGQAINVTCEVQQLLGNNRVRAVAMSATDGLMRGMEVIDTGAPLSV : 100
               Mr NPTTSGsGVStLEKkNlGR66QIIGPVLDvAFpPG MPNIYNAL6VKGrDTvGQpINVTCEVQQLLGNNrVRAVAMSATDGLmRGMEVIDTGAPLSV

                       *       120         *       140         *       160         *       180         *       200
ATPB SAMI    : PVGGATLGRIFNVLGEPVDNLGPVDTRTTFPIHRSAPAFIQLDTKLSIFETGIKVVDLLAPYRRGGKIGLFGGAGVGKTVLIMELINNIAKAHGGVSVFG : 200
YP 009232049 : PVGGATLGRIFNVLGEPVDNLGPVDTRTTFPIHRSAPAFIQLDTKLSIFETGIKVVDLLAPYRRGGKIGLFGGAGVGKTVLIMELINNIAKAHGGVSVFG : 200
YP 009144522 : PVGGATLGRIFNVLGEPVDNLGPVDTRTTFPIHRSAPAFIQLDTKLSIFETGIKVVDLLAPYRRGGKIGLFGGAGVGKTVLIMELINNIAKAHGGVSVFG : 200
YP 007353922 : PVGGATLGRIFNVLGEPVDNLGPVDTRTTFPIHRSAPAFIQLDTKLSIFETGIKVVDLLAPYRRGGKIGLFGGAGVGKTVLIMELINNIAKAHGGVSVFG : 200
YP 009117228 : PVGGATLGRIFNVLGEPIDNLGPVDTRTTFPIHRSAPAFIQLDTKLSIFETGIKVVDLLAPYRRGGKIGLFGGAGVGKTVLIMELINNIAKAHGGVSVFG : 200
YP 009270919 : PVGGATLGRIFNVLGEPVDNLGPVDTRTTFPIHRSAPAFIQLDTKLSIFETGIKVVDLLAPYRRGGKIGLFGGAGVGKTVLIMELINNIAKAHGGVSVFG : 200
YP 004935673 : PVGGATLGRIFNVLGEPVDNLGPVDTRTTFPIHRSAPAFIQLDTKLSIFETGIKVVDLLAPYRRGGKIGLFGGAGVGKTVLIMELINNIAKAHGGVSVFG : 200
YP 008964041 : PVGGATLGRIFNVLGEPVDNLGPVDTRTTFPIHRSAPAFIQLDTKLSIFETGIKVVDLLAPYRRGGKIGLFGGAGVGKTVLIMELINNIAKAHGGVSVFG : 200
YP 009254222 : PVGGATLGRIFNVLGEPVDNLGPVDTSTTFPIHRSAPAFIQLDTKLSIFETGIKVVDLLAPYRRGGKIGLFGGAGVGKTVLIMELINNIAKAHGGVSVFG : 200
YP 009164582 : PVGGATLGRIFNVLGEPVDNLGPVDTRTTFPIHRSAPAFIQLDTKLSIFETGIKVVDLLAPYRRGGKIGLFGGAGVGKTVLIMELINNIAKAHGGVSVFG : 200
YP 009110609 : PVGGATLGRIFNVLGEPVDNLGPVDTRTTSPIHRSAPAFIQLDTKLSIFETGIKVVDLLAPYRRGGKIGLFGGAGVGKTVLIMELINNIAKAHGGVSVFG : 200
               PVGGATLGRIFNVLGEP6DNLGPVDTrTTfPIHRSAPAFIQLDTKLSIFETGIKVVDLLAPYRRGGKIGLFGGAGVGKTVLIMELINNIAKAHGGVSVFG

                       *       220         *       240         *       260         *       280         *       300
ATPB SAMI    : GVGERTREGNDLYMEMKESGVINEENIAESKVALVYGQMNEPPGARMRVGLTALTMAEYFRDVNEQDVLLFIDNIFRFVQAGSEVSALLGRMPSAVGYQP : 300
YP 009232049 : GVGERTREGNDLYMEMKESGVINEENIAESKVALVYGQMNEPPGARMRVGLTALTMAEYFRDVNEQDVLLFIDNIFRFVQAGSEVSALLGRMPSAVGYQP : 300
YP 009144522 : GVGERTREGNDLYMEMKESGVINEENIAESKVALVYGQMNEPPGARMRVGLTALTMAEYFRDVNEQDVLLFIDNIFRFVQAGSEVSALLGRMPSAVGYQP : 300
YP 007353922 : GVGERTREGNDLYMEMKESGVINEENIAESKVALVYGQMNEPPGARMRVGLTALTMAEYFRDVNEQDVLLFIDNIFRFVQAGSEVSALLGRMPSAVGYQP : 300
YP 009117228 : GVGERTREGNDLYMEMKESGVINEENIAESKVALVYGQMNEPPGARMRVGLTALTMAEYFRDVNEQDVLLFIDNIFRFVQAGSEVSALLGRMPSAVGYQP : 300
YP 009270919 : GVGERTREGNDLYMEMKESGVINEENIAESKVALVYGQMNEPPGARMRVGLTALTMAEYFRDVNEQDVLLFIDNIFRFVQAGSEVSALLGRMPSAVGYQP : 300
YP 004935673 : GVGERTREGNDLYMEMKESGVINEENIAESKVALVYGQMNEPPGARMRVGLTALTMAEYFRDVNEQDVLLFIDNIFRFVQAGSEVSALLGRMPSAVGYQP : 300
YP 008964041 : GVGERTREGNDLYMEMKESGVINEENIAESKVALVYGQMNEPPGARMRVGLTALTMAEYFRDVNEQDVLLFIDNIFRFVQAGSEVSALLGRMPSAVGYQP : 300
YP 009254222 : GVGERTREGNDLYMEMKESGVINEENIAESKVALVYGQMNEPPGARMRVGLTALTMAEYFRDVNEQDVLLFIDNIFRFVQAGSEVSALLGRMPSAVGYQP : 300
YP 009164582 : GVGERTREGNDLYMEMKESGVINEENIAESKVALVYGQMNEPPGARMRVGLTALTMAEYFRDVNEQDVLLFIDNIFRFVQAGSEVSALLGRMPSAVGYQP : 300
YP 009110609 : GVGERTREGNDLYMEMKESGVINEENIAESKVALVYGQMNEPPGARMRVGLTALTMAEYFRDVNEQDVLLFIDNIFRFVQAGSEVSALLGRMPSAVGYQP : 300
               GVGERTREGNDLYMEMKESGVINEENIAESKVALVYGQMNEPPGARMRVGLTALTMAEYFRDVNEQDVLLFIDNIFRFVQAGSEVSALLGRMPSAVGYQP
```

```
                            *       320         *       340         *       360         *       380         *       400
ATPB SAMI    : TLSTEMGSLQERITSTKEGSITSIQAVYVPADDLTDPAPATTFAHLDATTVLSRGLAAKGIYPAVDPLDSTSTMLQPRIVGEEHYETAQRVKQTLQRYKE : 400
YP 009232049 : TLSTEMGSLQERITSTKEGSITSIQAVYVPADDLTDPAPATTFAHLDATTVLSRGLAAKGIYPAVDPLDSTSTMLQPRIVGEEHYETAQRVKQTLQRYKE : 400
YP 009144522 : TLSTEMGSLQERITSTKEGSITSIQAVYVPADDLTDPAPATTFAHLDATTVLSRGLAAKGIYPAVDPLDSTSTMLQPRIVGEEHYETAQRVKQTLQRYKE : 400
YP 007353922 : TLSTEMGSLQERITSTKEGSITSIQAVYVPADDLTDPAPATTFAHLDATTVLSRGLAAKGIYPAVDPLDSTSTMLQPRIVGEEHYETAQRVKQTLQRYKE : 400
YP 009117228 : TLSTEMGSLQERITSTKEGSITSIQAVYVPADDLTDPAPATTFAHLDATTVLSRGLAAKGIYPAVDPLDSTSTMLQPRIVGEEHYETAQRVKQTLQRYKE : 400
YP 009270919 : TLSTEMGSLQERITSTKEGSITSIQAVYVPADDLTDPAPATTFAHLDATTVLSRGLAAKGIYPAVDPLDSTSTMLQPRIVGEEHYETAQRVKQTLQRYKE : 400
YP 004935673 : TLSTEMGSLQERITSTKEGSITSIQAVYVPADDLTDPAPATTFAHLDATTVLSRGLAAKGIYPAVDPLDSTSTMLQPRIVGEEHYETAQRVKQTLQRYKE : 400
YP 008964041 : TLSTEMGSLQERITSTKEGSITSIQAVYVPADDLTDPAPATTFAHLDATTVLSRGLAAKGIYPAVDPLDSTSTMLQPRIVGEEHYETAQRVKQTLQRYKE : 400
YP 009254222 : TLSTEMGSLQERITSTKEGSITSIQAVYVPADDLTDPAPATTFAHLDATTVLSRGLAAKGIYPAVDPLDSTSTMLQPRIVGEEHYETAQRVKQTLQRYKE : 400
YP 009164582 : TLSTEMGSLQERITSTKEGSITSIQAVYVPADDLTDPAPATTFAHLDATTVLSRGLAAKGIYPAVDPLDSTSTMLQPRIVGEEHYETAQRVKQTLQRYKE : 400
YP 009110609 : TLSTEMGTLQERITSTKEGSITSIQAVYVPADDLTDPAPATTFAHLDATTVLSRGLAAKGIYPAVDPLDSTSTMLQPRIVGEEHYETAQRVKQTLQRYKE : 400
               TLSTEMG3LQERITSTKEGSITSIQAVYVPADDLTDPAPATTFAHLDATTVLSRGLAAKGIYPAVDPLDSTSTMLQPRIVGEEHYETAQRVKQTLQRYKE

                            *       420         *       440         *       460         *       480         *
ATPB SAMI    : LQDIIAILGLDELSEEDRLTVARARKIERFLSQPFFVAEVFTGSPGKYVGLAETIKGFQLILSGELDALPEQAFYLVGNIDEATAKAINLEMESNLKK : 498
YP 009232049 : LQDIIAILGLDELSEEDRLTVARARKIERFLSQPFFVAEVFTGSPGKYVGLAETIKGFQLILSGELDGLPEQAFYLVGNIDEATAKAINLEMESNLKK : 498
YP 009144522 : LQDIIAILGLDELSEEDRLTVARARKIERFLSQPFFVAEVFTGSPGKYVGLAETIKGFQLILSGELDGLPEQAFYLVGNIDEATAKAINLEMESNLKK : 498
YP 007353922 : LQDIIAILGLDELSEEDRLTVARARKIERFLSQPFFVAEVFTGSPGKYVGLAETIRGFQLILSGELDGLPEQAFYLVGNIDEATAKAMNLEMESNLKK : 498
YP 009117228 : LQDIIAILGLDELSEEDRLTVARARKIERFLSQPFFVAEVFTGSPGKYVGLAETIRGFQLILSGELDGLPEQAFYLVGNIDEATAKAMNLEMESNLKK : 498
YP 009270919 : LQDIIAILGLDELSEEDRLTVARARKIERFLSQPFFVAEVFTGSPGKYVGLAETIKGFQLILSGELDGLPEQAFYLVGNIDEATAKAMNLEMESNLKK : 498
YP 004935673 : LQDIIAILGLDELSEEDRLTVARARKIERFLSQPFFVAEVFTGSPGKYVGLAETIRGFQLILSGELDGLPEQAFYLVGNIDEATAKAMNLEMESNLKK : 498
YP 008964041 : LQDIIAILGLDELSEEDRLTVSRARKIERFLSQPFFVAEVFTGSPGKYVGLAETIRGFQLILSGELDGLPEQAFYLVGNIDEATAKAMNLEMESNLKK : 498
YP 009254222 : LQDIIAILGLDELSEEDRLTVARARKIERFLSQPFFVAEVFTGSPGKYVGLAETIRGFQLILSGELDGLPEQAFYLVGNIDEATAKAINLEMESNLKK : 498
YP 009164582 : LQDIIAILGLDELSEEDRLTVARARKIERFLSQPFFVAEVFTGSPGKYVGLAETIRGFQLILSGELDGLPEQAFFLVGNIDEATAKAMNLEMESNLKK : 498
YP 009110609 : LQDIIAILGLDELSEEDRLTVARARKIERFLSQPFFVAEVFTGSPGKYVGLAETIRGFQLILSGELDGLPEQAFYLVGNIDEATAKAMNLEMESNLKK : 498
               LQDIIAILGLDELSEEDRLTVaRARKIERFLSQPFFVAEVFTGSPGKYVGLAETI4GFQLILSGELDgLPEQAF5LVGNIDEATAKA6NLEMESNLKK
```

Figure S3.7 The amino acid sequence allignment of ATPE in mitogenome of *S. miltiorrhiz*a and the corresponding homologs in other species

```
                        *        20         *        40         *        60         *        80         *       100
YP 007507117 : MTLNLCVLTPNRIVWDSEVKEIILSTNSGQIGVLPNHAPIATAVDIGILRIRLKDQWLTMALMGGFARIGNNEITVLVNDAEKGSDIDPQEAQETLEIAE : 100
YP 009232048 : MTLNLCVLTPNRIVWDSEVKEIILSTNSGQIGVLPNHAPIATAVDIGILRIRLKDQWLTMALMGGFARIGNNEITVLVNDAEKGSDIDPQEAQETLKIAE : 100
YP 009183598 : MTLNLCVLTPNRIVWDSEVKEIILSTNSGQIGVLPNHAPIATAVDIGILRIRLKDQWLTMALMGGFARIGNNEITVLVNDAEKGSDIDPQEAQQTLEIAE : 100
YP 009162267 : MTLNLCVLTPNRIVWDSEVKEIILSTNSGQIGVLPNHAPIATAVDIGILRIRLKDQWLTMALMGGFARIGNNEITVLVNDAERGSDIDPQEAQQTLEIAE : 100
YP 008815943 : MTLNLCVLTPNRIVWDSEVKEIILSTNSGQIGVLPNHAPIATAVDIGILRIRLKDQWLTMALMGGFARIGNNEITVLVNDAEKGSDIDPQEAQQTLEIAE : 100
YP 007353921 : MTLNLCVLTPNRIVWDSEVKEIVLSTNSGQIGVLPNHAPIATAVDIGILRIRLKDQWLTMALMGGFARIGKNEITVLVNDAEKGSDIDPQEAQQTLEIAE : 100
YP 009110608 : MTLNLCVLTPNRIVWDSEVKEIILSTNSGQIGVLPNHAPIATAVDIGILRIRFKDQWLTMALMGGFARIGNNEITVLVNDAEKGSDIDPQEAQQTLEIAE : 100
YP 009166696 : MTLNLCVLTPNRIVWDSEVKEIILSTNSGQIGVLPNHAPIATAVDIGILRIRLKDQWLTIALMGGFARIGNNEITVLVNDAEKGSDIDPQEAQQTLEIAE : 100
YP 009115901 : MTLNLCVLTPNRIVWDSEVKEIILSTNSGQIGILPNHAPIATAVDIGILRIRLKDQWLTMALMGGFARIGNNEITVLVNDAEKGSNIDPQEAQQTLEIAE : 100
YP 009117227 : MTLNLCVLTPNRIVWDSEVKEIILSTNSGQIGVLPNHAPIATAVDIGILRVRLKDQWLTMALMGGFARIGNNEITVLVNDAEKGSDIDPQEAQQALEIAE : 100
ATPE SAMI    : MTLNLCVLTPNRIVWDSEVKEIILSTNSGQIGVLPNHAPIATAVDIGILRIRLKDQWLTMALMGGFARIGNNEITVLVNDAEKGSDIDPQEAQETLEIAE : 100
               MTLNLCVLTPNRIVWDSEVKEI6LSTNSGQIG6LPNHAPIATAVDIGILR6RlKDQWLT6ALMGGFARIGnNEITVLVNDAE4GS1IDPQEAQ2tLeIAE

                        *       120         *
YP 007507117 : ASLRKAEGKRQIIEANLALRRARTRVEAVSAIS : 133
YP 009232048 : ASLRKAEGKRQIIEANLALRRARTRVEAVSAIS : 133
YP 009183598 : ANLRKAEGKRQIIEANLALRRARTRVEAVNAIS : 133
YP 009162267 : ANLRKAEGKRQIIEANLALRRARTRVEAVNAIS : 133
YP 008815943 : ANLRKAEGKRQIIEANLALQRARTRVEAVNAIS : 133
YP 007353921 : ANLRKAEGKRQIIEANLALRRARTRVEAVNAIS : 133
YP 009110608 : ANLRKAEGKRQIIEANLALRRARTRVEAVNVIS : 133
YP 009166696 : ANLKKAEGKRQIIEANLALRRARTRVEAVNAIS : 133
YP 009115901 : ANLRKAEGKRQIIEANLALRRARTRVEAINAIS : 133
YP 009117227 : ANLKKAEGKRQIIEANLALRRARTRVEAVNAIS : 133
ATPE SAMI    : ASLRKAEGKRQIIEANLALRRARTRVEAVSAIS : 133
               A L4KAEGKRQIIEANLALrRARTRVEA6 aIS
```

Figure S3.8 The amino acid sequence allignment of CCMB in mitogenome of *S. miltiorrhiza* and the corresponding homologs in other species

```
                          *        20         *        40         *        60         *        80         *       100
YP 009153927 : MRRLFLELYHKQIFPSTPITSFSPFLSYIVVTPLILGFEKDFSCHFHLGPIRIPPLFSFPPAPFPRNEKEDGTLELYYLSAYCLPKILLLQLVGHRVIQI : 100
YP 009049759 : MRRLFLELYHKQIFPSTPITSFSLFLSYIVVTPLMLGFEKDFSCHSHLGPIRIPPLFPFPSAPFPRNEKEDGTLELYYLSAYCLPKILLLQLVGHRVIQI : 100
YP 009041179 : MRRLFLELYHKQIFPSTPITSFSLFLSYIVVTPLMLGFERDFSCHSHLGPIRIPPLFPFPSAPFPRNEKEDGTLELYYLSAYCLPKILLLQLVGHRVIQI : 100
YP 008999592 : MRRLFLELYHKRIFPSTPITSFSPFLSYIVVTPLMLGFEKDFSCHSHLGPIRIPPLFPFPSAPFPRNEKEDGTLELYNLSAYCLPKILLLQLVGHRVIQI : 100
CCMB SAMI    : MRRLFLELYHKQIFSSTPITSFSLFLSYIVVTPLMLGFEKDFSCHSHLGPIRIPPLFPFPSAPFPRNEKEDGILELYYLSAYCFQKILLLQLVGHRVIQI : 100
YP 008964108 : MKRLFLELYHREIFSCTPIPCFSLFLSYIAVTPLMLGFEKDFSCHSHLGLIRIPPLFPFPSAPFPRNDQEDGILELCYLSAYCFQKILLLQLVSHRVIQI : 100
YP 007516883 : MRRLFLELYNKKIFPSTPITSFSPFLSYIVVTPLILGFEKDFSCHSHLGPIRIPSLFSFPPAPFPRNEKEDGTLELYYLSAYCLPKILLLQLVGHRVIQI : 100
YP 006460163 : MRRLFLELYHKQIFSSTPITSFSLFLSYIVVTPLMLGFEKDFSCHSHLGPIRIPPLFPFPSAPFPRNEKEDGTLELYYLSAYCFQKILLLQLVGHRVIQI : 100
YP 006291818 : MRRLFLSLYHKQIFPSTPITSFSLFLSYIVVTPLMLGFEKDFSCHSHLGPIRIPPLFPFPSAPFPRNDKEDGTLELYYLSAYCLPKILLLQLVGHWVIQI : 100
YP 005090414 : MRRLFLELYHKQIFSYTPITSFSLFLSYIVVTPLMLGFEKDFSCHSHLGPIRIPPLFPFLSAPFPRNDQEDGTLELYYLSAYCFPKILLLQLVGHRVIQI : 100
YP 173483    : MRRLFLELYHKQIFPSTPITSFSLFLSYIVVTPLMLGFEKDFSCHSHLGPIRIPPLFPFPSAPFPRNEKEDGTLELYYLSAYCLPKILLLQLVGHRVIQI : 100
               M4RLFLeLYh4 IF sTPItsFS FLSYIvVTPL6LGFE4DFSCHsHLGpIRIPpLFpFpsAPFPRN kEDGtLELyyLSAYC  KILLLQLVgHrVIQI

                          *       120         *       140         *       160         *       180         *       200
YP 009153927 : SCVFCGFPMLQLPYQFGRSGMDRLNIPLGSLVLTLLCGIHSRSALGITSSSGWNSSQNPTTSPTLLPLTVSRTSIETEWFHVLLSIGYSSPFVSLFPISV : 200
YP 009049759 : SRVFRGFPMLQLPYQFGRSGMDRLNIPLGSLVLTLLCGIHSRSALGITSSSGWNSSQNPTTSPTSLPPTVSRTSIETEWFHVLSSIGYSSPFVSLFPISV : 200
YP 009041179 : SRVFCGFPMLQLLYQFGRSGMDRLNILLGSLVLTLLCGIHSRSALGITSSSGWNSSQNPTTSPTSLPPTLSRTSIETEWFHVLSSIGYSSPFVSLFPIFV : 200
YP 008999592 : SRVFRGFPMLQLPYQFGRSGIDRLNILLGSLVLTLLCGIHSRSALGITSSSGWNSSQNPTTSPTSLPPTLSRTSIETEWFHVFSSIGYSSLFVSLFPISV : 200
CCMB SAMI    : SRVFRGFPMLQLPYQFGRSGMDRLNIPLGSLVLTLLCGIHSRSALGITSSSGWNSSQNPTTSPTSLPPTLSRTSIETEWFHVLSSIGYSSPFVSLFPISV : 200
YP 008964108 : SRVFRGFPMLQLPYQFGRSGMDRLNIPLGSLVLTLLCGIRSRSALGITSSSGWNSSQNPTTSPTSLPPTLSRTSIETEWFHVLSSIGYSSPFVSIFPISV : 200
YP 007516883 : SCVFCGFPMLQLPYQFGRSGMDRLNIPLGSLVFTLLCGIHSRLALGITSSSGWNSSQNPTTSPTSLPPTVSRTSIETEWFHVLSSIGYSSPFVSLFPIAV : 200
YP 006460163 : SRVFRGFPMLQLPYQFGRSGMDRLNIPLGSLVLTLLCGIHSRSALGITSSSGWNSSQNPTTSPTSLPPTLSRTSIETEWFHVLSSIGYSSPFVSLFPISV : 200
YP 006291818 : SRVFCGFPMLQLPYQFGRSGMDRLNIPLGSLVLTLLCGIHSRSALGITSSSGWNSLQNPTTSPTSLPPTLSRTSIETEWFHVLSSIGYSSPFVSLSPILV : 200
YP 005090414 : SRVFRGFPMLELPYQFGLSGIDRLNIPLGSLVLTLLCGIHSCSALGITSSSGWNSSQNPTTSPTSLPPTLSRTSIETEWFHVLSSIGYSSPFVSLFPISV : 200
YP 173483    : SRVFRGFPMLQLPYQFGRSGMDRLNIPLGSLVLTLLCGIHSRSALGITSSSGWNSSQNPTTSPTSLPPTLSRTSIETEWFHVLSSIGYSSPFVSLFPISV : 200
               SrVF GFPML2LpYQFGrSG6DRLNIpLGSLVlTLLCGIhSrsALGITSSSGWNSsQNPTTSPTsLPpT6SRTSIETEWFHVlsSIGYSSpFVS6fPI V

YP 009153927 : SISLQD : 206
YP 009049759 : SISSQD : 206
YP 009041179 : SISSQD : 206
YP 008999592 : SISSQD : 206
CCMB SAMI    : SISSQD : 206
YP 008964108 : SISSQD : 206
YP 007516883 : SMSLQD : 206
YP 006460163 : SISSQD : 206
YP 006291818 : SISSQD : 206
YP 005090414 : SISSQD : 206
YP 173483    : SISSQD : 206
               S6SsQD
```

Figure S3.9 The amino acid sequence allignment of CCMC in mitogenome of *S. miltiorrhiza* and the corresponding homologs in other species

```
                       *        20         *        40         *        60         *        80         *       100
YP 009243656 : MSVSLLQPYFLMSKTRSYAQIIIGSRLFLTAMAIHLSLRVAPLDLQQGGNSRIPYVHVPAARMSILVYIATAINTLFFLLTKHPLFLRSSGTGTEMGAFF : 100
YP 009241679 : MSVSLLQPSFLMSKTRSYAQILIGSRLFLTAMAIHLSLRVAPLDLQQGGNSRIPYVHVPAARMSILVYIATAINTFFFLLTKHPLFLRSSGTGTEMGAFF : 100
XP 015584144 : MSVSLLQPSFLMSKTRSYAQILIGSRLFLTAMAIHLSLRVAPLDLQQGGNSRIPYVHVPAARMSILVYIATAINTFFFLLTKHPLFLRSSGTGTEMGAFF : 100
YP 009173850 : MSISLLQPSFLMSKTRSYSQILIGSRLFLTAMAIHLSLRVAPLDLQQGGNSRIPYVHVPAARMSILVYIATAINTFLFLLTKHPLFLRSSGTGTEMGAFF : 100
YP 009041161 : MPLSLLQPYFLMSKTRSYAQILIGSRLFLTAMAIHLSLRVAPLDLQQGGNSRIPYVHVPAARMSILVYIATAINTFLFLLTKHPLFLRSSGTGIEMGAFF : 100
CCMC SAMI    : MSLSLLQPYFLMSKTRSYAQILIGSRLFLTAMAIHLSLRVAPLDLQQGGNSRIPYVHVPAARMSILVYIATAINTFFFLLTKHPLFLRSSGTGTEMGAFS : 100
YP 006666113 : MSVSLLQPYFLMSKTRSYAQILIGSRLFLTAMAIHLSLRVAPLDLQQGGNSRIPYVHVPAARMSILVYIATAINTFFFLLTKHPLFLRSSGTGTEIGAFF : 100
YP 006460181 : MSLSLLQPSFLMSKTRSYAQILIGSRLFLTAMAIHLSLRVAPLDLQQGGNSRIPYVHVPAARMSILVYIATAINTFFFLLTKHPLLLRSSGTGTEMGAFS : 100
YP 006291836 : MPVSLLQPYFLMSKTRSYAQILIGSRLFLTAMAIHLSLRVAPLDLQQGGNSRIPYVHVPAARMSIIVYIATAINTFLFLLTKHPLFLRSSGTGTEMGAFS : 100
YP 004237264 : MSVSLLQPSFLMSKTRSYAQILIGSRLFLTAMAIHLSLRVAPLDLQQGGNSRIPYVHVPAARMSILVYIATAINTFFFLLTKHPLFLRSSGTGTEMGAFF : 100
YP 173360     : MSLSLLQPSFLMSKTRSYAQILIGSRLFLTAMAIHLSLRVAPLDLQQGGNSRIPYVHVPAARMSILVYIVTAINTFLFLLTKHPLFLRSSGTGTEIGAFS : 100
               Ms6SLLQP FLMSKTRSYaQI6IGSRLFLTAMAIHLSLRVAPLDLQQGGNSRIPYVHVPAARMSI6VYIaTAINTf FLLTKHPLfLRSSGTGtE6GAF

                       *       120         *       140         *       160         *       180         *       200
YP 009243656 : TLFTLVTGGFRGRPMWGTFWVWDARLTSVFISFLIYLGALRFQKLPVEPASISIRAGPIDIPIIKSSVNWWNTSHQPGSISRSGTSIHVPMPIPILSNFA : 200
YP 009241679 : TLFTLVTGGFRGRPMWGTFWVWDARLTSVFISFLIYLGALRFQKLPVEPASISIRAGPIDIPIIKSSVNWWNTSHQPGSISRSGTSIHVPMPIPILSNFA : 200
XP 015584144 : TLFTLVTGGFRGRPMWGTFWVWDARLTSVFISFLIYLGALRFQKLPVEPASISIRAGPIDIPIIKSSVNWWNTSHQPGSISRSGTSIHVPMPIPILSNFA : 200
YP 009173850 : TLFTLVTGGFRGRPMWGTFWVWDARLTSVFISFFIYLGALRFQKLPVEPASISICAGPIDIPIIKSSVNWWNTSHQPGSISRSGTSIHVPMPIPILSNFA : 200
YP 009041161 : TLFTLVTGGFRGRPMWGTFWVWDARLTSVFILFLIYLGALRFQKLSVEPAPISICAGPIDIPIIKSSVNWWNTSHQPGSISRFGTSIHVPMPIPILSNFA : 200
CCMC SAMI    : TLFTLVTGGFRGRLMWGTFWVWDARLTSVFILFLIYLGALCFQKLPVEPAPISIRAGPIDIPIIKSSVNWWNTSHQPGSISRSGTSIHVPMPIPILSNFA : 200
YP 006666113 : TLFTLVTGGFRGRPMWGTFWVWDARLTSVFISFLIYLGALRFQKLPVEPASISIRAGPIDIPIIKSSVNWWNTSHQPGSISRSGTSIHVPMPIPILSNFA : 200
YP 006460181 : TLFTLVTGGFRGRPMWGTFWVWDARLTSVFISFLIYLGALCFQKLPVEPAPISIRAGPIDIPIIKSSVNWWNTSHQPGSISRSGTSIHVPMPIPILSNFA : 200
YP 006291836 : TLFTLVTGGFRGRPMWGTFRVWDARLTSVFISFLIYLGALRFQKLSVEPAPISIRVGPIDIPIIKSSVNWWNTSHQPGSISRSGTSIHVPMPIPILSNFA : 200
YP 004237264 : TLFTLVTGGFRGRPMWGTFWVWDARLTSVFISFLIYLGALRFQKLPVEPASISIRAGPIDIPIIKSSVNWWNTSHQPGSISRSGTSIHVPMPIPILSNFA : 200
YP 173360     : TLFTLVTGGFRGKPMWGTFWVWDARLTSVFILFLIYLGALRFQKLSVEPAPISIRAGPIDIPIIKSSVNWWNTLHQPGSISRSGTSIHVPMPIPILSNFA : 200
               TLFTLVTGGFRG4pMWGTFwVWDARLTSVFI FlIYLGALrFQKL VEPA ISIraGPIDIPIIKSSVNWWNTsHQPGSISRsGTSIHVPMPIPILSNFA

                       *       220         *       240         *
YP 009243656 : NSPFSTRILFVLETRLPIPSFPESPLTDEIEAREGIPKPSSLAESLSIHG : 250
YP 009241679 : NSPFSTRILFVLETRLPIPSFPESPLTEEIEAREGIPKPSSLAEYLCIHG : 250
XP 015584144 : NSPFSTRILFVLETRLPIPSFLESPLTEEIEAREGIPKPSSLAESLCVHG : 250
YP 009173850 : NSPLSTRIFFVLETRLPIPSFLESPLTEEIEARESIPKPSSLADSLCIHG : 250
YP 009041161 : NSPFSTRILFVLETRLPIPSFLESPLTEEIEARERIPKPSSLAESLCIHG : 250
CCMC SAMI    : NSPLSTPILFVLETRLPIPSFLESPLKEEIK------------------- : 231
YP 006666113 : NSPFSTRILFVLETRLPIPSFPESPLTEEIEAREGIPKPSSLAESLCIHG : 250
YP 006460181 : NSPLSTPILFVLETRLPIPSFLESSLKEEIEAREGIPKPSSLAESLCIHG : 250
YP 006291836 : NSPFSTRILFVLETRLPIPSFLESSLTEEIEAREGIPKPSSLAESLCIHG : 250
YP 004237264 : NSPFSTRILFVLETRLPIPSFLESPLTEEIEA------------------ : 232
YP 173360     : NSPFSTRILFVLETRLPIPSFLESPLTEEIEAREGIPKPISLAESFCIHG : 250
               NSP STrIlFVLETRLPIPSF ESpLteEIeare ipkp sla    hg
```

Figure S3.10 The amino acid sequence allignment of CCMFC in mitogenome of *S. miltiorrhiza* and the corresponding homologs in other species

```
                        *        20         *        40         *        60         *        80         *       100
YP 009239002 : MVQLHNFFFFITSMVVPRGTAAPVLLKWFFSRDVPIGAPSSNGTIIPIPISLFPLLIYLHSRKFIRSMDGAKSGVLVRASRPILLPDKIRRSSPETRARN : 100
YP 009230357 : MVQLHNFFFFITSMVVPRGTAAPVLLKWFFSRDVPIGAPSSNGTIIPIPISLFPLLIYLHSRKFIRSMDGAKSGVLVRASRPILLPDKIRRSSPETRARN : 100
YP 009173853 : MVQLHNFFFFITSMVVPRGTAAPVLLKWFFSRDVPIGAPSSNGTIIPIPISLFPLLVYLHSRKFIRSMDGAKSGVLVRASRPILLPDKIRRSSPETRARN : 100
YP 009153929 : MVQLHNFFFFIIFMVVPRGTAAPVLLKWFVSRDVSTGAPFSNGTIIPIPISSFPLLVYLHSRKFIRSMDRAKSGVLVRASRPILLPDIIGRSSSDTRARN : 100
YP 009121946 : MVQLHNFFFFITSMVVPRGTAAPVLLKWFVSRDVPTGALFSNGTIIPIPIPSFPLLVYLHSRKFIRSADGAKSGVLVRASRPILLPDIIGRSSSETRARN : 100
YP 009049753 : MVQLHNFFFFITSMVVPRGTAAPVLLKWFVSRDVPTGALFSNGTIIPIPIPSFPLLVYLHSRKFIRSADGAKSGVLVRASRPILLPDIIGRSSSETRARN : 100
CCMFC SAMI   : MVQLHNFLFLITSMVVPRGTAAPVLLKWFVSRDVPTGAPFSNGTIIPIPIPSFPLLVYLHSRKFIRSTDRAKSRVLVRASRPILLPDIIGRSSSETRARN : 100
YP 008964115 : MVQLQNFFFLITSMVVPRGTAAPVLLKWFVSRDVPTGAPFSNGTLIPIPIPSFPLLVYLHSRKFIRSTDRAKSRVLVRASRPILLPDIIGRSSSETRARN : 100
YP 006460172 : MVQLHNFFFLITSMVVPRGTAAPVLLKWFVSRDVPTGAPFSNGTIIPIPIPSFPLLVYLHSRKFIRSTDRAKSRVLVRASRPILLPDRIGRSSSETRARN : 100
YP 005090424 : MVQLHNFFFLITSMVVPRGTAAPILLKWFVSRDVPTGAPFSNGTQIPIPIPSFPLLVYLHSRKFIRSTDRAKSRVLVRASRPILLPDIIGRSSSETRARN : 100
YP 173470    : MVQLHNFFFFITSMVVPRGTAAPVLLKWFVSRDVPTGALFSNGTIIPIPIPSFPLLVYLHSRKFIRSADGAKSGVLVRASRPILLPDIIGRSSSETRARN : 100
               MVQLhNFfF ItsMVVPRGTAAP6LLKWF SRDVp GA  SNGT IPIPI  FPLL6YLHSRKFIRS D AKS VLVRASRPILLPD I RSS eTRARN

                        *       120         *       140         *       160         *       180         *       200
YP 009239002 : ALFRFVPVLHFLLLESKGDFSYLESFCGVLCLLFFRTFFSLPRDRSAKRERARKRKRQTLRPNGNEQRRNDKMRCSGHPHLKRRVEGFGPLAFPVPPSSG : 200
YP 009230357 : ALFRFVPVLHFLLLESKGDFSYLESFCGVLCLLFFRTFFSLPRDRSAKRERARKRKRQTLRPNGNEQRRNDKMRCSGHPHLKRRVEGFGPLAFPVPPSSG : 200
YP 009173853 : ALFRFVPVLHFLLLESKGDFSYLESFCGVLCLLFFRTFFSLPRDRSAKRERARRRKRQTLRPNGNEQRRNDKMRCSGHPHLKRRVEGFGPLAFPVPPSSG : 200
YP 009153929 : ALFRFVPVLHFLLLESKGDFSYLESFCGVLRLLFFRTFFFLPRDRSAKHERARRRKRQTLRPNGNEQRRNDKMRCSGHPHLERRVEGFGPVAFPVPPSSG : 200
YP 009121946 : ALFRFVPVLHFLLLESKGDFSYLESFCGVLRLLFFRTFFFLPRDRSAKPERARRRKGQTLRPNGNEQRRNEKMRCLGHPHLERRVEGFGPVAFPVPPSSG : 200
YP 009049753 : ALFRFVPVLHFLLLESKGDFSYLESFCGVLRLLFFRTFFFLPRDRSAKPERARRRKGQTLRPNGNEQRRNEKMRCLGHPHLERRVEGFGPVAFPVPPSSG : 200
CCMFC SAMI   : ALFRFVPVLHFLLL---GDNSYLESFCGVLGLLFFRTFFFLPRDRSAKRERARRRKGQTLRPNGNKQGRNEKMRCPGHP--QRRVEAFRPVAFPVPPSSC : 195
YP 008964115 : ALFRFVPVLHFLLLESKGDNSYLESFCGVLGLLFFRTFFFLPRDRSAKRERARRRKGQRLRPNGNKQGRNEKMRCPGHP--QRRVEAFRPVAFPVPPSSC : 198
YP 006460172 : ALFRFVPVLHFLLLESKGDNSYLESFCGVLRLLFFRTLFFLPRDRSAERERARRRKGQRLRPNGNEQRRNEKMRCPGHPHLERRVEAFRPVAFPVPPSPG : 200
YP 005090424 : ALFRFVPVLHFLLLESKGDNSYLESFCGVLRLLFFRTFFFLPRDRSAKRERARRRKGQTLWPNGNDQRRNDKMRCLGHPHLKRRVEAFCPVAFPVPPSSG : 200
YP 173470    : ALFRFVPVLHFLLLESKGDFSYLESFCGVLRLLFFRTFFFLPRDRSAKPERARRRKGQTLRPNGNEQRRNEKMRCLGHPHLERRVEGFGPVAFPVPPSSG : 200
               ALFRFVPVLHFLLLeskGD SYLESFCGVL LLFFRTfF LPRDRSAk ERAR4RK QtLrPNGN QrRN KMRC GHPhl RRVE F P6AFPVPPSsg

                        *       220         *       240         *       260         *       280         *       300
YP 009239002 : GACVGGVPPERGVEALALPTSRQLMA--VGHDYYQKAPMKMNISHGGVCIFMLGVLLSNTKKIQFTQRLPLGSELHMGKERCCLRGLDHLHGPTSHSICG : 298
YP 009230357 : GACVGGVPPERGVEALALPTSRQLMA--VGHDYYQKAPMKMNISHGGVCIFMLGVLLSNTKKIQFTQRLPLGSELHMGKERCCLRGLDHLHGPTSHSICG : 298
YP 009173853 : GACVGGVPPEIGVEALALPTSRQLMA--VGHDYYQKAPMKMNISHGGVCICMLGVLLSNTKKIQFTQRLPLGSELHMGKERCCLRGLDHLHGPTSHSICG : 298
YP 009153929 : GACVGDMPPEIGLEALALPTSRQLMAMAVGHDYYQKAPMKMNISHGGVCICMLGVLLSNTKKIQFTQRLPLGSELHMGKERCCLRGLDHLHAPTFHSICG : 300
YP 009121946 : GACVEGAPPEIGLEALTLPTSRELMA--VGHDYYQKAPMKMNISHGGVCIFMLGVLLTNTKKIQFTQRLPLGSELHMGKERCCLRGLDHLHGPTFHSICG : 298
YP 009049753 : GACVEGAPPEIGLEALTLPTSRELMA--VGHDYYQKAPMKMNISHGGVCIFMLGVLLSNTKKIEFTQRLPLGSELHMGKERCCLRGLDHLHGPTFHSICG : 298
CCMFC SAMI   : GACVGDAPPEIGLEALTLPTNRQLMA--VGHDDYQKAPMKMNISHGGVCIFMLGVLLSNTKKIEFTQRLPLGSELHMGKERCSLRGLDHLHGPTFHSICG : 293
YP 008964115 : GACVGDAPPEIGLEALTLPRSRQRMA--VGHDDYQKAPMKMNISHGGVCIFMLGVLLSNTKKIEFTQRLPLGSELHMGKERCSLRGLDHLHGPTFHSICG : 296
YP 006460172 : GACVGDAPPEIGLEALTLPTSRQLMA--VGHDDYQKAPMKMNISHGGVCILMLGVLLSNTKKIEFTQRLPLGSELHMGKERCCLRGLDHLHGPTFHSICG : 298
YP 005090424 : GAYVGDAPPEIRLETLTLPTSRQLMA--VGHDYYQKAPMKMNISHVGVCIFMLGVLLSYTKKIEFTQRLPLGSELHMGKKRCCLRGIDHLHGPTFHSICG : 298
YP 173470    : GACVEGAPPEIGLEALTLPTSRELMA--VGHDYYQKAPMKMNISHGGVCIFMLGVLLSNTKKIQFTQRLPLGSELHMGKERCCLRGLDHLHGPTFHSICG : 298
               GAcV   PPEig6EaL LPtsR2lMA  VGHD YQKAPMKMNISHgGVCI MLGVLL3nTKKI2FTQRLPLGSELHMGKeRCcLRG6DHLHgPT HSICG
```

```
                        *       320         *       340         *       360         *       380         *       400
YP 009239002 : NLMIYKPSLTNDRLMFEHDESLRADLLLINFPASYENGKLEHFLHRWMKNREHNNFWLTMFPEKRYFRETTSTTEVAIHTNLFTDLYASIGTGSSRTGGW : 398
YP 009230357 : NLMIYKPSLTNDRLMFEHDESLRADLLLINFPASYENGKLEHFLHRWMKNREHNNFWLTMFPEKRYFRETTSTTEVAIHTNLFTDLYASIGTGSSRTGGW : 398
YP 009173853 : NLMIYKPSLTNDRLMFEHDESLRADLLLINFPASYENGKLEHFLHRWMKNREHNNFWLTMFPEKRYFRETTSTTEVAIHTNLFTDLYASIGTGSSRTGGW : 398
YP 009153929 : NLMIYKPSLTNDRLMFEHDESLRADPLLINFPASYENGKLEHFLHRWMKNRKHNNFWLTMFPEKRYFRETTSTTEVAIHTNLFTDLYASIGTGSSRTGGW : 400
YP 009121946 : NLMIYKPSLTSDRLMFEHDESLRADLLPIHFPASYENGKLEHFFHRWMKNREHNNFWLTMFPEKRYFRERTSTTEVAIHTNLFTDLYASIGTGSSRTGGW : 398
YP 009049753 : NLMIYKPSLTSDRLMFEHDESLRADLLPIHFPASYENGKLEHFIHRWMKNREHNNFWLTMFPEKRYFRERTSTTEVAIHTNLFTDLYASIGTGSSRTGGW : 398
CCMFC SAMI   : NLMIYKPSLTNDRLIFEHDESLRADLLPIHFPTSYENGKLEHFLHRWMKNREHNNFWLTMFPEKRYFRERTSTTEVAIHTNLFTDLYALIGTGSSRTGGW : 393
YP 008964115 : NFSIYKPSLTNDRLIFEHDESLRADLLPIHFPTSYENGKLEHFLHRWMKNREHKNFWLTMFPEKRYFRERTSTTEVAIHTNLFTDLYALIGTGSSRTGGW : 396
YP 006460172 : NLVIYKPSLTNDRLIFEHDESLRADLLPIHFPTSYENGKLEHFLHRWMKNREHNNLWLTMFPEKRYFRERTSTTEVAIHTNLFTDLYALIGTGSSRTGGW : 398
YP 005090424 : NFMIYKPSLTNDRLMFEHDESLRADLLPIHFLASYENGKLEHFLHRWMKNREHNNFWLIMFPENRYFRERTSTTEVSIQTNLFTDLYALIGTGSSRTRGW : 398
YP 173470     : NLMIYKPSLTSDRLMFEHDESLRADLLPIHFPASYENGKLEHFFHRWMKNREHNNFWLTMFPEKRYFRERTSTTEVAIHTNLFTDLYASIGTGSSRTGGW : 398
               Nl IYKPSLT DRL6FEHDESLRADlL I Fp SYENGKLEHF HRWMKNReHnNfWLtMFPEkRYFRE TSTTEVaIhTNLFTDLYA IGTGSSRTgGW

                        *       420         *       440         *       460
YP 009239002 : YTTIMKLPFIFFIRIGFMLASLGGSRSLLRQLKK--DKLRWYRESSVEFIIAKKE------------ : 451
YP 009230357 : YTTIMKLPFIFFIRIGFMLASLGGSRSLLRQLKK--DKLRWYRESSVEFIIAKKEVK---------- : 453
YP 009173853 : YTTIMKLPFIFFIRIGFMLASLGGSRSLLRQLKK--DKLRWYRESSVEFIIAKKESK---------- : 453
YP 009153929 : YTTIMKLPFIFFIWIGFMLASLGGSRSLLRQLQK--DKLRWNRESSVEFIIA--------------- : 450
YP 009121946 : YTTIIKLPFIFFIRIGFMLASLGGSRSLLRQLQK--DKLRWN------------------------- : 438
YP 009049753 : YTTIIKLPFIFFIRIGFMLASLGGSRSLLRQLQK--DKLRWN------------------------- : 438
CCMFC SAMI   : YTTIMKLPFYFFIRIGFMLASLGGWHSLLRQLQK--ELLPWN------------------------- : 433
YP 008964115 : YTTIMKLPFIFFIRIGFMLASLGGWHSLLRQLQKEKELLPWN------------------------- : 438
YP 006460172 : YTTIMKLPFLFFIRIGFMLASLGGSCSLLPQLQK--ELLPWN------------------------- : 438
YP 005090424 : YTTIMKLPFIFFIRIGFMLASLGGSHSLLRQLQK--DKLCWNLESSVKFIIALKSKKKCTSMCCSIP : 463
YP 173470     : YTTIIKLPFIFFIRIGFMLASLGGSRSLLRQLQK--DKLRWN------------------------- : 438
               YTTI6KLPF FFIrIGFMLASLGGs SLLrQL K    L W
```

Figure S3.11 The amino acid sequence allignment of CCMFN in mitogenome of *S. miltiorrhiza* and the corresponding homologs in other species

```
                                 *        20         *        40         *        60         *        80         *       100
YP 009173852 : --------------MSIYELFHYSLFPGPFVAFTYNKKQPPAFGAAPAFWCILLSFLGLSFRHIPNNLSNYNVLTANAPFFYQISGTWSNHEGSILSWCR :  86
YP 009153939 : --------------MPIYELFHYSLFPGLFVAFTYNKKQPPAFGAAPAFWCILLSFLGLSFRHIPNNLSNYNVLTANAPFFYQISGTWSNHEGSILSWCR :  86
XP 012482014 : MMGITKQNFRNKHEMPIYELFHYSLFPGLFVAFTYNKKQPPAFGAAPAFWCILLSFLGLSFRHIPNNLSNYNVLTANAPFFYQISGTWSNHEGSILSWCR : 100
XP 011102275 : --------------MSIYEFFHYSLFPGLFVAFTYNKKEPPVFGAAPAFWCILLSFLGLSFRHIPNNLSNYNVLTANAPFFYQISGTWSNHEGSILSWCR :  86
CCMFN SAMI   : --------------MSIYEFFHYSLFPGLFVAFTYNKKEPPVFGAAPAFWCILLSFLGLSFRHIPNNLSNYNVLTANAPFFYQISGTWSNHEGSILSWCR :  86
YP 008964118 : --------------MSIYEFFHYSLFPGLFVAF--NKKEPPVFGAAPAFWCILLSFLGLSFRHIPNNLSNYNVLTANAPFFYQISGTWSNHEGSILSWCR :  84
YP 006460184 : --------------MSIYEFFHYSLFPGLFVAFTYNKKEPPVFGAAPAFWCILLSFLGLSFRHIPNNLSNYNVLTANAPFFYQISGTWSNHEGSILSWCR :  86
YP 006291807 : --------------MSIYELFHYSLFPGLFVAFTYNKKQPPVFGAALAFWCILLSFLGLSFRHIPNNLSNYNVLTANAPFFYQISGTWSNHEGSILSWCR :  86
YP 005090423 : --------------MSIYELFHCSLFPGLFVAFTYNKKQPLVFGAAPAFWCILLSFLGLSFRHIPNNLSNYNVLTANAPFFYQISGTWSNHEGSILSWCR :  86
YP 004237259 : --------------MSIYELFHYSLFPGLFVAFTYNKKQPPAFGAAPAFWCILLSFLGLSFRHIPNNLSNYNVLTANAPFFYQISGTWSNHEGSILSWCR :  86
YP 173394     : --------------MSIYELFHYSLFTGLFVAFTYNKKQPPVFGAALAFWCILLSFLGLSFRHIPNNLSNYNVLTANAPFFYQISGTWSNHEGSILSWCR :  86
                             MsIYE FHySLFpGlFVAFtyNKK2Pp FGAApAFWCILLSFLGLSFRHIPNNLSNYNVLTANAPFFYQISGTWSNHEGSILSWCR

                                 *       120         *       140         *       160         *       180         *       200
YP 009173852 : IPSFYGFLLCY--RGRLQSHNVSKRGDHRETLFYSFVSNFVKNSILSLPRYEQKSGAAPQLYTPFDS-DLVDSELRLQRNRTFDGPALF--YAPLYPERR : 181
YP 009153939 : IPNFYGFLLCY--RGRPQRHNVSKRGGHRETIFSFFVSNFVKNSILSLPRYEQKSGAAQKLYTPFVLRTFVDSELCSRRNRTFDGPALF--YAPLYPERK : 182
XP 012482014 : IPNFYGFLLCY--RGRPQRHNVSKRGGHRETIFSFFVSNFVKNSILSLPRYEQKSGAAQKLYTPFVLRTFVDSELCSRRNRTFDGPALF--YAPLYPERK : 196
XP 011102275 : ILSFYGFLLCY--RGRPKSHNVSKRGGHRETLFYSFLSNFVKNSILSLPRYEQKSGAQPQLYTPFVLRTLVDSELRSRRNRTFDGPALF--YAPLYPERK : 182
CCMFN SAMI   : ILSFYGFLLCYCYRGRPKSHNVSKRGGYRETLFYSFLSNFVKNSILSLPRYEQKSGAQPQLYTPFVLRTLVDSELRSRRNRTFYGPALF--YAPLYPERK : 184
YP 008964118 : ILSFYGFLLCY--RGRPKSHNVSKRGGHRESLFYSFLSNFVNNSILSLPRYEQKSGAQPQLYTPFVLRTLVDSELRSRRNRTFDRPALF--YAPLYPERK : 180
YP 006460184 : ILSFYGFLLCY--RGRPKSHNVSKRGGHRETLFYSFLSNFVKNSILSLPRYEQKSGAQPQLYTPFVRRTLVDSELRSRRNRTFDGPALF--YAPLYPERK : 182
YP 006291807 : ILSFYGFLLCY--RGRPQSHNVSKRGGHRETLFYSFVSNFVKNPILSLPRYEQKSRAAPQLYTPFVLRTFVDSELRSRRNRTFDGPALFYVYAPLYPERK : 184
YP 005090423 : ILSFYGFLLCY--RGRPKSHNVSKRGGHRETLFSSFVSNFVKNSILSLPRYEQKSGAQTQLYTPFVLRTLVDSELRSRKNRTFDGPALF--YAPLFPERK : 182
YP 004237259 : IPSFYGFLLCY--RGRPQSHNVSKRGGHRETIFYSFVSNFVKNSILSLSRYEQKSGAAPQLYTPFVLRILVDSELRSRRNRTFDGPALF--YAPLYPERK : 182
YP 173394     : ILSFYGFLLCY--RGRPLSHNVSKRGGHRETFFYSFVSNFVKNSILSLPRYEQKSGAAPQLYTPFVLRTLVDSELRSRRNRTFDGPALF--YAPLYPERK : 182
               I sFYGFLLCY  RGRp sHNVSKRGghRE3 F sF6SNFVkNsILSLpRYEQKSgA  qLYTPFvlrt VDSELrsr4NRTFdgPALF  YAPL5PER4

                                 *       220         *       240         *       260         *       280         *       300
YP 009173852 : MSFAPLGARRSRGSQEGKRTHPLLHLARDDKERASSIDEKRIDGALGIALFFSLFLSASSDPFVRNFFVRTEPLAESNPVPQDPISAIHPPCIYAGDVAS : 281
YP 009153939 : MSFSPLGARRSRGSREGKRTHPLLHLARDDKERASSIDEQRIDGALGIALFFSPFLSVSSDPFVRNFFVRTEPLAESNPVPQDPISAIHPPCIYAGDVAS : 282
XP 012482014 : MSFSPLGARRSRGSREGKRTHPLLHLARDDKERASSIDEQRIDGALGIALFFSPFLSVSSDPFVRNFFVRTEPLAESNPVPQDPISAIHPPCIYAGDVAS : 296
XP 011102275 : MSFAPMGARRSRGSREGKRTHPLLHLARDDKERASFIDEQRIDGALGIALFFSPFLSASSDPFVRNFFVRTEPLAESNPVPQDPISAIHPPCIYAGDVAS : 282
CCMFN SAMI   : MSFALLGARRSRGSREGKRTHPLLHLARDDKERASFIDEQRIDGALGIALFFSPFLSASSDPFVRNFFVRTEPLAESNPVPQDPISAIHPPCIYAGAVAS : 284
YP 008964118 : IRFALLGARRSRGSRKAKRTHPLLHLARDDKERASFIDEQRIDGALGIALFFSPFLSASSDPFVRNFFVRTEPLAESNPVPQDPISAIHPPCIYAGAVAS : 280
YP 006460184 : MSFAPLGARRSRGSREGKRTPPLLHLARDDKERASFIEEQRIDGALGIALFFSPFLSVSSDPFVRNFFVRTEPLAESNPVPQDPISAIHPPCIYAGDVAS : 282
YP 006291807 : MSFAPLGARRSRGSREGKRMSPLLHLARDDKERASSIDEQRIDGALGIALFFSPFLSASSDPFVRNFFVRTEPLAESNPVPQDPISAIHPPCIYAGDVAS : 284
YP 005090423 : MSFAPLGARRSRGSGEGKRTHPLLHLARDDKERASFIDEQRID--LGIALFFSPFLSASSDPFVRNFFVRTEPLAESNPVPQDPLSAIHPPCIYAGDVAS : 280
YP 004237259 : MSFAPLGARRSRGSREGKRMSPLLHLARDDKERASSIDEKRIDGALGIALFFSLFLSASSDPFVRNFFVRTEPLAESNPVPQDPISAIHPPCIYAGDVAS : 282
YP 173394     : MSFAPLGARRSRGSREGKR---MLHLARDDKERASSIDEQRIDGALGIALFFSPFLSASSDPFVRNFFVCTEPLAESNPVPQDPISAIHPPCIYAGDVAS : 279
               6sFap6GARRSRGSregKR p6LHLARDDKERAS IdEqRIDgaLGIALFFSpFLS SSDPFVRNFFVrTEPLAESNPVPQDP6SAIHPPCIYAGdVAS
```

```
                        *        320         *        340         *        360         *        380         *        400
YP 009173852 : AMGFGLCRSKMMNGIVSLHSPPMRKDAVEKKGTLLRSAGCVGSRITSEFFTLKFKHVGAKCYPALLLRSNRSPLMLLRRRFFAFSSL-WTGALVDTGGEQ : 380
YP 009153939 : AMGFGLCRSKMMNGIVALHSPPMRKDAAEKNGTLLCSAGCVGSRITSELFTLKFKHVGAKCYPALLLRSNRSPLMLLRRRFFAFSSLCWTGALVDTWREQ : 382
XP 012482014 : AMGFGLCRSKMMNGIVALHSPPMRKDAAEKNGTLLCSAGCVGSRITSELFTLKFKHVGAKCYPALLLRSNRSPLMLLRRRFFAFSSLCWTGALVDTWREQ : 396
XP 011102275 : AMGFGLCRSKMMNGIVALHSPLMRKDAAEKNGTLFRSVGCVGSRITSELFTLKFKDVGAKCYPALLLRSNRSLLMLLRRRFFAFSSL-WTRALVDTGREQ : 381
CCMFN SAMI   : AMGCGLCRSKMMNGIVALHSPLMRKDAAEKTGRLFRSVGCVGSRIASELFTLKFKDVGAKCYPALLLRSNRSLLMLLRRRFFAFSSL-WTRALVDTGREQ : 383
YP 008964118 : AMGCGLCRSKMMNGIVALHSPLMRKDAAEKTGRLFRSVGCVGSRIASELFTLKLKDVGAKCYPALLLRSNRSLLMLLRRRFFAFSSL-WTRALVDTGREQ : 379
YP 006460184 : AMGFGLCKSKMMNGIVALHSPLMRKDAAEQNGRLFRSVGCVGSRITSELFTLKFKDVGAKCYPALLLRSNRSLLMLLRRRFFAFSSL-WTRALVDTGREQ : 381
YP 006291807 : AMGFGLCRSKMMNGIVALHSPPMRKDAAEKNGTLFRSAGCVGSRITSELFTLKFKHVGEKCYPALLLRSNRSPLMLLRRRFFALSSL-WTGALVDTGREQ : 383
YP 005090423 : AMGFGLCRSKMMNGIVALHSPLMRKDAAEKNGTLFRSVGCVGSRITSELFTLKFKYVGAKCYPALLLRSNRSLLMLLRWRFFDLSSL-WTRALVDTGREQ : 379
YP 004237259 : AMGFSLCRSKMMNGIVALHSPPMRKDAAEKNGTLLCSAGCVGSRITSELFTLKFKHVGAKCYPALLLRSNRSPLMLLRRRFFAFSSL-WTGALVDTGREQ : 381
YP 173394     : AMGFGLCRSKMMNGIVALHSPPMRKDAVEKNGTLFRSVGCVGSRITSELFTLKFKHVGAKCYPALLLRSNRSLLMLLRRRFFAFSSL-WTGALVDTGREQ : 378
               AMGfgLC4SKMMNGIVaLHSP MRKDAaEk G L  S GCVGSRItSElFTLKfK VGaKCYPALLLRSNRS LMLLRrRFFafSSL WT ALVDTgrEQ

                        *        420         *        440         *        460         *        480         *        500
YP 009173852 : TKRVFRNGKKETTTSPLCWTAGANTVVSDQDQEPIRIWILTCWWLVTVGILPGSWWAHHELGRGGWWFRDPVENASFMPRVLATARIHSVIIPLLHSWTS : 480
YP 009153939 : AKRVVRNGKKDTTTSPLCWTAAANTVVSDQDQEPIRIWILTCWWFLTVGILPGSWWAYHELGRGGWWFRDPVENASFMPRVLATARIHSVILPLLHSWTS : 482
XP 012482014 : AKRVVRNGKKDTTTSPLCWTAAANTVVSDQDQEPIRIWILTCWWFLTVGILPGSWWAYHELGRGGWWFRDPVENASFMPRVLATARIHSVILPLLHSWTS : 496
XP 011102275 : AKRVVRNGKKDTTTAPLCWTAGANTVVSDQDQEPIRIWILTCRWFLTVGILPGSWWAHHELGRGGWWFRDPVENASFMPRVLATACIHSVILPLLHSWTS : 481
CCMFN SAMI   : AKRVVRNGKKDTTTAPLCWTAGANTVVSDQDQEQIRIWILACRWFLTVGILPGSWWAHHELGRGGWWFRDPVENASFMPRVLATACIHSVILPLLHSCTS : 483
YP 008964118 : AKRVVRNGKKETTTAPLCWTAGANTVVSDQDQEPIRIWILACRWFLTVGILPGSWWAHHELGRGGWWFRDPVENASFMPRVLATACIHSVILPLLHSCTS : 479
YP 006460184 : AKRVVRNGKKDTTTAPLCWTAGANTVVSDQDQEPIRIWILTCRWFLTVGILPGSWWAHHELGRGGWWFRDPVENASFMPRVLATACIHSVILPLLHSCTS : 481
YP 006291807 : AKRVVRNGKKDTTTSPLCWTAGANTVVSDQDQEPIRIWILTCRWFLTVGILPGSWWAHHELGRGGWWFRDPVENASFMPRVLATARIHSVILPLLHSWTS : 483
YP 005090423 : AKHVVRNEKKDTTTAPLCWTDGANTVVSDQDQEPIRIWILTCRWFLTVGILPGSWWAHHELGRGGWWFRDPVENASFMPRVLATACIHSVIQPLLHSWTS : 479
YP 004237259 : AKRVVRNGKKDTTTSPLCWTAGANTVVSDQDQEPIRIWILTCWWFLTVGILPGSWWAHHELGRGGWWFRDPVENASFMPRVLATARIHSVILPLLHSWTS : 481
YP 173394     : AKRVVRNGKKDTTTSPLCWTAGANTVVSDQDQEPIRIWILTCRWFLTVGILPGSWWAYHELGRGGWWFRDPVENASFMPRVLATARIHSVILPLLHSWTS : 478
               aKrVvRNgKKdTTT PLCWTagANTVVSDQDQEpIRIWILtC Wf6TVGILPGSWWA HELGRGGWWFRDPVENASFMPRVLATA IHSVI PLLHS TS

                        *        520         *        540         *        560         *        580         *        600
YP 009173852 : FLNIVTLPCCVSGTFSIRSGLLAPVHSFATDDTRGIFLWRFFLLMTGISMILFSQMKQQASVRRTYKKEMVVARSTLVHLRHSARAQPRPVMLWKN---- : 576
YP 009153939 : FLNIVTFPCCVLGTFSIRSGLLAPVHSFATDDTRGIFLWRFFLLMTGISMILFSQMKQQASVRITYKKEMVVARSTLVHLRHSARAQPRPVMLWKN---- : 578
XP 012482014 : FLNIVTFPCCVLGTFSIRSGLLAPVHSFATDDTRGIFLWRFFLLMTGISMILFSQMKQQASVRITYKKEMVVARSTLVHLRHSARAQPRPVMLWKN---- : 592
XP 011102275 : FLNIVTFPCCVSGTFSIRSGLLAPVHSFATDDTQGIFLWQFFLPMTGISMILFCQMKQXTSVRRTYKKEMAVARSTLVHLRHSTRAQARPVILYKNXTYC : 581
CCMFN SAMI   : FLNIVTFPCCVSGTFSIRSGLLAPVHSFATDDTRGIFLWPFFLLMTGISMILFSQMKQQASVHRTYKREMVVARSTLVHLRHSTRAQARPVILWKN---- : 579
YP 008964118 : FLNIVTFPCCVSGTFSIRSGLLAPVHSFATDDTRGIFLWPFFLLMTGISMILFSQMKQQASVHRTYKKEMVVARSTLVHLRHSTRAQARPVILWKNLAYC : 579
YP 006460184 : FLNIVTFPCCVSGTFSIRSGLLAPVHSFATDDTRGIFLWWFFLLMTGISMILCCQMKQQASVRRTYKKEMVVARSTLVHLRHSTRAQARPVILWKN---- : 577
YP 006291807 : FLNIVTLPCCVSGTSSIRSGLLAPVHSFATDDTRGIFLWRFFLLMTGISMILFSQMKQQASVRRTYKKEMVMARSTLVHLRHSARAQPRPVMLWKN---- : 579
YP 005090423 : FLNIVTFPCCVSGTFSIRSGLLAPVHSFATDDTRGIFLWRFFLLMTGISMILFSQMKQQASVRRTYKKEMVVARSTLVHLRHSTPAQARPVILWKN---- : 575
YP 004237259 : FLNIVTLPCCVSGTFSIRSGLLAPVHSFATDDTRGIFLWRFFLLMTGISMILFSQMKQQASVRRTYKKEMVVARSTLVHLRHSARAQPRPVMLWKNLTYC : 581
YP 173394     : FLNIVTFPCCVSGTFSIRSGLLAPVHSFATDDTRGIFLWRFFLLMTGISMILFSQMKQQASVRRTYKKEMVVARSTLVHLRHSARAQARPLMLWKN---- : 574
               FLNIVT PCCVsGTfSIRSGLLAPVHSFATDDTrGIFLW FFLlMTGISMILfsQMKQqaSVrrTYK4EMv6ARSTLVHLRHS rAQ RP66L5KN
```

```
                       *
YP 009173852 : ----------------- :   -
YP 009153939 : ----------------- :   -
XP 012482014 : ----------------- :   -
XP 011102275 : XAGYSKPAIGCRISSRN : 598
CCMFN SAMI   : ----------------- :   -
YP 008964118 : ----------------- :   -
YP 006460184 : ----------------- :   -
YP 006291807 : ----------------- :   -
YP 005090423 : ----------------- :   -
YP 004237259 : WAGYSEPAIGCRISSRN : 598
YP 173394    : ----------------- :   -
```

Figure S3.12 The amino acid sequence allignment of COB in mitogenome of *S. miltiorrhiza* and the corresponding homologs in other species

```
                       *        20         *        40         *        60         *        80         *       100
YP 009153936 : MTIRNQRFSLLKQPISSTLNQHLIDYPTPSNLSYWWGFGSLAGICLVIQIVTGVFLAMHYTPHVDLAFNSVEHVMRDVEGGWLLRYMHANGASMFLIVVY : 100
YP 009049703 : MTIRNQRLSLLKQPISSTLNQHLIDYPTPSNLSYWWGFGSLAGICLVIQIVTGVFLAMHYTPHVDLAFNSVEHIMRDVEGGWLLRYMHANGASMFFIVVH : 100
YP 009041167 : MTIRNQRFSLLKQPISSTLNQHLIDYPTPSNLSYWWGFGSLAGLCLVIQIVTGVFLAMHYTPHVDLAFNSVEHIMRDVEGGWLLRYMHANGASMFLIVVH : 100
COB SAMI     : MTLRNQRFSLLKQPIASTLNQHLIDYPTPSNLSYWWGFGSLAGICLVIQIVTGIFLAMHYTPHVDLAFNSVEHIMRDVEGGWLLRYMHANGASMFFIVVH : 100
YP 008964107 : MTLRNQRLSLLKQPVLSTLNQHLIDYPTPSNLSYWWGFGSLAGICLVIQIVTGIFLAMHYTPHVDLAFNSVEHIMRDVEGGWLLRYMHANGASMFFIVVH : 100
YP 006460158 : MTLRNQRFSLLKQPISSTLNQHLIDYPTPSNLSYWWGFGSLAGICLVIQIVTGVFLAMHYTPHVDLAFNSVEHIMRDVEGGWLLRYMHANGASMFFIVVH : 100
YP 005090407 : MTIRNQRFSLLKQPISSTLNQHLIDYPTPSNLSYWWGFGSLAGICLVIQIVTGVFLAMHYTPHVDLAFNSVEHIMRDVEGGWLLRYMHANGASMFFIVVH : 100
YP 004927538 : MTIRNQRFSLLKQPISSTLNQHLVDYPTPSNLSYWWGFGSLAGICLVIQIVTGVFLAMHYTPHVDLAFNSVEHIMRDVEGGWLLRYMHANGASMFFIVVY : 100
YP 004237271 : MTIRNQRFSLLKQPISSTLNQHLIDYPTPSNLSYWWGFGPLAGICLVIQIVTGVFLAMHHTPHVDLAFNSVEHIMRDVEGGWLLRYMHANGASMFLIVVH : 100
YP 004222814 : MTIRNQRFSLLKEPISSTLNQHLIDYPTPSNLSYWWGFGSLAGICLVIQIVTGVFLAMHYTPHVDLAFNSVEHVMRDVEGGWLLRYMHANGASMFLIVVH : 100
YP 173366     : MTIRNQRLSLLKQPISSTLNQHLIDYPTPSNLSYWWGFGSLAGICLVIQIVTGVFLAMHYTPHVDLAFNSVEHIMRDVEGGWLLRYMHANGASMFFIVVH : 100
               MT6RNQR SLLK2P6sSTLNQHL6DYPTPSNLSYWWGFGsLAG6CLVIQIVTG6FLAMHyTPHVDLAFNSVEH6MRDVEGGWLLRYMHANGASMF IVVh

                       *       120         *       140         *       160         *       180         *       200
YP 009153936 : LHIFRGLYHASYSSPREFVRCLGVVIFLLMIVTAFIGYVLPWGQMSFWGATVITSLASAIPVVGDTIVTWLWGGFSVDNATLNRFFSLHHLLPFILVGAS : 200
YP 009049703 : LHIFRGLYHASYSSPREFVRCLGVVIFLLMIVTAFIGYVLPWGQMSFWGATVITSLASAIPVVGDTIVTWLWGGFSVDNATLNRFFSLHHLLPFILVGAS : 200
YP 009041167 : LHIFRGLYHASYSSPREFVRCLGVVIFLLMIVTAFIGYVLPWGQMSFWGATVITSLASAIPVVGDTIVTWLWGGFSVDNATLNRFFSLHHLLPFILVGAS : 200
COB SAMI     : LHIFRGLYHGSYSSPREFVRCLGVVIFLLMIVTAFIGYVLPWGQMSFWGATVITSLASAIPVVGDTIVTWLWGGFSVDNATLNRFFSLHHLLPFILVGAS : 200
YP 008964107 : LHIFRGLYHASYSSPREFVRCLGVVIFLLMIVTAFIGYVLPWGQMSFWGATVITSLASAIPVVGDTIVSWLWGGFSVDNATLNRFFSLHHLLPFLLVGAS : 200
YP 006460158 : LHIFRGLYHASYSSPREFVRCLGVVIFLLMIVTAFIGYVLPWGQMSFWGATVITSLASAIPVVGDTIVTWLWGGFSVDNATLNRFFSLHHLLPFILVGAS : 200
YP 005090407 : LHIFRGLYYASYSSPREFVRCLGVVIFLLMIVTAFIGYVLPWGQMSFWGATVITSLASAIPVVGDTIVTWLWGGFSVDNATLNRFFSLHHLLPFILVGAS : 200
YP 004927538 : LHIFRGLYHASYSSPREFVWCLGVVIFLLMIVTAFIGYVLPWGQMSFWGATVITSLASAIPVVGDTIVTWLWGGFSVDNATLNRFFSLHHLLPFILVGAS : 200
YP 004237271 : LHIFRGLYHASYSSPREFVRCLGVVIFLLMIVTAFIGYVLPWGQMSFWGATVITSLASAIPVVGDTIVTWLWGGFSVDNATLNRFFSLHHLLPFILVGAS : 200
YP 004222814 : LHIFRGLYHASYSSPREFVRCLGVVIFLLMIVTAFTGYVLPWGQMSFWGATVITSLASAIPVVGDTIVTWLWGGFSVDNATLNRFFSLHHLLPFLLVGAS : 200
YP 173366     : LHIFRGLYHASYSSPREFVRCLGVVIFLLMIVTAFIGYVLPWGQMSFWGATVITSLASAIPVVGDTIVTWLWGGFSVDNATLNRFFSLHHLLPFILVGAS : 200
               LHIFRGLYhaSYSSPREFVrCLGVVIFLLMIVTAFiGYVLPWGQMSFWGATVITSLASAIPVVGDTIV3WLWGGFSVDNATLNRFFSLHHLLPF6LVGAS

                       *       220         *       240         *       260         *       280         *       300
YP 009153936 : LLHLAALHQYGSNNPLGVHSEMDKISFYPYFYVKDLVGWVAFAIFFSIWIFYAPNVLGHPDNYIPANPMPTPPHIVPEWYFLPIHAILRSIPDKSGGVAA : 300
YP 009049703 : LLHLAALHQYGSNNPLGVHSEMDKIASYPYFYVKDLVGWVAFAIFFSIWIFYAPNVLGHPDNYIPANPMSTPPHIVPEWYFLPIHAILRSIPDKSGGVAA : 300
YP 009041167 : ILHLAALHQYGSNNPLGVHSEMDKIASYPYFYVKDLVGWVAFAIFFSIWIFYAPNVLGHPDNYIPANPMSTPPHIVPEWYFLPIHAILRSIPDKSGGVAA : 300
COB SAMI     : ILHLAALHQYGSNNPLGVHSEMDKIAFYPYFYVKDLVGWVAFAIFFSIWIFYAPNVLGHPDNYIPANPMSTPPHIVPEWYFLPIHAILRSIPDKSGGVAA : 300
YP 008964107 : LIHLAALHQYGSNNPLGVHSEMDKIAFYPYFYVKDLVGWVAFAIFFSVWIFYAPNVLGHPDNYIPANPMSTPPHIVPEWYFLPIHAILRSIPDKSGGVAA : 300
YP 006460158 : LLHLAALHQYGSNNPLGVHSEMDKIAFYPYFYVKDLVGWVAFAIFFSIWIFYAPNVLGHPDNYIPANPMSTPPHIVPEWYFLPIHAILRSIPDKSGGVAA : 300
YP 005090407 : LLHLAALHQYGSNNPLGVHSEMDKIAFYPYFYVKDLVGWVAFAIFFSIWIFYAPNVLGHPDNYIPANPMSTPPHIVPEWYFLPIHAILRSIPDKSGGVAA : 300
YP 004927538 : LLHLAALHQYGSNNPLGVHSEMDKIAFYPYFYVKDLVGWVAFAIFFSIWIFYAPNVLGHPDNYIPANPMSTPPHIVPEWYFLPIHAILRSIPDKAGGVAA : 300
YP 004237271 : LLHLAALHQYGSNNPLGVHSEMDKIAFYPYFYVKDLVGWVAFAIFFSIWIFYAPNVLGHPDNYIPANPMPTPPHIVPEWYFLPIYAILRSIPDKSGGVAA : 300
YP 004222814 : LLHLAALHQYGSNNPLGVHSEMDQISFYPYFYVKDLVGWVAFAIFFSIWIFYAPNVLGHPDNYIPANPMPTPPHIVPEWYFLPIHAILRSIPDKSGGVAA : 300
YP 173366     : LLHLAALHQYGSNNPLGVHSEMDKIASYPYFYVKDLVGWVAFAIFFSIWIFYAPNVLGHPDNYIPANPMSTPPHIVPEWYFLPIHAILRSIPDKAGGVAA : 300
               66HLAALHQYGSNNPLGVHSEMDkIa YPYFYVKDLVGWVAFAIFFS6WIFYAPNVLGHPDNYIPANPM TPPHIVPEWYFLPIhAILRSIPDKsGGVAA
```

```
                          *      320         *      340         *      360         *      380         *      400
YP 009153936 : IAPVFICLLALPFFKSMYVRSSSFRPIYQGIFWLLLADCLLLGWIGCQPVEAPFVTIGQISPLVFFLFFAITPILGRVGRGIPNSYTETEHA-------- : 392
YP 009049703 : IAPVFICLLALPFFKSMYVRSSSFRPIHQGIFWLLLADCLLLGWIGCQPVEAPFVTIGQISPLVFFLFFAITPILGRVGRGIPNSYTDETDHT------- : 393
YP 009041167 : IAPVFICLLALPFFKSMYVRSSSFRPIHQGIFWLLLADCLLLGWIGCQPVEAPFVTIGQISPFVFFLFFAITPILGRVGRGIPNSYTDETDNT------- : 393
COB SAMI     : IASVFICLLALPFFKNMYVRSSSFRPIHQGIFWLLLADRLLLGWIGCQPVEAPFVTIGQISPFLFFFFFAITPILGRVGRGIPNSYTDNEIQKR------ : 394
YP 008964107 : IALVFICLLALAFFPNMYVRSSSFRPIHQGIFWLLLADCLLLGWIGCQPVEAPFVTIGQMSSLIFFLFFAITPVLGRVGRGIPHSYTDDKEIQ------- : 393
YP 006460158 : IAPVFICLLALPFFKNMYVRSSSFRPIHQGIFWLLLADRLLLGWIGCQPVEAPFVTIGQISPFLFFLFFAITPILGRVGRGIPNSYTDNEFLKIK----- : 395
YP 005090407 : IAPVFICLLALPFFKNMYIRSSSFRPIHQGIFWLLLADRLLLGWIGCQPVEAPFVTIGQISPFLFFLFFAITPILGRVGIGIPNSYTDNEIQKRKENRKS : 400
YP 004927538 : IAPVFICLLALPFFKSMYVRSSSFRPIHQGMFWLLLADCLLLGWIGCQPVEAPFVTIGQISPLVFFLFFAITPILGRVGRGIPNSYTDETDHT------- : 393
YP 004237271 : IAPVFICLLALPFFKSMYVRSSSFRPIHQGIFWLLLADCLLLGWIGCQPVEAPFVTIGQISPFVFFLFFAITPIPGRVGKGIPNSYTDETDHT------- : 393
YP 004222814 : IAPVFICLLALPFFKSMYVRSSSFRPIHQGIFWLLLADRLLLGWIGCQPVEAPFVTIGQIPPFVFFLFFAITPIPGRVGRGIPNSYTTDETEM------- : 393
YP 173366     : IAPVFICLLALPFFKSMYVRSSSFRPIHQGIFWLLLADCLLLGWIGCQPVEAPFVTIGQISPLVFFLFFAITPILGRVGRGIPNSYTDETDHT------- : 393
               IApVFICLLALpFFk MY6RSSSFRPIhQG6FWLLLAD LLLGWIGCQPVEAPFVTIGQ6sp 6FFlFFAITP6lGRVG GIPnSYTd

YP 009153936 : - :   -
YP 009049703 : - :   -
YP 009041167 : - :   -
COB SAMI     : - :   -
YP 008964107 : - :   -
YP 006460158 : - :   -
YP 005090407 : V : 401
YP 004927538 : - :   -
YP 004237271 : - :   -
YP 004222814 : - :   -
YP 173366    : - :   -
```

Figure S3.13 The amino acid sequence allignment of COX1 in mitogenome of *S. miltiorrhiza* and the corresponding homologs in other species

```
                         *        20         *        40         *        60         *        80         *       100
YP 009153984 : MTNPVRWLFSTNHKDIGTLYFIFGAIAGVMGTCFSVLIRMELARPGDQILGGNHQLYNVLITAHAFLMIFFMVMPAMIGGSGNWFVPILIGAPDMAFPRL : 100
YP 009153951 : MTNPVRWLFSTNHKDIGTLYFIFGAIAGVMGTCFSVLIRMELARPGDQILGGNHQLYNVLITAHAFLMIFFMVMPAMIGGSGNWFVPILIGAPDMAFPRL : 100
YP 009121943 : MTNPVRWLFSTNHKDIGTLYFIFGAIAGVMGTCFSVLIRMELARPGDQILGGNHQLYNVLITAHAFLMIFFMVMPAMIGGSGNWSVPILIGAPDMAFPRL : 100
YP 009049663 : MTNPVRWLFSTNHKDIGTLYFIFGAIAGVMGTCFSVLIRMELARPGDQILGGNHQLYNVLITAHAFLMIFFMVMPAMIGGSGNWSVPILIGAPDMAFPRL : 100
YP 009045759 : MTNPVRWLFSTNHKDIGTLYFIFGAIAGVMGTCFSVLIRMELARPGDQILGGNHQLYNVLITAHAFLMIFFMVMPAMIGGSGNWSVPILIGAPDMAFPRL : 100
YP 009041186 : MTNPVRWLFSTNHKDIGTLYFIFGAIAGVMGTCFSVLIRMELARPGDQILGGNHQLYNVLITAHAFLMIFFMVMPAMIGGSGNWSVPILIGAPDMAFPRL : 100
COX1 SAMI     : MRNLVRWLFSTNHKDIGTLYFIFGAIAGVMGTCFSVLIRMELARPGDQILGGNHQLYNVLITAHAFLMIFFMVMPAMIGGSGNWSVPILIGAPDMAFPRL : 100
YP 006460170 : MTNPVRWLFSTNHKDIGTLYFIFGAIAGVMGTCFSVLIRMELARPGDQILGGNHQLYNVLITAHAFLMIFFMVMPAMIGGSGNWSVPILIGAPDMAFPRL : 100
YP 005090432 : MTNPVRWLFSTNHKDIGTLYFIFGAIAGVMGTCFSVLIRMELARPGDQILGGNHQLYNVLITAHAFLMIFFMVMPAMIGGSGNWSVPILIGAPDMAFPRL : 100
YP 002608199 : MTNPVRWLFSTNHKDIGTLYFIFGAIAGVMGTCFSVLIRMELARPGDQILGGNHQLYNVLITAHAFLMIFFMVMPAMIGGSGNWSVPILIGAPDMAFPRL : 100
YP 173395     : MTNPVRWLFSTNHKDIGTLYFIFGAIAGVMGTCFSVLIRMELARPGDQILGGNHQLYNVLITAHAFLMIFFMVMPAMIGGSGNWSVPILIGAPDMAFPRL : 100
               MtNpVRWLFSTNHKDIGTLYFIFGAIAGVMGTCFSVLIRMELARPGDQILGGNHQLYNVLITAHAFLMIFFMVMPAMIGGSGNWsVPILIGAPDMAFPRL

                         *       120         *       140         *       160         *       180         *       200
YP 009153984 : NNISFWLLPPSLLLLLSSALVEVGSGTGWTVYPPLSGIPSHSGGAVDSAISSLHLSGVSSILGSINFITTISNMRGPGMTMHRSPLFVWSVLVTAFPLLL : 200
YP 009153951 : NNISFWLLPPSLLLLLSSALVEVGSGTGWTVYPPLSGITSHSGGAVDSAISSLHLSGVSSILGSINFITTISNMRGPGMTMHRSPLFVWSVLVTAFPLLL : 200
YP 009121943 : NNISFWLLPPSLLLLLSSALVEVGSGTGWTVYPPLSGITSHSGGAVDLAISSLHLSGVSSILGSINFITTIFNMRGPGMTMHRSPLFVWSVLVTAFPLLL : 200
YP 009049663 : NNISFWLLPPSLLLLLSSALVEVGSGTGWTVYPPLSGITSHSGGAVDLAISSLHLSGVSSILGSINFITTIFNMRGPGMTMHRSPLFVWSVLVTAFPLLL : 200
YP 009045759 : NNISFWLLPPSLLLLLSSALVEVGSGTGWTVYPPLSGITSHSGGAVDSAISSLHLSGVSSILGSINFITTISNMRGPGMTMHRSPLFVWSVLVTAFPLLL : 200
YP 009041186 : NNISFWLLPPSLLLLLSSALVEVGSGTGWTVYPPLSGITSHSGGAVDSAISSLHLSGISSILGSINFITTISNMRGPGMTMHRSPLFVWSVLVTAFPLLL : 200
COX1 SAMI     : NNISFWLLPPSLLLLLSSALVEVGSGTGWTVYPPLSGITSHSGGAVDLAISSLHLSGVSSILGSINFITTISNMRGPGMTMHRSPLFVWSVLVTAFPLLL : 200
YP 006460170 : NNISFWLLPPSLLLLLSSALVEVGSGTGWTVYPPLSGITSHSGGAVDLAISSLHLSGVSSILGSINFITTISNMRGPGMTMHRSPLFVWSVLVTAFPLLL : 200
YP 005090432 : NNISFWLLPPSLLLLLSSALVEVGSGTGWTVYPPLSGITSHSGGAVDLAISSLHLSGVSSILGSINFITTISNMRGPGMTMHRSPLFVWSVLVTAFPLLL : 200
YP 002608199 : NNISFWLLPPSLLLLLSSALVEVGSGTGWTVYPPLSGITSHSGGAVDLAISSLHLSGVSSILGSINFITTISNMRGPGMTMHRSPLFVWSVLVTAFPLLL : 200
YP 173395     : NNISFWLLPPSLLLLLSSALVEVGSGTGWTVYPPLSGITSHSGGAVDLAISSLHLSGVSSILGSINFITTISNMRGPGMTMHRSPLFVWSVLVTAFPLLL : 200
               NNISFWLLPPSLLLLLSSALVEVGSGTGWTVYPPLSGItSHSGGAVD AISSLHLSG6SSILGSINFITTIsNMRGPGMTMHRSPLFVWSVLVTAFPLLL

                         *       220         *       240         *       260         *       280         *       300
YP 009153984 : SLPVLAGAITMLLTDRNFNTTFSDPAGGGDPILYQHLFRFFGHPEVYIPILPGSGIISHIVSTFSGKPVFGYLGMVYAMISIGVLGFLVWAHHMFTVGLD : 300
YP 009153951 : SLPVLAGAITMLLTDRNFNTTFSDPAGGGDPILYQHLFRFFGHPEVYIPILPGSGIISHIVSTFSGKPVFGYLGMVYAMISIGVLGFLVWAHHMFTVGLD : 300
YP 009121943 : SLPVLAGAITMLLTDRNFNTTFSDPAGGGDPILYQHLFWFFGHPEVYILILPGFGIISHIVSTFSGKPVFGYLGMVYAMISIGVLGFLVWAHHMFTVGLD : 300
YP 009049663 : SLPVLAGAITMLLTDRNFNTTFSDPAGGGDPILYQHLFWFFGHPEVYIPILPGSGIISHIVSTFSGKPVFGYLGMVYAMISIGVLGFLVWAHHMFTVGLD : 300
YP 009045759 : SLPVLAGAITMLLTDRNFNTTFSDPAGGGDPILYQHLFWFFGHPEVYIPILPGSGIISHIVSTFSGKPVFGYLGMVYAMISIGVLGFLVWAHHMFTVGLD : 300
YP 009041186 : SLPVLAGAITMLLTDRNFNTTFSDPAGGGDPILYQHLFRFFGHPEVYILILPGSGIISHIVSTFSGKPVFGYLGMVYAMISIGVLGFLVWAHHMFTVGLD : 300
COX1 SAMI     : SLPVLAGAITMLLTDRNFNTTFFDPAGGGDPILYQHLFRFFGHPEVYILILPGSGIISHIVSTFSGKPVFGYLGMVYAMISIGVLGFLVWAHHMFTVGLD : 300
YP 006460170 : SLPVLAGAITMLLTDRNFNTTFFDPAGGGDPILYQHLFRFFGHPEVYILILPGSGIISHIVSTFSGKPVFGYLGMVYAMISIGVLGFLVWAHHMFTVGLD : 300
YP 005090432 : SLPVLAGAITMLLTDRNFNTTFFDPAGGGDPILYQHLFWFFGHPEVYILILPGSGIISHIVSTFSGKPVFGYLGMVYAMISIGVLGFLVWAHHMFTVGLD : 300
YP 002608199 : SLPVLAGAITMLLTDRNFNTTFSDPAGGGDPILYQHLFRFFGHPEVYIPILPGSGIISHIVSTFSGKPVFGYLGMVYAMISIGVLGFLVWAHHMFTVGLD : 300
YP 173395     : SLPVLAGAITMLLTDRNFNTTFSDPAGGGDPILYQHLFRFFGHPEVYIPILPGSGIISHIVSTFSGKPVFGYLGMVYAMISIGVLGFLVWAHHMFTVGLD : 300
               SLPVLAGAITMLLTDRNFNTTF DPAGGGDPILYQHLF FFGHPEVYI ILPGsGIISHIVSTFSGKPVFGYLGMVYAMISIGVLGFLVWAHHMFTVGLD
```

```
                 *       320         *       340         *       360         *       380         *       400
YP 009153984 : VDTRAYFTAATMIIAVPTGIKIFSWIATMWGGSIQYKTPMLFAVGFIFLFTIGGLTGIVPANSGLDIALHDTYYVVAHFHYVLSMGAVFALFAGFHYWVG : 400
YP 009153951 : VDTRAYFTAATMIIAVPTGIKIFSWIATMWGGSIQYKTPMLFAVGFIFLFTIGGLTGIVPANSGLDIALHDTYYVVAHFHYVLSMGAVFALFAGFHYWVG : 400
YP 009121943 : VDTRAYFTAATMIIAVPTGIKIFSWIATMWGGSIQYKTPMLFAVGFIFLFTIGGLTGIVLANSGLDIALHDTYYVVAHFHYVLSMGAVFALFAGFHYWVG : 400
YP 009049663 : VDTRAYFTAATMIIAVPTGIKIFSWIATMWGGSIQYKTPMLFAVGFIFLFTIGGLTGIVLANSGLDIALHDTYYVVAHFHYVLSMGAVFALFAGFHYWVG : 400
YP 009045759 : VDTRAYFTAATMIIAVPTGIKIFSWIATMWGGSIQYKTPMLFAVGFIFLFTIGGLTGIVPANSGLDIALHDTYYVVAHFHYVLSMGAVFALFAGFHYWVG : 400
YP 009041186 : VDTRAYFTAATMIIAVPTGIKIFSWIATMWGGSIQYKTPMLFAVGSIFLFTIGGLTGIVLANSGLDIALHDTYYVVAHFHYVLSMGAVFALFAGFHYWVG : 400
COX1 SAMI     : VDTRAYFTAATMIIAVPTGIKIFSWIATMWGGSIQYKTPMLFAVGFIFLFTIGGLTGIVLANSGLDIALHDTYYVVAHFHYVLSMGAVFALFAGFHYWVG : 400
YP 006460170 : VDTRAYFTAATMIIAVPTGIKIFSWIATMWGGSIQYKTPMLFAVGFIFLFTIGGLTGIVLANSGLDIALHDTYYVVAHFHYVLSMGAVFALFAGFHYWVG : 400
YP 005090432 : VDTRAYFTAATMIIAVPTGIKIFSWIATMWGGSIQYKTPMLFAVGFIFLFTIGGLTGIVLANSGLDIALHDTYYVVAHFHYVLSMGAVFALFAGFYYWVG : 400
YP 002608199 : VDTRAYFTAATMIIAVPTGIKIFSWIATMWGGSIQYKTPMLFAVGFIFLFTIGGLTGIVPANSGLDIALHDTYYVVAHFHYVLSMGAVFALFAGFHYWVG : 400
YP 173395     : VDTRAYFTAATMIIAVPTGIKIFSWIATMWGGSIQYKTPMLFAVGFIFLFTIGGLTGIVPANSGLDIALHDTYYVVAHFHYVLSMGAVFALFAGFHYWVG : 400
               VDTRAYFTAATMIIAVPTGIKIFSWIATMWGGSIQYKTPMLFAVGfIFLFTIGGLTGIV ANSGLDIALHDTYYVVAHFHYVLSMGAVFALFAGFhYWVG

                 *       420         *       440         *       460         *       480         *       500
YP 009153984 : KIFGRTYPETLGQIHFWITFFGVNLTFFPMHFLGLSGMPRRIPDYPDAYAGWNALSSFGSYISVVGICRFFVVVTITSSSGNNKRCAPSPWAVEQNSTTP : 500
YP 009153951 : KIFGRTYPETLGQIHFWITFFGVNLTFFPMHFLGLSGMPRRIPDYPDAYAGWNALSSFGSYISVVGICRFFVVVTITSSSGNNKRCAPSPWAVEQNSTTP : 500
YP 009121943 : KIFGRTYPETLGQIHFWITFFGVNMTFFPMHFLGLSGMPRRIPDYPDAYAGWNALSSFGSYISVVGICRFFVVVTITSSSGKNKRCAPSPWAVEQNPTTP : 500
YP 009049663 : KIFGRTYPETLGQIHFWITFFGVNMTFFPMHFLGLSGMPRRIPDYPDAYAGWNALSSFGSYISVVGICRFFVVVTITSSSGKNKRCAPSPWAVEQNPTTP : 500
YP 009045759 : KIFGRTYPETLGQIHFWITFFGVNPTLFPMHFLGLSGMPRRIPDYPDAYAGWNALSSFGSYISVVGIRRFFVVVTITSSSGNNKRCAPSPWAVEQNPTTP : 500
YP 009041186 : KIFGRTYPETLGQIHFWITFFGVNLTFFPMHFLGLSGMPRRIPDYPDAYAGWNALSSFGSYISVVGICRFFVVVTITSSSGKNKRCAPSPWALEQNPTTP : 500
COX1 SAMI     : KIFGRTYPETLGQIHFWITFFGVNLTFFPMHFLGLSGMPRRIPDYPDAYAGWNALSSFGSYISVVGICRFFVVVTITSSSGKNKRCAPSPWAVEQNPTTP : 500
YP 006460170 : KIFGRTYPETLGQIHFWITFFGVNLTFFPMHFLGLSGMPRRIPDYPDAYAGWNALSSFGSYISVVGICRFFVVVTITSTSGKNKRCAPSPWAVEQNPTTP : 500
YP 005090432 : KIFGRTYPETLGQIHFWITFFGVNLTFFPMHFLGLSGMPRRIPDYPDAYAGWNALSSFGSYISVVGICRFFVVVTITSSSGKNKRCAPSPWAVEQNPTTP : 500
YP 002608199 : KIFGRTYPETLGQIHFWITFFGVNPTLFPMHFLGLSGMPRRIPDYPDAYAGWNALSSFGSYISVVGIRRFFVVVTITSSSGNNKRCAPSPWAVEQNSTTP : 500
YP 173395     : KIFGRTYPETLGQIHFWITFFGVNPTLFPMHFLGLSGMPRRIPDYPDAYAGWNALSSFGSYISVVGIRRFFVVVTITSSSGNNKRCAPSPWAVEQNSTTP : 500
               KIFGRTYPETLGQIHFWITFFGVN T FPMHFLGLSGMPRRIPDYPDAYAGWNALSSFGSYISVVGI RFFVVVTITS3SG NKRCAPSPWA6EQN TTP

                 *       520         *
YP 009153984 : EWMVQSPPAFHTFGELPAIKETKTFENKQN : 530
YP 009153951 : EWMVQSPPAFHTFGELPAIKETKTFENKQN : 530
YP 009121943 : EWMVQSPPAFHTFGELPAIKETKSYVK--- : 527
YP 009049663 : EWMVQSPPAFHTFGELPAIKETKSYVK--- : 527
YP 009045759 : EWMVQSPPAFHTFGELPAIKETKSYVK--- : 527
YP 009041186 : EWMVQSPPAFHTFGELPAIKETKSYVK--- : 527
COX1 SAMI     : EWMVQSPPAFHTFGELPAIKETKSYVK--- : 527
YP 006460170 : EWMVQSPPAFHTFGELPAIKETKSYVK--- : 527
YP 005090432 : EWMVQSPPAFHTFGELPAIKETKSDVK--- : 527
YP 002608199 : EWMVQSPPAFHTFGELPAIKETKSYVK--- : 527
YP 173395     : EWMVQSPPAFHTFGELPAIKETKSYVK--- : 527
               EWMVQSPPAFHTFGELPAIKETK3 vk
```

Figure S3.14 The amino acid sequence allignment of COX2 in mitogenome of *S. miltiorrhiza* and the corresponding homologs in other species

```
                        *        20         *        40         *        60         *        80         *       100
YP 009243659 : -------------------MIVLEWLFLTISPCDAAEPWQLGFQDAATPMMQGIIDLHHDIFFFLILILVFVSRILVRALWHFHYQKNPIPQRIVHGTTI :  81
YP 009241671 : -------------------MIVLEWLFLTIAPCDAAEPWQLGSQDAATPMMQGIMDLHHDIFFFLILILVFVSRILVRALWHFHYKKNPIPQRIVHGTTI :  81
YP 009121950 : -------------------MIVLEWLFLTIAPCDAAEPWQLGSQDAATPIMQGITDLHHDVFFFVILILVFVSWILGRALWHFHYKKNPIPQRIVHGTTI :  81
XP 009595053 : MKSLFFLGGAEQSKNEPNQMIVLEWLFLTIAPCDAAEPWQLGSQDAATPIMQGITDLHHDVFFFVILILVFVSWILGRALWHFHYKKNPIPQRIVHGTTI : 100
YP 009049822 : -------------------MIVLEWLFLTIAPCDAAEPWQLGSQDAATPIMQGITDLHHDVFFFVILILVFVSWILGRALWHFHYKKNPIPQRIVHGTTI :  81
YP 009041178 : -------------------MIVLEWLFLTIAPCDAAEPWQLGFQDAATPMMQGIIDLHHDIFFFLILILVFVLWILVRTLWHFHYKKNPIPQRIVHGTTI :  81
COX2 SAMI    : -------------------MIVLECLFLTIAPCDAAEPWQLGSQDAATPMMQGIIDLHHDIFFFLILIFVFVSWILARALWHFHYQKNPIPQRIVHGTTI :  81
YP 008964106 : -------------------MIVLERLFLPIAPCDAAEPWQLGSQDGATPMMQGIIDLHHDIFFFVILISVFVSWILVRALWHFHYQKNTIPQRIVHGTTI :  81
YP 006460161 : -------------------MIVLECLFLTIAPCDAAEPWQLGSQDAATPMMQGIIDLHHDIFFFLILIFVFVSWILVRALWHFHYQKNPIPQRIVHGTTI :  81
YP 005090429 : -------------------MIVLEWLFFTIAPCDAAEPWQLGSQDAATPMMQGIIDLHHDIFFFLILIFVFVSWILVRALWHFHYKKNPIPQRIVHGTTI :  81
YP 173412     : -------------------MIVLEWLFLTIAPCDAAEPWQLGSQDAATPIMQGITDLHHDVFFFVILILVFVSWILGRALWHFHYKKNPIPQRIVHGTTI :  81
                                  MIVLE LFltIaPCDAAEPWQLGsQDaATP6MQGI DLHHD6FFF6ILI VFVswIL RaLWHFHY KNpIPQRIVHGTTI

                        *       120         *       140         *       160         *       180         *       200
YP 009243659 : EILWTIFPSIILMFIAIPSFALLYSMDEVVVDPAITIKAIGHQWYWTYEYSDYNSSDEESLTFDSYMIPEDDLELGQLRLLEVDNRVVVPAKTHLRIIVT : 181
YP 009241671 : EILRTIFPSIIPMFIAIPSFALLYSMDEVVVDPAITIKAIGHQWYRTYEYSDYNSSDEQSLTFDSYTIPEDDLELGQSRLLEVDNRVVVPAKTHLRIIVT : 181
YP 009121950 : EILRTIFPSIIPMFIAIPSFALLYSMDEVVVDPAITIKAIGHQWYRTYEYSDYNSSDEQSLTFDSYTIPEDDLELGQSRLLEVDNRVVLPAKSYIRFIVT : 181
XP 009595053 : EILRTIFPSIILMFIAIPSFALLYSMDEVVVDPAITIKAIGHQWYRTYEYSDYNSSDEQSLTFDSYTIPEDDPELGQSRLLEVDNRVVVPAKSYIRFIVT : 200
YP 009049822 : EILRTIFPSIIPMFIAIPSFALLYSMDEVVVDPAITIKAIGHQWYRTYEYSDYNSSDEQSLTFDSYTIPEDDLELGQSRLLEVDNRVVLPAKSHIRFIVT : 181
YP 009041178 : EILWTIFPSIILMFIAIPSFALLYSMDEVVVDPAITIKAIGHQWYRTYEYSDYNSSDEQSLTFDSYTIPEDDPELGQSRLLEVDNRVVVPAKTHLRIIVT : 181
COX2 SAMI    : EILWTIFPSIILMFIAIPSFALLYSMDEVVVDPAITIKAIGHQWYWTYEYSDYNSSDEQSLTFDSYTIPEDDPELGQSRLLEVDNRVVVPEKSHLRIIVT : 181
YP 008964106 : EILWTLFPSIILMFMAIPSFALLYSMDEGVADPAITIKAIGHQWYWTYEYSDYNSSDEQSLTFDSYTIPEDDPELGQSRLLEVDNRVVVPERSHLRIIVT : 181
YP 006460161 : EILWTIFPSIILMFIAIPSFALLYSMDEVVVDPAITIKAIGHQWYWTYEYSDYNSSDEQSLTFDSYTIPEDDPELGQSRLLEVDNRVVVPEKTHLRIIVT : 181
YP 005090429 : EILWTLFPSIILMFIAIPSFALLYSMDEVVVDPAITIKAIGHQWYWTYEYSDYNSSDEQSLTFDSYTIPEDDPELGQSRLLEVDNRVVVPEKTHLRIIVT : 181
YP 173412     : EILRTIFPSIIPMFIAIPSFALLYSMDEVVVDPAITIKAIGHQWYRTYEYSDYNSSDEQSLTFDSYTIPEDDPELGQSRLLEVDNRVVVPAKSYIRFIVT : 181
               EIL T6FPSII MF6AIPSFALLYSMDEvVvDPAITIKAIGHQWY TYEYSDYNSSDE2SLTFDSYtIPEDD ELGQsRLLEVDNRVV6P 43 6R IVT

                        *       220         *       240         *       260         *       280
YP 009243659 : SADVLHSWAVPSLGVKCDAVPGRLNQISILVQREGVYYGQCSEICGTNHAFS--IVVEAVPRKDYGSRVSNQLIPQTGEA : 259
YP 009241671 : PADVPHSWAVPSSGVKCDAVPGRLNQISISVQREGVYYGQCSEICGTNHAFTP-IVVEAVPRKDYGSRVSNQLIPQTGEA : 260
YP 009121950 : SADVPHSWAVPSLGVKCDAVPGRLNQTSISVQREGVYYGQCSEICGTNHAFMP-IVVEAVPRKDYGSRVSNQLIPQSAEA : 260
XP 009595053 : SADVPHSWAVPSLGVKCDAVPGRLNQTSISVQREGVYYGQCSEICGTNHAFMP-IVVEAVPRKDYGSRVSNQLIPQTGEA : 279
YP 009049822 : SADVPHSWAVPSLGVKCDAVPGRLNQTSISVQREGVYYGQCSEICGTNHAFMP-IVVEAVPRKDYGSRVSNQLIP----- : 255
YP 009041178 : PADVPHSWAVPSLGVKCDAVPGRLNQTSISVQREGVYYGQCSEICGTNHAFMP-IVVEAVSRKDYGDRVSNQLSN----- : 255
COX2 SAMI    : PADVPHSWAVPSLGVKCDAVPGRLNQTSISVQREGVYYGQCSEICGTNHAFMLSIVVEAVPRKDYGSRVSNQLIP----- : 256
YP 008964106 : PADVPHSWAVPSLGVKCDAVPGRLNQTSISVQREGVYYGQCSEICGTNHAFMP-IVVEAVSRKDYGSWVSNQLIP----- : 255
YP 006460161 : PADVPHSWAVPSLGVKCDAVPGRLNQTSISVQREGVYYGQCSEICGTNHAFMP-IVVEAVPRKDYGSWVSNQLIPQTGGA : 260
YP 005090429 : PADVPHSWAVPSLGVKCDAVPGRLNQTSISVQREGVYYGQCSEICGTNHAFMLSIVVEAVPGKDYGSRVSNQFIPQTGRA : 261
YP 173412     : SADVPHSWAVPSLGVKCDAVPGRLNQTSISVQREGVYYGQCSEICGTNHAFMP-IVVEAVPRKDYGSRVSNQLIPQTGEA : 260
                ADVpHSWAVPSlGVKCDAVPGRLNQtSIsVQREGVYYGQCSEICGTNHAFm  IVVEAVprKDYGsrVSNQlip
```

Figure S3.15 The amino acid sequence allignment of COX3 in mitogenome of *S. miltiorrhiza* and the corresponding homologs in other species

```
                           *        20         *        40         *        60         *        80         *       100
XP 016673547 : MGVKGGLHTTEAKWFMIESQRHSYHLVDPSPWPISGSLGALATTVGGVMYMHSFQGGARLLSLGLIFLLYTMFVWWRDVLRESTLEGHHTKVVQLGLRYG : 100
YP 009177615 : ---------------MIESQRHSYHLVDPSPWPISGSLGALATTVGGVMYMHSFQGGARLLSLGLIFLLYTMFVWWRDVLRESTLEGHHTKVVQLGLRYG :  85
YP 009153952 : ---------------MIESQRHSYHLVDPSPWPISGSLGALATTVGGVMYMHSFQGGARLLSLGLIFILYTMFVWWRDVLRESTLEGHHTKVVQLGLRYG :  85
YP 009041182 : ---------------MIDSQRHSYHLVDPSPWPISGSLGTLATTVGGVMYMHSFQGGATLLSLGLIFILYTMFVWWRDVLRESTLEGHHTKVVQLGLRYG :  85
YP 008999556 : ---------------MIESQRHSYHLVDPSPWPISGSLGALATTVGGVMYMHSFQGGATLLSLGLIFILYTMFVWWRDVLRESTLEGHHTKVVQLGPRYG :  85
YP 008758154 : ---------------MIESQRHSYHLVDPSPWPISGSLGALATTVGGVMYMHSFQGGATLLSLGLIFILYTMFVWWRDVLRESTLEGHHTKAVQLGPRYG :  85
YP 006460154 : ---------------MIESQRHSYHLVDPSPWPISGSLGALATTVGGVMYMHSFQGGATLLSLGLIFILYTMFVWWRDVLRESTLEGHHTKVVQLGPRYG :  85
YP 006291820 : ---------------MIESQRHSYHLVDPSPWPISGSLGALATTVGGVMYMHPFQGGATLLSLGLIFLLYTMFVWWRDVLRESTLEGHHTKVVQLGPRYG :  85
YP 005090416 : ---------------MIESQRHSYHLVDPSPWPISGSLGALATTVGGVMYMHSFQGGATLLSLGLIFILYTMFVWWRDVLRESTLEGHHTKVVQLGPRYG :  85
YP 173456     : ---------------MIESQRHSYHLVDPSPWPISGSLGALATTVGGVMYMHSFQGGATLLSLGLIFILYTMFVWWRDVLRESTFEGHHTKVVQLGPRYG :  85
COX3 SAMI     : ---------------MIESQRHSYHLVDPSPWPISGSLGALATTVGGVMYMHSFQGGATLLSLGLIFILYTMFVWWRDVLRESTLEGHHTKVVQLGPRYG :  85
                              MIeSQRHSYHLVDPSPWPISGSLGaLATTVGGVMYMHsFQGGA LLSLGLIF6LYTMFVWWRDVLRESTlEGHHTKvVQLG RYG

                           *       120         *       140         *       160         *       180         *       200
XP 016673547 : FILFIVSEVMFFFAFFWASSHSSLAPTVEIGGIWPPKGIGVLDPWEIPFLNTPILLSSGAAVTWAHHAILAGKEKRAVYALVATVFLALVFTGFQGMEYY : 200
YP 009177615 : FILFIVSEVMFFFAFFWASSHSSLAPTVEIGGIWPPKGIGVLDPWEIPFLNTPILLSSGAAVTWAHHAILAGKEKRAVYALVATVFLALVFTGFQGMEYY : 185
YP 009153952 : FILFIVSEVMFFFAFFWASSHSSLAPTVEIGGIWPPKGIGVLDPWEIPFLNTPILLSSGAAVTWAHHAILAGKEKRAVYALVATVFLALVFTGFQGMEYY : 185
YP 009041182 : FILFIVSEVMFFFALFWASSHSSLAPTVEIGGIWPPKGIEVLDPWEIPFLNTLIPPSSGAAVTWAHHAILAGKEKRAVYALVATVSLALVFTGFQGMEYY : 185
YP 008999556 : FILFIVSEVMFLFALFRASSHSSLAPTVEIGGIWPPKGIAVLDPREIPFLNTLIPLSSGAAVTWAHHAILAGKEKRAVYALVATVSLALVFTAFQGMEYY : 185
YP 008758154 : SILFIVSEVMFLFAFFWASSHSSLAPTVEIGGIWPPKGIGVLDPWEIPLLNTPILPSSGAAVTWAHHAILAGKEKRAVYALVATVSLALVSTGFQGMEYY : 185
YP 006460154 : FILFIVSEVMFFFAFFRASSHSSLAPTVEIGGIWPPKGIGVLDPWEIPFLNTLIPPSSGAAVTWAHHAILAGKEKRAVYALVATVLLALVFTGFQGMEYY : 185
YP 006291820 : FILFIVSEVMFLFALFWASSHSSLAPTVEIGGIWPPKGIGVLDPREIPFLNTLILPSSGAAVTWAHHAILAGKEKRAVYALVATVSLALVFTGFQGMEYY : 185
YP 005090416 : FILFIVSEVMFFFAFFRASSHSSLAPTVEIGGIWPPKGIGVLDPWEIPFLNTLILPSSGAAVTWAHHAILAEKEKRAVYALVATVLLALVFTGFQGMEYY : 185
YP 173456     : FILFIVSEVMFFFALFRASSHSSLAPTVEIGGIWPPKGIAVLDPWEIPFLNTLIPLSSGAAVTWAHHAILAGKEKRAVYALVATVSLALVFTGFQGMEYY : 185
COX3 SAMI     : FILFIVSEVMFFFAFFRASSHSSLAPTVEIGGIWPPKGIGVLDPWEIPFLNTLIPPSSGAAVTWAHHAILAGKEKRAVYALVATVLLALVFTGFQGMEYY : 185
               fILFIVSEVMF FA F ASSHSSLAPTVEIGGIWPPKGI VLDPwEIPfLNT I  SSGAAVTWAHHAILAgKEKRAVYALVATV LALVfTgFQGMEYY

                           *       220         *       240         *       260         *       280
XP 016673547 : QAPFTISDSIYGSTFFLATGFHGFHVIIGTLFLIICGIRQYLGHLTKEHHVGFEAAAWYWHFVDVVRLFPFVSIYWWGGI : 280
YP 009177615 : QAPFTISDSIYGSTFFLATGFHGFHVIIGTLFLIICGIRQYLGHLTKEHHVGFEAAAWYWHFVDVVRLFPFVSIYWWGGI : 265
YP 009153952 : QAPFTISDSIYGSTFFLATGFHGFHVIIGTLFLIICGIRQYLGHLTKEHHVGFEAAAWYWHFVDVVRLFPFVSIYWWGGI : 265
YP 009041182 : QAPSTISDSIYGSSFFLATGFHGFHVIIGTLFLIICGIRQYLGHLTKEHHVGFEAAAWYWHFVDVVRLFPFVSIYWWGGI : 265
YP 008999556 : QAPSTISDSIYGSTFFLATGFHGFHVIIGTLFSIVCGIRQYLGHLTKEHHVGFEAAAWYWHFVDVVRLFPFVSIYWWGGI : 265
YP 008758154 : QAPSTISDSIYGSTFFLATGFHGFHVIIGTLFLIVCGIRQYLGHLTKKHHVGFEAAAWYWHFVDVVRLFPFVSIYWWGGI : 265
YP 006460154 : QAPSTISDSIYGSTFFLATGFHGFHVIIGTLFLIICGIRQYLGHLTKEHHVGFEAAAWYWHFVDVVRLFPFVSIYWWGGI : 265
YP 006291820 : QAPSTISDSIYGSTFFLATGFHGFHVIIGTLFLIVCGIRQYLGHLTKEHHVGFEAAAWYWHFVDVVRLFPFVSIYWWGGI : 265
YP 005090416 : QAPSTISDSIYGSTFFLATGFHGFHVIIGTLFLIICGIRQYLGHLTKEHHVGFEAAAWYWHFVDVVRLFPFVSIYWWGGI : 265
YP 173456     : QAPFTISDSIYGSTFFLATGFHGFHVIIGTIFSIICGIRQYLGHLTKEHHVGFEAAAWYWHFVDVVRLFLFVSIYWWGGI : 265
COX3 SAMI     : QAPSTISDSIYGSTFFLATGFHGFHVIIGTLFLIICGIRQYLGHLTKEHHVGFEAAAWYWHFVDVVRLFPFVSIYWWGGI : 265
               QAP TISDSIYGS3FFLATGFHGFHVIIGT6FlI6CGIRQYLGHLTKeHHVGFEAAAWYWHFVDVVRLFpFVSIYWWGGI
```

Figure S3.16 The amino acid sequence allignment of MATR in mitogenome of *S. miltiorrhiza* and the corresponding homologs in other species

```
                          *        20         *        40         *        60         *        80         *       100
YP 009241663 : MKEAIRMVLESIYDPEFPDTSHFRSGRGRHSALRRIKEEWGTSRWFLEFDIRKCFHTIDRHRLIPIFKEEIDDPKFFYSIQKVFSAGRLVGGEKGPYSVP : 100
YP 009121952 : MKEAIRMVLESIYDLEFPDTSHFRSGRGFHSVLRRIKEEWGTSRWFLEFDIRKCFHTIDRHRLIPIFKEEIDDPKFFYPIQKVFSAGRLVGGEKGPYSVP : 100
YP 009045743 : MKEAIRMVLESIYDPEFPDTSHFRSGQGCHSVLRRIKEEWGISRWFLEFDIRKCFHPIDRHRLIPIFKEEIDDPKFFYSIQKVFSAGRLVGGERGPYSVP : 100
YP 009041166 : MKEAIRIVLESIYDPEFPDTSHFRSGRGCHSVLRRIKEEWGTSRWFLEFDIRKCFHTIDRHRLISIFKEEIDDPKFFYSIHKVFSAGRLVGGEKGPYSVP : 100
YP 008999573 : MKEAIRMVLESIYDPEFPDTSHFRSGRGCHSALRRIKEEWGTSRWFLEFDIRKCFHTIDRHRLIPIFKEEIGDPKFFYSIQKVFSAGRLVGAEKGPYSVP : 100
MATR SAMI    : MKEAIRMVLESIYDPEFPDTSHFRSGRGCHSVLRRIKEEWGTSRWFLEFDIRKSFHTIDRHRLISIFKEEIDDPKFFYSIQKVFSAGRLVGGEKGPYSVP : 100
YP 008964116 : MKEAIRMVLESIYDPEFPDTSHLRLGRGCHSVLRRIKEEWGTSRWFLEFDIRKSFHTIDRHRLISIFKEEIDDPKFFYSIQKVFSAGRLVGGEKGPYSVP : 100
YP 006460156 : MKEAIRMVLESIYDPEFPDTSHFRSGRGCHSVLRRIKEEWGTSRWFLEFDIRKSFHTIDRHRLISIFKEEIDDPKFFYSIQKVFSAGRLVGGEKGPYSVP : 100
YP 006291793 : MKEAIRMVLESIYDPEFPDTSHFRSGRGCHSALRRIKGEWGTSRWFLEFDIRKCFHTIDRHRLIPIFKEEIDDPKFFYPIQKVFSAGRPVGGEKGPYSVP : 100
YP 005090427 : MKEAIRMVLESIYDPEFPDTSHFRSGQGCHSVLRRIKEEWGTSRWFLEFDIRKYFHTIDRHRLISIFKEEIDDLKFFYSIQKVFSDGRLVGGEKGPYSVP : 100
YP 173354    : MKEAIRMVLESIYDLEFPDTSHFRSGRGFHSVLRRIKEEWGTSRWFLEFDIRKCFHTIDRHRLIPIFKEEIDDPKFFYPIQKVFSAGRLVGGEKGPYSVP : 100
               MKEAIR6VLESIYDpEFPDTSHfRsGrG HS LRRIKeEWGtSRWFLEFDIRK FHtIDRHRLI IFKEEIdDpKFFY IqKVFSaGRlVGgE4GPYSVP

                          *       120         *       140         *       160         *       180         *       200
YP 009241663 : HCLLLSALPGNIYLHKLDQEIGRIRQKYEIPIVQRIRSVLLRTGRIDDQENSGEEASFNAPQDNRAIIVGSVKSLQRKAAFHSLVSSWHTPPTSTPRLRG : 200
YP 009121952 : HSVLLSALPGNIYLHKLDQEIGRIRQKYEIPIVQRIRSVLLRTGRIDDQEKSSEEASFNAPQDNRAIIVGRLKSIQRKAAFHSLVSSWHTPPTSTPRLRG : 200
YP 009045743 : HSVLLSALPGNIYLHKLDQEIGRIRQKYEIPIVQRIRSVLLRTGRIDDQENPGEEASFNAPQDNRAIIVGRVKSMQRKAAFHSLVSSWHTPPTSTLRLRG : 200
YP 009041166 : HSVLLSALPGNIYLHKLDQEIGRIRQKYEIPIVQRIRSVLLRTGRIDDQENSGEEASFNAPQDNRAIIVGRVKSIQRKAAFHSLVSSWHTSPTSTPRLRG : 200
YP 008999573 : HSVLLSALPDNIYLHKLDQEIGRIQQKYEIPIVQRIRSVLLRTGRIDDQEDSGEEASFNPPQDNRAIIVGRVKSIQRKAAFHSLVSSWHTPPASTPRLRG : 200
MATR SAMI    : HSVLLSALPGNIYLHKLDQDIGRIRQKYEIPIVQRIRSVLLRTGHIDDQENSGEEASFNAPQDNRAIIVGRVKSIQRKAAFHSLVSSWHTPPTSTPRLMG : 200
YP 008964116 : HSVLLSALPGNIYLHKLDQDIGRIRQKYEIPIVQRIRSVLLRTGRIDDQENSGEEASFNAPQDNRAIIVGRVKSIQHRSSFHSLVSSWHTPPTSTPRLMG : 200
YP 006460156 : HSVLLSALPGNIYLHKLDQDIGRIRQKYEIPIVQRIRSVLLRTGRIDDQENSGEEASFNAPQDNRAIIVGRVKSIQRKAAFHSLVSSWHTPPTSTPRLRG : 200
YP 006291793 : HSVLLSALPGNIYLHKLDQEIGRIRQKYEIPIVQRIRSVLLKTGRIDDQENSGEEASFNAPQDNRAFIVGRVKSIQRKAAFHSLVSSWHTPPTSTPRLRG : 200
YP 005090427 : HSVLLSALPGNIYLHKLDQEIGRIRQKYEIPIVQRIRSVLLRTGHIDDQENSGEEASFNAPQDNRAIIVGRVKSIQRKTAFHSLVSSCHTPPTSTPLCRG : 200
YP 173354    : HSVLLSALPGNIYLHKLDQEIGRIRQKYEIPIVQRIRSVLLRTGRIDDQEKSSEEASFNAPQDNRAIIVGRLKSIQRKAAFHSLVSSWHTPPTSTPRLRG : 200
               Hs6LLSALPgNIYLHKLDQ IGRIrQKYEIPIVQRIRSVLL4TGrIDDQE sgEEASFNaPQDNRAiIVGr6KS6Qr4aaFHSLVSSwHTpPtSTprlrG

                          *       220         *       240         *       260         *       280         *       300
YP 009241663 : DQKTPFVFPPSSALAAFLNKPSSLLCAAFLIEAAGLTPKAEFYGRERCNNNWAMRDLIKYCK--RKGPLIELGGEAILVIRSERRLARKLAPLKTHYLIR : 298
YP 009121952 : DQKRPFVFHPSSALAAFLNKPSSLLCAAFFIEAAGFTRKSEFYGRERCNNNWAMRDSFKYCK--RKGPLIELGGEAILVIRSERGLARKLAPLKTYYLIR : 298
YP 009045743 : DQKMSFVFPPSSALAVFLNKPSSLLCAAFLIEAAGLTPKAEFYGGERCNNNWAIRDLIKYCK--RKGLLIDLGGEAILVIRSERGLARKLAPLKSHYLIR : 298
YP 009041166 : DQKRPFVFPPSSALAAFLNKPSSLLCAAFLVEAAGLTPKAEFYGREHCNNNWAMRDFFKYCKSKRKGLLIELGGEAIIVIRSERGLARKLAPLKSHYLIR : 300
YP 008999573 : DQKRSFVFPPSSALAAFLNKPSSLLCAAFLIEAAGLTPKAEFYGRERCNNNWAMRDFFKYCK--RKGLLIELGGEAILVIRSERGLARKLAPLKSHYLIR : 298
MATR SAMI    : DQKRPFVFPLSSALAAFLNKPSSLLCAAFLIEAAGLTPKAEFYGRERCNNNWAMRDFFKSCK--RK--LIELGGEAILVIRSERGLARKLAPLKSYYNIR : 296
YP 008964116 : DQKRPFVFHSSSALAAFLNKPSSLLCAAFLIEAAGLTPKAEFYGRERCNNNWAMRDFFKSCK--RKGLLIELGGEARLVIRSERGLARKLAPLKSYYNIR : 298
YP 006460156 : DQKRPFVFPPSSALGAFLNKPSSLLCAAFLIEAAGLTPKAEFYGREHCNNNWAMRDFFKSCK--RKGLLIELGGEAILVIRSERGLARKLAPLKTHYNIK : 298
YP 006291793 : DQKTPFVFPPSSALAAFLNKPSSLLCAAFLIEAAGLAPKAEFYGRERCNNNLAMSSFFKYCK--RKSLLIEPGREAVLVIGSERGLARKLAPLKSHYLIR : 298
YP 005090427 : DQKTPFIFPPSSALAALLNKPSSLLCAAFLIEAFGLTPKAEFYGTERCNNNWAMRYFFKSCK--RKGLLIELGVEAILVIRSERGLTRKLAPLKTHYNIR : 298
YP 173354    : DQKTPFVFHPSSALAAFLNKPSSLLCAAFFIEAAGFTRKSEFYGRERCNNNWAMRDSFKYCK--RKGPLIELGGEAILVIRSERGLARKLAPLKTYYLIR : 298
               DQK pF6F pSSALaafLNKPSSLLCAAFl6EAaGltpKaEFYGrErCNNNwA6rd fK CK  RKg LIelGgEA 6VIrSERgLaRKLAPLK3 Y I4
```

```
                          *       320         *       340         *       360         *       380         *       400
YP 009241663 : ICYARYADDSLLGIVGAIELLIEIQKRIAHFLQSGLNLLVGSAGSTTIAARSTVEFPGTVIREVPPRTTPIQFLRELEKRLRVKHRIHITACHLRSAIHS : 398
YP 009121952 : ICYARYADDLLLGIVGSVELLIEIQKRIAHFLQSGLNLWVDSAGSTTIAARSTVEFLGTVIREVPPRATPIQFLRELEKRLRVKHRIHITACHLRSAIHS : 398
YP 009045743 : ICYARYADDLLLGIVGAVELLIEIQKRIAHFLQSGLNLWVGSAGSTTIAARSTVEFLGTVIREVPPRTTPIQFLRELEKRLRVKHRIHITACHLRSAIHS : 398
YP 009041166 : ICYARYADDLLLGIVGAVELLIEIQKRIAHFLQSGLNLWVDSAGSTTIAARSTVEFLGTVIREVPPRTTPIQFLRELEKRLRVKHRIHITACHLRSAIHS : 400
YP 008999573 : ICYARYADDLLLGIVGAVELLIEIKKLLAHFLQSGLNLWVGSAGSTTIAARSTVEFLGTVIREVPPRTTPIQFLRELEKRLRVKQRIHITACHLRSAIHS : 398
MATR SAMI    : ICYARYADDLLLGIVGAVELLIEIQKRINHFLQSGLNLWVDSAGSTTIAARSTVEFLGTVIREVPPRTTPIQFLRELEKRLRVKHRIHITACHLRSAIHS : 396
YP 008964116 : ICYARYADDLLLGIVGAVELIIEIQKRINHFLKSGLNLWVDSAGSTTIAARSTVEFLGTVIREVPPGTTPIQFLRELEKRLRVKHRIHITACHLRSAIHS : 398
YP 006460156 : ICYARYADDLLLGIVGAVELLIEIQKSINHFLQSGLNLWVDSAGSTTIAARSTVEFLGTVIREVPPRMTPIQFLRELEKRLRVKHRIHITACHLRSAIHS : 398
YP 006291793 : ICYARYADDLLLGIVGAVELLIEIQKRIAHFLQSGLNLWVGSAGSTTIAARSTIEFLGTVIREVPPRTTPIQLLRELEKRLRVKHRIHITACHLRSAIHS : 398
YP 005090427 : ICYARYADDLLLGIVGAVELLIEIQKRINHFLQYGLNLWVDSAGSTTIAARSTVEFLGTVIREVPPRTTPIQFLRELEKRLRVKHRIHITACHLRSAIHS : 398
YP 173354    : ICYARYADDLLLGIVGSVELLIEIQKRIAHFLQSGLNLWVDSAGSTTIAARSTVEFLGTVIREVPPRATPIQFLRELEKRLRVKHRIHITACHLRSAIHS : 398
               ICYARYADDlLLGIVGa6EL6IEIqKr6 HFLqsGLNLwV SAGSTTIAARST6EFlGTVIREVPPr TPIQfLRELEKRLRVKhRIHITACHLRSAIHS

                          *       420         *       440         *       460         *       480         *       500
YP 009241663 : KFRNLGNSIPIKQLTKGMSETGSLLDGVQLAETLGTAGVRGPQVSVLWGTVKHIRQGSRG-ISLLHSSGRSNAPSDVQQAVSRSGMSVRKLSLYTPAGRK : 497
YP 009121952 : KFRNLGNSIPIKELTKGMSGRGSLLDAVQLAETLGTAGVRSPQVSVLWGAVKHIRQGSRE-ISLLHSSGRSKVPSDVQQVVSRSGTHAPTLSLYTPAGRK : 497
YP 009045743 : KFRNLGDSIPIKQLTKGMSKTGSLLDGVQLAETLGTAGVRSPQVSVLWGTVKHIRQGSRG-ISLLHSSGRSNASSDVQQAVSRSGTHARKLSLYTPTGRK : 497
YP 009041166 : KFRNLGNSIPIKQLTKGMSGTGSLLDAVQLAETFGTAGVRSPQVSVLWGTVKHIRQGSRG-IPLLHSSGRSKVPSDVQQAVSRSGTHARKLSLYTPAGRK : 499
YP 008999573 : KFRNLGNSIPIKELTKGMSGTGSLLDAVQLADTLGTAGVRSPQVSVLWGTVKHIRQGSRA-LSLLHSSGRSKVPSDVQQAVSRSGMSVRKLSLYTPVGRK : 497
MATR SAMI    : KLRNLGNSIPIKQLTKGMSGTGSLLDAVQLAETLGTAGVRSPQVSVLWGTIKHIRQGSRG-ISLLHSSGRSKVPSD--QAVSRSGTHARKLSLYTPAGRK : 493
YP 008964116 : KLRNLGNSIPIKQLTKGMSGTGSLLDAVQLAETLGTAGVRSPQVSVLWGTIKHIRQGSRGGISLLHSSGRSKVPSDVQQAVSRSGMSARKLSLYTPAGRK : 498
YP 006460156 : KFRNLGNSIPIKQLTKGMSGTGSLLDAVQLAETLGTAGVRSPQVSVLWGTVKHIRQGSRG-ISLLHSSGRSKVPSDVQQAVSRSGTHARKLSLYTPAGRK : 497
YP 006291793 : KFRNLGNSIPIKELTKGMSGTGSLLDAVQLAETLGTAGVRSPQVSVLWGTVKHIRQGSRG-ISLLHSSGRSKAPSDVQQAVSRSGMSVRKLSLYTPASRK : 497
YP 005090427 : KFRNLRNSIPIKQLTKGMSGTGSLLDAVQLAETLGTAGVRGPQVSVLWGTVKHIRQGSRG-ISLLHSSGRSKVPSDVQQAVSRSVTHARKLSLYTPAGRK : 497
YP 173354    : KFRNLGNSIPIKELTKGMSGTGSLLDAVQLAETLGTAGVRSPQVSVLWGAVKHIRQGSRE-ISLLHSSGRSKVPSDVQQVVSRSGTHAPTLSLYTPAGRK : 497
               KfRNLg1SIPIK2LTKGMSgtGSLLDaVQLAeTlGTAGVRsPQVSVLWGt6KHIRQGSR  6sLLHSSGRSk pSDvqQaVSRSg   rkLSLYTPagRK

                          *       520         *       540         *       560         *       580         *       600
YP 009241663 : AAGEGGGHWAGSISSEFPIQ--IEAPIKKILRRLRDRGIISRRRPWPIHVACLTNVSDGDIVNWSAGIAISPLSYYRCRDNLYQVRTIVDHQIRWSAIFT : 595
YP 009121952 : AAGEGGGHWARSISSEFPIQ--IEAPIRKILRRLRDRGLISRRRPWPIHVACLTNVSDGDIVNWSAGIAISPLSYYRCCDNLYQVRTIVDHQIRWSAIFT : 595
YP 009045743 : AAGEGGGHWAGSISSEFPIQ--IEAPIKKILRRLRDRGIISRKRPWPIHVACLTNVSDEDIVNWSAGIAISPLSYYRCRDNLYQVRTIVDHQIRWSAIFT : 595
YP 009041166 : AAGEGGGHWARSISSEFPIQIEIEAPIKKILRRLRDRGLISRRRPWPIHVACLTNVSDGDIVNWSAGIAISPLSYYRCRDNFYQVRTIVDHQIRWSAIFT : 599
YP 008999573 : AAGEGEGDWARSIRSEFPIQ--IEAPIKKILRRLRDRGLISRRRPWPIHVACLTNVSDGDIVNWSAGIAISPLSYYRCRDNLYQVRTIVDHQIRWSAIFT : 595
MATR SAMI    : AAGEGGGHWARSISSEFPIQ--MEAPIKKILRRLRDRGLISRRRPWPIHVACLTNVSDEDIVNWSAGIAISPLSYYRCRDNLYQVRTIVDHQIRWSAIFT : 591
YP 008964116 : AAGEGGGHWARSISSEFPIQ--MEAPIKKILRRLRDRGLISRRRPWPIHVACLTNVGDEDIVNWSTGIAISPLSYYRCRDNLYQVRTIVDHQIRWSAIFT : 596
YP 006460156 : AAGEGGGHWARSISSELPIQ--MEAPIKKILRRLRDRGLISRRRPWPIHVACLTNVSDEDIVNWSAGIAISPLSYYRCHDNLYQVRTIVDHQIRWSAIFT : 595
YP 006291793 : AAGEGGGHWARSISSEFPIQ--IEAPIKKILRRLRDRGLISRRRPWPIHVACLTNVSDGDIVNWSAGIAISPLSYYRCRDNLYQVRTIVDHQIRWSAIFT : 595
YP 005090427 : AAGEGGGHWARSISSEFPIQ--MGAPIKKILRRLRDRGLISRRRPWPIHVDCLTNVSDGDIINWSAGIAISPLSYYRCRDNLYQVRTMVDHQIRWSAIFT : 595
YP 173354    : AAGEGGGHWARSISSEFPIQ--IEAPIKKILRRLRDRGLISRRRPWPIHVACLTNVSDGDIVNWSAGIAISPLSYYRCCDNLYQVRTIVDHQIRWSAIFT : 595
               AAGEGgGhWArSIsSEfPIQ  6eAPI4KILRRLRDRG6ISR4RPWPIHVaCLTNVsD DI6NWSaGIAISPLSYYRC DNlYQVRT6VDHQIRWSAIFT
```

```
                       *       620         *       640         *       660         *
YP 009241663 : LAHKHKSSARNIIPKYSKDSNIVNQEGGKTLAEFPNSIELGKLGPGQDPNNKEHSTTSLV------------------ : 655
YP 009121952 : PAHKHKSSARNIILKYSKDSNIVNQEGGKTLAEFPNSIELGKLGSGQDPNNNEHSTTSKKGEKVDFEKEGASCRFISK : 673
YP 009045743 : LAHKHKSSARNIIPKYSKDSNIVNPEGGKTLAEFPNSIELGKLGPGQDMNNKEHSTTSLV------------------ : 655
YP 009041166 : PAHKHKSSARNIIPKYSKDSNIVNQEGGKTIAEFPNSIELGKLGSGQDPNNKEHSTTSLV------------------ : 659
YP 008999573 : PAHKHKSSARNIIPKYSKDSNIVNKEGGKTLAEFPNSIELGKLGPGQDPNNKEHSITSLV------------------ : 655
MATR SAMI     : PAHKHKSSARNIIPKYSKDSNRVNQEGGKTLAEFPNSIELGKLGSGQDPKNKEHSTTSLV------------------ : 651
YP 008964116 : PAHKHKSSARNIIPKYSKDSNRVNQEG--ALAEFPNSIELGKLGSGQDPKNKEHSTTSLV------------------ : 654
YP 006460156 : PAHKHKSSARNIIPKYSKDSNRVNQEDGKILAEFPNSIELGKLGSGQDPKNKEHSTTSLV------------------ : 655
YP 006291793 : PAHKHKSSARNIIPKYSKDSNIVNQEGGKTLAEFPNSIELGKLGSGQDPNNKEHSTTSLV------------------ : 655
YP 005090427 : PAHKHKCSARNIIPKYSKDSNIVNQKGGKTLAEFPNSIELGKLGSGQDPNNKEHSTTSLV------------------ : 655
YP 173354     : PAHKHKSSARNIILKYSKDSNIVNQEGGKTLAEFPNSIELGKLGSGQDPNNNEHSTTSKKGES--------------- : 658
               pAHKHKsSARNIIpKYSKDSN VNqeggkt6AEFPNSIELGKLG GQDp NkEHStTSlv
```

Figure S3.17 The amino acid sequence allignment of MTTB in mitogenome of *S. miltiorrhiza* and the corresponding homologs in other species

```
                          *        20         *        40         *        60         *        80         *       100
YP 009243652 : MNRILSNIYISPLNYSYISYEFHFAPETILGEVRIRSVRILIGLGLTWFTRYWFPEELISPLAKPFLTLPLDSYFVRTQSTEASPTYVATSSIACSYFVF : 100
YP 009121951 : MNRILSNIYISPLNYSYISYEFHFAPETILGEVRIRSVRILIGLGLTWFTRYWFPEELISPLAKPFLTLPLDSYFVRTQSTEAFPTYVATSPIACSYFVF : 100
YP 009049757 : ---ILSNISIFPLNYSYISYEFHFAPETILGEVRIRSVRILIGLGLTWFTRYWFPEELIFPLAKPFLTLPLDSYFVRTQSTEAFPTYVATSPIACSYFVF :  97
YP 009045716 : ------------LNYSYISYEFDFALETILGEVRIRSLRILIGLGLTWFTRYWFPEELISPLAKPFLTLPFDSYFVCTQSTEAFPTYVATSSIACSYFVF :  88
YP 008999587 : -----------------MSYEFHFAPETILGEVRIRSLRILIGLGLTWFTRYWFPEELISPLAKPFLTLPLDSYFVRTQSTEASPTYVATSSIACSYFVF :  83
MTTB SAMI     : ------------MNFSYISYEFHFAPETILGEVRIRSVRILIGLGLTWFTRYWFPEELISPLAKPFLTLPFDSYFVRTQSTEAFPTYVATSSIACSYFVF :  88
YP 008964117 : --MISGRSSIFPLNFSYISYEFHFAPETLLGEVRIRSVRILIGLGLTWFTRYWFPEELIYPLAKPFLTLPFDSYFVRTQSTEAFPTYVATSSIACSYFVF :  98
YP 006460157 : ----------------YISYEFHFAPETILGEVRIRSVRILIGLGLTWFTRYWFPEELISPLAKPFLTLPFDSYFVRTQSTEAFPTYVATSSIACSYFVF :  84
YP 005090428 : --MMAGRSSIFPLNFSYISYEFHFAPETILGEVRIRSVRILIGLGLTWFTRYWFPEELISPLAKPFLTLPFDSYFVRTQSTEAFSTYVTTSSIACSYFVF :  98
YP 002608217 : ------------ITYKYISYEFHFAPETILGEVRIRSVRILIGLGLTWFTRYWFPEELISPLAKPFLTLPLDSYFVCTQSTEAFPTYVATSSIACSYFVF :  88
YP 173481     : MNRILSNIYIYPLNYSYISYEFHFAPETILGEVRIRSVRILIGLGLTWFTRYWFPEELISPLAKPFLTLPLDSYFVRTQSTEAFPTYVATSPIACSYFVF : 100
                                y6SYEFhFApET6LGEVRIRS6RILIGLGLTWFTRYWFPEELIsPLAKPFLTLP DSYFVrTQSTEAfpTYVaTS IACSYFVF

                          *       120         *       140         *       160         *       180         *       200
YP 009243652 : PLISHQIWCFLIPSCYGEQRTKYNRFLYLSGSRFSLFLFLTPPRVVPNVWHFPYFVGATSTNSLMIKLQPKIYDHIMLTVRISFIPSVCSQVPVIVIRLP : 200
YP 009121951 : PLISHQIWCFLIPSCYGEQRTKYNRFLHLSGSRFSLFLFLTPPRVVPNVWHFPYFMGATSTNSLMIKLQPKIYDHIMLTVRISFIPSVCSQVPVIVIRLP : 200
YP 009049757 : PLISHQIWCFLIPSCYEEQRTKYNRFLYLSGSRFSLFLFLTPPRVVPNVWHFPYFMGATSTNSLMIKLEAKIYDHIMLTVRISFIPSVCSQVPVIVIRLP : 197
YP 009045716 : PLISYQIWCFLIPSCYGEQRTKYNRFLHLSGSRFSLFLFLTPPRVVPNVWHFPYFVGATSTNSLMIKLQPKIYDHIMLTVRISFIPSVCSQIPVIVIRLP : 188
YP 008999587 : PLISHQIWCFLIPSCYGEQRTKYNRFLHLSGSRFSLFLFLTLSRVVPNVWHFPYFMGATSTNSLMIKLQPKIYDHIMLTVRISFIPSVCSQVPVIVIRLP : 183
MTTB SAMI     : PLISHQIWCFLIPSCYGEQRRKYNRFLHLSGSRFSLLLFLTLPRVVPNVWHFPYFMGETSTNSLMIKLQPKIYDHIMLTVRISFIPSVCSQVPVIVIRLL : 188
YP 008964117 : PLISHQIWCFFIPSCYGEQRRKYNRFLHLSGSRFSLLLFLTLPRVVPNVWHFPYFMGETSTNSLMIKLQPKIYDHIMLTVRISFIPSVCSQVPVIVIRLL : 198
YP 006460157 : PLISHQIWCFLIPSCYGEQRRKYNRFLHLSGSRFSLFLFLTLPRVVPNVWHFPYFMGETSTNSLMIKLQPKIYDHIMLTVRISFIPSVCSQVPVIVIRLL : 184
YP 005090428 : PLISHQIWCFLIPSCYMEQRRKYNRFLHLSGSRFSLFLFLTLPRVVPNVWHFPYFMGETSTNSLMIKLQPKIYDHIMLTVRISFIPSVCSQVPVIVIRLL : 198
YP 002608217 : PLISYQIWCFLIPCSYGEQRTKYNRFLHLSGSRFSLFLFLTPPRVVPNVWHFPYFVGETSTNSLMIKLQPKIYDHIMLTVRISFIPSVCSQVPVIVIRLP : 188
YP 173481     : PLISHQIWCFLIPSCYGEQRTKYNRFLHLSGSRFSLFLFLTPPRVVPNVWHFPYFMGATSTNSLMIKLQPKIYDHIMLTVRISFIPSVCSQVPVIVIRLP : 200
               PLIShQIWCFlIPscYgEQR KYNRFLhLSGSRFSLfLFLT pRVVPNVWHFPYF6G TSTNSLMIKL2pKIYDHIMLTVRISFIPSVCSQ6PVIVIRL

                          *       220         *       240         *       260         *       280         *       300
YP 009243652 : ELRGLSVETFTNNRRFLMVFPLLTAALSTPPDIWCQIVALFLISSIIELAIFVASIVQVREEGWTSGMRESGSID--KKEE-------------------- : 279
YP 009121951 : EPRGLSVETSTNNRRFLMVFPLLTAALSTPPDIWCQIVAPFLISLIIELAIFVASIVQVREEGWTSGMRESGSID--KKEE-------------------- : 279
YP 009049757 : EPRDLSVETSTNSRRFLMVFPLLTAALSTPPDIWCQIVAPFLISLIIELAIFVASIVQVREEGWTSGMRESGSID--KKEAVFRTACS------------ : 283
YP 009045716 : EPRGLSVETFTNNRRFLMVFPLLTAALSTPPDIWCQIVARFLISFIIELAIFVASIVQVREEGWTSGMRESGSIE--KKEE-------------------- : 267
YP 008999587 : EPRALSVETSTNNRRFLMVFPLLTAALSTPPDIWCQIVARFLISSIIELAISVASIVQVREEGWTSGMRESGSID--KKEE-------------------- : 262
MTTB SAMI     : EPRGLSVETSTNNRRFLMVFPLLTAALSTPPDIWCQIVARFLISLIIELAIFVASIVQVREEGWTSGMRESGSID--KKEE-------------------- : 267
YP 008964117 : EQRALSVETSTNNRRFLMVFPLLTAALSTPPDIWCQIVARFLISLIIELAIFVASIVQVREEGWTSGMRESGSID--RRVAP------------------ : 278
YP 006460157 : EPRGLSVETSTNNRRFLMVFPLLTAALSTPPDIWCQIVARFLISLIIELAIFVASIVQVREEGWTSGMRESGSID--KK---------------------- : 261
YP 005090428 : EPRGLSVETSTNNRRFLMVFPLLTAALSTPPDIWCQIVARFLISLIIELAIFVASIVQVREEGWTSGMRESGSIDKKRRVAPLEPGKVIINININEFQSN : 298
YP 002608217 : EPRGLSVETSTNNRRFLMVFPLLTAALSTPPDIWCQIVARFLISLIIELAIFVASIVQVREEGWTSGMRESGSID--KKEE-------------------- : 267
YP_173481     : EPRGLSVETSTNNRRFLMVFPLLTAALSTPPDIWCQIVAPFLISLIIELAIFVASIVQVREEGWTSGMRESGSID--KKEE-------------------- : 279
```

```
             EpR LSVETsTNnRRFLMVFPLLTAALSTPPDIWCQIVA FLIS IIELAIfVASIVQVREEGWTSGMRESGSId  44

                        *      320       *
YP 009243652 : --------------------------------- :   -
YP 009121951 : --------------------------------- :   -
YP 009049757 : --------------------------------- :   -
YP 009045716 : --------------------------------- :   -
YP 008999587 : --------------------------------- :   -
MTTB SAMI    : --------------------------------- :   -
YP 008964117 : --------------------------------- :   -
YP 006460157 : --------------------------------- :   -
YP 005090428 : SQPTDVIHSRKDYNTQKTPYLRSGMSDTSGARR : 331
YP 002608217 : --------------------------------- :   -
YP 173481    : --------------------------------- :   -
```

Figure S3.18 The amino acid sequence allignment of NAD1 in mitogenome of *S. miltiorrhiza* and the corresponding homologs in other species

```
                             *        20         *        40         *        60         *        80         *       100
YP 009049642 : MYIAVPAEILGIILPLLLGVAFLVLAERKVMAFVQRRKGPDVVGSFGLLQPLADGLKLILKEPISPSSANFSLFRMAPVATFMLSLVARAVVPFDYGMVL : 100
YP 009041160 : MYIAVPAEILGIILPLLLGVAFLVLAERKVMAFVQRRKGPDVVGSFGLLQPIADGLKLILKEPISPSSANFSLFRMAPVTTFMLSLVAWAVVPFDYGMVL : 100
YP 008999604 : MYIAVPAEILGIILPLLLGVAFLVLAERKVMAFVQRRKGPDVVGSFGLLQPIADGLKLILKEPISPSSANFSLFRMAPVATFMLSLVARAVVPFDYGMVL : 100
NAD1 SAMI     : MYIAVPAEILGIILPLLLGVAFLVLAERKVMAFVQRRKGPDVVGSFGLLQPLADGLKLILKEPISPSSANFSLFRMAPVATFMLSLVARAVVPFDYGMVL : 100
YP 006460148 : MYIAVPAEILGIILPLLLGVAFLVLAERKVMAFVQRRKGPDVVGSFGLLQPLADGLKLILKEPISPSSANFSLFRMAPVATFMLSLVARAVVPFDYGMVL : 100
YP 006291792 : MYIAVPAEILGIILPLLLGVAFLVLAERKVMAFVQRRKGPDVVGSFGLLQPLADGLKLILKEPISPSSANFSLFRMAPVATFMLSLVARAVVPFDYGMVL : 100
YP 005090403 : MYIAVPAEILGIILPLLLGVAFLVLAERKVMAFVQRRKGPDVVGSFGLLQPLADGLKLILKEPISPSSANFSLFRMAPVATFMLSLVARAVVPFDYGMVL : 100
YP 004935334 : MYIAVPAEILGIILPLLLGVAFLVLAERKVMAFVQRRKGPDVVGSFGLLQPLADGSKLILKEPISPSSANFSLFRMAPVTTFMLSLVARAVVPFDYGMVL : 100
YP 004237246 : MYIAVPAEILGIILPLLLGVAFLVLAERKVMAFVQRRKGPDVVGLFGLLQPLADGSKLILKEPISPSSANFSLFRMAPVATFMLSLVARAVVPFDYGMVL : 100
YP 173351     : MYIAVPAEILGIILPLLLGVAFLVLAERKVMAFVQRRKGPDVVGSFGLLQPLADGLKLILKEPISPSSANFSLFRMAPVATFMLSLVARAVVPFDYGMVL : 100
NP 064011     : MYIAVPAEILGIILPLLLGVAFLVLAERKVMAFVQRRKGPDVVGSFGLLQPLADGSKLILKEPISPSSANFSLFRMAPVTTFMLSLVARAVVPFDYGMVL : 100
               MYIAVPAEILGIILPLLLGVAFLVLAERKVMAFVQRRKGPDVVGsFGLLQP6ADG KLILKEPISPSSANFSLFRMAPV TFMLSLVArAVVPFDYGMVL

                             *       120         *       140         *       160         *       180         *       200
YP 009049642 : SDPNIGLLYLFAISSLGVYGIIIAGWSSNSKYAFLGALRSAAQMVPYEVSIGLILIT----VLICVGSRNSSEIVMAQKQIWSGIPLFPVLVMFFISRLA : 196
YP 009041160 : SDPNIGLLYLFAISSLGVYGIIIAGWSSNSKYAFLGALRSAAQMVSYEVSIGLILIT----VLICVGPRNSSEIVMAQKQIWSGIPLFPVLVMFFISCLA : 196
YP 008999604 : SDPNIGLLYLFAISSLGVYGIIIAGRSSNSKYAFLGALRSAAQMVSYEVSIGLILIVRLVTVLICVGSCNLSEIVMAQKQIWFGIPLFPVLVMFFISCLA : 200
NAD1 SAMI     : SDPNIGLLYLFAISSLGVYGIIIAGRSSNSKYASLGALRSAAQMVPYEVSIGLILIT----VLICVGPRNSSEIVMAQKQIWSGIPLFPVLVMFFISCLA : 196
YP 006460148 : SDPNIGLLYLFAISSLGVYGIIIAGRSSNSKYASLGALRSAAQMVPYEVSIGLILIT----VLICVGPRNSSEIVMAQKQIWSGIPLFPVLVMFFISRLA : 196
YP 006291792 : SDPNIGLLYLFAISSLGVYGIIIAGRSSNSKYAFLGALRSAAQMVPYEVSIGLILIT----VLICVGPRNSSEIVMAQKQIWSGIPLFPVLVMFFISRLA : 196
YP 005090403 : SDPNIGLLYLFAISSLGVYGIIIAGRSSNSKYAFLGALRSAAQMVSYEVSIGLILIT----VLICVGPRNSSEIVMAQKQIWSGIPLFPVLVMFFISRLA : 196
YP 004935334 : SDPNIGLLYLFAISSLGVYGIIIAGWSSNSKYAFLGALRSAAQMVPYEVSIGLILIT----VLICVGPRNSSEIVMAQKQIWSGIPLFPVLVMFFISCLA : 196
YP 004237246 : SDPNIGLLYLFAISSLGVYGIIIAGRSSNSKYAFLGALRSAAQMVSYEVSIGLILIT----VLICVGPRNSSEIVMAQKQIWSGIPLFPVLVMFLISCLA : 196
YP 173351     : SDPNIGLLYLFAISSLGVYGIIIAGWSSNSKYASLGALRSAAQMVPYEVSIGLILIT----VLICVGSRNSSEIVMAQKQIWSGIPLFPVLVMFFISRLA : 196
NP 064011     : SDPNIGLLYLFAISSLGVYGIIIAGWSSNSKYAFLGALRSAAQMVPYEVSIGLILIT----VLICVGPRNSSEIVMAQKQIWSGIPLFPVLVMFFISCLA : 196
               SDPNIGLLYLFAISSLGVYGIIIAG SSNSKYA LGALRSAAQMV YEVSIGLILIt    VLICVG rNsSEIVMAQKQIWsGIPLFPVLVMFfIS LA

                             *       220         *       240         *       260         *       280         *       300
YP 009049642 : ETNRAPFDLPEAEAESVAGYNVEYSSMGSALSFLGEYANMILMSGLCTSLSPGGWPPILDLPISKKIPGSIWFSIKVILFLFLYIWVRAAFPRYRYDQLM : 296
YP 009041160 : ETNRAPFDLPEAEAELVAGYNVEYSSMGSALFFLGEYANMILMG-PCTSLSPGGWPPILDLPISKKIPGSIWFSIKVILFLFLYIWVRAAFPRYRYDQLM : 295
YP 008999604 : ETNRAPFDLPEAEAESVAGYNVEYARDAILNSSLLAEANVPGSRGLILTETRGGWLPILDLSIFKKIPGSIWFSIKVILFLFLYIWVRAAFPRYRYDQLM : 300
NAD1 SAMI     : ETNRAPFDLPEAEAESVAGYNVEYAR--DAILNSSLLAEANVPG-PCTSLSPGGWPPILDLPISKKIPGSIWFSIKVILFLFLYIWVRAAFPRYRYDQLM : 293
YP 006460148 : ETNRAPFDLPEAEAESVAGYNVEYSSMGSALFFLGEYANMILMSGPCTSLSPGGWPPILDLPISKKIPGSIWFSIKVILFLFLYIWVRAAFPRYRYDQLM : 296
YP 006291792 : ETNRAPFDLPEAEAESVAGYNVEYSSMGSALSFLGEYANMILMRCPCTSLSPGGWPPFLDLPISKKIPGSIRFSIKVILFPFLYIWVRAAFPRYRYDQLM : 296
YP 005090403 : ETNRAPFDLPEAEAESVAGYNVEYA--RDAILNSSLLAEANVLG-PCTSLSPGGWPPILDLPISKKIPGSIWFSIKVILFLFLYIWVRAAFPRYRYDQLM : 293
YP 004935334 : ETNRAPFDLPEAEAELVAGYNVEYSSMGSALFFLGEYANMILMSGLCTLLSPGGWPPILDLPISKKIPGSIWFSIKVILFLFLYIWVRAAFPRYRYDQLM : 296
YP 004237246 : ETNRAPFDLPEAEAESVAGYNVEYSSMGSALFFLGEYANMILMSGPCTSLSPGGWPPILDLPIFKKIPGSIWFSIKVILFLFLYIWVRAAFPRYRYDQLM : 296
YP 173351     : ETNRAPFDLPEAEAESVAGYNVEYSSMGSALSFLGEYANMILMSGLCTSLSPGGWPPILDLPISKKIPGSIWFSIKVILFLFLYIWVRAAFPRYRYDQLM : 296
NP 064011     : ETNRAPFDLPEAERELVAGYNVEYSSMGSALFFLGEYANMILMSGLCTSLSPGGWPPILDLPISKRIPGSIWFSIKVILFLFLYIWVRAAFPRYRYDQLM : 296
               ETNRAPFDLPEAEaE VAGYNVEY     a   l   An      ct l3pGGWpPiLDLpIsK4IPGSIwFSIKVILFlFLYIWVRAAFPRYRYDQLM
```

```
                         *      320
YP 009049642 : GLGRKVFLPLSLARVVPVSGVLVTFQWLP : 325
YP 009041160 : GLGRKVFLPLSLARVVPVSGVLVTFQWLP : 324
YP 008999604 : GLGRKVFLPLSLARVVAVSGVLVTFQWLP : 329
NAD1 SAMI    : GLGRKVFLPLSLARVVVVSGVLVTFQWLP : 322
YP 006460148 : GLGRKVFLPLSLARVVVVSGVLVTFQWLP : 325
YP 006291792 : GLGRKVFLPLSLARVVPVSGVLVTFQWLP : 325
YP 005090403 : GLGRKVFLPLSLARVVVVSGVLVTFQWLP : 322
YP 004935334 : GLGRKVFLPLSLARVVAVSGVLVTFQWLP : 325
YP 004237246 : GLGRKVFLPLSLARVVPVSGVLVTFQWLP : 325
YP 173351    : GLGRKVFLPLSLARVVPVSGVLVTFQWLP : 325
NP 064011    : GLGRKVFLPLSLARVVAVSGVLVTFQWLP : 325
               GLGRKVFLPLSLARVV VSGVLVTFQWLP
```

Figure S3.19 The amino acid sequence allignment of NAD2 in mitogenome of *S. miltiorrhiza* and the corresponding homologs in other species

```
                         *        20         *        40         *        60         *        80         *       100
YP 009241652 : MFNLFLAVSPEIFIINATFILLIHGVVFSTSKKYDYPPLVSNVGWLGLLSVLIT---LLLLAAGAPLLTIAHLFRNNLFRRDNFTYFCQILLLLSTAGTI :  97
YP 009177612 : MFNLFLAVSPEIFIINATSILLIHGVVFSTSKKYDYPPLVSNVGWLGLLSVLIT---LLLLAAGAPLLTIAHLFWNNLFRRDNFTYFCQILLLLSTAGTI :  97
YP 009041151 : MFNLFLAVSPEIFIINATFILLIHGVVFSTSKKYDYPPLVSNVGWLGLLSVLIT---LLLLAAGAPLLTIAHLFWNNLFRRDNFTYFCQILLLLSTAGTI :  97
YP 008999580 : MFNLFLAVSPEIFIINATFILLIHGVVFSTSKKYDYPPLVSNVGWLGLLSVARLGGQRALGCGGAIIATIAHLFWNNLFRRDNFTYFCQILLLLSTAGTI : 100
NAD2 SAMI     : MFNLFLAVSPEIFIINATFILLIHGVVFSTSKKYDYPPLVSNVGWLGLLSVT-----------------IYNIFWNNLFRRDNFTYFCQILLLLSTAGTI :  83
YP 008964105 : MFNLFLAVSPEIFLINATFILLIHGVVFSTSKKYDYPPLVSNVGWLGLLSVLIT---LLLLAAGAPLLTIAYLFWNNLFRRDNFTYFCQILLLLSTAGTI :  97
YP 006666123 : MFNLFLAVSPEIFIINATFILLIHGVVFSTSKKYDYPPLVSNVGWLGLLSVLIT---LLLLAAGAPLLTIAHLFWNNLFRRDNFTYFCQILLLLSTAGTI :  97
YP 006460152 : MFNLFLAVSPEIFLINATFILLIHGVVFSTSKKYDYPPLVSNVGWLGLLSVLIT---LLLLAAGAPLLTIAYLFWNNLFRRDNFTYLCQILLLCCTAGTI :  97
YP 006291809 : MFNLFLAVSPEIFIINATFILLIHGVVFSTSKKYDYPPLVSNVGWLGLLSVLIT---LLLLAAGAPLLTIAHLFWNNLFRRDNFTYFCQILLLLSTAGTI :  97
YP 005090405 : MFNLFLAVSPEIFIINATFILLIHGVVFSTSKKYDYPPLVSNVGWLGLLSVLIT---LLLLAAGAPLLTIAYLFWNNLFRRDNFTYFCQILLLLSTAGTI :  97
YP 002608182 : MFNLFLAVSPEIFIINATSILLIHGVVFSTSKKYDYPPLVSNVGWLGLLSVLIT---LLLLAAGAPLLTIAHLFWNNLFRRDNFTYFCQILLLLSTAGTI :  97
               MFNLFLAVSPEIF6INATfILLIHGVVFSTSKKYDYPPLVSNVGWLGLLSVlit   llllaagap ltIa 6FwNNLFRRDNFTYfCQILLLlsTAGTI

                         *       120         *       140         *       160         *       180         *       200
YP 009241652 : SMCFDSSEQERFDAFESIVLIPLPTRSMLFMISAHDSIAMYLAIEPQSLCFYVIAASKRKSEFSTEAGSKYLILGAFPSGILLFGCSMIYGSTGATHFDQ : 197
YP 009177612 : SMCFDSFEQERFDAFEFIVLIPLPTRSMLFMISAHDSIAMYLAIEPQSLCFYVIAASKRKSEFSTEAGSKYLILGAFSSGILLFGCSMIYGSTGATHFDQ : 197
YP 009041151 : SMCFDSSEQERFDAFEFIVLIPLPTRSMLFMISAYDSIAMYLAIEPQSLCFYVIAASKRKSEFSTEAGSKYLILGAFPSGILLFGCSMIYGSTGATHFDQ : 197
YP 008999580 : SMCFDSSEQERFDASESIVLIPLPTCSMLFMISAHDSIAMYLAIEPQSLCFYVIAASKRKSEFSTEAGSKYLILGAFSSGILLFGCSMIYGSTGATHFDQ : 200
NAD2 SAMI     : SMCFDSSEQERFDAFEFIVLIPLPTRSMLFMISAYDSIAMYLAIEPQSLCFYVIAASKRKSEFSTEAGSKYLILGAFSSGILLFGCSMIYGSTGATHFDQ : 183
YP 008964105 : SMCFDSSEQERFDAFEFIVLIPLPTRSMLFMISAYDSIAMYLAIEPQSLCFYVIAASKRKSEFSTEAGSKYLILGAFSSGILLFGCSMIYGSTGATHFDQ : 197
YP 006666123 : SMCFDSSEQERFDAFEFIVLIPLPTRSMLFMISAHDSIAMYLAIEPQSLCFYVIAASKRKSEFSTEAGSKYLILGAFPSGILLFGCSMIYGSTGATHFDQ : 197
YP 006460152 : SMCFDSSEQERFDAFEFIVLIPLPTRSMLFMISAYDSIAMYLAIEPQSLCFYVIAASKRKSEFSTEAGSKYLILGAFSSGILLFGCSMIYGSTGATHFDQ : 197
YP 006291809 : SMCFDSSEQERFDASESIVLIPLPTRSMLFMISAYDSIAMYLAIEPQSLCFYVIAASKRKSEFSTEAGSKYLILGAFPSGILLFGCSMIYGSTGATHFDQ : 197
YP 005090405 : SMCFDSSEQERFDAFEFIVLIPLPTRSMLFMISAYDSIAMYLAIEPQSLCFYVIAASKRKSEFSTEAGSKYLILGAFSSGILLFGCSMIYGSTGATHFDQ : 197
YP 002608182 : SMCFDSSEQERFDAFEFIVLIPLPTRSMLFMISAHDSIAMYLAIEPQSLCFYVIAASKRKSEFSTEAGSKYLILGAFSSGILLFGCSMIYGSTGATHFDQ : 197
               SMCFDSsEQERFDAfE IVLIPLPTrSMLFMISA DSIAMYLAIEPQSLCFYVIAASKRKSEFSTEAGSKYLILGAF SGILLFGCSMIYGSTGATHFDQ

                         *       220         *       240         *       260         *       280         *       300
YP 009241652 : LAKILTGYEITGARSSGIFMGILFIAVGFLFKITAVPFHMWAPDIYEGSPTPVTAFLSIAPKISISANISRVSIYGSYGATLQQIFFFCSIASMILGALA : 297
YP 009177612 : LAKILTGYEITGARSSGIFMGILFIAVGFLFKITAVPFHMWAPDIYEGSPTPVTAFLSIAPKISISANISRVSIYGSYGATLQQIFFFCSIASMILGALA : 297
YP 009041151 : LAKILTGYEITGARSSGIFMGILFIAVGSLFKITAVPFHMWAPDIYEGSPTPVTAFLSIAPKISISANISRLSIYGSYGATLQQIFFFCSIASMILGALA : 297
YP 008999580 : LAKILTGYEITGARSSGIFMGILSIAVGSLFKITAVPFYMWAPDIYEGSPTPVTAFLSIAPKISISANISRVSIYGSYGATLQQIFFFCSIASMILGALA : 300
NAD2 SAMI     : LAKILTGYEITGARSSGIFMGILFIAVGFLFKITAVP-YMWAPDIYEGSPTPVTAFLSIAPKISISANISRLSIYGSYGATLQQIFFFCSIASMILGALA : 282
YP 008964105 : LAKIWTGYEITGARSSGIFMGILFIAIGFLFKITAVPFHMWAPDIYEGSPTPVTAFLSIAPKISISANISRLSIYGSYGATLQQIFLFCSIASMILGALA : 297
YP 006666123 : LAKILTGYEITGARSSGIFMGILSIAVGFLFKITAVPFHMWAPDIYEGSPTPVTAFLSIAPKISISANISRVSIYGSYGATLQQIFFFCSIASMILGALA : 297
YP 006460152 : LAKILTGYEITGARSSGIYMGILFIAVGFLFKITAVPFHMWAPDIYEGSPTPVTAFLSIAPKISISANISRVSIYGSYGDTLQQIFFFCSIASMILGALA : 297
YP 006291809 : LAKILTGYEITGARSSGIFMGILSIAVGSLFKITAVPFYMWAPDIYEGSPTPVTAFLSIAPKISISANISRVSIYGSYGATLQQIFFFCSIASMILGALA : 297
YP 005090405 : LAKILTGYEITGARSSGIFMGILFIAVGFLFKITAVPFYMWAPDIYEGSPTPVTAFLSIAPKISISANISRVSIYSSYGATLQQIFFFCSIASMILGALA : 297
YP_002608182 : LAKILTGYEITGARSSGLFMGILSIAVGFLFKITAVPFHMWAPDIYEGSPTPVTAFLSIAPKISISANILRVSIYGSYGATLQQIFFFCSIASMILGALA : 297
```

```
                     LAKIlTGYEITGARSSG65MGIL IA6G LFKITAVPf MWAPDIYEGSPTPVTAFLSIAPKISISANIsR6SIYgSYGaTLQQIFfFCSIASMILGALA

                                *       320         *       340         *       360         *       380         *       400
YP 009241652 : AMAQTKVKRPLAHSSIGHVGYIRTGFSCGTIEGIQSLLIGIFIYASMTIDAFAIVSALRQTRVKYIADLGALAKTNPISAITFSITMFSYAGIPPLAGFC : 397
YP 009177612 : AMAQTKVKRPLAHSSIGHVGYIRTGFSCGTIEGIQSLLIGIFIYALMTIDAFAIVSALRQTRVKYIADLGALAKTNPISAITFSITMFSYAGIPPLAGFC : 397
YP 009041151 : AMAQTKVKRLLAHSSIGHVGYIRTGLSCGTIEGIQSLLIGIFIYASMTIDAFAIVLALRQTRVKYIADLGALAKTNPILAITFSITMFSYAGIPPLAGFC : 397
YP 008999580 : AMAQTKVKRLLAYSSIGHVGYIRTGFSCGTIEGIQSLLIGIFIYASMTIDAFAIVSALRQTRVKYIADLGALAKTNPISAITFSITMFSYAGIPPLAGFC : 400
NAD2 SAMI     : AMAQTKVK---------------------------------------------------------------------------------------------- : 290
YP 008964105 : AMAQTKVKRLLAHSSIGHVGYIRTGFSCGTIEGIQSLLIGIFIYASMTIDAFAIVSALRQTRVKYIADLGALAKTNPILAITFSITMFSYVGIPPLAGFY : 397
YP 006666123 : AMAQTKVKRPLAHSSIGHVGYIRTGLSCGTIEGIQSLLIGIFIYALMTIDAFAIVLALRQTRVKYIADLGALAKTNPISAITFSITMFSYAGIPPLAGFC : 397
YP 006460152 : AIAQTKVKRLLAHSSIGHVGYIRIGLSCGTIEGIQSLLMAIFIYASMTIDAFAIVSALRQTRVKYIADLGALAKTNPILAITFSITMFSYIGIPPLAGFF : 397
YP 006291809 : AMAQTKVKRLLAHSSIGHVGYIRTGFSCGTIEGIQSLLIGIFIYASMTIDAFAIVSALRQTRVKYIADLGALAKTNPISAITFSITMFSYAGIPPLAGFC : 397
YP 005090405 : AMAQTKVKRLLAHSSIGHVGYIRTGFSCGTIEGIQSLLIGIFIYASMTIDAFAIVSALRQTRVKYIADLGALAKTNPILAITFSITMFSYAGIPPLAGFC : 397
YP 002608182 : AMAQTKVKRPLAHSSIGHVGYICTGFSCGTIEGIQSLLIGIFIYALMTIDAFAIVSALRQTRVKYIADLGALAKTNPISAITFSITMFSYAGIPPLAGFC : 397
               A6AQTKVKr lahssighvgyirtg scgtiegiqsll gifiya mtidafaiv alrqtrvkyiadlgalaktnpi aitfsitmfsy gipplagf

                                *       420         *       440         *       460         *       480         *
YP 009241652 : SKFYLFFAALGCGAYFLAPVGVVTSVIGRFYYIRLAKRMFFDTPRTWILYEPMDRDKSLLLAMTSSFITSFFPYPSPLFSVTHQMALSLYL : 488
YP 009177612 : SKFYLFFAALGCGAYFLAPVGVVTSVIGCFYYIRLAKRMFFDTPRTWILYEPMDRNKSLLLAMTSSFITSSFLYPSPLFSVTHQMALSSYL : 488
YP 009041151 : SKFYLFFAALGCGAYFLAPVGVVTSVIGCFYYIRLAKRMFFDTPRTWILYEPMDRDKSLLLAMTSSFITSFFPYPSPLFSVTHQMALSSYL : 488
YP 008999580 : SKFYLFFAALGCGAYFLAPVGVVTSVIGRFYYIRLVKRMFFDTPRTWILYEPMDRDKSLLLAMTSSFITSFFPYPSPLFSVTHQMALSSYL : 491
NAD2 SAMI     : ----------------------------RFYYIRLAKRMFFDTPRTWILYEPMDRDKSLLLAMTSSFITSFFPYPSPLFSVTHQMALSSYL : 353
YP 008964105 : SKFYLFFAALGCGATFLALVGVVSSVIGRFYYIRLAKRMFFDTPRTWILYEPMDRDKSLLLAMTSSFITSFFPYPSPLFSVTHQMALSSYL : 488
YP 006666123 : SKFYLFFAALGCGAYFLAPVGVVTSVIGRFYYIRLAKRMFFDTPRTWILYEPMDRDKSLLLAMTSSFITSSFSYPSPLFSVTHQMALSSYL : 488
YP 006460152 : SKFYLFFAALACGAYLLALVGVVTSVIGRFYYIRLAKRMFFDTPRTWILYEPMDRDKSLLLAMTSSFITSFFPYPSPLFSVTHQMALSSYL : 488
YP 006291809 : SKFYLFFAALGCGAYFLAPVGIVTSVIGRFYYIRLAKRMFFDTPRTWILYEPMDRDKSLLLAMTSSFITSFFPYPSPLFSVTHQMALSSYL : 488
YP 005090405 : SKFYLFFAALGCGAYFLAPVGVVTSVIGRFYYIRLAKRMFFDTPRTWILYEPMDRDKSLLLAMTSSFITSFFPYPSPLFSVTHQMALSSYL : 488
YP 002608182 : SKFYLFFAALGCGAYFLAPVGVVTSVIGRFYYIRLAKRMFFDTPRTWILYEPMDRNKSLLLAMTSSFITSSFLYPSPLFSVTHQMALSSYL : 488
               skfylffaalgcgayfla vg v svigrFYYIRLaKRMFFDTPRTWILYEPMDR1KSLLLAMTSSFITS F YPSPLFSVTHQMALSsYL
```

Figure S3.20 The amino acid sequence allignment of NAD3 in mitogenome of *S. miltiorrhiza* and the corresponding homologs in other species

```
                            *        20         *        40         *        60         *        80         *       100
YP 009252180 : MSEFSPIFIYLVMSPLVSLIPLGLPFPFASNSSTYPEKLSAHECGSDPSGDARSRFDIRFYLVPILFIIPDPEVTFSSPWAVPPNKIDPFGSWSMMAFLL : 100
YP 009241665 : MSEFAPICIYLVISSLVSLIPLGVPFPFASNSSTYPEKLSAYECGFDPSGDARSRFDIRFYLVSILFIILDPEVTFSFPWAVPLNKIDPFGSWSMMAFLL : 100
YP 009121960 : MSEFAPICIYLVISPLVSLIPLGLPFIFSSNSSTYPEKLSAYECGFDPSGDARSRFDIRFYLVSILFIIPDPEVTFSFPWAVPPNKIDPFGSWSMMAFLL : 100
YP 009049747 : MSEFAPICIYLVISPLVSLIPLGLPFLFSSNSSTYPEKLSAYECGFDPSGDARSRFDIRFYLVSILFIIPDPEVTFSFPWAVPPNKIDPFGSWSMMAFLL : 100
YP 009041157 : MSEFAPIFIYLVISPLVSLILLGLPFPFSSNSSTYPEKLSAYECGFDPSGDARSRFDIRFYLVSILFIIPDPEVTFSFPWAVPPNKIDPFGSWSMMAFLL : 100
NDA3 SAMI    : MSEFEPILIYLVISSLVSLIPLGLPFPFSSNSSTYPEKLSAHECGFDPSGDARSRFDIRFYLVSILFIIPDPEVTFSFPWAVPPNKIDPFGSWSMMAFLL : 100
YP 008964104 : MSEFSPILIYLVISSLVSLIPLGLPFPFSSNSSTYPEKLSAYECGFDPFGDARSRFDIRFYLVSILFIISDPEVTFSFPWAVPPNKIDPFGSWSMMAFLL : 100
YP 006460185 : MSEFSPILIYLVISSLVSLIPLGVPFPFSSNSSTYPEKLSAYECGFDPFGDARSRFDIRFYLVSILFIIPDPEVTFSFPWAVPPNKIDPFGSWSMMAFLL : 100
YP 006280934 : MSEFAPICIYLVISPLVSLIPLGLPFPFASNSSTYPEKLSAHECGSDPSGDARSRFDIRFYLVSILFIIPDPEVTFSFPWAVPPNKIDSFGSWSMMAFLL : 100
YP 005090425 : MSEFSPILIYLVISSLVSLIPLGLPFPFSSNSSTYPEKLSAYECGFDPSGDARSRFDIRFYLVSILFIIPDPEVTFSFPWAVPPNKIDPFGSWSMMAFLL : 100
YP 002608384 : MSEFAPICIYLVISPLVSLIPLGVPFPFASNSSTYPEKLSAYECGFDPFGDARSRFDIRFYLVSILFIIPDPEVTFSFPWAVPPNKIDPFGSWSMMAFLL : 100
               MSEF PI IYLV6S LVSLIpLG6PFpF SNSSTYPEKLSA ECGfDP GDARSRFDIRFYLVsILFIIpDPEVTFSfPWAVPpNKIDpFGSWSMMAFLL

                            *
YP 009252180 : ILTIGSLYEWKRGASDRE : 118
YP 009241665 : ILTIGSLYEWKRGASDRE : 118
YP 009121960 : ILTIGSLYEWKRGASDRE : 118
YP 009049747 : ILTIGSLYEWKRGASDRE : 118
YP 009041157 : ILTIGSLYEWKRGALDRE : 118
NDA3 SAMI    : ILTIGSLYEWKRGASDRE : 118
YP 008964104 : ILTIGSLYEWKRGASDRE : 118
YP 006460185 : ILTIGSLYEWKRGASDRE : 118
YP 006280934 : ILTIGSLYEWKRGASDRE : 118
YP 005090425 : ILTIGSLYEWKRGASDRE : 118
YP 002608384 : ILTIGSLYEWKRGASDRE : 118
               ILTIGSLYEWKRGAsDRE
```

Figure S3.21 The amino acid sequence allignment of NAD4 in mitogenome of *S. miltiorrhiza* and the corresponding homologs in other species

```
                           *        20         *        40         *        60         *        80         *       100
YP 009230375 : MLEHFCECYSDLSGPILCPVLGSITPLFIPNSRIRPIRLIGLCASLITFLYSPAPRIQFDPSTAKSQFVESLRWLPYENIHFDLGIDGISLFFVILTTFL : 100
YP 009041184 : MLEHFCECYSNLSGLILCPVLGSITPLFIPNSRIRPIRLIGLCVSLITFLYPPVLRIQFDPSTAKSQFVESLRWLPYENINFYLGIDGISLFCVILTTFL : 100
NAD4 SAMI     : MLE---QWYFDLSGLILSPVLGSITPLFIPNSRIRPIRLIGLCASLITFLYSPVLRIQFDPSTAKSQFVESLRWLPYENIHFDLGIDGISLFCVILTTFL :  97
YP 008964101 : MLE---QWYFDLSGLILSPVLGSITPLFIPNSRIRPIRLIGLCASLITFLYPPVLRIQFDPSTAKSQFVESPRWLTYENIHFDLGIDGISLFFVILTTFL :  97
YP 006460174 : MLE---QWYFDLSGLILSPVLGSITPLFIPNSRIRPIRLIGLCASLITFLYPPALRIQFDPSTAKSQFVESLRWLPYENINLDLGIDGISLFCVILTTFL :  97
YP 006291798 : MLEYFCECYSDLSGPILCPVLGSITPLFIPNSRIRPIRLIGLCASLITFLYPPVLRIQFDPSTAKSQFVESLRWLPYENIHFYLGIDGISLFFVILTTFL : 100
YP 005090410 : MLE---QCYFDLSGLILCPVLGSITPLFIPNSRIRPIRLIGLCASLITFLYPPVLRIQFDPSTAKSQFVESLRWLPYENINFYLGIDGISLFFVILTTFL :  97
YP 004927458 : MLEHFCECYSNLSGLILCPVLGSITPLFIPNSRIRPIRLIGLCASLITFLYSPVPRIQFDSSTAKSQFVESLRWLPYENINFYLGIDGISLFFVILTTFL : 100
YP 004237266 : MLEHFCECYSDLSGLILCPVLGSITPLFIPNSRIRPIRLIGLCASLITFLYSPVLRIQFDPSTAKSQFVESLRWLPYENIHFFFGIDGISLFFVILTTFL : 100
YP 173416     : MLEHFCECYSDLSGLILCPVLGSITPLFIPNSRIRPIRLIGLCASLITFLYPPVLRIQFDPSTAKSQFVESLRWLPYENINFYLGIDGISLFFVILTTFL : 100
NP 085518     : MLEHFCECYSNLSGLILCPVLGSITLLFIPNSRIRPIRLIGLCASLITFLYSPVPRIQFDSSTAKSQFVESLRWLPYENINFYLGIDGISLFFVILTTFL : 100
               MLE   2 Y 1LSGlIL PVLGSITpLFIPNSRIRPIRLIGLCaSLITFLY Pv RIQFDpSTAKSQFVESlRWLpYENI f lGIDGISLF VILTTFL

                           *       120         *       140         *       160         *       180         *       200
YP 009230375 : IPICILVGWSGMRSYGKEYITASLIREFLMIAVFRMLDLLLFYVLPESVLIPMFIIIGVWGSRQRKIKAAYQFFLYTLLGSVFMLLAILLILLQTGTTDL : 200
YP 009041184 : IPICILVGWSGMRSYGKEYITASLIREFLMIAVFCMLDPLLFYVLPESVLIPMFIIIGVWGSRQRKIKAAYQFFLYTLLGSVFMLLAILLILLQTGTTDL : 200
NAD4 SAMI     : IPICILVGWSGMRSYGKEYITASLIREFLMIAVFCMLDPLLFYVLPESVPIPMFIIIGVWGSRQRKIKAAYQFFLYTLLGSVFMLLAILLILFQTGTTDL : 197
YP 008964101 : IPICILVGWSGMRSYGKEYITASLIREFLMITVFCMLDPLLFYVLPESVPIPMFIIIGVWGSRQRKIKAAYQFFLYTLLGSVFMLLAIMFILFQTGTTNL : 197
YP 006460174 : IPICILVGWSGMRSYGKEYITASLIRECGMIAVFCILDSILFYVFPESVPIPMFIIIGVWGSRQRKIKAAYQFFLYTLLGSVFMLLAILLILLQTGTTDL : 197
YP 006291798 : IPICISVGWSGMRSYGKEYITASLIREFLMIAVFRMLDPLLFYVFPESVPIPMFIIIGVWGSRQRKIKAAYQFFLYTLLGSVFMLLAILLILLQTGTTDL : 200
YP 005090410 : IPICILVGWSGMRSYGKEYITASLIREFIMIAVFCMLDPLLFYVLPESVPIPMLIIIGVWGSRQRKIKAAYQFFLYTLLGSVFMLLAILLILLQTGTTDL : 197
YP 004927458 : IPICISVGWSGMRSYGKEYITAFLIREFLMIAVFRMLDLLLFYVFPESVPIPMFIIIGVWGSRQRKIKAAYQFFLYTLLGSLFMLLAILLILFQTGTTDL : 200
YP 004237266 : IPICISVGWSGMRSYGKEYITASLIREFLMIAVFRMLDPLLFYVLPESVLIPMFIIIGVWGSRQRKIKAAYQFFLYTLLGSVFMLLAILLILLQTGTTDL : 200
YP 173416     : IPICILVGWSGMRSYGKEYITAFLIREFIMIAVFCMLDLLLFYVLPESVLIPMFIIIGVWGSRQRKIKAAYQLFLYTLLGSVFMLLAILLILLQTGTTDL : 200
NP 085518     : IPICISVGWSGMRSYGKEYITAFLIREFLMIAVFRMLDLLLFYVFPESVPIPMFIIIGVWGSRQRKIKAAYQFFLYTLLGSLFMLLAILLILFQTGTTDL : 200
               IPICI VGWSGMRSYGKEYITA LIREf MIaVF 6LD 6LFYV PESV IPMfIIIGVWGSRQRKIKAAYQfFLYTLLGS6FMLLAI6lIL QTGTT1L

                           *       220         *       240         *       260         *       280         *       300
YP 009230375 : QISLTTEFSERRQIFLWIASFASFAVKVPMVPVHIWLPEAHVEAPTAGSVILAGIPLKLGTYGFLRFSIPMFPEATLFSTPFIYTPSAIAIIYTSLTTSR : 300
YP 009041184 : QISLTTEFSERRQIFLWIASFASFAVKVPMVPVHIWLPEAHVEAPTAGSVLLAGIPLKLGTYGFLRFSIPMFPEATLCSTPFIYTSSAIAIIYTSSTTLR : 300
NAD4 SAMI     : QISLTTEFSERRQIFLWIASFASFAVKVPMVPVHIWLPEAHVEAPTAGSVILAGIPLKLGTYGFLRFSIPMFPEATLCSTPFIYTPSAIAIIYTSSTTLR : 297
YP 008964101 : QISLTTEFSERRQIFLWIASFAAFAVKVPMVPVHIWLPEAHVEAPTAGSVILAGIPLKFGTYGFLRFSIPMFPEATLCSTPFIYTPSAIAIIYTSSTTLR : 297
YP 006460174 : QISLTTEFSERRQIFLWIASFAAFAVKVPMVPVHIWLPEAHVEAPTAGSVILAGIPLKLGTYGFLRFSIPMFPEATLCSTPFIYTPSAIAIIYTSSTTLR : 297
YP 006291798 : QISLTTEFSERRQIFLWIASFASFAVKVPMVPVHIWSPEAHVEAPTAGSVILAGIPLKLGTYGFLRFSIPMFPEATLCSTPFIYTSSAIAIIYTSSTTLR : 300
YP 005090410 : QISLTTEFSERRQMFLWIASFASFAVKVPMVPVHIWLPEAHVEAPTAGSVILAGIPLKLGTYGFLRFSIPMFPEATLCSTPFIYTPSAIAIIYTSSTTLR : 297
YP 004927458 : QISLTTEFSERRQIFLWIASFASFAVKVPMVPVHIWLPEAHVEAPTAGSVILAGIPLKFGTHGFLRFSIPMFPEATLCSTPFIYTLSAIAIIYTSLTTSR : 300
YP 004237266 : QISLTTEFSERRQIFLWIASFASFAVKVPMVPVHIWLPEAHVEAPTAGSVILAGIPLKLGTYGFLRFSIPMFPEATLSSTPFIYTPSAIAIIYTSLTTSR : 300
YP 173416     : QISLTTEFSERRQIFLWIASFASFAVKVPMVPVHIWLPEAHVEAPTAGSVILAGIALKLGTYGFLRFSIPMFPEATLCSTPFIYTLSAIAIIYTSSTTLR : 300
NP 085518     : QISLTTEFSERRQIFLWIASFASFAVKVPMVPVHIWLPEAHVEAPTAGSVILAGIPLKFGTHGFLRFSIPMFPEATLCSTPFIYTLSAIAIIYTSLTTSR : 300
               QISLTTEFSERRQ6FLWIASFAsFAVKVPMVPVHIWlPEAHVEAPTAGSV6LAGIpLK GTyGFLRFSIPMFPEATLcSTPFIYT SAIAIIYTS TT R
```

```
                        *       320         *       340         *       360         *       380         *       400
YP 009230375 : QIDLKKIIAYSSVAHMNLVTIGMFSPNIQGIGGSILPMLSHGLVPSALFLCVGVLYDRHKTRLVRYYGGLVSTMPNLSTIFFSSTLANMSSPGTSSFIGE : 400
YP 009041184 : QIDLKKIIAYSSVAHMNLATIGMFSPNIQGIGGSILPMSSHGLVSSALFLCVGVLYDRHKTRLVRYYGGSVSTMPNLPTIFFSSTLANMSSPGTSSFIGE : 400
NAD4 SAMI     : QIDLKKIIAYSSVAHMNLVTIGMFSPNIQGIGGSILPMSSHGLVSSALFLCVGVLYDRHKTRLVRYYGGSVSTMPNLPTIFFFSTLANMSSPGTSSFIGE : 397
YP 008964101 : QIDLKKIIAYSSVAHMGLVTIGMFSPNIQGIGGSILPMSSHGLVSSALFLCVGVLYDRHKTRLVRYYGGSVSTMPNLPTIFFSSTLANMSSPGTSSFIGE : 397
YP 006460174 : QIDLKKIIAYSSVAHMNLVTIGMFSPNIQGIGGSILPMSSHGLVSSALFLCVGVLYDRHKTRLVRYYGGSVSTMPNLPTIFFSSTLANMSSPGTSSFIGE : 397
YP 006291798 : QIDLKKIIAYSPVAHMNLVTIGMFSRNIQGIGGSIPPMSSHGLVPSALFLCVGVLYDRHKTRLVRYYGGSVSTMPNLPTIFFSSTLANMSSPGTSSFIGE : 400
YP 005090410 : QIDLKKIIAYSSVAHMNLVTIGMFSRNIQGIGGSILPMSSHGLVSSALFLCVGVLYDRHKTRLVRYYGGLVSTMPNFPTIFFSSTLANMSSPGTSSFIGE : 397
YP 004927458 : QIDLKKIIAYSSVAHMNLVTIGMFSPNIQGIGGSILPMLSHGLVPSALFLCVGVLYDRHKTRLVRYYGGLVSTMPNLSTIFFSFTLANMSSPGTSSFIGE : 400
YP 004237266 : QIDLKKIIAYSSVAHMNLVTIGMFSPNIQGIGGSILPMLSHGLVSSALFLCVGVLYDRHKTRLVRYYGGLVSTMPNLSTIFFSSTLANMSSPGTSSFIGE : 400
YP 173416     : QIDLKKIIAYSSVAHMNLVTIGMFSPNIQGIGGSILPMSSHGLVSSALFLCVGVLYDRHKTRLVRYYGGSVSTMPNLSTIFFSSTLANMSSPGTSSFIGE : 400
NP 085518     : QIDLKKIIAYSSVAHMNLVTIGMFSPNIQGIGGSILPMLSHGLVPSALFLCVGVLYDRHKTRLVRYYGGLVSTMPNLSTIFFSFTLANMSSPGTSSFIGE : 400
               QIDLKKIIAYSsVAHMnLvTIGMFSpNIQGIGGSIlPM SHGLV SALFLCVGVLYDRHKTRLVRYYGG VSTMPNl TIFFssTLANMSSPGTSSFIGE

                        *       420         *       440         *       460         *       480         *
YP 009230375 : FLISVGAFQRNSLVATLSALGMILGAAYSLWLYNRVVSGNLKPDFLHKFSDSNGREVSIFIPFLVGLVRMGVHPKVFPDCMHTSVSNLVQHGKFH : 495
YP 009041184 : FLILVGAFQRNSLVATLAALGMILGAAYSLWLYNRVVSGNLKPDFLHKFSDPNGREVSIFIPFLVGVVRIGVHPKVFPDRMHTSVSNLVQHGKFH : 495
NAD4 SAMI     : FLILVGAFQRNSLVATLAALGMILGAAYSLWLYNRAVSGNLKPDFLHKFSDPNGREVSIFIPFLVGVVWMGVHPKVFPDCMHTSVSNLVQHGKFH : 492
YP 008964101 : FLILVGAFQRNSLVATLAALGMILGAAYSLWLYNRAVSGNLKPDFLHKFSDPNGREVSIFIPFIVGVVWMGVHPKVFPDCMHTSVSNLVQHGKFH : 492
YP 006460174 : FLILVGAFQRNSLVATLAALGMILGAAYSLWLYNRAVSGNLKPDFLHKFSDPNGREVSIFIPFLVGVVWMGVHPKVFPDCMHTSVSNLVQHGKFH : 492
YP 006291798 : FLILVGAFQRNSLVATLAALGMILGAAYSLWLYNRAVSGNLKPDFLHKFSDPNGREVSIFIPFLVGLVRMGVHPKVFPDRMHTSVSNLVQHGKFN : 495
YP 005090410 : FLILVGAFQRNSLVATLAALGMILGAAYSLWLYNRAVSGNLKPDFLHKFSDPNGREVSIFIPFIVGLVWMGVHPKVFPDCIHTSVSNLVQHGKFH : 492
YP 004927458 : FPILVGAFQRNSLVATLAALGMILGAAYSLWLYNRVVSGNLKPDFLHKFSDSNGREVSIFIPFLVGLVRMGVHPKVFPDCMHTSVSNLVQHGKFH : 495
YP 004237266 : FLISVGAFQKNSVVATLAALGMILGAAYSLWLYNRVVSGNLKPDFLHKFSDSNGREVSIFIPFLVGVVWMGVHPKVFPDCMHTSVSNLVQHGKFH : 495
YP 173416     : FLILVGAFQRNSLVATLAALGMILGAAYSLWLYNRAVSGNLKPDFLHKFSDPNGREVSIFIPFLVGVVRMGVHPKVFPDRMHTSVSNLVQHGKFH : 495
NP 085518     : FLILVGAFQRNSLVATLAALGMILGAAYSLWLYNRVVSGNLKPDFLHKFSDSNGREVSIFIPFLVGLVRMGVHPKVFPDCMHTSVSNLVQHGKFH : 495
               FlIlVGAFQ4NS6VATLaALGMILGAAYSLWLYNR VSGNLKPDFLHKFSD NGREVSIFIPF6VG6V 6GVHPKVFPD 6HTSVSNLVQHGKFh
```

Figure S3.22 The amino acid sequence allignment of NAD4L in mitogenome of *S. miltiorrhiza* and the corresponding homologs in other species

```
                         *        20         *        40         *        60         *        80         *       100
XP 002535062 : MDPIKYFTFSMIISILGIRGILLNRRNIPIMSMPIESMLLAVNLNFLVFSVSSDDMMGQSFASLVPTVAAAESAIGLAIFVITFRVRGTIAVEFINSIQG : 100
YP 009045755 : MDPIKYFTFSMIISILGIRGILLNRRNIPIMSMPIESMLLAVNSNFLVFSVSSDDMMGQSFASLVPTVAAAESAIGLAIFVITFRVRGTIAVEFINSIQG : 100
YP 008964114 : MDPIQYFTFSMIISISGIRGIILNRRNIPIMSMPIESMLLAVNSNFLVFSVSLDDMMGQSFASLVSTVAAAESAIGLAIFVITFRVRGTIAVESINSIQG : 100
YP 006460177 : MDPIKYFTFSMIISISGIRGILLNRRNIPIMSMPIESMLLAVNSNFLVFSVSSDDMMGQLFASLVSTVAAAESAIGLAIFVITFRVRGTIAVESINSIQG : 100
YP 005090409 : MDPIKYFTFSMIISISGIRGILLNRRNIPIMSMPIESMLLAVNSNFLVFSVSSDDMMGQSFASLVSTVAAAESAIGLAIFVITFRVRGTIAVESINSIQG : 100
YP 004849353 : MDPIKYFTFSMIISILGIRGILLNRRNIPIMSMPIESMLLAVNSNFLVFSVSSDDMMGQSFASLVPTVAAAESAIGLAIFVITFRVRGTIAVEFLNSIQG : 100
YP 004237262 : MDPIKYFTFSMIISILGIRGILLNRRNIPIMSMPIESMLLAVNLNFLVFSVSSDDMMGQSFASLVPTVAAAESAIGLAIFVITFRVRGTIAVEFINSIQG : 100
YP 004222831 : MDPIKYFTFSMIISILGIRGILLNRRNIPIMSMPIESMLLAVNSNFLVFSVSSDDMMGQSFASLVSTVAAAESAIGLAIFVITFRVRGTIAVEFINSIQG : 100
YP 002608364 : MDPIKYFTFSMIISILGIRGILLNRRNIPIMSMPIESMLLAVNSNFLVFSVSSDDMMGQSFASLVPTVAAAESAIGLAIFVITFRVRGTIAVESINSIQG : 100
YP 173389     : MDPIKYFTFSMIISISGIRGILLNRRNIPIMSMPIESMLLAVNSNFLVFSVSSDDMMGQSFASLVPTVAAAESAIGLAIFVITFRVRGTIAVESINSIQG : 100
NAD4L SAMI   : MDPIKYFTFSMIISISGIRGILLNRRNIPIMSMPIESMLLAVNSNFLVFSVSSDDMMGQSFASLVSTVAAAESAIGLAIFVITFRVRGTIAVESINSIQG : 100
               MDPIkYFTFSMIISI GIRGI6LNRRNIPIMSMPIESMLLAVNsNFLVFSVSsDDMMGQsFASLV TVAAAESAIGLAIFVITFRVRGTIAVE 6NSIQG

                         *       120         *       140         *       160         *       180         *       200
XP 002535062 : SGPFSLGELSSTNMQARKMLFAAILSICALSSKKISIYNEEMIVARCFIGFIIFSRKSLGNTFKVTLDGRIQAIQEESQQFLNPNEVVPPESNEQQRLLR : 200
YP 009045755 : ---------------------------------------------------------------------------------------------------- :   -
YP 008964114 : ---------------------------------------------------------------------------------------------------- :   -
YP 006460177 : ---------------------------------------------------------------------------------------------------- :   -
YP 005090409 : ---------------------------------------------------------------------------------------------------- :   -
YP 004849353 : ---------------------------------------------------------------------------------------------------- :   -
YP 004237262 : ---------------------------------------------------------------------------------------------------- :   -
YP 004222831 : ---------------------------------------------------------------------------------------------------- :   -
YP 002608364 : ---------------------------------------------------------------------------------------------------- :   -
YP 173389     : ---------------------------------------------------------------------------------------------------- :   -
NAD4L SAMI   : ---------------------------------------------------------------------------------------------------- :   -

                         *       220         *       240         *       260         *       280         *       300
XP 002535062 : ISLRICGTVVESLPMARCAPKCEKTVQALLCRNLNVKSATLSNATSSRRIRLQDDLVTGFHFSVSERFFPGCTLKASIVELIREGLVVLRMVRVGGSLKN : 300
YP 009045755 : ---------------------------------------------------------------------------------------------------- :   -
YP 008964114 : ---------------------------------------------------------------------------------------------------- :   -
YP 006460177 : ---------------------------------------------------------------------------------------------------- :   -
YP 005090409 : ---------------------------------------------------------------------------------------------------- :   -
YP 004849353 : ---------------------------------------------------------------------------------------------------- :   -
YP 004237262 : ---------------------------------------------------------------------------------------------------- :   -
YP 004222831 : ---------------------------------------------------------------------------------------------------- :   -
YP 002608364 : ---------------------------------------------------------------------------------------------------- :   -
YP 173389     : ---------------------------------------------------------------------------------------------------- :   -
NAD4L_SAMI   : ---------------------------------------------------------------------------------------------------- :   -
```

```
XP 002535062 : KEDE : 304
YP 009045755 : ---- :   -
YP 008964114 : ---- :   -
YP 006460177 : ---- :   -
YP 005090409 : ---- :   -
YP 004849353 : ---- :   -
YP 004237262 : ---- :   -
YP 004222831 : ---- :   -
YP 002608364 : ---- :   -
YP 173389    : ---- :   -
NAD4L SAMI   : ---- :   -
```

Figure S3.23 The amino acid sequence allignment of NAD5 in mitogenome of *S. miltiorrhiza* and the corresponding homologs in other species

```
                            *        20         *        40         *        60         *        80         *       100
YP 009177610 : ----------------------MYLLIVFLPLLGSGVAGFFGRFLGSEGTAIMTTTCVSFSSILSLIAFYEVAPGASACYLRIAPWISSEMFDASWGFLF :  78
YP 009121953 : ----------------------MYLLIVFLPLLGSFVAGCFGRFLGKEGTAIITTTCVSFSSIFSLIAFYEVAPGASACYLRIAPWISSEMFDASWGFLF :  78
YP 009049647 : ----------------------MYLLIVFLPLLGSFVAGCFGRFLGKEGTAIITTTCVSFSSIFSLIAFYEVAPGASACYLRIAPWISSEMFDASWGFLF :  78
YP 009041162 : ----------------------MYLLIVFLPLLGSGVAGFFGRFLGSEGTAIMTTTCLSFSSILSLIAFYEVAPGASACYLRIAPWISSEMFDASWGFLF :  78
NAD5 SAMI    : ----------------------MYLLIVFLPLLGSSVAGFFGRFLGSEGTAIITTTCVSFSSILSLIAFYEVAPGASACYLRVAPWISSEMFDASWGFVF :  78
YP 008964103 : ----------------------MYLLIVFLPLLGSSVAGFFGRFLGSEGTAIITTTCVSFSSIFSLIAFYEVAPGASACYLRVAPWISSEMFDASWGFLF :  78
YP 006460149 : ----------------------MYLLIVFLPLLGSSVAGFFGRFLGSEGTAIITTTCVSFSSIFSLIAFYEVAPGASACYLRVAPWISSEMFDASWGFLF :  78
YP 006291784 : ----------------------MYLLIVFLPLLGSSVAGFFGRFLGSEGTAIITTTCLSFSSILSLIAFYEVAPGASACYLRIAPWISSEMFDASWGFVF :  78
YP 005090406 : MGSKLLLVGFLRIPLSLGGGSSMYLLIVFLPLLGSSVAGFFGRFLGSEGSAIITTTCVSFSSILSLIAFYEVAPGASACYLRVAPWISSEMFDASWGFFF : 100
YP 004237247 : ----------------------MYLLIVFLPLLGSSIAGFFGRFLGSEGTAIMTTTCVSFSSILSLIAFYEVAPGASACYLRIAPWISSEMFDASWGFLF :  78
YP 173349     : ----------------------MYLLIVFLPLLGSFVAGCFGRFLGKEGTAIITTTCVSFSSIFSLIAFYEVAPGASACYLRIAPWISSEMFDASWGFLF :  78
                                      MYLLIVFLPLLGS 6AG FGRFLG EG3AI6TTTC6SFSSI SLIAFYEVAPGASACYLR6APWISSEMFDASWGF F

                            *       120         *       140         *       160         *       180         *       200
YP 009177610 : DSPTVVMLIVVTSISSLVHIYSISYMSEDPHSPRFMCYLSILTFFMPMLVTGDNSLQLFLGWEGVGLASYLLIHFWFTRLQADKAAIKAMPVNRVGDFGL : 178
YP 009121953 : DSPTVVMLIVVTFISSLVHLYSISYMSEDPHSPRFMCYLSIPTFFMPMLVTGDNSLQLFLGWEGVGLASYLLIHFWFTRLQADKAAIKAMLVNRVGDFGL : 178
YP 009049647 : DSPTVVMLIVVTFISSLVHLYSISYMSEDPHSPRFMCYLSIPTFFMPMLVTGDNSLQLFLGWEGVGLASYLLIHFWFTRLQADKAAIKAMLVNRVGDFGL : 178
YP 009041162 : DSPTVVMLIVVTFISSLVHLYSISYMSEDPHSPRFMCYLSIPTFFMPMLVTGDNSLQLFLGWEGVGLASYLLIHFWFTRLQADKAAIKAMLVNRVGDFGL : 178
NAD5 SAMI    : DSPTVVMLIVVTFISSLVHLYSISYMSEDPHSPRFMCYLSISTFFMPMLVTGDNSLQLFLGWEGVGLASYLLIHFWFTRLQADKAAIKAMLVNRVGDFGL : 178
YP 008964103 : DSPTVVMLIVVTFISSLVHLYSISYMSEDPHSPRFMCYLSISTFFMPMLVTGDNSLQLFLGWEGVGLASYLLIHFWFTRLQADKAAIKAMLVNRVGDFGL : 178
YP 006460149 : DSPTVVMLIVVTFISSLVHLYSISYMSEDPHSPRFMCYLSISTFFMPMLVTGDNSLQLFLGWEGVGLASYLLIHFWFTRLQADKAAIKAMLVNRVGDFGL : 178
YP 006291784 : DSPTVVMLIVVTSISSLVHLYSISYMSEDPHSPRFMCYLSIPTFFMPMLVTGDNSLQLFLGWEGVGLASYLLIHFWFTRLQADKAAIKAMLVNRVGDFGL : 178
YP 005090406 : DSPTVVMLIVVTFISSLVHLYSISYMSEDPHSPRFMCYLSISTFFMPMLVTGDNSLQLFLGWEGVGLASYLLIHFWFTRLQADKAAIKAMLVNRVGDFGL : 200
YP 004237247 : DSLTVVMLIVVTFISSLVHLYSISYMSEDPHSPRFMCYLSIFTFFMPMLVTGDNSLQLFLGWEGVGLASYLLIHFWFTRLQADKAAIKAMLVNRVGDFGL : 178
YP 173349     : DSPTVVMLIVVTFISSLVHLYSISYMSEDPHSPRFMCYLSIPTFFMPMLVTGDNSLQLFLGWEGVGLASYLLIHFWFTRLQADKAAIKAMLVNRVGDFGL : 178
               DSpTVVMLIVVTfISSLVH6YSISYMSEDPHSPRFMCYLSI TFFMPMLVTGDNSLQLFLGWEGVGLASYLLIHFWFTRLQADKAAIKAMlVNRVGDFGL

                            *       220         *       240         *       260         *       280         *       300
YP 009177610 : APGISGRFTLFQTVDFSTIFACASAPRNSWISCNMRLNAITLICILLLIGAVGKSAQIGSHTWSPDAMEGPTPVSALIHAATMVTAGVFMIARCSPLFEY : 278
YP 009121953 : APGILGCFTLFQTVDFSTIFARASAPRNSWISCNMRLNAITLICILLFIGAVGKSAQIGSHTWSPDAMEGPTPVSALIHAATMVTAGVFMIARCSPLFEY : 278
YP 009049647 : APGILGCFTLFQTVDFSTIFARASAPRNSWISCNMRLNAITLICILLFIGAVGKSAQIGSHTWSPDAMEGPTPVSALIHAATMVTAGVFMIARCSPLFEY : 278
YP 009041162 : APGISGCFTLFQTVDFSTIFACASAPRNSWISCNMRLNAITLICILLLIGAVGKSAQIGSHTWSPDAMEGPTPVSALIHAATMVTAGVFMIARCSPLFEY : 278
NAD5 SAMI    : APGISGCFTLFQTVDFSTIFACASAPRNSWISCNMRFNAITLICILLLIGAVGKSAQIGSHTWSPDAMEGPTPVSALIHAATMVTAGVFMIARCSPLFEY : 278
YP 008964103 : APGIPGCFTLFQTVDFSTIFACASAPRNSWISCNMRFNAITLICILLLIGAVGKSAQIGSHTWSPDAMEGPTPVSALIHAATMVTAGVFMIARCSPLFEY : 278
YP 006460149 : APGISGCFTLFQTVDFSTIFACASTPRNSWISCNMRLNAITLICILLLIGAVGKSAQIGSHTWSPDAMEGPTPVSALIHAATMVTAGVFMIARCSPLFEY : 278
YP 006291784 : APGISGCFTLFQTVDFSTIFARASAPRNSWISRNMRLNAITLICILLLIGAVGKSAQIGSHTWSPDAMEGPTPVSALIHAATMVTAGVFMIARCSPLFEY : 278
YP 005090406 : APGISGCFTLFQTVDFSTIFACANAPRNYWISCNMRLNAITLICILLLIGAVGKSAQIGSHTWSPDAMEGPTPVSALIHAATMVTAGVFMIARCSPLFEY : 300
YP 004237247 : APGISGRFTLFQTVDFSTIFARASAPRNSWISCNMRFNAITLICILLLIGAVGKSAQIGSHTWSPDAMEGPTPVSALIHAATMVTAGVFMIARCSPLFEY : 278
YP_173349    : APGILGCFTLFQTVDFSTIFARASAPRNSWISCNMRLNAITLICILLFIGAVGKSAQIGSHTWSPDAMEGPTPVSALIHAATMVTAGVFMIARCSPLFEY : 278
```

```
               APGI GcFTLFQTVDFSTIFA AsaPRNsWIScNMR NAITLICILL IGAVGKSAQIGSHTWSPDAMEGPTPVSALIHAATMVTAGVFMIARCSPLFEY

                        *       320         *       340         *       360         *       380         *       400
YP 009177610 : PPTALIVITFAGATTSFLAATTGILQNDLKRVIAYSTCSQLGYMIFACGISNYSVSVFHLMNHAFFKALLFLSAGSVIHAMSDEQDMRKMGGLASSFFPFT : 378
YP 009121953 : PPTALIVITFAGAMTSFLAATTGILQNDLKRVIAYSTCSQLGYMIFACGISNYSVSVFHLMNHAFFKALLFLSAGSVIHAMSDEQDMRKMGGLASSFFPFT : 378
YP 009049647 : PPTALIVITFAGAMTSFLAATTGILQNDLKRVIAYSTCSQLGYMIFACGISNYSVSVFHLMNHAFFKALLFLSAGSVIHAMSDEQDMRKMGGLASSFFPFT : 378
YP 009041162 : PPTALIVITFAGAMTSFLAATTGILQNDLKRVIAYSTCSQLGYMIFACGISNYSVSVFHLMNHAFFKALLFLSAGSVIHAMSDEQDMRKMGGLASSFFPFT : 378
NAD5 SAMI    : PPTALIVITFAGAMTSFLAATTGILQNDLKRVIAYSTCSQLGYMIFACGISNYSVSVFHLMNHAFFKALLFLSAGSVIHAMSDEQDMRKMGGLASSFFPFT : 378
YP 008964103 : PPTALIVITFAGAMTSFLAATTGILQNDLKRVIAYSTCSQLGYMIFACGISNYSVSVFHLMNHAFFKALLFLSAGSVIHAMSDEQDMRKMGGLASSFFPFT : 378
YP 006460149 : PPTALIVITFAGAMTSFLAATTGILQNDLKRVIAYSTCSQLGYMIFACGISNYSVSVFHLMNHAFFKALLFLSAGSVIHAMSDEQDMRKMGGLASSFFPFT : 378
YP 006291784 : PPTALIVITFAGAMTSFLAATTGILQNDLKRVIAYSTCSQLGYMIFACGISNYSVSVFHLMNHAFFKALLFLSAGSVIHAMSDEQDMRKMGGLASSFFPFT : 378
YP 005090406 : PPTALIVITFAGAMTSFLAATTGILQNDLKRVIAYSTCSQLGYMIFACGISNYSVSVFHLMNHAFFKALLFLSAGSVIHAMSDEQDMRKMGGLASSFFPFT : 400
YP 004237247 : SPTALIVITFAGAMTSFLAATTGILQNDLKRVIAYSTCSQLGYMIFACGISNYSVSVFHLMNHAFFKALLFLSAGSVIHAMSDEQDMRKMGGLASSFFPFT : 378
YP 173349    : PPSALIVITFAGAMTSFLAATTGILQNDLKRVIAYSTCSQLGYMIFACGISNYSVSVFHLMNHAFFKALLFLSAGSVIHAMSDEQDMRKMGGLASSFFPFT : 378
               pP3ALIVITFAGAmTSFLAATTGILQNDLKRVIAYSTCSQLGYMIFACGISNYSVSVFHLMNHAFFKALLFLSAGSVIHAMSDEQDMRKMGGLASSFFPFT

                        *       420         *       440         *       460         *       480         *       500
YP 009177610 : YAMMLMGSLSLIGFPFLTGFYSKDVILELAYTKYTISGNFAFWLGSVSVLFTSYYSFRLLFLTFLVPTNSFGRDILRCHDAPIPMAIPLILLALGSLFVG : 478
YP 009121953 : YAMMLMGSLSLIGFPFLTGFYSKDVILELAYTKYTISGNFAFWLGSVSVLFTSYYSFRSLFLTFLVPTNSFGRDILRCHDAPIPMAIPLILLALGSLFVG : 478
YP 009049647 : YAMMLMGSLSLIGFPFLTGFYSKDVILELAYTKYTISGNFAFWLGSVSVLFTSYYSFRSLFLTFLVPTNSFGRDILRCHDAPIPMAIPLILLALGSLFVG : 478
YP 009041162 : YAMMLIGSLSLIGFPFLTGFYSKDVILELAYTKYTISGNFAFWLGSVSVLFTSYYSFRSLFLTFLVPTNSFGRDILRCHDAPIPMAIPLILLALGSLFVG : 478
NAD5 SAMI    : YAMMLMGSLSLIGFPFLTGFYSKDVILELAYTKYTISGNFAFWLGSVSVLFTSYYSFRSLFLTFLVPTNSFGRDILRCHDAPIPMAIPLILLALGSLFVG : 478
YP 008964103 : YAMMLMGSLSLIGFPFLTGFYSKDVILELAYTKYTISGNFAFWLGSVSVLFTSYYSFRSLFLTFLVPTNSFGRDILRCHDAPIPMAIPLILLALGSLFVG : 478
YP 006460149 : YAMMLLGSLSLIGFPFLTGFYSKDVILELAYTKYTISGNFAFWLGSVSVLFTSYYSFRSLFLTFLVPTNSFGRDILRCHDAPIPMAIPLILLALGSLFVG : 478
YP 006291784 : YAMMLMGSLSLIGFPFPTGFYSKDVILELAYTKYTISGNFAFWLGSVSVLFTSYYSFRLLFLTFLVPTNSFGRDILRCHDAPIPMAIPLILLALGSLFVG : 478
YP 005090406 : YAMMLMGSLSLIGFPFLTGFYSKDVILELAYTKYTISGNFAFWLGSVSVLFTSYYSFRSLFLTFLVPTNSFGRDILRCHDAPIPMAIPLILLALGSLFVG : 500
YP 004237247 : YAMMLMGSLSLIGFPFLTGFYSKDVILELAYTKYTISGNFAFWLGSVSVLFTSYYSFRSLFLTFLVPTNSFGRDIVRCHDAPIPMAIPLILLALGSLFVG : 478
YP 173349    : YAMMLMGSLSLIGFPFLTGFYSKDVILELAYTKYTISGNFAFWLGSVSVLFTSYYSFRSLFLTFLVPTNSFGRDILRCHDAPIPMAIPLILLALGSLFVG : 478
               YAMML6GSLSLIGFPFlTGFYSKDVILELAYTKYTISGNFAFWLGSVSVLFTSYYSFRsLFLTFLVPTNSFGRDI6RCHDAPIPMAIPLILLALGSLFVG

                        *       520         *       540         *       560         *       580         *       600
YP 009177610 : YLAKDMMIGLGTNFWANSPFVLPKNEILAESEFAAPTITKLIPIPFSTLGASVAYNVNPVADQFQRAFQTSTFCNRLYSFFNKRWFFDQVLNDFLVRSFL : 578
YP 009121953 : YLAKDMMIGLGTNFWANSPFVLPKNEILAESEFAAPTITKLIPIPFSTSGAYVAYNVNPVADQFQRAFQTSTISNRLYSFFNKRWFFDQVLNDFLVRSFL : 578
YP 009049647 : YLAKYLNQSSYPNFWANSPFVLPKNEILAESEFAAPTITKLIPIPFSTSGAYVAYNVNPVADQFQRAFQTSTISNRLYSFFNKRWFFDQVLNDFLVRSFL : 578
YP 009041162 : YLAKDMMIGLGTNFWANSPFVLPKNEILAESEFAAPTITKLIPIPFSTSGASVAYNVNPVADQFQRAFQTSTFCNRLYTFFNKRWFFDQVLNDFLVRSFL : 578
NAD5 SAMI    : YFAS--------NFWANSPFVLPKNEILAESEFAAPTITKLIPIPFSTTGASVAYNVNSVADQFQRAFQTSTFCNRLYSFFNKRWFFDQVLNDFLVRSFL : 570
YP 008964103 : YFAKDMMIGLGTNFWANSPFVLPKNEILAESEFAAPTITKLIPIPFSTSGASVAYNVNSVADQFQRAFQTSTFCNRLYSFFNKRWFFDQVFNDFLVRSFL : 578
YP 006460149 : YFAKDMMIGLGTNFWANSPFVLPKNEILAESEFAAPTITKLIPIPFSTSGASVAYNVNSVADQFQRAFQTSTFCNRLYSFFNKRWFFDQVLNDFLVRSFL : 578
YP 006291784 : YFAKDMMIGLGTNFWANSPFVLPKNEILAESEFAAPTITKLIPIPFSTSGASVAYNVNPVADQFQRAFQTSTFCNRLYSFFNKRWFFDQVLNDFLVRSFL : 578
YP 005090406 : YFAKS-------HFWANSPFVLPKNEILAESEFATPTITKLIPIPFSTSGASVAYNVNSVADQFQRAFQTSTFCNRLYSFFNKRWFFDQVLNDFLVRSFL : 593
YP 004237247 : YLAKDMMIGLGTHFWANSPFVLPKNEILAESEFAAPTITKLIPIPFSTSGASVAYNVNPVADQFQQAFQTSTFCNRLYSFFNKRWFFDQVLNDFLVRSFL : 578
YP 173349    : YLAKDMMIGLGTNFWANSPFVLPKNEILAESEFAAPTITKLIPIPFSTSGAYVAYNVNPVADQFQRAFQTSTISNRLYSFFNKRWFFDQVLNDFLVRSFL : 578
               Y Ak        nFWANSPFVLPKNEILAESEFAaPTITKLIPIPFST GA VAYNVN VADQFQrAFQTST  NRLY3FFNKRWFFDQVlNDFLVRSFL
```

```
                       *       620       *      640       *      660        *      680       *
YP 009177610 : RFGYEVSFEALDKGAIEILGPYGISYTFRRLAERISQLQSGFVYHYAFAMLLGSTLFVTFSCMWDSLSSWVDNRSSFIL-IVSSFYNNKSSQE : 670
YP 009121953 : RFGYEVSFEALDKGAIEILGPYGISYTFRRLAERISQLQSGFVYHYAFAMLLGSTLFVTFSRMWDSLSSWVDNRSSFIW-IVSSFYNNKSSQ- : 669
YP 009049647 : RFGYEVSFEALDKGAIEILGPYGISYTFRRLAERISQLQSGFVYHYAFAMLLGSTLFVTFSRMWDSLSSWVDNRSSFIW-IVSSFYNNKSSQ- : 669
YP 009041162 : RFGYEVSFEALDKGAIEILGPYGISYTFRRLAERISQLQSGFVYHYAFAMLLGSTLFVTFSRMWDSLSSWVDNRSSFIW-IVSCFYNNKSSQE : 670
NAD5 SAMI     : RFGYEVSFEALDKGAIEILGPYGISYTFRRLAERISQLQSGFVYHYAFAMLLGSTLFVTFSRMWDSLSSWVDNRSSFIW-IVSRFYNNKSSQE : 662
YP 008964103 : RFGYEVSFEALDKGAIEILGPFGISYTFRRLAERISQLQSGFVYHYAFAMLLGSTLFVTFSRMWDSLSSWVDNRSSFIW-IVSRFYNNKSSQE : 670
YP 006460149 : RFGYEVSFEALDKGAIEILGPYGISYTFRRLVERISQLQSGFVYHYAFAMLLGSTLFLTFSRMWDSLSSWVDNRSFFIFLIVSYFYNNKSSQE : 671
YP 006291784 : RFGYEVSFEALDKGAIEILGPYGISYTFRRLAERISQLQSGFVYHYAFAMLLGSTLFVTFSRMWDSLSSWVDNRSSFIW-IVSSFYNNKSSQE : 670
YP 005090406 : RFGYEVSFEALDKGAIEILGPYGISYTFRRFAERISQLQSGFVYHYAFAMLLGSTLFVTFSRMWDSLSSWVDNRSSFIW-IVSRFYNKKSSQE : 685
YP 004237247 : RFGYEVSFEALDKGAIEILGPYGISYTFRRLAERISQLQSGFVYHYAFAMLLGSTLFVTFSRMWDSLSSWVDNRSSFIL-IVSTFSNNKSSQE : 670
YP 173349     : RFGYEVSFEALDKGAIEILGPYGISYTFRRLAERISQLQSGFVYHYAFAMLLGSTLFVTFSRMWDSLSSWVDNRSSFIW-IVSSFYNNKSSQ- : 669
               RFGYEVSFEALDKGAIEILGP5GISYTFRRlaERISQLQSGFVYHYAFAMLLGSTLF6TFSrMWDSLSSWVDNRSsFI  IVS FyNnKSSQ
```

Figure S3.24 The amino acid sequence allignment of NAD6 in mitogenome of *S. miltiorrhiza* and the corresponding homologs in other species

```
                        *        20         *        40         *        60         *        80         *       100
YP 009243675 : MILSVLSSPALVSGLMVARAKNPVHSVLFPIPVFRDTSGLLILLGLDFFAMIFPVVHIGAIAVSFLFVVMMFHIQIAEIHEEVLRYLPVSGIIGLIFWWE : 100
YP 009121978 : MILSVLSSPALVSGLMVVRAKNPVHSVLFLIPVFRNTSGLLLLLGLDFFAMIFPVVYIGAIAVSFLFVVMMFHIQIAEIHEEVLRYLPVSGIIGLIFWWE : 100
YP 009049789 : MILSVLSSPALVSGLMVVRAKNPVHSVLFLIPVFRNTSGLLLLLGLDFFAMIFPVVYIGAIAVSFLFVVMMFHIQIAEIHEEVLRYLPVSGIIGLIFWWE : 100
YP 009041169 : MILSVLSSPALVSGLMVVRAKNPVHSVLFPIPVFRNTSGLLLLLGLDFFAMIFPVVHIGAIAVLFLFVVMMFHIQIAEIHEEVLRYLPVSGIIGLIFWWE : 100
NAD6 SAMI     : MILSVLSSPALVSGLMVVRAKNPVHSVLFPIPVFRNTSGLLLFLGLDFFAMIFPVVHIGAIAVSFLFVVMMFNIQIAEIHEEVLRYLPVSGIIGLIFWWE : 100
YP 008964123 : MILSVLSSPALVSGLMVVRAKNPVHSVLFPIPVFRNTSGLLLFLGLDFFAMIFPVVHIGAIAVSFLFVVMMFNIQIAEIHEEVLRYLPVSGIIGLIFWWE : 100
YP 006460165 : MILSVLSSPALVSGLMVVRAKNPVHSVLFPIPVFRNTSGLLLFLGLDFFAMIFSVVHIGAIAVSFLFVVMMFNIQIAEIHEEVLRYLPVSGIIGLIFWWE : 100
YP 005090431 : MILSVLSSPALVSGFMVVRAKNPVHSVLFPIPVFCNTSGLLLFLGLDFFAMIFPVVHIGAIAVSFLFVVMMFNIQIAEIHEEVLRYLPVSGIIGLIFWWE : 100
YP 004935344 : MILSVLSSPALVSGLMVVRAKNPVHSVLFPIPVFRNTSGLLLLLGLDFSAMIFLVVYIGAIAVSFLFVVMMFHIQIAEIHEEVLRYLPVSGIIGLIFWWE : 100
YP 003587236 : MILSVLSSPALVSGLMVVRAKNPVHSVLFPIPVFRNTSGLLLLLGLDFFAMIFPVVHIGAIAVSFLFVVMMFHIQIAEIHEEVLRYLPVSGIIGLIFWWE : 100
YP 173471     : MILSVLSSPALVSGLMVVRAKNPVHSVLFLIPVFRNTSGLLLLLGLDFFAMIFPVVYIGAIAVSFLFVVMMFHIQIAEIHEEVLRYLPVSGIIGLLFWWE : 100
               MILSVLSSPALVSGlMVvRAKNPVHSVLF IPVFr1TSGLL6 LGLDFfAMIFpVV IGAIAVsFLFVVMMF IQIAEIHEEVLRYLPVSGIIGL6FWWE

                        *       120         *       140         *       160         *       180         *       200
YP 009243675 : MFFILDNETIPLLPTQRNTTSLRYTVYAGKVRSWTNLETLGNLLYTYYFVWFLVPSLILLVAMIGAIVLTMHRTTKVKRQDVFRRNAIDFRRTIMRRTTD : 200
YP 009121978 : MFFILDNESIPLLPTQRNTTSLRYTVYAGKVRSWTNLETLGNLLYTYYFVWFLVSSLILLVAMIGAIVLTMHRTTKVKRQDVFRRNALDSRRTIMRRTTD : 200
YP 009049789 : MFFILDNESIPLLPTQRNTTSLRYTVYAGKVRSWTNLETLGNLLYTYYFVWFLVSSLILLVAMIGAIVLTMHRTTKVKRQDVFRRNALDSRRTIMRRTTD : 200
YP 009041169 : MFFILDNESIPLLPTQRNTTSLRYTVYAGKVRSWTNLETLGNLLYTYYSVWFLVPSLILLVAMIGAIVLTMHRTTKVKRQDVFRRNAIDFRRTIMRRTTD : 200
NAD6 SAMI     : MFFILDNESIPLLPTQRNTTSLRYTVYAEKVRSWTNLETLGNLLYTYYFVWFLVPSLILLVAMIGAIVLTMHRTTKVKRQDVFRRNAIDSRRTIMRRTTD : 200
YP 008964123 : MFFILDNESIPLLPTQRNTTSLRYTVYAEKVRSWTNLETLGNLLYTYYFVWFLVPSLILLVAMIGAIVLTMHRTTKVKRQDVFRRNAIDSRRTIMRKEDD : 200
YP 006460165 : MFFILDNESIPLLPTQRNTTSLRYTVYAEKVRSWTNLETLGNLLYTYYFVWFLVPSLILLVAMIGAIVLTMHRTTKVKRQDVFRRNAIDSRRTIMRRTTD : 200
YP 005090431 : MFFILDNESIPLLPTQRNTTSLRYTVYAEKVRSWTNLETLGNLLYTYYSVWFLVPSLILLVAMIGAIVLTMHRTTKVKRQDVFRRNAIDSRRTIMRRTTD : 200
YP 004935344 : MFFILDNETIPLLPTQRNTTSLRYTVYAGKVRSWTNLETLGNLLYTYYFVWFLVSSLILLVAMIGAIVLTMHRTTKVKRQDVFRRNAIDSRRTIMRRTTD : 200
YP 003587236 : MFFILDNETIPLLPTQRNTTSLRYTVYAGKVRSWTNLETLGNLLYTYYSVWFLVSSLILLVAMIGAIVLTMHRTTKVKRQDVFRRNAIDFRRTIMRRTTD : 200
YP 173471     : MFFILDNESIPLLPTQRNTTSLRYTVYAGKVRSWTNLETLGNLLYTYYFVWFLVSSLILLVAMIGAIVLTMHRTTKVKRQDVFRRNALDSRRTIMRRTTD : 200
               MFFILDNE3IPLLPTQRNTTSLRYTVYA KVRSWTNLETLGNLLYTYY VWFLV SLILLVAMIGAIVLTMHRTTKVKRQDVFRRNA6D RRTIMR4ttD

                        *       220
YP 009243675 : -PLTIY------------------------ : 205
YP 009121978 : -PLTIH------------------------ : 205
YP 009049789 : -PLTTIRRSSGSNPHRELK----------- : 218
YP 009041169 : -PLTIY------------------------ : 205
NAD6 SAMI     : -PLTID------------------------ : 205
YP 008964123 : RPITIY------------------------ : 206
YP 006460165 : -PLTID------------------------ : 205
YP 005090431 : -PLTIY------------------------ : 205
YP 004935344 : -PLTIY------------------------ : 205
YP 003587236 : -PLTIY------------------------ : 205
YP 173471     : -PLTTIRRSSGSNPHRETRTVWRSIDKCY : 228
                P6Ti
```

Figure S3.25 The amino acid sequence allignment of NAD7 in mitogenome of *S. miltiorrhiza* and the corresponding homologs in other species

```
                         *        20         *        40         *        60         *        80         *        100
YP 009049635 : MTTKNRQIQNFTSNFGPQHPAAHGVSRSVLEMNGEVVERAEPHIGLLHRGTEKLIEYKTYLQALPYSDRSDYVSMMAQEHAHSSAVERLLNCEVPLRAQY : 100
YP 009045735 : MTTRNRQIKNFTSNFGPQHPAAHGVSRLVLEMNGEVVERAEPHIGSLHRGTEKLIEYKTYLQALPYSDRSDYVSMMAQEHAHSSAVEKLLNCEVPLRAQY : 100
YP 009041156 : MTTRNRQIKNFTSNFGPQHPAAHGVSRSVLEMNGEVVERAEPHIGSLHRGTEKLIEYKTYLQALPYSDRSDYVSMMAQEHAYSSAVERLLNCEVPLRAQY : 100
NAD7 SAMI     : MTTRNRQIKNFTLNFGPQHPAAHGVSRLVLEMNGEVVERAEPHIGSLHRGTEKLIEYKTYLQALPYSDRSDYVSMMAQEHAHSSAVERLLNCEVPLRAQY : 100
YP 008964099 : MTTRNRQIQNFTLNFGPQHPAAHGVSRLVLEMNGEVVERAEPHIGSLHRGTEKLIEYKTYLQALPYSDRSDYVSMMAQEHAHSSAVERLLNCEVPLRAQY : 100
YP 006666126 : MTTRNGQIKNFTSNSGPQHPAAHGVSRSVLEMNGEVVERAEPHIGSLHRGTEKLIEYKTYLQALPYSDRSDYVSMMAQEHAHSSAVERLLNCEVPLRAQY : 100
YP 006460175 : MTTRNRQIKNFTLNFGPQHPAAHGVSRLVLEMNGEVVERAEPHIGSLHRGTEKLIEYKTYLQALPYSDRSDYVSMMAQEHAHSSAVERLLNCEVPLRAQY : 100
YP 005090430 : MTTRNRQIKNFTLNFGPQHPAAHGVSRLVLEMNGEVVERAEPHIGSLQ--TEKLIEYKTYLQALPYSDRSEYVSMMAQEHAYSSAVERLLNCEVPLRAQY :  98
YP 004927561 : MTTRKRQIKNFTSNFGPQHPAAHGVSRLVLEMNGEVVERAEPHIGSLHRGTEKLIEYKTYLQALPYSDRSDYVSMMAQEHAHSSAVEKLLNCEVPLRAQY : 100
YP 004927501 : MTTRKRQIKNFTSNFGPQHPAAHGVSRLVLEMNGEVVERAEPHIGSLHRGTEKLIEYKTYLQALPYSDRSDYVSMMAQEHAHSSAVEKLLNCEVPLRAQY : 100
YP 173488     : MTTKNRQIKNFTSNFGPQHPAAHGVSRSVLEMNGEVVERAEPHIGLLHRGTEKLIEYKTYLQALPYSDRSDYVSMMAQEHAHSSAVERLLNCEVPLRAQY : 100
               MTT4nrQIkNFT NfGPQHPAAHGVSR VLEMNGEVVERAEPHIGsLhrgTEKLIEYKTYLQALPYSDRSdYVSMMAQEHAhSSAVE4LLNCEVPLRAQY

                         *       120         *       140         *       160         *       180         *       200
YP 009049635 : IRVLFREITRISNHSLALTTHAMDVGASTPFLWAFEEREKLLEFYERVSGARMHASFIRPGGVAQDLPLGLCIDIDSFTQQFASRIDELEEMSTGNRIWK : 200
YP 009045735 : IRVLFREITRISNHSLALTTHAMDVGASTPFLWAFEEREKLLEFYERVSGARMHASFIRPGGVAQDLPLGLCRDIDSFTQQFASRIDELEEMSTGNRIWK : 200
YP 009041156 : IRVLFREITRISNHSLALTTHAMDVGASTPFLWAFEEREKLLEFYERVSGARMHASFIRPGGVAQDLPLGLCRDIDSSTQQFASRIDELEEMSTGNRIWK : 200
NAD7 SAMI     : IRVLFREITRISNHSLALTTHAMDVGASTPFLWAFEEREKLLEFYERVSGARMHASFIRPGGVAQDLPLGLCRDIDSFTQQFSSRIDELEEMSTGNRIWK : 200
YP 008964099 : IRVLFREITRISNHSLALTTHAMDVGASTPFLWAFEEREKLLEFYERVSGARMHASFIRPGGVAQDLPLGLCRDIDSFTQQFSSRIDELEEMSTGNRIWK : 200
YP 006666126 : IRVLFREITRISNHSLALTTHAMDVGASTPFLWAFEEREKLLEFYERVSGARMHASFIRPGGVAQDLPLGLCRDIDSSTQQFASRIDELEEMSTGNRIWK : 200
YP 006460175 : IRVLFREITRISNHSLALTTHAMDVGASTPFLWAFEEREKLLEFYERVSGARMHASFIRPGGVAQDLPLGLCRDIDSFTQQFSSRIDELEEMSTGNRIWK : 200
YP 005090430 : IRVLFREITRISNHSLALTTHAMDVGASTPFLWAFEEREKLLEFYERVSGARMHASFIRPGGVAQDLPLGLCRDIDSFTQQFASRIDELEEMSTGNRIWK : 198
YP 004927561 : IRVLFREITRISNHSLALTTHAMDVGALTPFLWAFEEREKLLEFYERVSGARMHASFIRPGGVAQDLPLGLCRDIDSFTQQFASRIDELEEMSTGNRIWK : 200
YP 004927501 : IRVLFREITRISNHSLALTTHAMDVGALTPFLWAFEEREKLLEFYERVSGARMHASFIRPGGVAQDLPLGLCRDIDSFTQQFASRIDELEEMSTGNRIWK : 200
YP 173488     : IRVLFREITRISNHSLALTTHAMDVGASTPFLWAFEEREKLLEFYERVSGARMHASFIRPGGVAQDLPLGLCIDIDSFTQQFASRIDELEEMSTGNRIWK : 200
               IRVLFREITRISNHSLALTTHAMDVGAsTPFLWAFEEREKLLEFYERVSGARMHASFIRPGGVAQDLPLGLCrDIDSfTQQF SRIDELEEMSTGNRIWK

                         *       220         *       240         *       260         *       280         *       300
YP 009049635 : QRLVDIGTVTAQQAKDWGFSGVMLRGSGVCWDLRKAAPYDVHDQLDPDIPVGTRGDRYDRYCIRIEEMRQSVRIIVQCLNQMPSGMIKADDRKLCPPSRS : 300
YP 009045735 : QRLVDIGTVTAQQAKDWGFSGVMLRGPGVCWDSRRAAPYDVHDQSDPDVPVGTRGDRYDRYCIRIEEMRQSLRIIVQCLNQMPSGMIKADDRKLCPPSRC : 300
YP 009041156 : QRLVDIGTVTAQQAKDWGFSGVMLRGSGVCWDLRRAAPYDVHDQSDPDVPVGTRGDRYDRYCIRIEEMRQSVRIIVQCLNQMPSGMIKADDRKLCPPSRS : 300
NAD7 SAMI     : QRLVDIGTVTAQQAKDWGFSGVMLRGSGVCWDLRRAAPYDVHDQLDPDVPVGTRGDRYDRYCIRIEEMRQSVRIIVQCLNQMPSGMIKADDRKLCPPSRC : 300
YP 008964099 : QRLVDIGTVTAQQAKDWGFSGVMLRGSGVCWDLRRAAPYDVHDQLDPDVPVGTRGDRYDRYCIRIEEMRQSVRIIVQCLNKMPSGMIKADDRKLCPPSRC : 300
YP 006666126 : QRLVDIGTVTAQQAKDWGFSGVMLRGPGVCWDSRRAAPYDVHDQSDPDVPVGTRGDRYDRYCIRIEEMRQSVRIIVQCLNQMPSGMIKADDRKLCPPSRC : 300
YP 006460175 : QRLVDIGTVTAQQAKDWGFSGVMLRGSGVCWDLRRAAPYDVHDQLDPDVPVGTRGDRYDRYCIRIEEMRQSVRIIVQCLNQMPSGMIKADDRKLCPPSRC : 300
YP 005090430 : QRLVDIGTVTAQQAKDWGFSGVMLRGSGVCWDLRRAAPYDVHDQLDPDVPVGTRGDRYDRYCIRIEEMRQSVRIIVQCLNKMPSGMIKADDRKLCPPSRC : 298
YP 004927561 : QRLVDIGTVTAQQAKDWGFSGVMLRGPGVCWDSRRAAPYDVHDQSDPDVPVGTRGDRYDRYCIRIEEMRQSLRIIVQCLNQMPSGMIKADDRKLCPPSRC : 300
YP 004927501 : QRLVDIGTVTAQQAKDWGFSGVMLRGPGVCWDSRRAAPYDVHDQSDPDVPVGTRGDRYDRYCIRIEEMRQSLRIIVQCLNQMPSGMIKADDRKLCPPSRC : 300
YP 173488     : QRLVDIGTVTAQQAKDWGFSGVMLRGSGVCWDLRKAAPYDVHDQLDPDIPVGTRGDRYDRYCIRIEEMRQSVRIIVQCLNQMPSGMIKADDRKLCPPSRS : 300
               QRLVDIGTVTAQQAKDWGFSGVMLRG GVCWD R4AAPYDVHDQ DPD6PVGTRGDRYDRYCIRIEEMRQS6RIIVQCLNqMPSGMIKADDRKLCPPSR
```

```
                        *       320         *       340         *       360         *       380         *
YP 009049635 : RMKLSMESSIHHFEPYTEGFSVPAPSTYTAVEAPKGEFGVFLVSNGSNRPYRRKIRAPGFAHSQGLDSMSKHHMPADVVTIIGTQDIVSGEVDR : 394
YP 009045735 : RMKLSMESSIHHFELYTEGFSVPASSTYTAVEAPKGEFGVFLVSNGSNRPYRRKIRAPGSAHSQGLDSMSKHHMPADVVTIIGTQDIVFGEVDR : 394
YP 009041156 : RMKLSMESSIHHFEPYTEGFSVPASSTYTAVEAPKGEFGVFLVSNGSNRPYRRKIRAPGFAHLQGLDSMSKHHMPADVVTIIGTQDIVSGEVDR : 394
NAD7 SAMI     : RMKLSMESSIHHFEPYTEGFSVPASSTYTAVEAPKGEFGVFLVSNGSNRPYRRKIRAPGFAHLQGLDSMSKHHMPADVVTIIGTQDIVSGEVDR : 394
YP 008964099 : RMKLSMESLIHHFEPYTEGFSVPASSTYTAVEAPKGEFGVFLVSNGSNRPYRRKIRAPGFAHSQGLDSMSKHHMPADVVTIIGTQDIVSGEVDR : 394
YP 006666126 : RMKLSMESSIHHFEPYTEGFSVPASSTYTAVEAPKGEFGVFLVSNGSNRPYRRKIRAPGSAHLQGLDSMSKHHMPADVVTIIGTQDIVSGEVDR : 394
YP 006460175 : RMKLSMESSIHHFEPYTEGFSVPASSTYTAVEAPKGEFGVFLVSNGSNRPYRRKIRAPGFAHSQGLDSMSKHHMPADVVTIIGTQDIVSGEVDR : 394
YP 005090430 : RMKLSMESSIHHFEPYTEGFSVPASSTYTAVEAPKGEFGVFLVSNGSNRPYRRKIRAPGFAHSQGLDSMSKHHMPADVVTIIGTQDIVSGEVDR : 392
YP 004927561 : RMKLSMESSIHHFELYTEGFSVPASSTYTAVEAPKGEFGVFLVSNGSNRPYRRKIRAPGSAHSQGLDSMSKHHMPADVVTIIGTQDIVFGEVDR : 394
YP 004927501 : RMKLSMESSIHHFELYTEGFSVPASSTYTAVEAPKGEFGVFLVSNGSNRPYRRKIRAPGFAHSQGLDSMSKHHMPADVVTIIGTQDIVFGEVDR : 394
YP 173488     : RMKLSMESSIHHFEPYTEGFSVPAPSTYTAVEAPKGEFGVFLVSNGSNRPYRRKIRAPGFAHSQGLDSMSKHHMPADVVTIIGTQDIVSGEVDR : 394
               RMKLSMESsIHHFE YTEGFSVPAsSTYTAVEAPKGEFGVFLVSNGSNRPYRRKIRAPG AH QGLDSMSKHHMPADVVTIIGTQDIV GEVDR
```

Figure S3.26 The amino acid sequence allignment of NAD9 in mitogenome of *S. miltiorrhiza* and the corresponding homologs in other species

```
                            *        20         *        40         *        60         *        80         *       100
YP 009243650 : MDNQFIFKYSWETFPKKWVKKMERSEHGNRSDTNTDYPFQLLCFLKLHTYTRVQVSIDICGVDYPSRKRRFEVIYNLLSTRYNSRIRVQTSADEVTRISP : 100
YP 009241668 : MDNQFIFKYSWETFPKKWVKKMERSEHGNRSDTNTDYPFQLLCFLKLHTYTRVQVSIDICGVDYPSRKRRFEVVYNLLSTRYNSRIRVQTSADEVTRISP : 100
YP 009153947 : MDNQFIFKYSWETFPKKWVKKMERSEHGNRSDTNTDYLFQLLCFLKLHTYTRVQVSIDICGVDHPSRKRRFEVVYNLLSTRYNSRIRVQTSADEVTRISP : 100
YP 009041188 : MDNQFIFKYSWETLPKKWVKKMERSEHGNRSDTNTDYLFQLLCFLKLHTYTRVQVSIDICGVDYPSRKRRFEVVYNLLSTRYNSRIRVQTCADEVTRISP : 100
NAD9 SAMI     : MDNQFIFKYSWEILPKKWVKKMERSEHGNRSDTNTDYPFKLLCFLKLHTYTRVQVSIDICGIDYPSRKRRFEVVYNLLSTRYNSRIRVQTSADEVTRISP : 100
YP 006666131 : MDNQFIFKYSWETLPKKWVKKMERSEHGNRSDTNTDYPFQLLCFLKLHTYTRVQVSIDICGVDYPSRKRRFEVVYNLLSTRYNSRIRVQTSADEVTRISP : 100
YP 006460169 : MDNQFIFKYSWEILPKKWVKKMERSEHGNRSDTNTDYPFQLLCFLKLHTYTRVQVSIDICGVDYPSRKRRFEVVYNLLSTRYNSRIRVQTSADEVTRISP : 100
YP 005090422 : MDNQFIFKYSWEILPKRWVKKMERSEHGNRSDTNTDYPFQLLCFLKLHTYTRVQVSIDICGVDYPSRKRRFEVVYNLLSTRYNSRIRVQTSADEVTRISP : 100
YP 004222820 : MDNQSIFKYSWETLPKKWVKKMERSEHGNRSDTKTDYLFQLLCFLKLHTYTRVQVSIDICGVDYPSRKRRFEVVYNLLSTRYNSRIRVQTSADEVTRISP : 100
YP 173479    : MDNQFIFKYSWETLPKKWVKKMERSEHGNRSDTNTDYLFQLLCFLKLHTYTRVQVSIDICGVDYPSRKQRFEVVYNLLSIRYNSRIRVQTSADEVTRISP : 100
NP 085479    : MDNQFIFKYSWETLPKKWVKKMERSEHGNRSDTNTDYLFQLLCFLKLHTYTRVQVSIDICGVDHPSRKRRFEVVYNLLSTRYNSRIRVQTSADEVTRISP : 100
               MDNQfIFKYSWE  PK4WVKKMERSEHGNRSDTnTDY FqLLCFLKLHTYTRVQVSIDICG6DyPSRKrRFEV6YNLLStRYNSRIRVQTsADEVTRISP

                            *       120         *       140         *       160         *       180         *
YP 009243650 : VVSLFPSAGRWEREVWDMFGVSSINHPDLRRISTDYGFEGHPLRKDLPLSGYVEVRYDDPEKRVVSEPIEMTQEFRYFDFASPWEQHSDG : 190
YP 009241668 : VVSLFPSAGWWEREVWDMFGVSSINHPDLRRISTDYGFEGHPLRKDLPLSGYVEVRYDDPEKRVVSEPIEMTQEFRYFDFASPWEQRSDG : 190
YP 009153947 : VVSIFPSAGRWEREVWDMFGVSSINHPDLRRISTDYGFEGHPLRKDLPLSGYVEVRYDDPEKRVVSEPIEMTQEFRYFDFASPWEQRSDG : 190
YP 009041188 : VVSLFPSAGWWEREVWDMFGVSSINHPDLRRISTDYGFEGHPLRKDLPLSGYVEVRYDDPEKRVVSEPIEMTQEFRYFDFASPWEQRSDG : 190
NAD9 SAMI     : VVSLFPSAGRWEREVWDMFGVSSINHPDLRRISTDYGFEGHPLRKDLPLSGYVEVRYDDPEKRVVSEPIEMTQEFRYFDFASPWEQRSDG : 190
YP 006666131 : VVSLFPSAGRWEREVWDMFGVSSINHPDLRRISTDYGFEGHPLRKDLPLSGYVEVRYDDPEKRVVSEPIEMTQEFRYFDFASPWEQRSDG : 190
YP 006460169 : VVSPFPSAGRWEREVWDMFGVSSINHPDLRRISTDYGFEGHPLRKDLPLSGYVEVRYDDPEKRVVSEPIEMTQEFRYFDFASPWEQRSDG : 190
YP 005090422 : VVSPFPSAGRWEREVWDMFGVSSINHPDLRRISTDYGFEGHPLRKDLPLSGYVEVRYDDPEKRVVSEPIEMTQEFRYFDFASPWEQRSDG : 190
YP 004222820 : VVSLFPSAGRWEREVWDMFGVSSINHPDLRRISTDYGFEGHPLRKDLPLSGYVEVRYDDPEKRVVSEPIEMTQEFRYFDFASPWEQRSDG : 190
YP 173479    : VVSLFPSAGRWEREVWDMFGVSSINHPDLRRISTDYGFEGHPLRKDLPLSGYVEVRYDDPEKRVVSEPIEMTQEFRYFDFASPWEQRSDG : 190
NP 085479    : VVSLFPSAGRWEREVWDMFGVSFINHPDLRRISTDYGFEGHPLRKDLPLSGYVQVRYDDPEKRVVSEPIEMTQEFRYFDFASPWEQRSDG : 190
               VVS FPSAGrWEREVWDMFGVSsINHPDLRRISTDYGFEGHPLRKDLPLSGYV2VRYDDPEKRVVSEPIEMTQEFRYFDFASPWEQrSDG
```

Figure S3.27 The amino acid sequence allignment of PETG in mitogenome of *S. miltiorrhiz*a and the corresponding homologs in other species

```
                        *        20         *        40         *        60         *        80         *
YP 009170086 : ------------------------------------------------------MIEVFLFGIVLGLIPITLAGLFVTAYLQYRRGDQLDFD : 38
YP 009053971 : ------------------------------------------------------MIEVFLFGIVLGLIPITVAGLFVTAYLQYRRGDQLDL- : 37
YP 009024812 : ------------------------------------------------------MIEVFLFGIVLGLIPVTLAGLFVTAYLQYRRGDQLDL- : 37
YP 005090197 : ------------------------------------------------------MIEVFLFGIVLGLIPITLAGLFITAYLQYRRGDQLDL- : 37
YP 004465206 : ------------------------------------------------------MIEVFLFGIVLGLIPITLSGLFVTAYLQYRRGDQLDL- : 37
YP 003934322 : ------------------------------------------------------MIEVFLFGIVLGLVPITLAGLFVTAYLQYRRGDQLDL- : 37
YP 002456442 : ------------------------------------------------------MIEVFLFGIVLGLIPITLAGLFVTAYLQYRRGDQLDI- : 37
YP 740584     : ------------------------------------------------------MVEVFLFGIVLGLIPITLAGLFVTAYLQYRRGDQLDL- : 37
YP 654227     : MNKAEKKKSLFWIPGILRTNYQFFFSWSLRFVDNLDYYLGINRTSFFYPLEQIEMIEVFLFGIVLGLIPITLAGLFVTAYLQYRRGDQLDL- : 91
NP 054520     : ------------------------------------------------------MIEVFLFGIVLGLIPITLAGLFVTAYLQYRRGDQLDL- : 37
PETG SAMI     : ------------------------------------------------------MIEVFLFGIVLGLIPITLAGLFVTAYLQYRRGDQLDL- : 37
                                                                     M6EVFLFGIVLGL6P6T6aGLF6TAYLQYRRGDQLD
```

Figure S3.28 The amino acid sequence allignment of PETL in mitogenome of *S. miltiorrhiza* and the corresponding homologs in other species

```
                              *        20        *
YP 009183610 : MLTITSYFGFLLVALTITSAIFIGLSKIRLI : 31
YP 009171171 : MLTITSYFGFLLVALTITSALFIGLSKIQLI : 31
YP 009166707 : MLTVTSYFGFLLVALTITSALFIGLSKIRLI : 31
YP 009002273 : MLTITSYFGFLLVALTITSALFISLSKIRLI : 31
YP 008592506 : MLTITSYFGFLLVALTITSVLFIGLSKIRLI : 31
YP 784083    : MLTITSYFGFLLVALTITSALFIGLSKIRLI : 31
YP 778507    : MLTITSYFGFLLVVLTITSALFIGLSKIRLI : 31
YP 398347    : MLTITSYFGFLLTALTITSALFIGLSKIRLI : 31
YP 086984    : MPTITSYFGFLLVALTITSALFIGLSKIRLI : 31
NP 054519    : MLTITSYFGFLLAALTITSALFIGLSKIRLI : 31
PETL SAMI    : MLTITSYFGFLLVALTITSALFIGLSKIRLI : 31
               MlT6TSYFGFLLvaLTITSa6FIgLSKIrLI
```

Figure S3.29 The amino acid sequence allignment of RBCL in mitogenome of *S. miltiorrhiza* and the corresponding homologs in other species

```
                         *        20         *        40         *        60         *        80         *       100
YP 007507119 : -------MSPQTETKASVGFKAGVKEYKLTYYTPEYETKDTDILAAFRVTPQPGVPPEEAGAAVAAESSTGTWTTVWTDGLTSLDRYKGRCYHIEPVPGE :  93
YP 009144523 : -------MSPQTETKASVGFKAGVKEYKLTYYTPEYETKDTDILAAFRVTPQPGVPPEEAGAAVAAESSTGTWTTVWTDGLTSLDRYKGRCYHIEPVPGE :  93
YP 009232050 : -------MSPQTETKAGVGFKAGVKEYKLTYYTPEYETKDTDILAAFRVTPQPGVPPEEAGAAVAAESSTGTWTTVWTDGLTSLDRYKGRCYHIEPVPGE :  93
YP 009270920 : MSCREGFMSPQTETKASVGFKAGVKEYKLTYYTPEYETKDTDILAAFRVTPQPGVPPEEAGAAVAAESSTGTWTTVWTDGLTSLDRYKGRCYHIEPVIGE : 100
YP 009242775 : -------MSPQTETKASVGFKAGVKEYKLTYYTPEYETKDTDILAAFRVTPQPGVPPEEAGAAVAAESSTGTWTTVWTDGLTSLDRYKGRCYHIEPVLGE :  93
YP 009183600 : -------MSPQTETKASVGFKAGVKEYKLTYYTPEYETKDTDILAAFRVTPQPGVPPEEAGAAVAAESSTGTWTTVWTDGLTSLDRYKGRCYHIEPVIGE :  93
YP 007353923 : MSCREGFMSPQTETKASVGFKAGVKEYKLTYYTPEYETKDTDILAAFRVTPQPGVPPEEAGAAVAAESSTGTWTTVWTDGLTSLDRYKGRCYHIEPVPGE : 100
YP 008964042 : -------MSPQTETKASVGFKAGVKEYKLTYYTPEYETKDTDILAAFRVTPQPGVPPEEAGAAVAAESSTGTWTTVWTDGLTSLDRYKGRCYHIEPVLGE :  93
YP 009239204 : -------MSPQTETKASVGFKAGVKEYKLTYYTPEYETKDTDILAAFRVTPQPGVPPEEAGAAVAAESSTGTWTTVWTDGLTSLDRYKGRCYHIEPVPGE :  93
YP 009294871 : -------MSPQTETKASVGFKAGVKEYKLTYYTPEYETKDTDILAAFRVTPQPGVPPEEAGAAVAAESSTGTWTTVWTDGLTSLDRYKGRCYHIEPVPGE :  93
RBCL SAMI    : -------MSPQTETKASVGFKAGVKEYKLTYYTPEYETKDTDILAAFRVTPQPGVPPEEAGAAVAAESSTGTWTTVWTDGLTSLDRYKGRCYHIEPVPGE :  93
                      MSPQTETKAsVGFKAGVKEYKLTYYTPEYETKDTDILAAFRVTPQPGVPPEEAGAAVAAESSTGTWTTVWTDGLTSLDRYKGRCYHIEPV GE

                         *       120         *       140         *       160         *       180         *       200
YP 007507119 : KDQYICYVAYPLDLFEEGSVTNMFTSIVGNVFGFKALRALRLEDLRIPVAYVKTFQGPPHGIQVERDKLNKYGRPLLGCTIKPKLGLSAKNYGRAVYECL : 193
YP 009144523 : KDQYICYVAYPLDLFEEGSVTNMFTSIVGNVFGFKALRALRLEDLRIPVAYVKTFQGPPHGIQVERDKLNKYGRPLLGCTIKPKLGLSAKNYGRAVYECL : 193
YP 009232050 : KDQYICYVAYPLDLFEEGSVTNMFTSIVGNVFGFKALRALRLEDLRIPVAYVKTFQGPPHGIQAERDKLNKYGRPLLGCTIKPKLGLSAKNYGRAVYECL : 193
YP 009270920 : KDQYICYVAYPLDLFEEGSVTNMFTSIVGNVFGFKALRALRLEDLRIPTAYIKTFQGPPHGIQVERDKLNKYGRPLLGCTIKPKLGLSAKNYGRAVYECL : 200
YP 009242775 : KDQYICYVAYPLDLFEEGSVTNMFTSIVGNVFGFKALRALRLEDLRVPTAYIKTFQGPPHGIQVERDKLNKYGRPLLGCTIKPKLGLSAKNYGRAVYECL : 193
YP 009183600 : KDQYICYVAYPLDLFEEGSVTNMFTSIVGNVFGFKALRALRLEDLRIPVAYVKTFQGPPHGIQVERDKLNKYGRPLLGCTIKPKLGLSAKNYGRAVYECL : 193
YP 007353923 : KDQYICYVAYPLDLFEEGSVTNMFTSIVGNVFGFKALRALRLEDLRIPTAYIKTFQGPPHGIQVERDKLNKYGRPLLGCTIKPKLGLSAKNYGRAVYECL : 200
YP 008964042 : KDQYICYVAYPLDLFEEGSVTNMFTSIVGNVFGFKALRALRLEDLRIPVAYIKTFQGPPHGIQVERDKLNKYGRPLLGCTIKPKLGLSAKNYGRAVYECL : 193
YP 009239204 : TDQYICYVAYPLDLFEEGSVTNMFTSIVGNVFGFKALRALRLEDLRIPPAYVKTFQGPPHGIQVERDKLNKYGRPLLGCTIKPKLGLSAKNYGRAVYECL : 193
YP 009294871 : ADQYICYVAYPLDLFEEGSVTNMFTSIVGNVFGFKALRALRLEDLRIPVAYVKTFQGPPHGIQVERDKLNKYGRPLLGCTIKPKLGLSAKNYGRACYECL : 193
RBCL SAMI    : KDQYICYVAYPLDLFEEGSVTNMFTSIVGNVFGFKALRALRLEDLRIPVAYVKTFQGPPHGIQVERDKLNKYGRPLLGCTIKPKLGLSAKNYGRAVYECL : 193
               kDQYICYVAYPLDLFEEGSVTNMFTSIVGNVFGFKALRALRLEDLR6P AY6KTFQGPPHGIQvERDKLNKYGRPLLGCTIKPKLGLSAKNYGRAvYECL

                         *       220         *       240         *       260         *       280         *       300
YP 007507119 : RGGLDFTKDDENVNSQPFMRWRDRFLFCAEAIYKAQTETGEIKGHYLNATAGTCEEMMKRAIFARELGVPIVMHDYLTGGFTANTTLAHYCRDNGLLLHI : 293
YP 009144523 : RGGLDFTKDDENVNSQPFMRWRDRFLFCAEAIYKAQTETGEIKGHYLNATAGTCEEMMKRAIFARELGVPIVMHDYLTGGFTANTTLAHYCRDNGLLLHI : 293
YP 009232050 : RGGLDFTKDDENVNSQPFMRWRDRFLFCAEAIYKSQAETGEIKGHYLNATAGTCEEMMKRAIFARELGVPIVMHDYLTGGFTANTSLAHYCRDNGLLLHI : 293
YP 009270920 : RGGLDFTKDDENVNSQPFMRWRDRFLFCAEAIYKAQAETGEIKGHYLNATAGTCEEMMKRAIFARELGVPIVMHDYLTGGFTANTSLAHYCRDNGLLLHI : 300
YP 009242775 : RGGLDFTKDDENVNSQPFMRWRDRFLFCAEAIYKAQAETGEIKGHYLNATAGTCEEMMKRAVFARELGVPIVMHDYLTGGFTANTSLAHYCRDNGLLLHI : 293
YP 009183600 : RGGLDFTKDDENVNSQPFMRWRDRFLFCAEAIYKAQAETGEIKGHYLNATAGTCEEMMKRAVFARELGVPIVMHDYLTGGFTANTSLAHYCRDNGLLLHI : 293
YP 007353923 : RGGLDFTKDDENVNSQPFMRWRDRFLFCAEAIYKAQAETGEIKGHYLNATAGTCEEMMKRAVFARELGVPIVMHDYLTGGFTANTSLAHYCRDNGLLLHI : 300
YP 008964042 : RGGLDFTKDDENVNSQPFMRWRDRFLFCAEAIYKAQAETGEIKGHYLNATAGTCEEMIKRAVFARELGVPIVMHDYLTGGFTANTTLAHYCRDNGLLLHI : 293
YP 009239204 : RGGLDFTKDDENVNSQPFMRWRDRFLFCAEALYKAQAETGEIKGHYLNATAGTCEEMIKRAVFARELGVPIVMHDYLTGGFTANTSLAHYCRDNGLLLHI : 293
YP 009294871 : RGGLDFTKDDENVNSQPFMRWRDRFLFCAEAIYKSQAETGEIKGHYLNATAGTCEEMMKRAIFARELGVPIVMHDYLTGGFTANTSLAHYCRDNGLLLHI : 293
RBCL SAMI    : RGGLDFTKDDENVNSQPFMRWRDRFLFCAEAIYKAQTETGEIKGHYLNATAGTCEEMMKRAIFARELGVPIVMHDYLTGGFTANTTLAHYCRDNGLLLHI : 293
               RGGLDFTKDDENVNSQPFMRWRDRFLFCAEA6YKaQ ETGEIKGHYLNATAGTCEEM6KRA6FARELGVPIVMHDYLTGGFTANT3LAHYCRDNGLLLHI
```

```
                   *        320         *        340         *        360         *        380         *        400
YP 007507119 : HRAMHAVIDRQKNHGMHFRVLAKALRLSGGDHIHAGTVVGKLEGERDITLGFVDLLRDDFVEKDRSRGIYFTQDWVSLPGVIPVASGGIHVWHMPALTEI : 393
YP 009144523 : HRAMHAVIDRQKNHGMHFRVLAKALRLSGGDHIHAGTVVGKLEGERDITLGFVDLLRDDFVEKDRSRGIYFTQDWVSLPGVIPVASGGIHVWHMPALTEI : 393
YP 009232050 : HRAMHAVIDRQKNHGMHFRVLAKALRLSGGDHVHSGTVVGKLEGERDITLGFVDLLRDDFVEKDRSRGIYFTQDWVSLPGVIPVASGGIHVWHMPALTEI : 393
YP 009270920 : HRAMHAVIDRQKNHGMHFRVLAKALRLSGGDHIHAGTVVGKLEGERDITLGFVDLLRDDFVEKDRSRGIYFTQDWVSLPGVIPVASGGIHVWHMPALTEI : 400
YP 009242775 : HRAMHAVIDRQKNHGMHFRVLAKALRLSGGDHIHAGTVVGKLEGEREITLGFVDLLRDDFIEKDRSRGIYFTQDWVSLPGVIPVASGGIHVWHMPALTEI : 393
YP 009183600 : HRAMHAVIDRQKNHGMHFRVLAKALRMSGGDHIHAGTVVGKLEGERDITLGFVDLLRDDFIEKDRSRGIYFTQDWVSLPGVIPVASGGIHVWHMPALTEI : 393
YP 007353923 : HRAMHAVIDRQKNHGMHFRVLAKALRLSGGDHIHAGTVVGKLEGEREITLGFVDLLRDDFIEKDRSRGIYFTQDWVSLPGVIPVASGGIHVWHMPALTEI : 400
YP 008964042 : HRAMHAVIDRQKNHGMHFRVLAKALRLSGGDHIHAGTVVGKLEGEREITLGFVDLLRDDFVEKDRSRGIYFTQDWVSLPGVIPVASGGIHVWHMPALTEI : 393
YP 009239204 : HRAMHAVIDRQKNHGIHFRVLAKALRMSGGDHIHSGTVVGKLEGERDITLGFVDLLRDDFIEKDRSRGIYFTQDWVSLPGVIPVASGGIHVWHMPALTEI : 393
YP 009294871 : HRAMHAVIDRQKNHGIHFRVLAKALRMSGGDHIHSGTVVGKLEGERDITLGFVDLLRDDFIEKDRSRGIYFTQDWVSLPGVIPVASGGIHVWHMPALTEI : 393
RBCL SAMI    : HRAMHAVIDRQKNHGMHFRVLAKALRLSGGDHIHAGTVVGKLEGERDITLGFVDLLRDDFVEKDRSRGIYFTQDWVSLPGVIPVASGGIHVWHMPALTEI : 393
               HRAMHAVIDRQKNHG6HFRVLAKALR6SGGDH6H GTVVGKLEGER ITLGFVDLLRDDF6EKDRSRGIYFTQDWVSLPGVIPVASGGIHVWHMPALTEI

                   *        420         *        440         *        460         *        480
YP 007507119 : FGDDSVLQFGGGTLGHPWGNAPGAVANRVAVEACVQARNEGRDLAAEGNAIIREACKWSPELAAACEVWKEIKFEFKAVDTLDK----- : 477
YP 009144523 : FGDDSVLQFGGGTLGHPWGNAPGAVANRVAVEACVQARNEGRDLAAEGNAIIREACKWSPELAAACEVWKEIKFEFKAVDTLDK----- : 477
YP 009232050 : FGDDSVLQFGGGTLGHPWGNAPGAVANRVAVEACVLARNEGRDLAAEGNAIIREACKWSPELAAACEVWKEIKFEFKAVDTLDK----- : 477
YP 009270920 : FGDDSVLQFGGGTLGHPWGNAPGAVANRVALEACVQARNEGRDLATEGNAIIREACKWSPELAAACEVWKEIKFEFKAVDVLDPEK--- : 486
YP 009242775 : FGDDSVLQFGGGTLGHPWGNAPGAVANRVALEACVQARNEGRDLAAEGNAIIREACKWSPELAAACEVWKEIKFEFKPVDTLDEEKKN- : 481
YP 009183600 : FGDDSVLQFGGGTLGHPWGNAPGAVANRVAVEACVQARNEGRDLAAEGNAIIREASKWSPELAAACEVWKEIKFEFAAMDTLDPTK--- : 479
YP 007353923 : FGDDSVLQFGGGTLGHPWGNAPGAVANRVALEACVQARNEGRDLAAEGNAIIREASKWSPELAAACEVWKEIRFEFKPVDTLDQ----- : 484
YP 008964042 : FGDDSVLQFGGGTLGHPWGNAPGAVANRVALEACVQARNEGRDLAAEGNTIIREACKWSPELAAACEVWKEIKFEFQPVDTLDPSKEDK : 482
YP 009239204 : FGDDSVLQFGGGTLGHPWGNAPGAVANRVALEACVQARNEGRDLAAEGNAIIREACKWSPELAAACEVWKEIKFEFTAVDTLDK----- : 477
YP 009294871 : FGDDSVLQFGGGTLGHPWGNAPGAVANRVALEACVQARNEGRDLASEGNAIIREACKWSPELAAACEVWKEIKFEFEAMDKLDK----- : 477
RBCL SAMI    : FGDDSVLQFGGGTLGHPWGNAPGAVANRVAVEACVQARNEGRDLAAEGNAIIREACKWSPELAAACEVWKEIKFEFKAVDTLDK----- : 477
               FGDDSVLQFGGGTLGHPWGNAPGAVANRVA6EACVqARNEGRDLAaEGNaIIREAcKWSPELAAACEVWKEI4FEF  6DtLD
```

Figure S3.30 The amino acid sequence allignment of RPL2 in mitogenome of *S. miltiorrhiza* and the corresponding homologs in other species.

```
                         *        20         *        40         *        60         *        80         *       100
YP 009242721 : MAIHLYKTSTPSTRNGTVGSQVKSNPRNNLIYGQHHCGKGRNARGIITARHRGGGHKRLYRKIDFRRNEKDIYGRIVTIEYDPNRNAYICLIHYGDGEKR : 100
YP 009242105 : MAIHLYKTSTPSTRNGTVGSQVKSNPRNNLIYGQHHCGKGRNARGIITARHRGGGHKRLYRKIDFRRNEKDIYGRIVTIEYDPNRNAYICLIHYGDGEKR : 100
YP 009232109 : MAIHLYKTSTPSTRNGTVDSQVKSNPRNNLIYGQHHCGKGRNARGIITARHRGGGHKRLYRKIDFRRNEKDIYGRIVTIEYDPNRNAYICLIHYGDGEKR : 100
YP 009002296 : MAIHLYKTSTPSTRNGTVDSQVKSNPRNNLIYGQHHCGKGRNARGIITARHRGGGHKRLYRKIDFRRNEKDIYGRIVTIEYDPNRNAYICLIHYGDGEKR : 100
RPL2 SAMI    : MAIHLYKTSTPSTRNGTVDSQVKSNPRNNLIYGQHHCGKGRNARGIITARHRGGGHKRLYRKIDFRRNEKDIYGRIVTIEYDPNRNAYICLIHYGDGEKR : 100
YP 008964096 : MAIHLYKTSTPSTRNGTVDSQVKSNPRNNLIYGQHHCGKGRNARGIITARHRGGGHKRLYRKIDFRRNEKDIYGRIVTIEYDPNRNAYICLIHYGDGEKR : 100
YP 008816000 : MAIHLYKTSTPSTRNGTVDSQVKSNPRNNLIYGQHHCGKGRNARGIITARHRGGGHKRLYRKIDFRRNEKDIYGRIVTIEYDPNRNAYICLIHYGDGEKR : 100
YP 007507153 : MAIHLYKTSTPSTRNGTVDSQVKSNPRNNLIYGQHHCGKGRNARGIITARHRGGGHKRLYRKIDFRRNEKDIYGRIVTIEYDPNRNAYICLIHYGDGEKR : 100
YP 004935708 : MAIHLYKTSTPSTRNGTVDSQVKSNPRNNLIYGQHHCGKGRNARGIITARHRGGGHKRLYRKIDFRRNEKDIYGRIVTIEHDPNRNAYICLIHYGDGEKR : 100
YP 004376461 : MAIHLYKTSTPSTRNGTVDSQVKSNPRNNLIYGQHHCGKGRNARGIITARHRGGGHKRLYRKIDFRRNEKDIYGRIVTIEYDPNRNAYICLIHYGDGEKR : 100
NP 054540    : MAIHLYKTSTPSTRNGTVDSQVKSNPRNNLIYGQHHCGKGRNARGIITARHRGGGHKRLYRKIDFRRNEKDIYGRIVTIEYDPNRNAYICLIHYGDGEKR : 100
               MAIHLYKTSTPSTRNGTVdSQVKSNPRNNLIYGQHHCGKGRNARGIITARHRGGGHKRLYRKIDFRRNEKDIYGRIVTIEyDPNRNAYICLIHYGDGEKR

                         *       120         *       140         *       160         *       180         *       200
YP 009242721 : YILHPRGAIIGDTIVSGTEVPIKMGNALPLTDMPLGTAIHNIEITLGKGGQLVRAAGAVAKLIAKEGKSATLKLPSGEVRLISKNCSATVGQVGNVGANQ : 200
YP 009242105 : YILHPRGAIIGDTIVSGTEVPIKMGNALPLTDMPLGTAIHNIEITLGKGGQLVRAAGAVAKLIAKEGKSATLKLPSGEVRLISKNCSATVGQVGNVGANQ : 200
YP 009232109 : YILHPRGAIIGDTIVSGTEVPIKMGNALPLTDMPLGTAIHNIEITLGKGGQLVRAAGAVAKLIAKEGKSATLKLPSGEVRLISKNCSATVGQVGNVGANQ : 200
YP 009002296 : YILHPRGAIIGDTIVSGTEVPIKMGNALPLTDMPLGTAIHNIEITLGKGGQLVRAAGAVAKLIAKEGKSATLKLPSGEVRLISKNCSATVGQVGNVGANQ : 200
RPL2 SAMI    : YILHPRGAIIGDTIVSGTEVPIKMGNALPLTDMPLGTAIHNIEITLGKGGQLVRAAGAVAKLIAKEGKSATLKLPSGEVRLISKNCSATVGQVGNVGANQ : 200
YP 008964096 : YILHPRGAIIGDTIVSGTEVPIKMGNALPLTDMPLGTAIHNIEITLGKGGQLVRAAGAVAKLIAKEGKSATLKLPSGEVRLISKNCSATVGQVGNVGANQ : 200
YP 008816000 : YILHPRGAIIGDTIVSGIEVPIKMGNALPLTDMPLGTAIHNIEITLGKGGQLVRAAGAVAKLIAKEGKSATLKLPSGEVRLISKNCSATVGQVGNVGANQ : 200
YP 007507153 : YILHPRGAIIGDTIVSGTEVPIKMGNALPLTDMPLGTAIHNIEITLGKGGQLVRAAGAVAKLIAKEGKSATLKLPSGEVRLISKNCSATVGQVGNVGANQ : 200
YP 004935708 : YILHPRGAIIGDTIVSGTEVPIKMGNALPLTDMPLGTAIHNIEITLGKGGQLVRAAGAVAKLIAKEGKSATLKLPSGEVRLISKNCSATVGQVGNVGANQ : 200
YP 004376461 : YILHPRGAIIGDTIVSGTEVPIKMGNALPLTDMPLGTAIHNIEITLGKGGQLVRAAGAVAKLIAKEGKSATLKLPSGEVRLISKNCSATVGQVGNVGANQ : 200
NP 054540    : YILHPRGAIIGDTIVSGTEVPIKMGNALPLTDMPLGTAIHNIEITLGKGGQLARAAGAVAKLIAKEGKSATLKLPSGEVRLISKNCSATVGQVGNVGVNQ : 200
               YILHPRGAIIGDTIVSGtEVPIKMGNALPLTDMPLGTAIHNIEITLGKGGQLvRAAGAVAKLIAKEGKSATLKLPSGEVRLISKNCSATVGQVGNVGaNQ

                         *       220         *       240         *       260         *       280         *       300
YP 009242721 : KSLGRAGSKRWLGKRPVVRGVVMNPVDHPHGGGEGRAPI------------------------------------------------------------- : 239
YP 009242105 : KSLGRAGSKRWLGKRPVVRGVVMNPVDHPHGGGEGRAPI------------------------------------------------------------- : 239
YP 009232109 : KSLGRAGSKRWLGKRPVVRGVVMNPVDHPHGGSVQGAPLLWVLSLMLIVSHLVVLRKGKVRLSAHFTTSSLDFPLLSFDLLFSSEREVISVTPSGLLCLV : 300
YP 009002296 : KSLGRAGSKRWLGKRPVVRGVVMNPVDHPHGGGEGRAPI------------------------------------------------------------- : 239
RPL2 SAMI    : KSLGRAGSKRWLGKRPVVRGVVMNPVDHP------QCS-------------------------------------------------------------- : 232
YP 008964096 : KSLGRAGSKRWRGKRPVVRGVVMNPVDHPHGGGEGRAPI------------------------------------------------------------- : 239
YP 008816000 : KSLGRAGSKRWLGKRPVVRGVVMNPVDHPHGGGEGRAPI------------------------------------------------------------- : 239
YP 007507153 : KSLGRAGSKRWLGKRPVVRGVVMNPVDHPHGGGEGRAPI------------------------------------------------------------- : 239
YP 004935708 : KSLGRAGSKRWLGKRPVVRGVVMNPVDHPHGGGEGRAPI------------------------------------------------------------- : 239
YP 004376461 : KSLGRAGSKRWLGKRPVVRGVVMNPVDHPHGGGEGRAPI------------------------------------------------------------- : 239
NP 054540    : KSLGRAGSKRWLGKRPVVRGVVMNPVDHPHGGGEGRAPI------------------------------------------------------------- : 239
               KSLGRAGSKRWlGKRPVVRGVVMNPVDHPhgggegrap
```

```
                       *      320         *      340         *      360         *      380         *      400
YP 009242721 : ---------GRKK---PT---------TPWG----YPA------------------LGRR---------SRKR---------------NK---------- : 262
YP 009242105 : ---------GRKK---PT---------TPWG----YPA------------------LGRR---------SRKR---------------NK---------- : 262
YP 009232109 : SPKEMVHIDGRTTGIEPARGGFTIHCLNPLGYIRPYPHTGLSLHLRSDPFLTPLSLLSKRSFFLSLQVLSRGKENFYFLKLKLPGKASNKTKRLGCSLHC : 400
YP 009002296 : ---------GRKK---PT---------TPWG----YPA------------------LGRR---------SRKR---------------NK---------- : 262
RPL2 SAMI     : ---------------------------------------------------------------------------------------------------- :   -
YP 008964096 : ---------GRKK---PT---------TPWG----YPA------------------LGRR---------SRKR---------------NK---------- : 262
YP 008816000 : ---------GRKK---PT---------TPWG----YPA------------------LGRR---------SRKR---------------NK---------- : 262
YP 007507153 : ---------GRKK---PT---------TPWG----YPA------------------LGRR---------SRKR---------------NK---------- : 262
YP 004935708 : ---------GRKK---PT---------TPWG----YPA------------------LGRR---------SRKR---------------NK---------- : 262
YP 004376461 : ---------GRKK---PT---------TPWG----YPA------------------LGSR---------SRKR---------------NK---------- : 262
NP 054540    : ---------GRKK---PT---------TPWG----YPA------------------LGRR---------SRKR---------------NK---------- : 262
                        grkk   pt         tpwg    ypa                  lg r         srk                 nk

                       *      420         *      440         *      460         *      480         *      500
YP 009242721 : --------------YSENWIVRRRSK-------------------------------------------------------------------------- : 274
YP 009242105 : --------------YSENLIVRRRSK-------------------------------------------------------------------------- : 274
YP 009232109 : FDFTLLKIKDRGRAGSEGSFSRQRASSKHLPTPNKMKSSKRNFQKRAPIRENHCTLASCIGFQTCAPLFYKLLSPYLLTKMGYLKKPLSTNLRRILSKIS : 500
YP 009002296 : --------------YSDNFIVRRRSK-------------------------------------------------------------------------- : 274
RPL2 SAMI     : --------------YSYLRIISI----------------------------------------------------------------------------- : 241
YP 008964096 : --------------YSDNLIVRRRSK-------------------------------------------------------------------------- : 274
YP 008816000 : --------------YSDNLIVRRRSK-------------------------------------------------------------------------- : 274
YP 007507153 : --------------YSDNLIVRRRSK-------------------------------------------------------------------------- : 274
YP 004935708 : --------------YSDNLIVRRRSK-------------------------------------------------------------------------- : 274
YP 004376461 : --------------YSDNLILRRRSK-------------------------------------------------------------------------- : 274
NP 054540    : --------------YSDNLILRRRSK-------------------------------------------------------------------------- : 274
                             yS n i rrrsk

                       *      520         *      540         *      560
YP 009242721 : ------------------------------------------------------------------- :   -
YP 009242105 : ------------------------------------------------------------------- :   -
YP 009232109 : SKSFLRMLERIPFLLVRLSKVRFLTKCLCANSGLKTLDQRERKGNSNNNIQCEGKVVVNHQLIGRRK : 567
YP 009002296 : ------------------------------------------------------------------- :   -
RPL2 SAMI     : ------------------------------------------------------------------- :   -
YP 008964096 : ------------------------------------------------------------------- :   -
YP 008816000 : ------------------------------------------------------------------- :   -
YP 007507153 : ------------------------------------------------------------------- :   -
YP 004935708 : ------------------------------------------------------------------- :   -
YP 004376461 : ------------------------------------------------------------------- :   -
NP 054540    : ------------------------------------------------------------------- :   -
```

Figure S3.31 The amino acid sequence allignment of RPL5 in mitogenome of *S. miltiorrhiza* and the corresponding homologs in other species.

```
                            *        20         *        40         *        60         *        80         *       100
YP 009243665 : ----MFPLYFHYEDVSRQDPLLKPNHANVMEVPGSCEIRVVPKAPYDF-IIKNGKLAMEIPRGQKFIQTERGSTGKSFRSNPFLGSNKDK-GYVSDLARQ :  94
YP 009121967 : ----MFPLYFHYEDVSRQDPLLKPNHANVMEVPGSCKIIVVPKTAPS---IKNGKLAMEIPCGQKLIQTQRASTGKSFRSNPFLGSNKDKKGYVSDLARQ :  93
XP 009759578 : MDKLMFPLYFHYEDVSRQDQLLKPNHANVMEVSRSCKIIVVPKTAPS---IKNGKLAMEIPCGQKLIQTQRASTGKSFRSNPFLGSNKDKKGYVSDLARQ :  97
YP 009049704 : ----MFPLYFHYEDVSRQDPLLKPNHANVMEVPGSCKIIVVPKTAPS---IKNGKLAMEIPCGQKLIQTQRASTGKSFRSNPFLGSNKDKKGYVSDLARQ :  93
YP 009041171 : ----MFPLYFHYEDVSRQDPLLKLNHANVMEVPGSCKIRVVPKAAPS---IKNGKLAMEIPRGQKLIQTQRVSTGKSFRSNPFLGSNKDKKGYVSDLARQ :  93
YP 008999601 : ----MFSLHFHYEDVLRQDLLLKLNYANVMEVPGLCKIRVVPKAAPSALIIKNGKLAMEISCGQKLIQTQRASTGKSFRSNP--GANKDKKGYVSDLARQ :  94
RPL5 SAMI     : ----MFPLYFHYEDVSRQDPLLKLNHANVMEVPGLCKIRVVPKAAPS---IKNGKLAMEIPCGQKCLQTQRSSTGREFRSNPFLGSNKDKKGYVSDLARQ :  93
YP 007889799 : ----MFPLHFHYEDVSRQDPLLKLNHANVMEVPGSCEIRVVPKAPYDF-IIKNGKLAMEIPRGQKFIQTQRGSTGKSFRSNPFLGSNKEK-GYVSDLARQ :  94
YP 007516856 : ----MFPLHFHYEDVSRQDPLLKLNHANVMEVPGSCEIRVVPKAPYDF-IIKNGKLAMEIPRGQKFIQTQRGSTGKSFRSNPFLGSNKDK-GYVSDLARQ :  94
YP 006460160 : ----MFPLYFHYEDVSRQDPLLKLNHANVMEVPGLCKIRVVPKAAPS---IKNGKLAMEIPCGQKLIQTQRSSTGKSFRSNPFLGSNKDKKGYVSDLARQ :  93
YP 173369     : ----MFPLYFHYENLSRQDLLLKPNHANVMEVPGSCKIIVVPKTAPS---IKNGKLAMEIPCGQKLIQTQRASTGKSFRSNPFLGSNKDKKGYVSDLARQ :  93
                   MFpL FHYE16sRQD LLK NhANVMEVpg C I VVPK      IKNGKLAMEIp GQK 6QT2R STG4sFRSNPflGsNKdK GYVSDLARQ

                        *       120         *       140         *       160         *       180         *
YP 009243665 : STLRGHGMSNFSVRISTVMSLLDSPVEIRENSIQFSMETEFCEFSPELEDHFEIFEHIRGFNVTIVTSANTQDETLPPWSGFLQKDEGETQ : 185
YP 009121967 : STLRGHGMSNFLVRISTVMSLLDSPVEIRERSIQFSMETEFCEFSPELEDHFEIFEHIRGFNVTIVTSANTQDETLPPWSGFLQKDEGESQ : 184
XP 009759578 : STLRGYGMSNFLVRVSTVMSLLDSPFEIRERSIQFSMETEFCKFSPELEDHFKIFEHIRGFNVTNVTSANTEDETLPPWSDFLQKDEGKTQ : 188
YP 009049704 : STLRGHGMSHFLVRISTVMSLLDSPLEIRERSIQFSMETEFCEFSPELEDHFEIFEHIRGFNVTIVTSANTQDETLPPWSGFLQKDEGETQ : 184
YP 009041171 : STLRGHGMSNFLVRISTVMSLLDSPVEIRENSIQFSMETEFCEFSPELEDHFEIFEHIRGFNVTIVTSANTQDETLPPWSGFLQKDEGESQ : 184
YP 008999601 : STLRGHGMSNFLVRISTVMSLLDSPVEIRENSIQFSMETEFCEFSPELEDHFEIFEHIRGFNVTIVTSANTQDETLLLWSGFLQKDEGETQ : 185
RPL5 SAMI     : STLRGHGMSNFLVRISTVMSLLDSPVEIRENSIQFSMETEFCEFSPELEDHFEIFEHIRGFNVTIVTSSNTQDETLPPWSGFLQKDEGETQ : 184
YP 007889799 : STLRGHGMSNFSVRISTVMSLLDSPVEIRENSIQFSMETEFCEFSPELEDHFEIFEHIRGFNVTIVTSANTQDETLPPWSGFLQKDEGETQ : 185
YP 007516856 : STLRGHGMSNFSVRISTVMSLLDSPVEIRENSIQFSMETEFCEFSPELEDHFEIFEHIRGFNVTIVTSANTQDETLPPWSGFLQKDEGETQ : 185
YP 006460160 : STLRGHGMSNFLVRISTVMSLLDSPVEIRENSIQFSMETEFCEFSPELEDHFEIFEHIRGFNVTIVTSSNTQDETLPPWSGFLQKDEGETQ : 184
YP 173369     : STLRGHGMSNFLVRISTVMSLLDSPVEIRERSILFSMETEFCEFSPELEDHFEIFEHIRGFNVTIVTSANTQDETLPPWSGFLQKDEGETQ : 184
               STLRGhGMSnF VR6STVMSLLDSP EIRE SIqFSMETEFCeFSPELEDHFeIFEHIRGFNVTiVTSaNT2DETLppWSgFLQKDEGe3Q
```

Figure S3.32 The amino acid sequence allignment of RPL10 in mitogenome of *S. miltiorrhiza* and the corresponding homologs in other species.

```
                          *        20         *        40         *        60         *        80         *       100
YP 009241685 : MPFGRSLLQREESLLRVSGEERSPEILISFHSSGSTSNQWRKLKNPWFPGRTPFRPSCCETGKKKRFFAQLAHSAGPTCISYLAEEASDRLEFLPSWDSMD : 100
YP 009177640 : MPFGRSLLQKESLYRVSGEERSPEILILFHSSGSTSNQWRKLKNPWFPGRTPFRPSCCGTGKKKRFFAQLAHSAGPTCILYLAEEASDRLEFLPSWDSMD : 100
YP 009153922 : MPFGRSLLQKESLYRVSGEERSPEILILFHSSGSTSNQWRKLKNPWFPGRTPFRPSCCGTGKKKRFFAQLAHSAGPTCILYLAEEASDRLEFLPSWDSMD : 100
YP 009121962 : MPFGRSLLQKKSLLRVSGEERSPEILISFHSSGSTSNQWRKLKNPWFPGRTLFRPSCFGTGKKKRFFAQLAHSAGPTCISYLAEEASDRLEFLPSWDSMD : 100
YP 009049779 : MPFGRSLLQKKSLLRVSGEERSPEILISFHSSGSTSNQWRKLKNPWFPGRTLFRPSCFGTGKKKRFFAQLAHSAGPTCISYLAEEASDRLEFLPSWDSMD : 100
YP 009041180 : MPFGRSLLQKKSLLRVSGEERYPEILISFHSSGSTSNQWRKLKNPWFPGRTLFRPSCFGTGKKKRFFAQLAHSAGPTCISYLAEKASDRLEFLPSWDSMD : 100
YP 008999559 : MPFGRSLLQRESLLRVSGEERSPEILISFHSSGSTSNQWRKLKNPWFPGRTLFRPSCFRTGKKKRFFAQLAHSAGPTCISYLAEEASDRLEFLPSWDSMD : 100
RPL10 SAMI   : MPFGRSILQKKSLLRVSGEERSTEILISFHSSGSTSNQWRKLKNPWFPGRTLFHPSCFGTGKKKWFFAQLAHSAGPTCILYLAEEASDRLEFLPSWDSMD : 100
YP 008802497 : MPFGRSLLQKKSLLRVSGEERSPEILISFHSSGLTSNQWRKLKNLWFPGRTLFRPSCFGTGKKKRFFAQLAHSAGPTCILYLAEKASDRLEFLPSWDSMD : 100
YP 006460164 : MPFGRSILQKKSLLRVSGEERSTEILISFHSSGSTSNQWRKLKNPWFPGRTLFRPSCFGTGKKKRFFAQLAHSAGPTCILYLAEEASDRLEFLPSWDSMD : 100
YP 006291832 : MPFGKSILQRESLLRVSGEERSPEILISFHSSGSTSNQWRKLKNPWFPGRTLFRPSCFGTGKKKRFFAQLAHSAGPTCISYLAEEASDRLEFLPSWDSMD : 100
               MPFG4S6LQ4 SLlRVSGEERspEILIsFHSSGsTSNQWRKLKNpWFPGRT FrPSC gTGKKKrFFAQLAHSAGPTCI YLAEeASDRLEFLPSWDSMD

                          *       120         *       140         *       160
YP 009241685 : QDLLSLYGQYRS--TLVDHMDVEKASDFDELETSLFHFYLPSSYLCFVCSREEFDLFNLGIPPK : 162
YP 009177640 : QDLLSLYGQYRS--TLVDHMDVEKASDFDEFETSLFHFYLPSSYLCFVCSREEFDLFNLGIPPK : 162
YP 009153922 : QDLLSLYGQYRS--TLVDHMDVKKASDFDEFETSLFHFYLPSSYLCFVCSREEFDLFNLGIPPK : 162
YP 009121962 : QDLLLLYGQYRS--TLVDHMDVEKASHLDEIETSLFHFYLPSSYLCFVCSWEE---FDLGIPPK : 159
YP 009049779 : QDLLLLYGQYRS--TLVDHMDVEKATNLDEIETSLFHFYLPSSYLCFVCSWEE---FDLGIPPK : 159
YP 009041180 : QDLLLLYGQYRS--TLVDHMDVEKAYNLDELETSFFHFYLPSSYLCFVCSREELYLFNLGIPPK : 162
YP 008999559 : QDLLLLYGQYRS--TLVDHMDVEKASHFDELETSLFHFYLPSSYLCFVCSPEEFDLFNLGIPPK : 162
RPL10 SAMI   : QDLLLLYGQYRS--TLADHMDVEKASHFDELETSLFHFYLPSSYLSFVCSREEFDLFNLGIPPK : 162
YP 008802497 : QDLLLLYGQYRS--TLVDHMDVEKASNLDELETYFFHFYLPSSYLCFVCSREELYLFNLSIPPK : 162
YP 006460164 : QDLLLLYGQYRS--TLADHMDVEKASHFNELETSLFHFYLPSSYLSFVCSREEFDLFNQSRNTT : 162
YP 006291832 : QDLLLLYGQYRSTFTLVDHMDVEKTYHFDELETSLFNFYLPSSYLCFVCSREELNLLNLGIPPK : 164
               QDLL LYGQYRS  TLvDHMDVeKa   1E ETslFhFYLPSSYLcFVCS EE  lf1lgippk
```

Figure S3.33 The amino acid sequence allignment of RPL16 in mitogenome of *S. miltiorrhiza* and the corresponding homologs in other species

```
                      *        20         *        40         *        60         *        80         *       100
RPL16 SAMI   : ---------------------------MSKCGFHIVKKERGVLYPKRTKYTKYRKGRCSRGCKADGTQLGFGRYGTKSCRAGRLSYRAIEAARRAIIGHF :  73
YP 006460180 : ---------------------------VSKCGFHIVKKERGVLYPKRTKYSKYRKGRCSRGCKPDGTQLGFGRYGTKSCRAGRLSYRAIEAARRAIIGHF :  73
YP 009121949 : --------------MLLRKYLLVTESQVSKCGFHIVKKKRDVLYPKRTKYSKYRKGRCSRGCKPDGTQLGFGRYGTKSCRAGRLSYRAIEAARRAIIGHF :  86
YP 173411     : --------------MLLRKYLLVTESQVSKCGFPIVKKKRDVLYPKRTKYSKYRKGRCSRGCKPDGTQLGFGRYGTKSCRAGRLSYRAIEAARRAIIGHF :  86
YP 005090456 : MEKHLVMYLTRKSIMLLRKYLLVTESQVSKCGFHIVKKKRDVLYPKRTKFSKYRKGRCSRGCKPDGTKLGFGRYGIQSCRAGRLSYRAIEAARRAIIGHF : 100
YP 009049821 : ---------------------------VSKCGFHIVKKKGDVLYPKRTKYSKYRKGRCSRGCKPDGTQLGFGRYGTQSCRAGRLSYRAIEAARRAIIGHF :  73
YP 006665986 : ------MYLTRKSIMLLRKYLLVTESQVSKCGFHIVKKKGDVLYPKRTKYSKYRKGRCSRGCKPDGTKLGFGRYGTKSCKAGRLSYRAIEAARRAIIGHF :  94
YP 007516918 : --------------MLLRKYLLVTESQVSKCGFHIVKKKGDVLYPKRTKFSKYRKGRCSRGCKPDGTKLGFGRYGTQSCRAGRLSYRAIEAARRAIIGHF :  86
YP 005090475 : MEKHLVMYLTRKSIMLLRKYLLVTESQVSKCGFHIVKKKGDVLYPKRTKFSKYRKGRCSRGCKPDGTKLGFGRYGIQSCRAGRLSYRAIEAARRAIIGHF : 100
YP 004927493 : ------MYLTRKSIMLLRKYLLVTESQVSKCGFHIVKKKGDVLYPKRTKYSKYRKGRCSRGCKPDGTKLGFGRYGTKSCKAGRLSYRAIEAARRAIIGHF :  94
XP 006398028 : ------MYLTRKSIMLLPKYLLVTESQVSKCGFHIVKKKGDVLYPKRTKYSKYRKGRCSRGCKPDGTKLGFGRYGTKSCKAGRLSYRAIEAARRAIIGHF :  94
                                          6SKCGFhIVKKk dVLYPKRTK53KYRKGRCSRGCKpDGT LGFGRYGt SC4AGRLSYRAIEAARRAIIGHF

                      *       120         *       140         *       160         *       180
RPL16 SAMI   : HRAMSGQFRRNGKIWVRVFADIPITGKPTEVRMGRGKGNPTGWIARVSTGQILFEMDGVSLSNARQAATLAAHKLCSSTKFVQWS : 158
YP 006460180 : HRAMSGQFRRNGKIWVRVLADIPITGKPTEVRMGRGKGNPTGWIARVSTGQILFEMDGVSLSNAQQAATLAAHKLCSSTKFVQWS : 158
YP 009121949 : HRAMSGQFRRNGKIWVRVLADIPITGKPTEVRMGRGKGNPTGWIARVSRGQILFEMDGVSLSNARQAATLAAHKLCSSTKFLQWS : 171
YP 173411     : HRAMSGQFRRNGKIWVRVLADIPITGKPTEVRMGRGKGNPTGWIARVSRGQILFEMDGVSLSNARQAATLAAHKLCSSTKFVQWS : 171
YP 005090456 : HRAMSGQFRKNGKIWVRVFADIPITGKPTEVRMGRGKGNPTGWIARVSTGQVLFEMDGVSLSNARQAATLAAHKLCLSTKFVQWS : 185
YP 009049821 : HRAMSGQFRRNGKIWVRVLADIPITGKPTEVRMGRGKGNPTGWIARVSRGQILFEMDGVSLSNARQAATLAAHKLCSSTKFVQWS : 158
YP 006665986 : HRAMSGQFRRNGKIWVRVFADLPITGKPTEVRMGRGKGNPTGWIARVSTGQIPFEMDGVSLANARQAATLAAHKLCSSTKFVQWS : 179
YP 007516918 : HRAMSGQFRKNGKIWVRVFADIPITGKPTEVRMGRGKGNPTGWIARVSTGQVLFEMDGVSLSNARQAATLAAHKPCSSTKFVQWS : 171
YP 005090475 : HRAMSGQFRKNGKIWVRVFADIPITGKPTEVRMGRGKGNPTGWIARVSTGQVLFEMDGVSLSNARQAATLAAHKLCLSTKFVQWS : 185
YP 004927493 : HRAMSGQFRRNGKIWVRVFADLPITGKPTEVRMGRGKGNPTGWIARVSTGQIPFEMDGVSLANARQAATLAAHKPCSSTKFVQWS : 179
XP 006398028 : HRAMSGQFRRNGKIWVRVFADLPITGKPTEVRMGRGKGNPTGWIARVSTGQIPFEMDGVSLANARQAATLAAHKPCSSTKFVQWS : 179
               HRAMSGQFR4NGKIWVRV AD6PITGKPTEVRMGRGKGNPTGWIARVS GQ6 FEMDGVSL NArQAATLAAHK CsSTKF6QWS
```

Figure S3.34 The amino acid sequence allignment of RPL23 in mitogenome of *S. miltiorrhiza* and the corresponding homologs in other species.

```
                       *        20         *        40         *        60         *        80         *
YP 009262355 : MDGIKYAVFTDKSIRLLGKNQYTSNVESGSTRTEIKHWVELFFGVKVIAMNSHQLPGKGRRMGPIMGHTMHYRRMIITLQPGYSIPPLRKKRT : 93
YP 009236321 : MDGIKYTVFTDKSIRLLGKNQYTSNVESGSTRTEIKHWVELFFGVKVIAMNSHRLPGKGRRMGPIMGHTMHYRRMIITLQPGYSIPPLRKKRT : 93
YP 009186210 : MNGIKYAVFTDKSIRLLGKNQYTSNVESGSTRTEIKHWVELFFGVKVIAMNSHRLPGKGRRMGPIMGHTMHYRRMIITLQPGYSIPPLRKKRT : 93
YP 009183032 : MDGIKYAVFTDKSIRLLGKNQYTSNVESGSTRTELKHWVELFFGVKVIAMNSHRLPGKGKRMGPIMGHTMHYRRMIITLQPGYSIPPLRKKRT : 93
YP 009108632 : MDGIKYAVFTDKSLRLLGKNQYTSNVESGSTRTEIKHWVELFFGVKVIAMNSHRLPGKGRRMGPIMGHTMHYRRMIITLQPGYSIPPLRKKRT : 93
YP 008082775 : MDGIKYVVFTDKSIRLLGKNQYTSNVESGSTRTEIKHWVELFFGVKVIAMNSHRLPGKGRRMGPIMGHTMHYRRMIITLQPGYSIPPLRKKRT : 93
YP 007353957 : MDGIKHAVFTDKSIRLLGKNQYTSNVESGSTRTELKHWVELFFGVKVIAMNSHRLPGKGRRMGPIMGHTMHYRRMIITLQPGYSIPPLRKKRT : 93
YP 004940551 : MDGIKYAVFTDKSIRLLGKNQYTSNVESGSTRTELKHWVELFFGVKVIAMNSHRLPGKGRRMGPIMGHTMHYRRMIITLQPGYSIPPLRKKRT : 93
YP 247642    : MDGIKYAVFTDKSIRLLGKNQYTSNVESGSTRTEIKHWVELFFGVKVIAMNSHRLPGKGRRMGPIMGHTMHYRRMIITLQPGYSIPPLRKKRT : 93
NP 054541    : MDGIKYAVFTDKSIRLLGKNQYTSNVESGSTRTEIKHWVELFFGVKVIAMNSHRLPGKSRRMGPIMGHTMHYRRMIITLQPGYSIPPLRKKRT : 93
RPL23 SAMI   : MDGIKYAVFTDKSIRLLGKNQYTSNVESGSTRTELKHWVELFFGVKVIAMNSHRLPGKGRRMGPIMGHTMHYRRMIITLQPGYSIPPLRKKRT : 93
               M1GIKyaVFTDKS6RLLGKNQYTSNVESGSTRTE6KHWVELFFGVKVIAMNSHrLPGKg4RMGPIMGHTMHYRRMIITLQPGYSIPPLRKKRT
```

Figure S3.35 The amino acid sequence allignment of RPS3 in mitogenome of *S. miltiorrhiza* and the corresponding homologs in other species.

```
                                     *        20         *        40         *        60         *        80         *       100
YP 009270680 : MARKGNPISVRLDLNRSSDSSWFSDYYYGKLVYQDVNLRSYFGSIRPPTRLTFGFRLGRCIILHFPKRTFIHFFLPRRPRRLKRRDKSRPGKEKGRWWAF : 100
YP 009241675 : MARKGNPISVRLDLNRSSDSSWFSDYYYGKSVYQDVNLRSYFGSIRPPTRLTFGFRLGRCIILHFPKRTFIHFFLPRRPRRLKRREKSRPGKEKGRWWAF : 100
YP 009121948 : MARKGNPISVRLDLNRSSDSSWFSDYYYGKSVYQDVNLRSYFGSIRPPTRLTFGFRLGRCIIIHFPKRTFIHFFLPRRPRRLKRREKSRPVKEKGRWGAF : 100
YP 009049819 : MARKGNPISVRLDLNRSSDSSWFSDYYYGKSVYQDVNLRSYFGSIRPPTRLTFGFRLGRCIIIHFPKRTFIHFFLPRRPRRLKRREKSRPVKEKGRWGAF : 100
YP 009041176 : MARKGNPISVRLDLNRSSDSSWFSDYYYGKSVYQDVNLRSYFGSIRPPTRLTFGFRLGRCIILHFPKRTFIHFFLPRRPRRLKRRDKSRPGKEKGRWWAF : 100
RPS3 SAMI    : MARKGNPISVRLDLNRSSDSSWFSEYYYGKSLYQDLNLRSYFGSIRPPTRLTFGFRLGRCIIIHFPKRTFIHFFLPRRPRRLKRREKSR----KGRWWEF :  96
YP 006666137 : MARKGNPISVRLDLNRSSDSSWFSDYYYGKLVYQDLNLRSYFGSIRPPTRLTFGFRLGTCIILHFPKRTFIHFFLPRRPRRLKRREKSRPGKEKGRWWAF : 100
YP 006460179 : MARKGNPISVRLDLNRSSDSSWFSDYYYGKSVYQDLNLRSYFGSIRPPTKLTFGFRLGRCIIIHFPKRTFIHFFLPRRPRRLKRRAKSRPGKAKSRRWEL : 100
YP 005090420 : MARKGNPISVRLDLNRSSDSSWFSEYYYAKSVYQDLNLRSYFGSIRPPTRLTFGFRLGRCIILHFPKRTFLHLFLPRRPRRLKRREKSRPGKAKGRWWEF : 100
YP 002608370 : MARKGNPISVRLDLNRSSDSSWFSDYYYGKSVYQDVNLRSYFGSIRPPTRLTFGFRLGRCIILHFPKRTFIHFFLPRRPRGLKRREKSRPGKEKGRWWAF : 100
YP 173410    : MARKGNPISVRLDLNRSSDSSWFSDYYYGKSVYQDVNLRSYFGSIRPPTRLTFGFRLGRCIIIHFPKRTFIHFFLPRRPRRLKRREKSRPVKEKGRWGAF : 100
               MARKGNPISVRLDLNRSSDSSWFSdYYYgKs6YQD6NLRSYFGSIRPPT4LTFGFRLGrCII6HFPKRTF6HfFLPRRPRrLKRR KSRp k KgRw  f

                                     *       120         *       140         *       160         *       180         *       200
YP 009270680 : GKVGPIGCLHSSDGTEEERNEVRGRGAGKRVESIRLDDREKQNEIRIWPQKKQRYGYHDRSPSRKKNLSKLLRVSGAFKHPKYAGVVNDIAFLIENDD-- : 198
YP 009241675 : GKVGPLRCLHSSDDTEEERNEVRGRGEGKRVESIRLDDREKQNEIRIWPKKKQRYGYHERSPSIKKNLSKSLRVSRAFKHPKYAGVVNDIAFLIENDD-- : 198
YP 009121948 : GKVGPIGCLHSSDGTEEERNEVRGRGAGKRVESIRLDDREKQNEIRIWPKKKQGYGYHDRSPSIKKNLSKSLRVSGAFKHPKYAGIENDIAFLIENDD-- : 198
YP 009049819 : GKVGPIGCLHSSDGTEEERNEVRGRGAGKRVESIRLDDREKQNEIRIWPKKKQGYGYHDRSPSIKKNLSKSLRVSGAFKHPKYAGIENDIAFLIENDD-- : 198
YP 009041176 : GKVGPIGCLHSNDGTEEERKEVRGRGAGKRVESIRLDDREKQNEIRIWPKKKQGYGYHDRSPSIKKNISKSLRGSGAFKHPKYAGVVNDIAFLIENDD-- : 198
RPS3 SAMI    : GKVGPIGCLHSSDGTEEERKEVRGRGSGKRVESIRLDDRKKQNEIRIWPKKKQGYGYHDRSPSIKKNLSKSLRVSGAFKHPKYAGVVNGIAFLIENDDSF : 196
YP 006666137 : GKVGPIGCLHSSGDTEEERNEVRGRGSGKRVESIRLDDREKQNEIRIWPKKKQRYGYHDRSPSIKKNLSKSLRVSRAFKHPKYAGVVNGIAFLIENDD-- : 198
YP 006460179 : GKVGPIRCLHSSDGTEEERNEVRGRGSGKRVESIRLDDRKKQNEIRIWPKKKQGYGYHDRSPSIKKNLSKSLRVSGAFKHPKYAGVLNG----------- : 189
YP 005090420 : GKVGPIGCLHSSDGTEEERNEVRGRGSGERVESIRLDDREKQNEIRIWPKKKQGYGYHDRSPSIKKNLSKSLRVSGAFKHPN-AGVVNGIAFLIENDD-- : 197
YP 002608370 : GKVGPIGCLHSSDGTEEERNEVRGRGAGKRVESIRLDDREKQNERRIWPKKKQRYGYHDQSPSIKKNLSKLLRVSGAFKHLKYAGVVNDIAFLIENDD-- : 198
YP 173410    : GKVGPIGCLHSSDGTEEERNEVRGRGAGKRVESIRLDDREKQNEIRIWPKKKQGYGYHDRSPSIKKNLSKSLRVSGAFKHPKYAGIENDIAFLIENDD-- : 198
               GKVGP6gCLHSsdgTEEERnEVRGRG GkRVESIRLDDReKQNEiRIWPkKKQ YGYHdrSPSiKKN6SKsLRvSgAFKHpkyAG6 N iafliendd

                                     *       220         *       240         *       260         *       280         *       300
YP 009270680 : SFRKTKLFKFFFPKKSRSDGPTSHLLKRTLPAVRPSLNYLVMQYLLNTKNKIHFDPVVVLNHFVAPGVAEPSTMGGANEQGRSLDKRIRSRIAFFVES-- : 296
YP 009241675 : FFRKTKLFKFFLPKKSRSDGPTSHLLKRTLPAVRPSLNYSVMQYLLNTKKKMDFDPVVVLNHFVAPGVSEPSTMGGANAQGRSLDKRIRSRIAFFVESST : 298
YP 009121948 : SFRKTNLFKFFFPKKSRSDRPTSHLLKRTLPAVRPSLNYSVMQYLLNTKNKMHFDPVVVLNHFVAPGVAEPSTMGGANAQGRSLDKRIRSCIAFFVES-- : 296
YP 009049819 : SFRKTNLFKFFFPKKSRSDRPTSHLLKRTLPAVRPSLNYSVMQYLLNTKKKMHFDPVVVLNHFVAPGVAEPSTMGGANAQGRSLDKRIRSCIAFFVES-- : 296
YP 009041176 : SFRKTKLFKFFFPKKSRSNGPTSHLLKKTLPAVRPSLNYLVMQYLLNTKNKIHFDPVVVLNHFVAPGVAEPSTMGGANAQERSLDKRIRSRIAFFVES-- : 296
RPS3 SAMI    : INIKTKLFLFFFPKKSR--GPTSHLQQGTLPAVRSSLNYSVMQYLLNTKNKMHFDPVVVLNHFVAPGVAEPSTMGRANAQERSLDKRIRSRIAFFVES-- : 292
YP 006666137 : PFRKTKLFKFFLPKKSRSDGSTSHLLKRTLPAVRPSFNYSVMQYLLNTKNQMHFDPVVVLNHFVAPGVAEPSTMGGANAQGRSLDKRIRSRIALFVES-- : 296
YP 006460179 : IFRKTKLFLFFCPKKSRSDGPTSHLQQRTLPAVRSSLNYSVMQYLLNTKNKMHFDPVVVLNHFVAPGVAEPSTMGGANAQERSLDKRIRARIAFFVES-- : 287
YP 005090420 : SFRKTKLFKFFFPKKSRSDGPTSHLLKRTLPAVRSSLNYSVMQYLLNTKKKMHFDPVVVLNHFVAPGVAEPSTMGGANAQERSLDKRIRSRIAFFLES-- : 295
YP 002608370 : SFRKTKLFKFFFPKKSRSDGPTSHILKRTLPAVRPSLNYSVMQYLLNTKNQIHFDSVVVLNHFVAPGVAEPSTMGGANAQGRSLDKRIRSRIAFFVES-- : 296
YP 173410    : SFRKTNLFKFFFPKKSRSDRPTSHLLKRTLPAVRPSLNYSVMQYLLNTKNKIHFDPVVVLNHFVAPGVAEPSTMGGANAQGRSLDKRIRSCIAFFVES-- : 296
                frKT LFkFF PKKSRs  pTSH6lk TLPAVR SlNYsVMQYLLNTK k6hFDpVVVLNHFVAPGVaEPSTMGgANaQ RSLDKRIRs IAfF6ES
```

```
                   *     320       *     340       *     360       *     380       *     400
YP 009270680 : LTSEKKCLAEAKKRLTHFIRQANDLRFAGTTKTTISLFPFFGATFFFLRDGVGVYNNLFIEDAREQ--LLGQLRIKCWNLMGKEKVMELIEKFIDLGGIG : 394
YP 009241675 : STSEKKCLAEAKKRLTHFIREANDLRFAGTTKTTISLFPFFGATFFFPRDGVGMYKNLFFEDAREQ--LLGQLRIKCWNLMGKDKVMELIEKFIDLGGIG : 396
YP 009121948 : STSEKKCLAEAKKRVTHFIRQANDLRFAGTTKTTISLFPFFGATFFFPRDGVGVYNNLFFEDAREQ--LLGQLRRKCWNLMGKDKVMELIEKFIDLNRIG : 394
YP 009049819 : STSEKKCLAEAKKRVTHFIRQANDLRFAGTTKTTISLFPFFGATFFFPRD--GVYNNLFFEDAREQ--LLGQLRRKCWNLMGKDKVMELIEKFIDLNRIG : 392
YP 009041176 : STSEKKCLAEAKKRLTHFIRQANDLRFAGTTKTTTSLFPFFGATFFFLRDGVGVYNNLFFEDAREQ--LLGKLRRKCWNLMGKDKVMELIEKFIDLGRIG : 394
RPS3 SAMI    : STSD---LAEAKKGLTHFIRQANDLRFAGTTKTTISLFPFFGATFFFLRDGVGVYNNLFFENAREQ--LLGQCW----NLMAKDKVMELIDKFIDLGRIG : 383
YP 006666137 : STSDKKCLAEAKKRLTRFIRQANDLRFAGTTKTTIPLFPFFGATFFFPRDGVGMYNNLFFEDAREQ--LLGQLRIKCWNLMGKDKVMELIEKFIDLDRIG : 394
YP 006460179 : STSEKKCLAEAKNRLTHFIRQANDLRFAGTTKTTISLFPFFGATFFFPRD--GVYNNLFFENAREQQKLLGQCWRKCWNLMAKDKVMELIDKFIDLGRIG : 385
YP 005090420 : STSEKKCLAEAKKRLTHFIRQAIDLRFAGTTKTTISLFPFFGATFFFPRV--GVYNNLFFENARQQ--LLRQLRRKCWNLMAKDKVMELIEKFIDLGRIG : 391
YP 002608370 : STSEKKCLAEAKKRLTHFIRQANDLRFAGTTKTTISLFPFFGATFFFPRDGVGVYNNLFFEDAREQ--LLGQLRIKCWNLMGKDKVMELIDKFIDLGGIG : 394
YP 173410    : STSEKKCLAEAKKRVTHFIRQANDLRFAGTTKTTISLFPFFGATFFFPRDGVGVYNNLFFEDAREQ--LLGQLRIKCWNLMGKDKVMELIEKFIDLNRIG : 394
               sTSekkcLAEAKkr6ThFIR2AnDLRFAGTTKTTisLFPFFGATFFF Rd  G6YnNLFfE1AR2Q  LLgqlr kcwNLM KdKVMELI KFIDL  IG

                   *     420       *     440       *     460       *     480       *     500
YP 009270680 : ELIKGIEMMIEIILRNRRIPYGYNSYLNEVKKMRSLLSNRTNTNTLIESVKIKSVYQSASLIAQDISFQLRNKT-RSFRSIFSQIVKDIPLVMPKGVEGI : 493
YP 009241675 : ELIKGIEMMIEIILRNRRIPYGYNSYLNEVKKMRSLLSNRAHTNTLMESVKIKSVYQSASPIAQDISFQLRNKT-RSFRSIFSKIVKDIPLIMKKGVEGI : 495
YP 009121948 : ELIRGIEMMIEIILRNRRIPYGYNYYLNEVKKMRSLLYNRTNTNTNIESVKIKSVYQSASPIAQDISFQPRNKT-RSFRSIFSQIVKDIPLVMKKGVEGI : 493
YP 009049819 : ELIRGIEMMIEIILRNRRIPYGYNYYLNEVKKMRSLLYNRTNTNTLIESVKIKSVYQSASPIAQDISFQPRNKT-RSFRSIFSKIVKDIPLVMKKGVEGI : 491
YP 009041176 : ELIKGIEMMIEIILRNRRIPYGYNSYLNEVKKMRSLLSNRTNTNTLIESVKIKSVYQSASPIAQDISFQPRNKT-RSFRSIFSKIVKDIPLVMKKGVEGV : 493
RPS3 SAMI    : ELIKGIEMMIEIILRNRRIPYGYNSYLNEVKKMRSLLSNRTNTNTLIESVKIKSVYQSASPIAQDISFQLRTKKTRSFRSIFSKIVKNIPLVMKKRVTGI : 483
YP 006666137 : ELIRGIEMMIEIILRNRQIPCGYNSYLNEVKKMRSLLSNRTNTNTLIESVKIKSVYQSASPIAQDISFQLRNKT-RSFRSIFSKIVKDIPLVMKKGVEGI : 493
YP 006460179 : ELIKGIEMMIEIILRNRRIPYGYNSYLNEVKKMRSLLSNRTNTNTLIESVKIKSVYQSASPIAQDISFQLRTKR-RSFRSIFSHIVKNIPLVMKKGVTGI : 484
YP 005090420 : ELIKGIEMMIEIILRNRRIPYGYNSYLNEMKKMRSLLSNRTNTNTLIESVKIKSVYQSASPIAQDISFQLRTKT-RSFRSIFTKIVKNIPLVMKKGVEGI : 490
YP 002608370 : ELIKGIEMMIEIILRNRRIPYGYNSYLNEVKKMRSLLSNRTNTNTLIESVKIKSVYQSASPIAQDISFQPRNKT-RSFRSIFSKIVKDIPLVMKKGVEGI : 493
YP 173410    : ELIRGIEMMIEIILRNRRIPYGYNYYLNEVKKMRSLLYNRTNTNTLIESVKIKSVYQSASPIAQDISFQPRNKT-RSFRSIFSKIVKDIPLVMKKGVEGI : 493
               ELI4GIEMMIEIILRNRrIPyGYN YLNE6KKMRSLL NRtnTNTl6ESVKIKSVYQSASpIAQDISFQ R Kt RSFRSIF3 IVK1IPL6MkKgVeG6

                   *     520       *     540       *     560       *
YP 009270680 : RICCSGRLEGAEIARTECGKYGKTSCNVFNQKIDYASAEVSTRYGILGVKVWISYSQKNKGRAISETYEI- : 563
YP 009241675 : RICCSGRSEGAEIARTECGKYGKTSRNVFNQKIDYAPAEVSTRYGISGVKVWISYSKNKKGRAISETYEIY : 566
YP 009121948 : RICCSGRLEGAEIARTECGKYGKTSRNVFNQKIDYAPAEVSTRYGISGVKVWISYSKKKKGRAISETYEI- : 563
YP 009049819 : RICCSGRLEGAEIARTECGKYGKTSRNVFNQKIDYAPAEVSTRYGISGVKVWISYSKKKGGRAISETYEI- : 561
YP 009041176 : RICCSGRLEGAEIARTECGKYGKTSRNVFNQKIDYAPAEVSTRYGISGVKVWISYSKKQKGRAISETYEI- : 563
RPS3 SAMI    : RICCSGRSKGAKIARTECGKYGKISRNVFNQKIDYAPAEVSTRYGILGVKVWISYSQKRKGRAISKTYEIY : 554
YP 006666137 : RICCSGRSKGAEIARTECGKYGKTSRNVFNQKIDYAPAKVSTRYGILGVKVWISYSKKK---SI------- : 554
YP 006460179 : RICCSGRSKGAEIARTECGKYGKTSRNVFNQKIDYAPAEVSTRYGILGVKVWISYSQKRKGRAISKTYEI- : 554
YP 005090420 : RICCSGRSEGAEIARTECGKYGKTSRNVFHQKIDYAPAEVSTRYGILGVKVWISYSQKRRGRAISKTY--- : 558
YP 002608370 : RICCSGRSEGAEIARTECGKYGKTSRNVFNQKIDYAPAEVSTRYGISGVKVWISYSKKKKGRAISETYEI- : 563
YP 173410    : RICCSGRLEGAEIARTECGKYGKTSRNVFNQKIDYAPAEVSTRYGISGVKVWISYSKKKKGRAISETYEI- : 563
               RICCSGR  GAeIARTECGKYGKtSrNVFnQKIDYApAeVSTRYGI GVKVWISYS k  graIs tyei
```

Figure S3.36 The amino acid sequence allignment of RPS4 in mitogenome of *S. miltiorrhiza* and the corresponding homologs in other species

```
                        *        20         *        40         *        60         *        80         *       100
XP 016500822 : MASRYTLQWIRRIIHLTLGFGKDIYETEVSFKRKTGVDFFALTGCEHGNCVAEQRAERNLVLQMLKPERDGPFEEGIFLVNISPARMDFRSFLVDNFIAI : 100
YP 009241446 : ---------------------------------------------------------------------------------------------------- :   -
YP 009121977 : ---------------------------------------------------------------------------------------------------- :   -
XP 011077036 : ---------------------------------------------------------------------------------------------------- :   -
XP 010314945 : ---------------------------------------------------------------------------------------------------- :   -
YP 009049648 : ---------------------------------------------------------------------------------------------------- :   -
YP 009041168 : ---------------------------------------------------------------------------------------------------- :   -
RPS4 SAMI    : ---------------------------------------------------------------------------------------------------- :   -
YP 006460178 : ---------------------------------------------------------------------------------------------------- :   -
YP 005090421 : ---------------------------------------------------------------------------------------------------- :   -
YP 173472    : ---------------------------------------------------------------------------------------------------- :   -

                        *       120         *       140         *       160         *       180         *       200
XP 016500822 : LSSLVTPAQATAVMDLIEERWEEWIGEMPLKITYPALEGHEWRIVTGFDPKNTGRSYLIDQGSRFYVKYPEKPKPLIPVLRKTTTSQELRKRRDVVSNNS : 200
YP 009241446 : ---------------------------------------------------------------------------------------------------- :   -
YP 009121977 : ---------------------------------------------------------------------------------------------------- :   -
XP 011077036 : ---------------------------------------------------------------------------------------------------- :   -
XP 010314945 : -------------MDLIEGRWEEWIGEMPLKITYPALEGHEWRIVTGFDPKNTGRSYLIEERGVQPLDCKEGGKP------KPT---------------- :  65
YP 009049648 : ---------------------------------------------------------------------------------------------------- :   -
YP 009041168 : ------------------------MSRFSLCSVYRSVSTRRKGVVEEFE---------IG------FDS------------HS----------------- :  32
RPS4 SAMI    : ---------------------------------------------------------------------------------------------------- :   -
YP 006460178 : ---------------------------------------------------------------------------------------------------- :   -
YP 005090421 : ---------------------------------------------------------------------------------------------------- :   -
YP 173472    : ---------------------------------------------------------------------------------------------------- :   -

                        *       220         *       240         *       260         *       280         *       300
XP 016500822 : AKAGGGDPQSLSSYQAELPSYIIHLPASSGGGSIPVIRDVAKKTDSTRYALPALRFKTCRLLSGNVWNRELTIIQRRILRRLRNKKRSIKRKIYSRENLN : 300
YP 009241446 : ----------------------------------------------MRYALPALRFKTCRLLSGNVWNRELTIIQRRILRRLRNKKRSIKRKIYSRENLN :  54
YP 009121977 : ----------------------------------------------MRYALPALRFKTCRLLSGNVWNRELTIIQRRILRRLRNKKRSIKRKIYSRENLN :  54
XP 011077036 : --------------------------------------MWRKRLIQRDMXLPALRFKTCHLLSGNVWNKELTIIQRRILQRLRNKKRSIKRKIYSRENLN :  62
XP 010314945 : -------PKKLLDRSSTL--YICP-PAAEAETELPLIRDVAKKTDSTRYALPALRFKTCRLLSGNVWNRELTIIQRRILGRLRNKKRSIKRKIYSRENLN : 155
YP 009049648 : --------------------------------------------------MPALRFKTCRLLSGNVWNRELTIIQRRILGRLRNKKRSIKRKIYSRENLN :  50
YP 009041168 : --------------------P-----------------DVAKKTDSTRYNVPALRFKTCRLLSGNVWKRELTIIQRRILQRLRNKKRSIKRKIYSRENLN :  95
RPS4 SAMI    : --------------------------------------------------MPALRFKTCRLLSGNVWNRELTIIQRRILKRLRNKKRSIKRKIYSRKNLN :  50
YP 006460178 : ----------------------------------------------MRYALPALRFKTCRLLSGNVWNRELTIIQRRILQRLRNKKRSIKRKIYSRENLN :  54
YP 005090421 : --------------------------------------------------MPALRFKTCRLLLGNVWNRELTIIQRRILKRLRNKKRSIKRKIYSRKNLN :  50
YP 173472    : ----------------------------------------------TRYALPALRFKTCRLLSGNVWNRELTIIQRRILRRLRNKKRSIKRKIYSRENLN :  54
                                                                 6PALRFKTCrLLsGNVWn4ELTIIQRRIL RLRNKKRSIKRKIYSReNLN
```

```
                     *     320        *     340        *     360        *     380        *     400
XP 016500822 : SYIQSQTTRKLSLFYGDLPITEMHRGRERTSYIPFLLNPETRSDVIPVRLHFCETIPQARQPISHRRVCVNNGMVNITHFKLSHGDIISFQENDARTRGE : 400
YP 009241446 : SYIQSQTTRKLSLFYGDLPITEMHRGRERTSYIPFLLNPETRSDVIPVRLHFCETIPQARQPISHRRVCVNNGMVNITHFKLSHGDIISFQENDARTRGE : 154
YP 009121977 : SYIQSQTTRKLSLFYGDLPITEMHRGRERTSYIPFLLNPETRSDVIPVRLHFCETIPQARQPISHRRVCVNNGMVNITHFKLSHGDIISFQENDARTRGE : 154
XP 011077036 : SYIQSQTTRKLSLFHGDLPITEMHRRKR-SYIPFLLNPETRSDVIPVRLHFRETIPQARQPISHRRVCVNNIMVNIIHFKVSHGDIISFQENDARTRGE : 161
XP 010314945 : SYIQSQTTRKLSLFYGDLPITEMHRGRERTSYIPFLLNPETRSDVIPVRLHFCETIPQARQPISHRRVCVNNGMVNITHFKLSHGDIISFQENDARTRGE : 255
YP 009049648 : SYIQSQTTRKLSLFYGDLPITEMHRGRERTSYIPFLLNPETRSDVIPVRLHFCETIPQARQPISHRRVCVNNGMVNITHFKLSHGDIISFQENDARTRGE : 150
YP 009041168 : SYIQSQTTRKLSLFHGDLPITEMHRRTERTSYIPFLLNPETRSDVIPVRLHFSETIPQARQPISHRRVCVNNGMVSITHFKVSHGDIISFQENDARTRGE : 195
RPS4 SAMI     : SYIQSQTTRKLSLFHGDLPITEMHRRKR-SYIPFLLNPETRSDVIPVRLHFRETIPQARQPISHRRVCVNHRMVNIIHFKVSHGDIISFQENDARTRGE : 149
YP 006460178 : SYIQSQTTRKLSLFHGDLPITEMHRRKR-SYIPFLLNPETRSDVIPVRLHFRETIPQARQPISHRRVCVNNRMVNIIHFKVSHSDIISFQQNDARTRGE : 153
YP 005090421 : SYIQSQTTRKLSLFHRDLPITEMHRRKR-SYIPFLLNPETRSDVIPVRLHFRETIPQARQTISHRRVCVNNRMVNIIHFKVSHGDIISFQENDART--- : 146
YP 173472    : SYIQSQTTRKLSLFYGDLPITEMHRGRERTSYIPFLLNPETRSDVIPVRLHFCETIPQARQPISHRRVCVNNGMVNITHFKLSHGDIISFQENDARTRGE : 154
               SYIQSQTTRKLSLF gDLPITEMHR r R SYIPFLLNPETRSDVIPVRLHF ETIPQARQpISHRRVCVNn MVnI HFK6SHgDIISFQ2NDARTrge

                     *     420        *     440        *     460        *     480        *     500
XP 016500822 : EIKRSFYIEISVEKIIGKFLDHP---WRRTKTEWFRLLKTKRGCRLLLKSRFLQQLRSSMQEEDLERTKKFGSEKVCLGSSFAEHNRMKRNLYHFKSLFL : 497
YP 009241446 : EIKRSFYIEISVEKIIGKFLDHP---WRRTKTEWFRLLKTKRGCRLLLKSRFLQQLRSSMQEEDLERTKKFGSEKVCLGSSFAEHNRMKRNLYHFKSLFL : 251
YP 009121977 : EIKRSFYIEISVEKIIGKFLDHP---WRRTKTEWFRLLKTKRGCRLLLKSRFLQQLRSSMQEEDLERTKKFGSEKVCLGSSFAEHNRMKRNLYHFKSLFL : 251
XP 011077036 : EIRRSFYIEISVEKFIGKFPDRPVRMWRRTKTEWFRKLKTKKECCLLLKSRFLQQLRSSMQEED---RKKFGSKKVCLGSYFDEHKRMKRNLYHFKSLFL : 258
XP 010314945 : EIKRSFYIEISVEKIIGKFLDHP---WRRTKTEWFRLLKTKRGCRLLLKSRFLQQLRSSMQEEDLERTKKFGSEKVCLGSSFAEHNRMKRNFYHFKSLFL : 352
YP 009049648 : EIKRSFYIEISVEKIIGKFLDHP---WRRTKTEWFRLLKTKRGCRLLLKSRFLQQLRSSMQEEDLERTKKFGSEKVCLGSSFAEHNRMKRNLYHFKSLFL : 247
YP 009041168 : EIRRFFYIEISIEKIIGKFLDHPIRMWRRTKTEWFRLLKTKRGCRLLLKSRFLQQLRSYMQEEDLERTKKFGSEKVCLGSSFAEHNRMKRNLYHFKSLFL : 295
RPS4 SAMI     : EIRRSFYIEISVEKFIGKFLDRPVRMWRRTKTKWFRKLKTKKECRLLLKSKFLQQLRSSMQEED---TKKFGSKKVCLGSYFDEHNRMKRNLYHFKSLFL : 246
YP 006460178 : EIRRSFYIEISVEKCIGKFLDRPVRMWRRNKTEWFRKLKTKKKCRLLLKSQFLQQLRSSMQEED---TKKFGSKKVCLGSYFDEHNRMKRNLYHFKCLFL : 250
YP 005090421 : ------------EKIIGKFLDRPVRMWRRTKTEWFRKFKTLKGCRLLLKSQFLQQLRSSMQEED---TKKFGSKKVCLGSYFDEHNRMKRNLYHFKSLFL : 231
YP 173472    : EIKRSFYIEISVEKIIGKFLDHP---WRRTKTEWFRLLKTKRGCRLLLKSRFLQQLRSSMQEEDLERTKKFGSEKVCLGSSFAEHNRMKRNLYHFKSLFL : 251
               ei rsfyieis EK IGKFlD P   WRRtKTeWFR lKTk4 CrLLLKS FLQQLRSsMQEED   tKKFGS KVCLGS F EHnRMKRNlYHFKsLFL

                     *     520        *     540        *     560        *     580        *     600
XP 016500822 : SKRRNEKNR--NIPTRTRSPIVYNSSLYSNSTYCSASPHQFTKKI-----KIKRIELPTHYSEVNHRTPKAVVSYGPNIGHIPHDIRLKDPNLLLRSGKG : 590
YP 009241446 : SKRRNEKNR--NIPTRTRSPIVYNSSLYSNSTYCSASPHQFTKKI-----KIKRIELPTHYSEVNHRTPKAVVSYGPNIGHIPHDIRLKDPNLLLRSGKG : 344
YP 009121977 : SKRRNEKNR--NIPTRTRSPIVYNSSLYSNSTYCSASPHQFTKKI-----KIKRIELPTHYSEVNHRTPKAVVYYGPNIGHIPHDIRLKDPNLLLRSGKG : 344
XP 011077036 : SKRRNEKNR--NIPTQTRNPIVYNSSLYSNSTYCSASPHQFTMKR-----KIKRIELPTHYSEVNHRTPKAVVSYGPNIGHIPHDIRLKDPNLLLRSGNE : 351
XP 010314945 : SKRRNEKNR--NIPTRTRSPIVYNSSLYSNSTYCSAPPHQFTKKI-----KIKRIELPTHYSEVNHRTPKAVVSYGPNIGHIPHDIRLKDPNLLLRSGKG : 445
YP 009049648 : SKRRNEKNR--NIPTRTRSPIVYNSSLYSNSTYCSASPHQFTKKI-----KIKRIELPTHYSEVNHRTPKAVVSYGPNIGHIPHDIRLKDPNLLLRSGKG : 340
YP 009041168 : SKRRNDKNL--NIPTRTRSPIVYNSSLYSNSTYCSASPHQFTMKR-----KIKRIELPTHYSEVNHRTPKAVVSYGPNIGHIPHDIRLKDPNLLLRSGNG : 388
RPS4 SAMI     : SKRRNEKNRNMNIPTQTRNPIVYNSSLYSNSTYCSASPHQFTMNRKRKNRKIKRIELPTHYSEVNHRTPKAVVSYGPNIGHIPHDIRLKDPNLLLRSGNE : 346
YP 006460178 : SKRRNEKNR--NIPTQTINPIVYNSSLYSNSPYCSASPHQFTMKRKRKKRKIKRIELPTHYSEVNHRTPKAVVSYGPNIGHIPHDIRLKDPNLLLRSGNE : 348
YP 005090421 : SKRRNEKNK--NIPTRTRNPIVYNSSLYSNSTYCSASPHQFTMKR-----KIKRIELPTHYSEVNHRTPKAVVFYGPNIGHIPHDIRLKDPNLLLRSGNE : 324
YP 173472    : SKRRNEKNR--NIPTRTRSPIVYNSSLYSNSTYCSASPHQFTKKI-----KIKRIELPTHYSEVNHRTPKAVVSYGPNIGHIPHDIRLKDPNLLLRSGKG : 344
               SKRRNeKN   NIPT Tr PIVYNSSLYSNStYCSAsPHQFT k      KIKRIELPTHYSEVNHRTPKAVVsYGPNIGHIPHDIRLKDPNLLLRSG
```

XP 016500822	:	RGQNI	:	595
YP 009241446	:	RGQNI	:	349
YP 009121977	:	RGQNI	:	349
XP 011077036	:	RGQNI	:	356
XP 010314945	:	RGQNI	:	450
YP 009049648	:	RGQNI	:	345
YP 009041168	:	RGQNI	:	393
RPS4 SAMI	:	RGQNI	:	351
YP 006460178	:	RGQNI	:	353
YP 005090421	:	RGQNI	:	329
YP 173472	:	RGQNI	:	349
		RGQNI		

Figure S3.37 The amino acid sequence allignment of RPS7 in mitogenome of *S. miltiorrhiza* and the corresponding homologs in other species.

```
                          *        20         *        40         *        60         *        80         *       100
WP 064723896 : MSRRGTAEEKTAKSDPIYRNRLVNMLVNRILKHGKKSLAYQIIYRAMKKIQQKTETNPLSVLRQAIRGVTPDIAVKARRVGGSTHQVPIEIGSTQGKALA : 100
YP 009164604 : MSRRGTAEEKTAKSDPIYRNRLVNMLVNRILKHGKKSLAYQIIYRAMKKIQQKTETNPLSVLRQAIRGVTPDIAVKARRVGGSTHQVPIEIGSTQGKALA : 100
YP 009144561 : MSRRGTAEEKTAKSDPIYRNRLVNMLVNRILKHGKKSLAYQIIYRAMKKMQQKTETNPLSVLRQAIRGVTPDIAVKARRVGGSTHQVPIEIGSTQGKALA : 100
YP 009108540 : MSRRGTAEEKTAKSDPIYRNRLVNMLVNRILKHGKKSLAYQILYRAMKKIQQKTETNPLSVLRQAIRGVTPDIAVKARRVGGSTHQVPIEIGSTQGKALA : 100
YP 009108267 : MSRRGTAEEKTAKSDPIYRNRLVNMLVNRILKHGKKSLAYQIIYRAMKKIQQKTETNPLSILRQAIRGVTPDIAVKARRVGGSTHQVPIEIGSTQGKALA : 100
YP 009108472 : MSRRGTAEKKTAKSDPIYRNRLVNMLVNRILKHGKKSLAYQIIYRAMKKIQQKTETNPLSVLRQAIRGVTPDIAVKARRVGGSTHQVPIEIGSTQGKALA : 100
YP 008964092 : MSRRGTAEEKTAKSDPIYRNRLVNMLVNRILKHGKKSLAYQIIYRAMKKIQQKTETNPLSVLRQAIRGVTPDIAVKARRVGGSTHQVPIEIGSTQGKALA : 100
YP 008592535 : MSRRGTAEEKTAKSDPIYRNRLVNMLVNRILKHGKKSLAYRIIYRAMKKIQQKTETNPLSVLRQAIRGVTPDIAVKARRVGGSTHQVPIEIGSTQGKALA : 100
YP 003359404 : MSRRGTAEEKTAKSDPIYRNRLVNMLVNRILKHGKKSLAYQIIYRAMKKIQQKTETNPLSVLRQAIRGVTPDIAVKARRVGGSTHQVPIEIGSTQGKALA : 100
YP 319809    : MSRRGTAEEKTAKSDPIYRNRLVNMLVNRILKHGKKSLAYQIIYRALKKIQQKTETNPLSVLRQAIRGVTPDIAVKSRRVGGSTHQVPIEIGSTQGKALA : 100
RPS7 SAMI    : MSRRGTAEEKTAKSDPIYRNRLVNMLVNRILKHGKKSLAYQIIYRAMKKIQQKTETNPLSVLRQAIRGVTPDIAVKARRVGGSTHQVPIEIGSTQGKALA : 100
               MSRRGTAEeKTAKSDPIYRNRLVNMLVNRILKHGKKSLAYqI6YRA6KK6QQKTETNPLS6LRQAIRGVTPDIAVKaRRVGGSTHQVPIEIGSTQGKALA

                          *       120         *       140         *
WP 064723896 : IRWLLGASRKRPGRNMAFKLSSELVDAAKGSGDAIRKKEETHRMAEANRAFAHFR : 155
YP 009164604 : IRWLLAASRKRPGRDMAFKLSSELVDAAKGSGDAIRKKEETHKMAEANRAFAHFR : 155
YP 009144561 : IRWLLAASRKRPGRNMAFKLSSELVDAAKGSGDAIRKKEETHKMAEANRAFAHFR : 155
YP 009108540 : VRWLLAASRKRPGRNMAFKLSSELVDAAKGSGDAIRKKEETHKMAEANRAFAHFR : 155
YP 009108267 : IRWLLAASQKRPGRNMAFKLSSELVDAAKGSGDAIRKKEETHKMAEANRAFAHFR : 155
YP 009108472 : VRWLLAASRKRPGRNMAFKLSSELVDAAKGSGDAIRKKEETHKMAEANRAFAHFR : 155
YP 008964092 : IRWLLAASRKRPGRNMAFKLSSELVDAANGSGDAIRKKEETHKMAEANRAFAHFR : 155
YP 008592535 : IRWLLAASRKRPGRNMAFKLSSELVDAAKGSGDAIRKKEETHKMAEANRAFAHFR : 155
YP 003359404 : IRWLLAASRKRPGRNMAFKLSSELVDAAKGSGDAIRKKEETHKMAEANRAFAHFR : 155
YP 319809    : IRWLLAASRKRPGRNMAFKLSSELVDAAKGSGDAIRKKEETHKMAEANRAFAHFR : 155
RPS7 SAMI    : IRWLLAASRKRPGRNMAFKLSSELVDAAKGSGDAIRKKEETHKMAEANRAFAHFR : 155
               6RWLLaASrKRPGR1MAFKLSSELVDAAkGSGDAIRKKEETH4MAEANRAFAHFR
```

Figure S3.38 The amino acid sequence allignment of RPS10 in mitogenome of *S. miltiorrhiza* and the corresponding homologs in other species

```
                         *        20         *        40         *        60         *        80         *       100
YP 009270668 : --------------------MTTKIRIVIRSFDHPFLEN-HFWGLPPYTRKIGLPESRVLYTVLRSPHIDKKSREQFEMEIKKQFLVIKTETHELRKKFF :  79
YP 009237631 : --------------------MTTKIRIVIRSFDHPFLEN-HFGGLPPYTRKIGLPESRVLYTVLRSPHIDKKSREQFEMEIKKKYLVIKTEKHELRKKFF :  79
XP 015076105 : -MRQRRALRRVSQKERPPKVTTTKIGIVIRSFDDPLLEN-HFWGLPPYTRKIGLPESRVLYAVLRSPHIDKKSREQFFMKIKKEFLVIKTERHELRKKFI :  98
YP 009121942 : --------------------MTTKIGIVIRSFDHPFFEN-PFWGLPPYTRKIGLPESRVLYTVLRSPHIDKKSREQFFMKIKKEFLVIKTERHELRKKFF :  79
XP 010646576 : MLLINRTGRGVSQKERPPKVTTTKIRIVIRSFDHPFFEN-HFWGLPPYTRKIGLPESXVLYTVLRSPHIDKKSREQFEMEIKKQFLVIKTETHELRKKFF :  99
YP 009045760 : --------------------MTTKIRIVIRSFDHPFMEKNHFLGLPPYTRKIGLPESRVLYTVLRSPHIDKKSREQFEMEIKKQFLVIKTERHELRKKFF :  80
YP 009041185 : --------------------MTTKIGIVIRSFDHPFLEN-NFLGLPPYTRKIGLPESRVLYTVLRSPHIDKKSREQFEMEIKKQFLVIKTERHELRKKFF :  79
RPS10 SAMI   : --------------------MTTKIGIVMKSFDHPFLEN-HFLGLPPYTRQIGLPESRVLYTVLRSPHIDKKSREQFEMKIKKQFLVIKTERHELRKKFF :  79
YP 004222833 : --------------------MTTKIRIVIRSFDHPFLEN-HFGGLQPYTRKIGLPESRVLYTVLRSPHIDKKSREQFEMEIKKKFLIIKTEKHELRKKFF :  79
YP 002608378 : --------------------MTTKIRIVIRSFDHPFFEN-HFWGLPPYTRKIGLPESRVLYTVLRSPHIDKKSREQFEMEIKKQFLVIKTETHELRKKFF :  79
YP 173396    : --------------------MTTKIGIVIRSFDHPFFEN-HFWGLPPYTRKIGLPESRVLYTVLRSPHIDKKSREQFYMKIKKEFLVIKTERHELRKKFF :  79
                                    mTTKI IV64SFDhPf En hF GLpPYTRkIGLPESrVLYtVLRSPHIDKKSREQF M IKK 5L6IKTE HELRKKFf

                         *       120         *       140         *       160
YP 009270668 : RLKRQRIFGAQYEILFSCKTRSDKGKLQRLLRSKILALT--LS------------------ : 120
YP 009237631 : RLKRQRLFGAQYEILFYCKTRSDKGKLQRLLRSKILALT--LS------------------ : 120
XP 015076105 : RLKRRRIFGAQYEILFSCKTRSDKGKLQRLLRNKILALT--LS------------------ : 139
YP 009121942 : RLKRQRIFGAQYEILFSCKTRSDKGKLQRLLRNKILALT--LS------------------ : 120
XP 010646576 : RLKRRRIFGAQYEILFYCKTRSDKGKLQRLLRSKILALT--LS------------------ : 140
YP 009045760 : RLKRQRIFGAQYEILFSCKTRSDKEKVQRLLRSQILALT--VS------------------ : 121
YP 009041185 : RLKRQRIFGAQYEILFSCKTRSDKGKLQRLLRSKILCLDPFLNQNRGRVRGKGDHNVPSIK : 140
RPS10 SAMI   : RLKRRRIFGAQYEILFSCKTRSDKGKLQRLLRSKILALT--LS------------------ : 120
YP 004222833 : RLKRQRIFGAQYEILFSCKTRSDKGKLQRLLRSKILALT--LS------------------ : 120
YP 002608378 : RLKRQRIFGAQYEILFYCKTRSDKGKLQRLLRSKILALT--LS------------------ : 120
YP 173396    : RLKRQRIFGAQYEILFSCKTRSDKGKLQRLLRNKILALT--LS------------------ : 120
               RLKR R6FGAQYEILF CKTRSDKgK6QRLLR kILaLt  6s
```

Figure S3.39 The amino acid sequence allignment of RPS12 in mitogenome of *S. miltiorrhiz*a and the corresponding homologs in other species

```
                        *        20         *        40         *        60         *        80         *       100
YP 009173845 : MPTLNQLIRHGREEKRRTDRTRALDQCPQKQGVCLRVSTRTPKKPNSALRKIVKVRLSNRHDIFAHIPGEGHNLQEHSMVLIRGGRVKDSPGVKSHCIRG : 100
YP 009153925 : MPTLNQLIRHGREEKRRTDRTRASDQCPQKQGVCPRVLTRTPKKPNSALRKIAKVRLSNRHDIFAYIPGEGHNSQEHSMVLIRGGRVKDLPGVKSHCIRG : 100
YP 009045786 : MPTVNQLIRHGREEKRRTDRTRASDQCPQKQGVCPRVSTRTPKKPNSAPRKIAKVRLSNRHDIFAHIPGEGHNSQEHSMVLIRGGRVKDSPGVKSHCIRG : 100
YP 009041158 : MPTLNQLIRHGREEKRRTDRTRALDQCPQKQGVCPRVSTRTPKKPNSALRKIAKVRLSNRHDIFAHIPGEGHNLQEHSTVLIRGGRVKDSPGVKFHCIRG : 100
RPS12 SAMI   : MPTLNQLIRHGREEKRRTDRTRALDQCPQKEGVCPRVSTRTPKKPNSALRKIAKVQLSNKHDIFAHIPGEGHNSQEHSKVLIRGGRVKDLPGVKSHCIRG : 100
YP 008802516 : MPTLNQLIRHGREEKRRTDRTRALDQCPQKQGVCLRVSTRTPKKPNSALRKIAKVRLSNRHDIFAYIPGEGHNLQEHSTVLIRGGRVKDLPGVKFHCIRG : 100
YP 007516865 : MPTLNQLIRHGREEKRRTDRTRASDQCPQKQGVRPRVSTRTPKKPNSAPRKIAKVRLSNRHDIFAHIPGEGHNSQEHSMVLIRGGRVKDLPGVKSHCIRG : 100
YP 006460182 : MPTLNQLIRHGREEKRRTDRTRALDQCPQKEGVCPRVSTRTPKKPNSALRKIAKVQLSNKHDIFAHIPGEGHNSQEHSKVLIRGGRVKDLPGVKSHCIRG : 100
YP 005090426 : MPTFNQLIRHGREEKRRTDRTRALDQCPQKEGVCPRVSTRTPKKPNSALRKIAKVQLSNKHAIFAHIPGEGHNSQEHSKVLIRGGRVKDLPGVKSHCIRG : 100
YP 005090462 : MPTLNQLIRHGREEKRRTDRTRALDQCPQKQGVCLRVSTRTPKKPNSALRKIAKVRLSNRHDIFAYIPGEGHNLQEHSMVLIRGGRVKDLPGVKFHCIRG : 100
NP 064075    : MPTINQLIRHGREEKRRTDRTRALDQCPQKQGVCLRVSTRTPKKPNSALRKIAKVRLSNRHDIFAYIPGEGHNLQEHSMVLIRGGRVKDLPGVKFHCIRG : 100
               MPT NQLIRHGREEKRRTDRTRA DQCPQK2GVc RVsTRTPKKPNSAlRKIaKV LSN4HdIFA IPGEGHN QEHS VLIRGGRVKD PGVK HCIRG

                        *       120
YP 009173845 : VKDLLGIPDRRRGRSKYGAEKPKSI : 125
YP 009153925 : VKDLLGIPDRRRGRSKYGAEKPK-- : 123
YP 009045786 : VKDLLGIPDRRRGRSKYGAEKPKSI : 125
YP 009041158 : VKDLLGIPDRRRGRSKYGAEKPKSI : 125
RPS12 SAMI   : VRDLLGIPDRRRGRSKYGAEKPKSI : 125
YP 008802516 : VKDLMGIPDRRRGRSKYGAEKPKSI : 125
YP 007516865 : VKDLLGIPDRRRGRSKYGAEKPKSI : 125
YP 006460182 : VRDLLGIPDRRRGRSKYGAEKPKSR : 125
YP 005090426 : VRDLLGIPDRRKGRSKYGAEKPKSI : 125
YP 005090462 : VKDLLGIPDRRRGRSKYGAEKPKSI : 125
NP 064075    : VKDLLGIPDRRRGRSKYGAEKPKSI : 125
               V4DL6GIPDRR4GRSKYGAEKPKsi
```

Figure S3.40 The amino acid sequence allignment of RPS13 in mitogenome of *S. miltiorrhiza* and the corresponding homologs in other species

```
                        *        20         *        40         *        60         *        80         *       100
YP 009121969 : MFYISGARLVGDEQVRIASTKIDGIGPKKAIQVRYRLGISGNIKIKELTKYQIDQIEQMIGQDHVVHWELKRGERADIERLISISCYRGIRYQDGSPLRG : 100
YP 009049641 : MLYISGARLVGDEQVRIASTKIDGIGPKKAIQVRYRLGISGNIKIKELTKYQIDQIEQMIGQDHVVHWELKRGERADIERLISISCYRGIRHQDGSPLRG : 100
YP 009041173 : MLYISGARSVADEQVRIASTKIDGIGPKKAIQVRYRLGISGNIKINELTKYQIDQIEQMIGQDHVVHWELKRGERADIERLISISCYRGIRHQDGSPVRG : 100
YP 008999564 : MLYISGARSVADEQVRIASTKIDGIGPKKAILVRYRLGISGNIKIKELTKYQIDQIEQMIGQDHVVHWELKRGERADIERLISISCYRGIRHQDGSPLRG : 100
RPS13 SAMI   : MLYISGARLVADKQVRIASTKIYGIGLKKAIQVRYRLGISGNIKIKELTKYQIDQIEQMIGQDHVVHWELKRGERADIERLISISCYRGIRHQDGSPLRG : 100
YP 008964111 : MFYISGARLVSDKQVRIASTQILGIGLKKAIQVRYRLGISGNIKIKELTKYQIGQIEQMIGQDHVVHWELKRGERADIQRLISISCYRGIRHQDGSPLRG : 100
YP 006460167 : MLYISGARLVADKQVRIASTKIYGIGLQKAIQVRYRLGISGNIKIKELTKYQIDQIEQMIGQDHVVHWELKRGERADIERFISISCYRGIRHQDGSPLRG : 100
YP 006291804 : MLYISGARSVADKQVRIASTKMYGIGPKKAIQVRYRLGISGNIKIKELTKYQIDQMEQMIGQDHVVHWELKRGERADIERFISISCYRGIRHQDGLPLRG : 100
YP 005090402 : MLYISGARLVADKQVRIASTKIYGIGLKKAIQVRYRLGISGNIKIKELTKYQIDQIEQMIGQDHVVHWELKRGERADIERFISISCYRGIRHQDGSPLRG : 100
YP 002608196 : MSYISGARLVANEQVRIASTKIDGIGPKKAIQVRYRLGISGNIKIKELTKYQIDQIEQMIGQDHVVHWELKRGERADIERFISISCYRGIRHQDGSPLRG : 100
YP 173403    : MLYISGARLVGDEQVRIASTKIDGIGPKKAIQVRYRLGISGNIKIKELTKYQIDQIEQMIGQDHVVHWELKRGERADIERLISISCYRGIRHQDGSPLRG : 100
               M YISGAR V 1 QVRIASTk6 GIG kKAIqVRYRLGISGNIKIkELTKYQIdQ6EQMIGQDHVVHWELKRGERADI2R ISISCYRGIRhQDGsP6RG

                        *
YP 009121969 : QRTHTNARTCRKLIRK : 116
YP 009049641 : QRTHTNARTCRKRIRK : 116
YP 009041173 : QRTHTNARTCRKLIRK : 116
YP 008999564 : QRTHTNARTCRKQIRK : 116
RPS13 SAMI   : QRTHTNARTCRKQIRK : 116
YP 008964111 : QRTHTNARTCRKQIRK : 116
YP 006460167 : QRTHTNARTCRKQIRK : 116
YP 006291804 : QRTHTNARTCRKQIRK : 116
YP 005090402 : QRTHTNARTCRKQIRK : 116
YP 002608196 : QRTHTNARTCRKLIRK : 116
YP 173403    : QRTHTNARTCRKLIRK : 116
               QRTHTNARTCRK IRK
```

Figure S3.41 The amino acid sequence allignment of RPS14 in mitogenome of *S. miltiorrhiz*a and the corresponding homologs in other species

```
                               *        20         *        40         *        60         *        80         *       100
YP 009270694 : ---------------------MSEKRNIRDHKRRLLAAKYELRRKLYKAFC--KDPDLPSDMRDKHRYKLSKLPRNSSFARVRNRCIFTGRPRSVYEFFRI :  78
XP 008790687 : -------------MREKLSKMSEKRNIRDHKRRLLAAKYELRRKLYKAFC--KDPDLPSDMRDKNRYKLSKLPRNSSFARVRNRCISTGRPRSVVEFFRI :  85
XP 016702844 : MRQSHRGAAFCKKMRGKLNKMSEKXNIRDHKRRFLAAKYELRRKLYKAFC--KDPDLPSDMRDKHRYKLSKLPRNSSFARVRNRCISTGRPRSVYEFFXI :  98
XP 010556267 : MRLYHRGAAFCKKMRGKLSKMSEKRNIRDHKRRLLAAKFELRRKLYKAFC--KDPDLPSDMRDKHRYKLSKLPRNSSFARVRNRCISTGRPRSVSGFFRI :  98
YP 009045790 : ---------------------MSEKRNIRDHKRRLLAAKFELRRKLYKAFC--KDPDLPSEMRDKHRYKLSKLPRNSSFARVRNRCISTGRPRSVYEFFRI :  78
RPS14 SAMI     : ---------------------MSEKRNIRDHKRRLLAAKYELRRKLYKAFCFCKDPDLP---RDKHRYKLSKLPRNSSFARVRNRCISTGRPRSVYKFFRI :  77
YP 007905721 : ---------------------MSEKRNIRDHKRRLLAAKYELRRKLYKAFC--KDPDLPSDMRDKHRYKLSKLPRNSSFARVRNRCISTGRPRSVYEFFRI :  78
YP 006460159 : ---------------------MSEKRNIRDHKRRLLAAKYELRRKLYKAFC--KDPDLPSDMRDKHRYKLSKLPRNSSFARVRNRCISTGRPRSVYKFFRI :  78
YP 005090361 : -------------MREKLSKMSEKRNIRDHKRRLLAAKYELRRKLYKAFC--KDPDLPSDMRDKHRYKLSKLPRNSSFARVRNRCISTGRPRSVVEFFRI :  85
YP 002608356 : MRLYHRGAAFFKKMR--VSKMSEKLNIRDHKRRLLAAKYELRRKLYKAFC--KDPDLPSDMRDKHRYKLSKLPRNSSFARVRNRCISTGRPRSVYEFFRI :  96
YP 002608213 : ---------------------MSEKRNIRDHKRRLLAAKYELRRKLYKAFC--KDPDLPSDMRDKHRYKLSKLPRKSSFARVRNRCISTGRPRSVYEFFRI :  78
                     MSEKrNIRDHKRRlLAAK5ELRRKLYKAFC  KDPDLPsdmRDKhRYKLSKLPRnSSFARVRNRCIsTGRPRSV  FFrI

                         *       120
YP 009270694 : SRIVFRGLASRGSLMGIKKSSW : 100
XP 008790687 : SRIVFRGLASRGPLMGIKKSSW : 107
XP 016702844 : SRIVFRGLASRGPLMGIKKSSW : 120
XP 010556267 : SRIVFRGLASRGSLMGIKKSSW : 120
YP 009045790 : SRIVFRGLASRGSLMGIKKSSW : 100
RPS14 SAMI     : SRIVFR--ISRS---GIKKSSW :  94
YP 007905721 : SRIVFRGLASRGPLMGIKKSSW : 100
YP 006460159 : SRIVFRGLASRGPLMGIKKSSW : 100
YP 005090361 : SRIVFRGLASRGPLMGIKKSSW : 107
YP 002608356 : SRIVFRGLASRGPLMGIKKSSW : 118
YP 002608213 : SRIVFRGLASRGPLMGIKKSSW : 100
                SRIVFRglaSRg lmGIKKSSW
```

Figure S4 Computational pipeline for the identification of RNA editing sites.

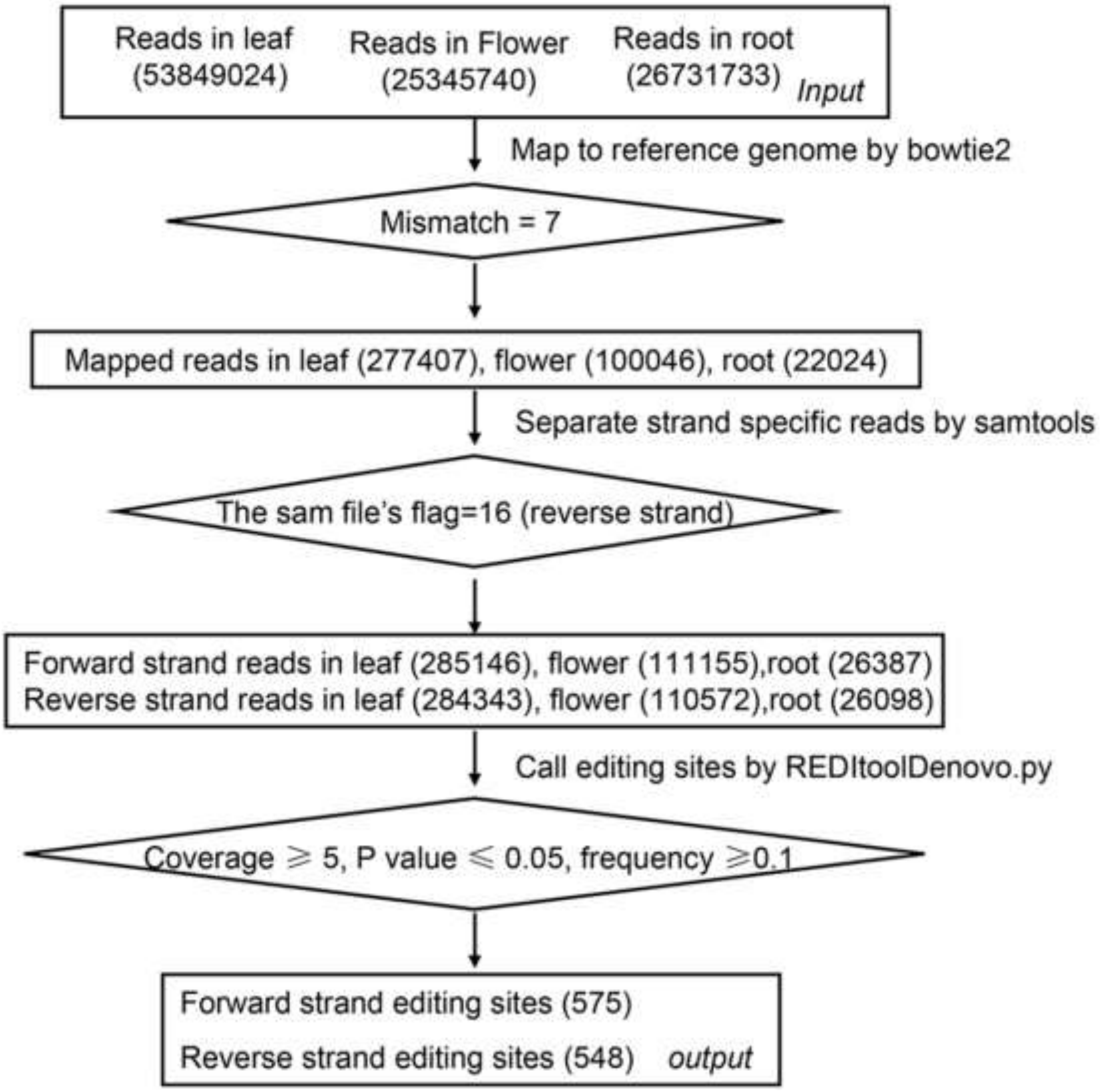

Figure S5 Venn diagram showing the RNA editing sites found across different tissues. (A) RNA editing sites found on the positive strand; (B) RNA editing sites found on the negative strand.

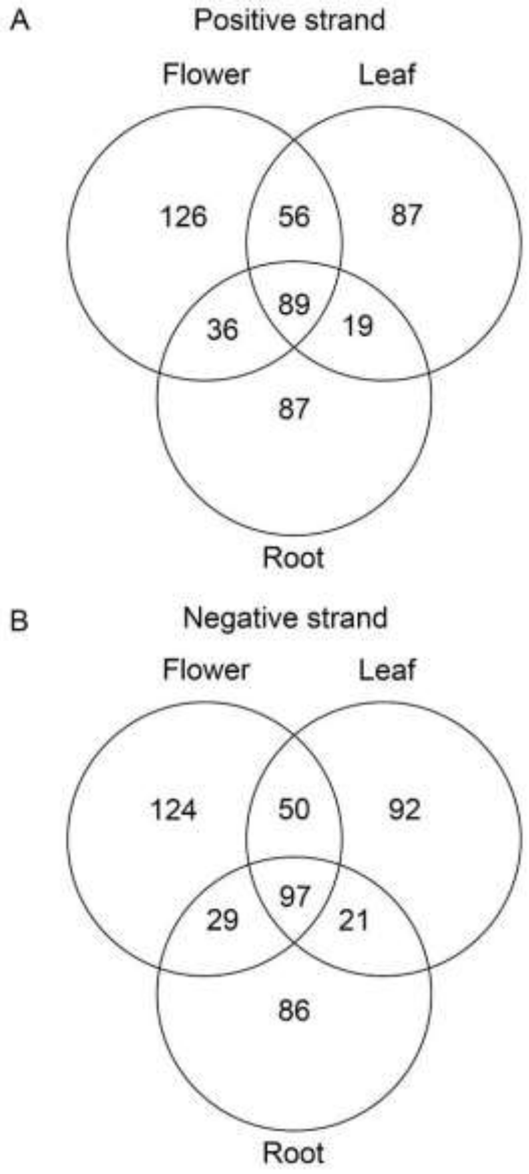

Figure S6 Distribution of "C to U" RNA editing sites at different codon positions (A) and the effects of RNA editing on the encoded amino acid (B).

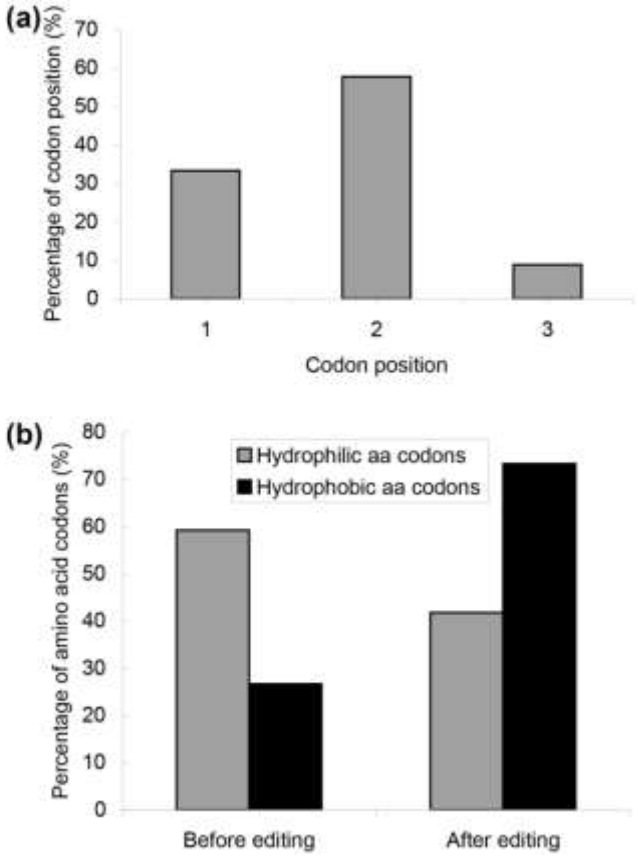

Figure S7 Potential effects of RNA editing at Rps3-1558 on the structures of the mRNA (A) and protein (B) of the *Rps3* gene.

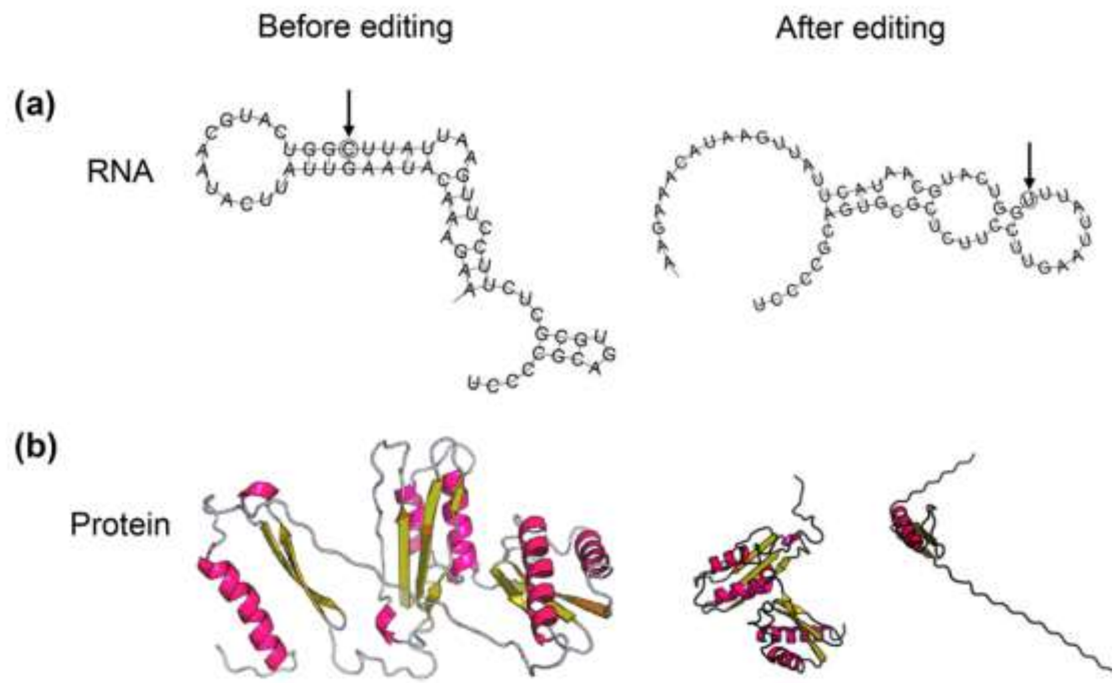

Figure S8 Venn diagram showing the RNA editing sites on the sense (A) and antisense (B) strands shared among homologous CDS from *A. thaliana*; *O. sativa*; and *S. miltiorrhiza*.

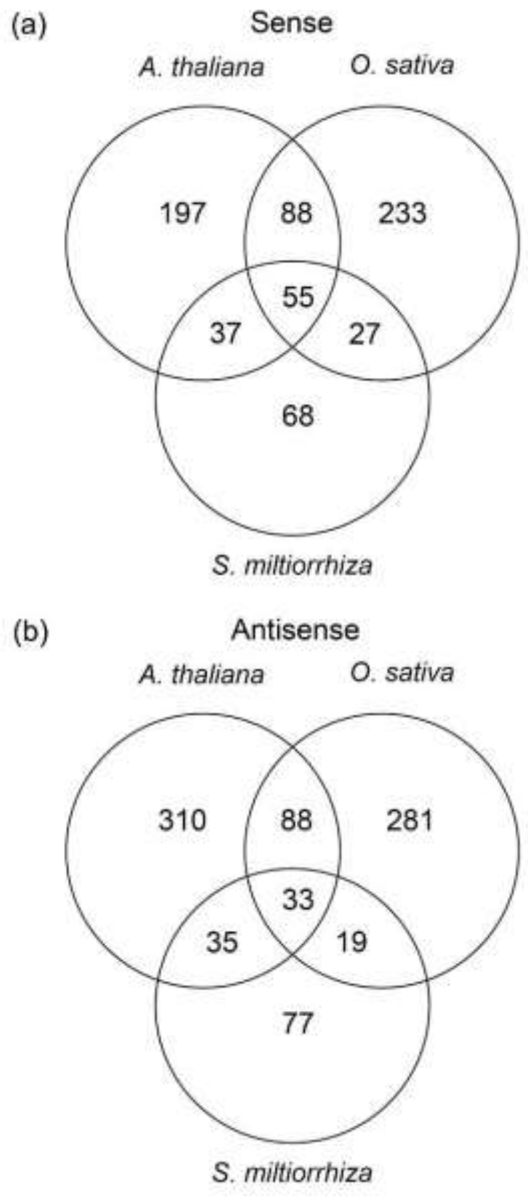

Figure S9 Venn diagram showing Symmetric RNA editing sites shared among homologous CDS from *A. thaliana*; *O. sativa*; and *S. miltiorrhiza*.

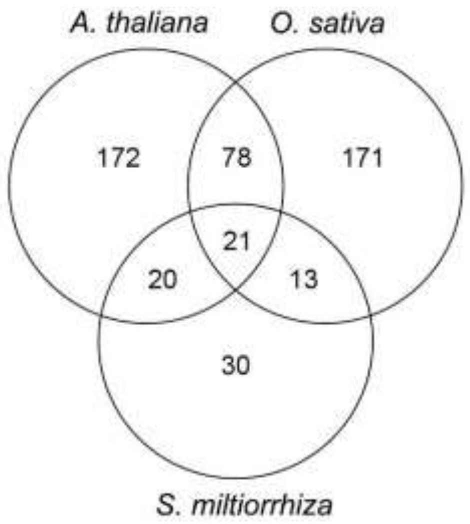

Figure S10 The sensitivities and specificities of the mapping-based method for various mismatch numbers using different mitogenomes. (a) ***A. thaliana*****; (b)** ***O. sativa*****; (c)** ***S. miltiorrhiza*****.**

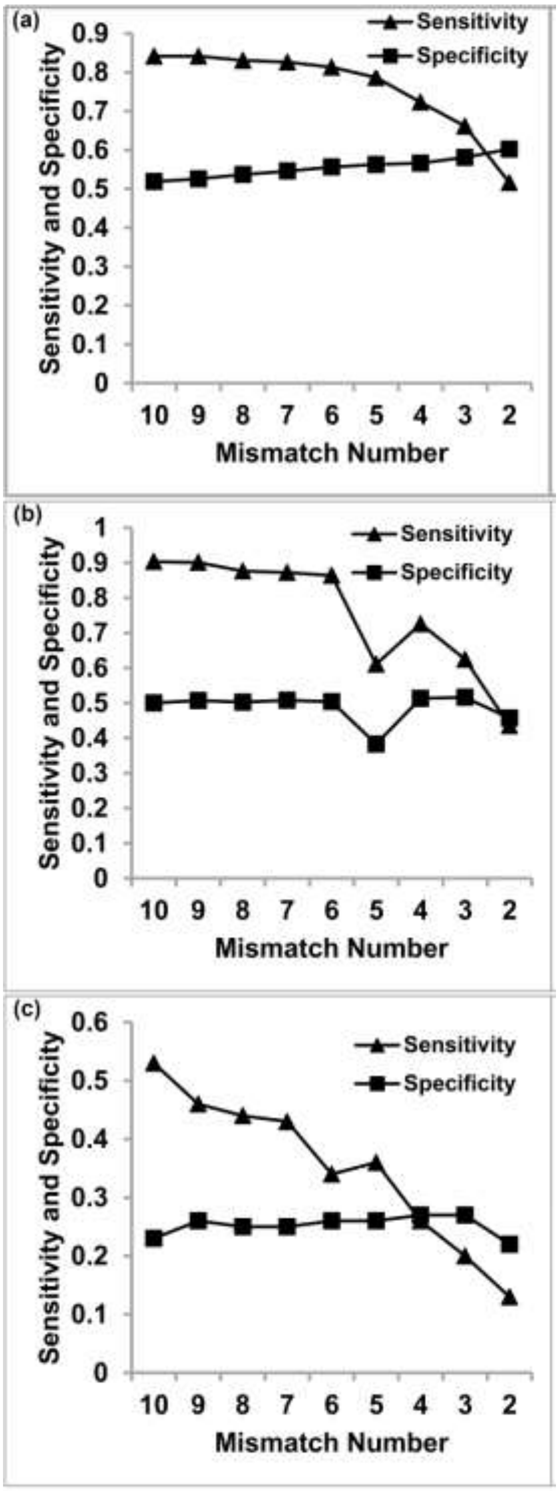

Supplementary Tables

Table S1 List of all predicted RNA editing sites on the mitogenome of *S. miltiorrhiza*.

Genomic Position	RES Name	RES Type	Coverage	Frequency	P value	RES Name	RES Type	Read Coverage at the site	RES Frequency	P value	Genomic regions	Gene Name	Orientation of the sense strand	Overlappi with SNP sites
2507	2507-F	AC	5	1	0.02381	NA	NA	NA	NA	NA	intergenic	NA	NA	
5985	matR-1845-as	AG	6	1	0.02381	NA	NA	NA	NA	NA	exon	matR	reverse	
6016	matR-1814-as	GA	7	0.86	0.014569	NA	NA	NA	NA	NA	exon	matR	reverse	
6055	matR-1775-as	GA	12	1	4.81E-06	NA	NA	NA	NA	NA	exon	matR	reverse	
6086	matR-1744-as	GA	6	1	0.007576	matR-1744	CU	8	1	0.0006993	exon	matR	reverse	
6122	matR-1708-as	GA	5	1	0.02381	matR-1708	CU	9	1	0.00020568	exon	matR	reverse	
6142	NA	NA	NA	NA	NA	matR-1688	CU	9	1	0.00020568	exon	matR	reverse	
6163	NA	NA	NA	NA	NA	matR-1667	CU	11	1	1.701e-005	exon	matR	reverse	
7787	NA	NA	NA	NA	NA	matR-43	CU	10	1	5.95E-05	exon	matR	reverse	
7798	NA	NA	NA	NA	NA	matR-32	CU	8	1	0.0006993	exon	matR	reverse	
8856	8856-F	CU	19	0.42	0.009376	NA	NA	NA	NA	NA	intergenic	NA	NA	
8979	NA	NA	NA	NA	NA	8979-R	CU	18	0.44	0.00888289	intergenic	NA	NA	
9236	mttB-728-as	GA	28	0.96	1.03E-13	NA	NA	NA	NA	NA	exon	mttB	reverse	
9269	NA	NA	NA	NA	NA	mttB-695	CU	28	0.56	2.29E-05	exon	mttB	reverse	
9277	mttB-687-as	GA	8	0.75	0.02028	mttB-687	CU	28	0.71	6.55E-08	exon	mttB	reverse	

9282	mttB-682-as	GA	8	1	0.000699	mttB-682	CU	27	0.96	3.75E-13	exon	mttB	reverse		
9333	NA	NA	NA	NA	NA	mttB-631	CU	31	0.81	1.10E-10	exon	mttB	reverse		
9339	NA	NA	NA	NA	NA	mttB-625	CU	30	0.97	7.62E-15	exon	mttB	reverse		
9371	NA	NA	NA	NA	NA	mttB-593	CU	8	0.88	0.00505051	exon	mttB	reverse		
9395	NA	NA	NA	NA	NA	mttB-569	CU	6	1	0.00757576	exon	mttB	reverse		
9542	mttB-422-as	GA	12	0.92	5.36E-05	NA	NA	NA	NA	NA	exon	mttB	reverse		
9570	mttB-394-as	GA	14	0.79	0.000171	mttB-394	CU	12	0.75	0.00137973	exon	mttB	reverse		
9573	mttB-391-as	GA	13	0.77	0.000491	mttB-391	CU	12	0.83	0.0003221	exon	mttB	reverse		
9576	mttB-388-as	GA	13	0.77	0.000491	mttB-388	CU	12	0.75	0.00137973	exon	mttB	reverse		
9582	mttB-382-as	GA	14	0.5	0.016425	NA	NA	NA	NA	NA	exon	mttB	reverse		
9603	NA	NA	NA	NA	NA	mttB-361	CU	12	0.75	0.00137973	exon	mttB	reverse		
9618	NA	NA	NA	NA	NA	mttB-346	CU	10	0.9	0.00054667	exon	mttB	reverse		
9621	NA	NA	NA	NA	NA	mttB-343	CU	12	0.75	0.00137973	exon	mttB	reverse		
10021	NA	NA	NA	NA	NA	10021-R	CU	8	1	0.0006993	intergenic	NA	NA		
11379	NA	NA	NA	NA	NA	11379-R	UC	5	1	0.02380952	intergenic	NA	NA		
19260	19260-F	CU	5	1	0.02381	NA	NA	NA	NA	NA	intergenic	NA	NA		
19318	NA	NA	NA	NA	NA	cob-1160	CU	5	1	0.02380952	exon	cob	reverse		
19394	cob-1084-as	GA	9	1	0.000206	cob-1084	CU	6	1	0.00757576	exon	cob	reverse		
19463	NA	NA	NA	NA	NA	cob-1015	CU	6	1	0.00757576	exon	cob	reverse		
19496	NA	NA	NA	NA	NA	cob-982	CU	9	0.89	0.00168655	exon	cob	reverse		
19625	cob-853-as	GA	27	1	1.44E-14	NA	NA	NA	NA	NA	exon	cob	reverse		
19910	cob-568-as	GA	7	1	0.002331	NA	NA	NA	NA	NA	exon	cob	reverse		
20120	cob-358-as	GA	7	1	0.002331	cob-358	CU	15	1	1.03E-07	exon	cob	reverse		
20153	NA	NA	NA	NA	NA	cob-325	CU	17	1	7.71E-09	exon	cob	reverse		
20180	NA	NA	NA	NA	NA	cob-298	CU	11	1	1.701e-005	exon	cob	reverse		
21156	21156-F	AG	10	1	5.95E-05	NA	NA	NA	NA	NA	intergenic	NA	NA		Yes

21188	21188-F	CU	11	1	1.7E-05	NA	NA	NA	NA	NA	intergenic	NA	NA	Yes
21451	NA	NA	NA	NA	NA	rbcL-1203	GU	129	0.1	0.00069687	exon	rbcL	reverse	Yes
21452	NA	NA	NA	NA	NA	rbcL-1202	AU	117	0.14	8.405e-005	exon	rbcL	reverse	Yes
21480	rbcL-1174-as	CU	1449	0.15	1.78E-62	NA	NA	NA	NA	NA	exon	rbcL	reverse	Yes
21688	rbcL-966-as	AG	1198	0.14	1.35E-47	NA	NA	NA	NA	NA	exon	rbcL	reverse	Yes
21883	rbcL-771-as	AG	185	0.12	3.12E-06	NA	NA	NA	NA	NA	exon	rbcL	reverse	Yes
21889	NA	NA	NA	NA	NA	rbcL-765	AU	100	0.2	4.02E-06	exon	rbcL	reverse	Yes
21905	rbcL-749-as	AC	250	0.11	5.32E-08	NA	NA	NA	NA	NA	exon	rbcL	reverse	Yes
22051	rbcL-603-as	UC	81	0.16	0.001146	NA	NA	NA	NA	NA	exon	rbcL	reverse	Yes
22123	NA	NA	NA	NA	NA	rbcL-531	AU	83	0.12	0.00458556	exon	rbcL	reverse	Yes
22201	rbcL-453-as	CU	85	0.12	0.004657	NA	NA	NA	NA	NA	exon	rbcL	reverse	Yes
22360	rbcL-294-as	GU	51	0.8	7.96E-18	NA	NA	NA	NA	NA	exon	rbcL	reverse	Yes
22382	rbcL-272-as	GA	88	0.44	2.01E-13	NA	NA	NA	NA	NA	exon	rbcL	reverse	Yes
22383	rbcL-271-as	GU	88	0.44	2.01E-13	NA	NA	NA	NA	NA	exon	rbcL	reverse	Yes
22424	rbcL-230-as	AU	103	0.1	0.004809	NA	NA	NA	NA	NA	exon	rbcL	reverse	Yes
22842	NA	NA	NA	NA	NA	22842-R	AG	12	0.58	0.01359537	intergenic	NA	NA	Yes
27235	27235-F	CU	27	0.63	2.4E-06	NA	NA	NA	NA	NA	intergenic	NA	NA	Yes
27237	27237-F	AC	26	0.58	4.43E-05	NA	NA	NA	NA	NA	intergenic	NA	NA	Yes
27294	cox2-745-as	GA	74	0.91	9.26E-33	cox2-745	CU	5	1	0.02380952	exon	cox2	reverse	Yes
27315	cox2-724-as	GA	73	0.9	3.31E-32	cox2-724	CU	28	1	3.79E-15	exon	cox2	reverse	Yes
27339	cox2-700-as	GA	27	0.59	7.64E-06	NA	NA	NA	NA	NA	exon	cox2	reverse	Yes
27340	27340-F	GA	25	0.56	5.1E-05	NA	NA	NA	NA	NA	intron	cox2	reverse	Yes
27433	NA	NA	NA	NA	NA	27433-R	UG	18	0.39	0.02037618	intron	cox2	reverse	Yes
27619	27619-F	CU	85	0.52	1.73E-15	NA	NA	NA	NA	NA	intron	cox2	reverse	
28245	28245-F	UA	25	0.54	0.000128	NA	NA	NA	NA	NA	intron	cox2	reverse	
30240	30240-F	UA	32	0.39	0.000551	NA	NA	NA	NA	NA	intron	cox2	reverse	

30241	30241-F	CU	96	0.36	3.26E-11	NA	NA	NA	NA	NA	intron	cox2	reverse
30416	NA	NA	NA	NA	NA	30416-R	CU	100	0.56	1.44E-19	intron	cox2	reverse
30484	cox2-632-as	GA	10	0.9	0.000547	cox2-632	CU	55	1	5.68E-31	exon	cox2	reverse
30559	cox2-557-as	GA	6	6	1.40E-06	cox2-557	CU	15	0.93	1.46E-06	exon	cox2	reverse
30572	cox2-544-as	GA	6	2	0.00055	cox2-544	CU	17	1	7.71E-09	exon	cox2	reverse
30640	NA	NA	NA	NA	NA	cox2-476	CU	5	1	0.02380952	exon	cox2	reverse
30673	cox2-443-as	GA	13	1	1.35E-06	NA	NA	NA	NA	NA	exon	cox2	reverse
31045	NA	NA	NA	NA	NA	cox2-71	CU	6	1	0.00757576	exon	cox2	reverse
34282	NA	NA	NA	NA	NA	34282-R	CU	37	0.41	5.34E-05	intergenic	NA	NA
35255	35255-F	GU	109	0.11	0.001303	NA	NA	NA	NA	NA	intron	nad5	forward
35301	NA	NA	NA	NA	NA	35301-R	CU	340	0.19	1.50E-19	intron	nad5	forward
35304	NA	NA	NA	NA	NA	35304-R	CU	342	0.13	3.05E-13	intron	nad5	forward
35350	NA	NA	NA	NA	NA	35350-R	AU	92	0.18	7.01E-05	intron	nad5	forward
35588	nad5-359	CU	5	1	0.02381	nad5-359-as	GA	11	1	1.701e-005	exon	nad5	forward
35603	nad5-374	CU	7	1	0.002331	nad5-374-as	GA	11	1	1.701e-005	exon	nad5	forward
35627	nad5-398	CU	6	1	0.007576	nad5-398-as	GA	9	1	0.00020568	exon	nad5	forward
35768	nad5-539	CU	13	1	1.35E-06	nad5-539-as	GA	13	0.54	0.01510297	exon	nad5	forward
35777	nad5-548	CU	6	1	0.007576	NA	NA	NA	NA	NA	exon	nad5	forward
35837	nad5-608	CU	8	1	0.000699	nad5-608-as	GA	14	0.79	0.0001705	exon	nad5	forward
35858	NA	NA	NA	NA	NA	nad5-629-as	GA	11	0.91	0.00017294	exon	nad5	forward
35905	nad5-676	CU	21	1	4.09E-11	nad5-676-as	GA	9	0.89	0.00168655	exon	nad5	forward
35942	nad5-713	CU	21	1	4.09E-11	NA	NA	NA	NA	NA	exon	nad5	forward
35954	nad5-725	CU	22	1	1.09E-11	NA	NA	NA	NA	NA	exon	nad5	forward
36064	nad5-835	CU	42	0.93	3.16E-19	nad5-835-as	GA	24	0.96	1.79E-11	exon	nad5	forward
36085	nad5-856	AU	62	0.18	0.002115	NA	NA	NA	NA	NA	exon	nad5	forward
36491	nad5-1262	GU	26	0.46	0.000412	NA	NA	NA	NA	NA	exon	nad5	forward

36539	nad5-1310	CU	33	0.64	6.53E-08	nad5-1310-as	GA	11	1	1.701e-005	exon	nad5	forward
36696	36696-F	AU	12	0.83	0.000322	NA	NA	NA	NA	NA	intergenic	NA	NA
36888	NA	NA	NA	NA	NA	36888-R	UC	32	0.34	0.00132591	intergenic	NA	NA
36941	NA	NA	NA	NA	NA	36941-R	GA	18	1	2.09E-09	intergenic	NA	NA
37682	37682-F	GA	6	1	0.007576	NA	NA	NA	NA	NA	intergenic	NA	NA
37694	37694-F	AC	5	1	0.02381	NA	NA	NA	NA	NA	intergenic	NA	NA
37721	37721-F	GA	5	1	0.02381	37721-R	CU	5	1	0.02380952	intergenic	NA	NA
37752	NA	NA	NA	NA	NA	37752-R	UA	7	1	0.002331	intergenic	NA	NA
41515	NA	NA	NA	NA	NA	41515-R	GA	7	1	0.002331	intergenic	NA	NA
41518	41518-F	AG	9	1	0.000206	41518-R	UC	16	1	2.83E-08	intergenic	NA	NA
41519	NA	NA	NA	NA	NA	41519-R	GC	21	0.38	0.01024289	intergenic	NA	NA
41520	41520-F	AC	5	1	0.02381	41520-R	UG	13	1	1.35E-06	intergenic	NA	NA
41521	41521-F	AC	6	1	0.02381	NA	NA	NA	NA	NA	intergenic	NA	NA
41522	41522-F	UG	8	1	0.000699	41522-R	AC	25	1	2.06E-13	intergenic	NA	NA
41806	NA	NA	NA	NA	NA	41806-R	GU	317	0.11	2.11E-10	intergenic	NA	NA
42970	42970-F	CG	7	0.86	0.014569	NA	NA	NA	NA	NA	intergenic	NA	NA
43785	NA	NA	NA	NA	NA	nad9-478	CU	24	0.33	0.01130447	exon	nad9	reverse
43865	NA	NA	NA	NA	NA	nad9-398	CU	12	0.83	0.0003221	exon	nad9	reverse
43895	NA	NA	NA	NA	NA	nad9-368	CU	9	1	0.00020568	exon	nad9	reverse
44061	nad9-202-as	UC	6	1	0.007576	NA	NA	NA	NA	NA	exon	nad9	reverse
44096	nad9-167-as	GA	6	1	0.007576	NA	NA	NA	NA	NA	exon	nad9	reverse
44150	nad9-113-as	GA	7	0.86	0.014569	nad9-113	CU	6	1	0.00757576	exon	nad9	reverse
44171	NA	NA	NA	NA	NA	nad9-92	CU	6	1	0.00757576	exon	nad9	reverse
44235	NA	NA	NA	NA	NA	nad9-28	AC	7	0.86	0.01456876	exon	nad9	reverse
47328	47328-F	CU	7	0.86	0.014569	NA	NA	NA	NA	NA	intergenic	NA	NA
47386	47386-F	CU	13	0.69	0.001804	NA	NA	NA	NA	NA	intergenic	NA	NA

47405	47405-F	CU	13	1	1.35E-06	NA	NA	NA	NA	NA	intergenic	NA	NA
48030	NA	NA	NA	NA	NA	48030-R	CU	7	1	0.002331	intergenic	NA	NA
49648	nad6-26	CU	12	0.92	5.36E-05	NA	NA	NA	NA	NA	exon	nad6	forward
49710	nad6-88	CU	45	0.98	1.95E-23	NA	NA	NA	NA	NA	exon	nad6	forward
49711	nad6-89	CU	43	0.98	2.79E-22	NA	NA	NA	NA	NA	exon	nad6	forward
49712	nad6-90	CU	44	0.27	0.000816	NA	NA	NA	NA	NA	exon	nad6	forward
49717	nad6-95	CU	44	0.98	7.38E-23	NA	NA	NA	NA	NA	exon	nad6	forward
49725	nad6-103	CU	41	0.98	3.96E-21	NA	NA	NA	NA	NA	exon	nad6	forward
49783	nad6-161	CU	10	0.9	0.000547	nad6-161-as	GA	46	0.98	5.15E-24	exon	nad6	forward
49791	nad6-169	CU	8	0.88	0.005051	nad6-169-as	GA	47	0.98	1.36E-24	exon	nad6	forward
49813	nad6-191	CU	8	0.88	0.005051	nad6-191-as	GA	49	1	1.96E-27	exon	nad6	forward
50029	nad6-407	AU	81	0.22	1.47E-05	NA	NA	NA	NA	NA	exon	nad6	forward
50085	nad6-463	CU	157	0.99	1.64E-89	NA	NA	NA	NA	NA	exon	nad6	forward
50198	NA	NA	NA	NA	NA	nad6-576-as	CU	133	0.16	4.86E-06	exon	nad6	forward
53657	NA	NA	NA	NA	NA	53657-R	AU	13	0.75	0.00137973	intergenic	NA	NA
55755	NA	NA	NA	NA	NA	55755-R	AG	7	1	0.002331	intergenic	NA	NA
58089	58089-F	AG	9	1	0.000206	NA	NA	NA	NA	NA	intergenic	NA	NA
61818	NA	NA	NA	NA	NA	61818-R	CU	27	0.26	0.02517804	intergenic	NA	NA
62257	62257-F	CU	18	0.56	0.001364	NA	NA	NA	NA	NA	intergenic	NA	NA
62414	NA	NA	NA	NA	NA	62414-R	UA	16	0.56	0.00296142	intergenic	NA	NA
66362	NA	NA	NA	NA	NA	66362-R	GA	31	0.23	0.02644275	intergenic	NA	NA
66384	NA	NA	NA	NA	NA	66384-R	CU	28	0.25	0.02552719	intergenic	NA	NA
66586	NA	NA	NA	NA	NA	66586-R	CU	41	0.18	0.02802697	intergenic	NA	NA
66588	NA	NA	NA	NA	NA	66588-R	CU	39	0.21	0.01431934	intergenic	NA	NA
66589	NA	NA	NA	NA	NA	66589-R	CU	37	0.32	0.00068896	intergenic	NA	NA
67815	67815-F	CU	12	0.58	0.013595	NA	NA	NA	NA	NA	intron	nad2	forward

67941	67941-F	GU	35	0.43	4.75E-05	NA	NA	NA	NA	NA	intron	nad2	forward
67942	67942-F	CU	37	0.41	5.34E-05	NA	NA	NA	NA	NA	intron	nad2	forward
68129	NA	NA	NA	NA	NA	68129-R	AU	42	0.36	6.75E-05	intron	nad2	forward
68943	68943-F	GU	30	0.27	0.012845	NA	NA	NA	NA	NA	intergenic	NA	NA
69127	NA	NA	NA	NA	NA	69127-R	AU	16	0.5	0.00774935	intergenic	NA	NA
69434	NA	NA	NA	NA	NA	69434-R	UC	7	1	0.002331	intergenic	NA	NA
69435	NA	NA	NA	NA	NA	69435-R	UA	5	1	0.02380952	intergenic	NA	NA
70439	nad2-967	CU	10	0.7	0.009883	NA	NA	NA	NA	NA	exon	nad2	forward
70467	NA	NA	NA	NA	NA	nad2-995-as	GA	7	0.86	0.01456876	exon	nad2	forward
70475	NA	NA	NA	NA	NA	nad2-1003-as	GA	12	0.92	5.36E-05	exon	nad2	forward
70476	NA	NA	NA	NA	NA	nad2-1004-as	GA	12	1	4.81E-06	exon	nad2	forward
70483	NA	NA	NA	NA	NA	nad2-1011-as	GA	12	0.92	5.36E-05	exon	nad2	forward
70524	NA	NA	NA	NA	NA	nad2-1052-as	GA	10	1	5.95E-05	exon	nad2	forward
71481	71481-F	UC	20	0.6	0.000216	NA	NA	NA	NA	NA	intergenic	NA	NA
73738	73738-F	AG	8	1	0.000699	NA	NA	NA	NA	NA	intergenic	NA	NA
73739	73739-F	CU	8	1	0.000699	NA	NA	NA	NA	NA	intergenic	NA	NA
76065	76065-F	AU	175	0.3	3.35E-16	NA	NA	NA	NA	NA	rRNA	rrn26	forward
76285	NA	NA	NA	NA	NA	76285-R	GA	4062	0.11	2.36E-126	rRNA	rrn26	forward
76391	NA	NA	NA	NA	NA	76391-R	AC	21	0.55	0.00062382	rRNA	rrn26	forward
76696	NA	NA	NA	NA	NA	76696-R	CU	74	0.18	0.00054893	rRNA	rrn26	forward
76708	76708-F	AU	305	0.11	1.76E-09	NA	NA	NA	NA	NA	rRNA	rrn26	forward
77033	NA	NA	NA	NA	NA	77033-R	CU	99	0.1	0.00481911	rRNA	rrn26	forward
77035	NA	NA	NA	NA	NA	77035-R	UC	93	0.11	0.00475514	rRNA	rrn26	forward
77501	NA	NA	NA	NA	NA	77501-R	CU	37	0.24	0.006892	rRNA	rrn26	forward
77505	NA	NA	NA	NA	NA	77505-R	AG	29	0.31	0.00594995	rRNA	rrn26	forward
77901	NA	NA	NA	NA	NA	77901-R	UC	22	0.32	0.02297398	rRNA	rrn26	forward

78216	78216-F	CU	225	0.11	4.27E-07	NA	NA	NA	NA	NA	rRNA	rrn26	forward
78250	NA	NA	NA	NA	NA	78250-R	CU	143	0.14	5.49E-06	rRNA	rrn26	forward
78465	NA	NA	NA	NA	NA	78465-R	CU	2027	0.26	1.41E-165	rRNA	rrn26	forward
78467	NA	NA	NA	NA	NA	78467-R	UA	969	0.19	3.31E-52	rRNA	rrn26	forward
78713	NA	NA	NA	NA	NA	78713-R	AU	83	0.16	0.00058759	rRNA	rrn26	forward
78714	NA	NA	NA	NA	NA	78714-R	AU	68	0.15	0.00437552	rRNA	rrn26	forward
78735	NA	NA	NA	NA	NA	78735-R	AG	37	0.22	0.01405033	rRNA	rrn26	forward
78777	78777-F	CU	47	0.17	0.015176	NA	NA	NA	NA	NA	rRNA	rrn26	forward
79127	NA	NA	NA	NA	NA	79127-R	AU	41	0.39	2.70E-05	rRNA	rrn26	forward
79236	NA	NA	NA	NA	NA	79236-R	UC	65	0.14	0.00847163	rRNA	rrn26	forward
79417	NA	NA	NA	NA	NA	79417-R	CU	152	0.19	7.25E-09	rRNA	rrn26	forward
81071	NA	NA	NA	NA	NA	81071-R	CU	6	1	0.00757576	intergenic	NA	NA
82369	82369-F	UC	9	0.89	0.001687	NA	NA	NA	NA	NA	intergenic	NA	NA
85560	85560-F	UG	18	0.39	0.020376	NA	NA	NA	NA	NA	intergenic	NA	NA
85850	NA	NA	NA	NA	NA	85850-R	GU	9	1	0.00020568	intergenic	NA	NA
86071	86071-F	UC	15	0.67	0.00085	NA	NA	NA	NA	NA	intergenic	NA	NA
87306	87306-F	UA	5	1	0.02381	NA	NA	NA	NA	NA	intergenic	NA	NA
87308	87308-F	AU	5	1	0.02381	NA	NA	NA	NA	NA	intergenic	NA	NA
88506	88506-F	AG	8	1	0.000699	NA	NA	NA	NA	NA	intergenic	NA	NA
97930	97930-F	CU	56	0.98	8.03E-30	NA	NA	NA	NA	NA	intergenic	NA	NA
97931	97931-F	GU	77	0.23	1.39E-05	NA	NA	NA	NA	NA	intergenic	NA	NA
97932	97932-F	GU	100	0.18	1.79E-05	NA	NA	NA	NA	NA	intergenic	NA	NA
97933	97933-F	AG	60	1	2.46E-33	NA	NA	NA	NA	NA	intergenic	NA	NA
97934	97934-F	GU	102	0.87	2.72E-42	NA	NA	NA	NA	NA	intergenic	NA	NA
97938	97938-F	GA	130	0.12	8.89E-05	NA	NA	NA	NA	NA	intergenic	NA	NA
98128	NA	NA	NA	NA	NA	98128-R	AG	346	0.1	9.10E-10	intergenic	NA	NA

98228	NA	NA	NA	NA	NA	98228-R	GA	6	1	0.00757576	intergenic	NA	NA
98229	NA	NA	NA	NA	NA	98229-R	AU	6	1	0.00757576	intergenic	NA	NA
98231	NA	NA	NA	NA	NA	98231-R	GC	6	1	0.00757576	intergenic	NA	NA
102579	atp1-460-as	CU	16	0.44	0.018626	NA	NA	NA	NA	NA	exon	atp1	reverse
102914	atp1-125-as	CU	88	0.19	3.38E-05	NA	NA	NA	NA	NA	exon	atp1	reverse
107063	107063-F	AU	46	0.2	0.007594	NA	NA	NA	NA	NA	rRNA	rrn18	reverse
107334	NA	NA	NA	NA	NA	107334-R	CU	109	0.17	1.91E-05	rRNA	rrn18	reverse
107346	NA	NA	NA	NA	NA	107346-R	AU	53	0.38	1.39E-06	rRNA	rrn18	reverse
107882	107882-F	CU	2494	0.15	3.9E-113	NA	NA	NA	NA	NA	rRNA	rrn18	reverse
107883	107883-F	AU	2689	0.1	4.32E-74	NA	NA	NA	NA	NA	rRNA	rrn18	reverse
107886	107886-F	CU	2833	0.12	1.44E-98	NA	NA	NA	NA	NA	rRNA	rrn18	reverse
108166	NA	NA	NA	NA	NA	108166-R	GU	459	0.17	1.90E-23	rRNA	rrn18	reverse
108306	NA	NA	NA	NA	NA	108306-R	AU	199	0.27	1.26E-16	rRNA	rrn18	reverse
119262	orf106b-1	AU	6	1	0.007576	NA	NA	NA	NA	NA	exon	orf106b	forward
122186	122186-F	CU	9	0.89	0.001687	NA	NA	NA	NA	NA	intergenic	NA	NA
125449	125449-F	GU	11	1	1.7E-05	NA	NA	NA	NA	NA	intergenic	NA	NA
125618	NA	NA	NA	NA	NA	125618-R	AU	11	1	1.701e-005	intergenic	NA	NA
127548	NA	NA	NA	NA	NA	127548-R	CU	35	0.23	0.01375231	intron	rps10	reverse
127580	127580-F	CA	51	0.16	0.015507	NA	NA	NA	NA	NA	intron	rps10	reverse
127728	NA	NA	NA	NA	NA	127728-R	CG	38	0.26	0.00334672	intron	rps10	reverse
127907	127907-F	AG	12	0.64	0.011868	NA	NA	NA	NA	NA	intron	rps10	reverse
127987	127987-F	AU	17	0.41	0.019548	NA	NA	NA	NA	NA	intron	rps10	reverse
128494	rps10-99-as	CU	7	1	0.002331	NA	NA	NA	NA	NA	exon	rps10	reverse
128591	NA	NA	NA	NA	NA	rps10-2	CU	7	1	0.002331	exon	rps10	reverse
128646	128646-F	GA	5	1	0.02381	128646-R	CU	7	1	0.002331	intergenic	NA	NA
129236	NA	NA	NA	NA	NA	129236-R	CU	8	0.75	0.02027972	intergenic	NA	NA

129267	129267-F	AG	13	0.69	0.001804	NA	NA	NA	NA	NA	intergenic	NA	NA	
129395	NA	NA	NA	NA	NA	129395-R	AU	9	0.67	0.02488688	intergenic	NA	NA	
129938	129938-F	CU	16	0.62	0.001027	NA	NA	NA	NA	NA	intergenic	NA	NA	
129941	129941-F	CU	15	0.67	0.00085	NA	NA	NA	NA	NA	intergenic	NA	NA	
129942	129942-F	UC	16	0.62	0.001027	NA	NA	NA	NA	NA	intergenic	NA	NA	
138153	138153-F	GA	43	0.31	0.000354	NA	NA	NA	NA	NA	intergenic	NA	NA	
138358	NA	NA	NA	NA	NA	138358-R	CU	34	0.65	2.36E-08	intergenic	NA	NA	
138815	NA	NA	NA	NA	NA	ccmC-400	CU	10	0.9	0.00054667	exon	ccmC	reverse	
138816	NA	NA	NA	NA	NA	ccmC-399	CU	11	0.91	0.00017294	exon	ccmC	reverse	
138884	NA	NA	NA	NA	NA	ccmC-331	CU	7	0.86	0.01456876	exon	ccmC	reverse	
139031	NA	NA	NA	NA	NA	ccmC-184	CU	5	1	0.02380952	exon	ccmC	reverse	
139036	NA	NA	NA	NA	NA	ccmC-179	CU	5	1	0.02380952	exon	ccmC	reverse	
139054	NA	NA	NA	NA	NA	ccmC-161	CU	6	1	0.00757576	exon	ccmC	reverse	
139112	ccmC-103-as	GA	5	1	0.02381	NA	NA	NA	NA	NA	exon	ccmC	reverse	
139177	ccmC-38-as	GA	15	0.73	0.000242	NA	NA	NA	NA	NA	exon	ccmC	reverse	
140026	140026-F	GU	62	1	4.14E-35	NA	NA	NA	NA	NA	intergenic	NA	NA	
140028	140028-F	GU	120	0.84	1.15E-46	NA	NA	NA	NA	NA	intergenic	NA	NA	
140029	140029-F	CU	162	0.88	7.34E-69	NA	NA	NA	NA	NA	intergenic	NA	NA	
140031	NA	NA	NA	NA	NA	140031-R	CG	9	1	0.00020568	intergenic	NA	NA	
140032	NA	NA	NA	NA	NA	140032-R	UA	11	1	1.701e-005	intergenic	NA	NA	
140168	140168-F	AU	8	1	0.002331	NA	NA	NA	NA	NA	intergenic	NA	NA	
140169	140169-F	AU	8	1	0.000699	NA	NA	NA	NA	NA	intergenic	NA	NA	
140170	140170-F	AG	7	1	0.007576	NA	NA	NA	NA	NA	intergenic	NA	NA	
140256	140256-F	CU	19	1	5.66E-10	NA	NA	NA	NA	NA	intergenic	NA	NA	Yes
140273	140273-F	AG	19	1	5.66E-10	NA	NA	NA	NA	NA	intergenic	NA	NA	
140276	140276-F	UC	19	1	5.66E-10	NA	NA	NA	NA	NA	intergenic	NA	NA	

140301	140301-F	CU	16	1	2.83E-08	NA	NA	NA	NA	NA	intergenic	NA	NA	
140302	140302-F	UC	18	1	2.09E-09	NA	NA	NA	NA	NA	intergenic	NA	NA	
140320	140320-F	AG	18	1	2.09E-09	NA	NA	NA	NA	NA	intergenic	NA	NA	
144302	rpl2-283-as	CU	31	0.23	0.026443	NA	NA	NA	NA	NA	exon	rpl2	reverse	
144424	rpl2-161-as	CU	262	0.35	1.33E-30	NA	NA	NA	NA	NA	exon	rpl2	reverse	
144427	rpl2-158-as	CU	265	0.15	8.76E-12	NA	NA	NA	NA	NA	exon	rpl2	reverse	
144428	rpl2-157-as	CU	263	0.17	1.95E-13	NA	NA	NA	NA	NA	exon	rpl2	reverse	
144579	rpl2-6-as	CU	26	0.31	0.01189	NA	NA	NA	NA	NA	exon	rpl2	reverse	
144796	rpl23-89-as	GA	44	0.84	4.81E-16	rpl23-89	CU	43	0.86	7.05E-17	exon	rpl23	reverse	
144814	rpl23-71-as	GA	15	1	1.03E-07	rpl23-71	CU	46	0.76	1.31E-14	exon	rpl23	reverse	
145792	NA	NA	NA	NA	NA	145792-R	GU	5	1	0.02380952	intergenic	NA	NA	
145812	NA	NA	NA	NA	NA	145812-R	CU	8	0.75	0.02027972	intergenic	NA	NA	
145845	NA	NA	NA	NA	NA	145845-R	UG	8	0.75	0.02027972	intergenic	NA	NA	
145854	NA	NA	NA	NA	NA	145854-R	AG	8	0.75	0.02027972	intergenic	NA	NA	
145903	NA	NA	NA	NA	NA	145903-R	CU	5	1	0.02380952	intergenic	NA	NA	
150198	NA	NA	NA	NA	NA	150198-R	AU	5	1	0.02380952	intergenic	NA	NA	Yes
150204	NA	NA	NA	NA	NA	150204-R	UC	6	1	0.00757576	intergenic	NA	NA	Yes
150815	150815-F	GA	7	0.86	0.014569	NA	NA	NA	NA	NA	intergenic	NA	NA	
150834	150834-F	AC	7	0.86	0.014569	NA	NA	NA	NA	NA	intergenic	NA	NA	
151637	NA	NA	NA	NA	NA	151637-R	UC	10	0.9	0.00054667	intergenic	NA	NA	
153301	153301-F	GA	20	0.5	0.001671	153301-R	CU	32	0.91	9.83E-14	intergenic	NA	NA	
153334	NA	NA	NA	NA	NA	153334-R	GU	27	0.26	0.02517804	intergenic	NA	NA	
153800	153800-F	AG	45	0.18	0.01489	NA	NA	NA	NA	NA	intergenic	NA	NA	
153946	153946-F	GA	16	0.88	3.42E-06	153946-R	CU	67	0.7	5.90E-19	intergenic	NA	NA	
153952	153952-F	GA	16	0.94	4.28E-07	153952-R	CU	62	0.85	2.32E-24	intergenic	NA	NA	
154711	154711-F	GA	125	0.97	1.43E-65	154711-R	CU	104	0.82	3.11E-38	intergenic	NA	NA	

154720	154720-F	GA	152	0.97	2.31E-81	154720-R	CU	97	0.89	1.88E-41	intergenic	NA	NA
154837	154837-F	CU	482	0.13	8.11E-18	NA	NA	NA	NA	NA	intergenic	NA	NA
154871	154871-F	GA	515	0.95	2.5E-262	154871-R	CU	106	0.95	3.47E-53	intergenic	NA	NA
154990	154990-F	GA	90	0.99	8.9E-50	154990-R	CU	498	0.95	3.42E-251	intergenic	NA	NA
155112	NA	NA	NA	NA	NA	155112-R	CU	78	0.14	0.00231364	intergenic	NA	NA
155308	155308-F	GA	408	0.96	4.6E-215	155308-R	CU	312	0.95	3.69E-159	intergenic	NA	NA
155709	155709-F	AG	674	0.1	6.99E-19	NA	NA	NA	NA	NA	intergenic	NA	NA
155792	rps7-407-as	CU	2075	0.15	1.64E-89	NA	NA	NA	NA	NA	exon	rps7	reverse
155795	rps7-404-as	AG	2131	0.12	7.09E-75	NA	NA	NA	NA	NA	exon	rps7	reverse
155910	rps7-289-as	UG	91	0.11	0.004732	NA	NA	NA	NA	NA	exon	rps7	reverse
155911	rps7-288-as	UC	91	0.11	0.004732	NA	NA	NA	NA	NA	exon	rps7	reverse
156024	NA	NA	NA	NA	NA	rps7-175	CU	212	0.14	1.10E-08	exon	rps7	reverse
156161	rps7-38-as	UG	45	0.24	0.001773	NA	NA	NA	NA	NA	exon	rps7	reverse
156198	NA	NA	NA	NA	NA	rps7-1	AU	135	0.11	0.00035702	exon	rps7	reverse
156635	NA	NA	NA	NA	NA	156635-R	UA	91	0.11	0.00473204	intergenic	NA	NA
156803	NA	NA	NA	NA	NA	156803-R	GA	41	0.2	0.01456336	intergenic	NA	NA
156929	156929-F	GA	166	0.94	2.34E-81	156929-R	CU	34	0.91	8.05E-15	intergenic	NA	NA
158666	158666-F	AG	13	1	1.35E-06	NA	NA	NA	NA	NA	tRNA	trnV-GAC	forward
158746	158746-F	UC	9	1	0.000206	NA	NA	NA	NA	NA	intergenic	NA	NA
158821	158821-F	GU	11	0.64	0.011868	NA	NA	NA	NA	NA	intergenic	NA	NA
158831	158831-F	CU	85	0.24	6.58E-06	NA	NA	NA	NA	NA	intergenic	NA	NA
158832	158832-F	GU	127	0.14	2.12E-05	NA	NA	NA	NA	NA	intergenic	NA	NA
158935	158935-F	AU	143	0.24	5.54E-10	NA	NA	NA	NA	NA	intergenic	NA	NA
159098	159098-F	CU	2727	0.13	3E-107	NA	NA	NA	NA	NA	intergenic	NA	NA
159343	159343-F	CU	4771	0.12	4.5E-169	NA	NA	NA	NA	NA	intergenic	NA	NA
159363	159363-F	AU	7655	0.15	0	NA	NA	NA	NA	NA	intergenic	NA	NA

159818	NA	NA	NA	NA	NA	159818-R	CU	3702	0.17	1.32E-178	intergenic	NA	NA
159851	NA	NA	NA	NA	NA	159851-R	CU	154	0.16	6.20E-07	intergenic	NA	NA
159924	NA	NA	NA	NA	NA	159924-R	GC	29	0.76	5.20E-09	intergenic	NA	NA
159925	159925-F	AG	8	1	0.000699	NA	NA	NA	NA	NA	intergenic	NA	NA
159929	159929-F	UC	6	1	0.007576	159929-R	AG	16	0.94	4.28E-07	intergenic	NA	NA
162216	NA	NA	NA	NA	NA	162216-R	AG	9	1	0.00020568	intergenic	NA	NA
162220	NA	NA	NA	NA	NA	162220-R	CU	10	0.8	0.00273875	intergenic	NA	NA
168359	rpl16-449-as	GA	59	0.98	1.43E-31	rpl16-449	CU	9	1	0.00020568	exon	rpl16	reverse
168662	rpl16-146-as	GA	170	0.98	5.63E-93	rpl16-146	CU	379	0.99	6.20E-220	exon	rpl16	reverse
168841	rps3-1558-as	GA	20	0.85	2.03E-07	rps3-1558	CU	80	0.99	6.95E-44	exon	rps3	reverse
168874	rps3-1525-as	GA	12	0.67	0.004711	rps3-1525	CU	36	0.81	1.84E-12	exon	rps3	reverse
168927	NA	NA	NA	NA	NA	rps3-1472	CU	31	0.81	1.10E-10	exon	rps3	reverse
169698	NA	NA	NA	NA	NA	rps3-701	CU	13	0.92	1.64E-05	exon	rps3	reverse
172201	NA	NA	NA	NA	NA	172201-R	CU	42	0.31	0.00035436	intergenic	NA	NA
173306	rps4-193-as	GA	10	1	5.95E-05	NA	NA	NA	NA	NA	exon	rps4	reverse
173335	rps4-164-as	GA	12	1	4.81E-06	NA	NA	NA	NA	NA	exon	rps4	reverse
175663	NA	NA	NA	NA	NA	175663-R	CA	7	0.86	0.01456876	intergenic	NA	NA
179573	179573-F	CU	14	0.5	0.016425	NA	NA	NA	NA	NA	intergenic	NA	NA
179757	nad1-376-as	GA	7	1	0.002331	nad1-376	CU	6	1	0.00757576	exon	nad1	reverse
179825	nad1-308-as	GA	5	1	0.02381	nad1-308	CU	11	0.82	0.00095261	exon	nad1	reverse
179826	nad1-307-as	GA	5	1	0.02381	nad1-307	CU	11	0.73	0.0037594	exon	nad1	reverse
179868	nad1-265-as	GA	13	1	1.35E-06	nad1-265	CU	11	1	1.701e-005	exon	nad1	reverse
179918	nad1-215-as	GA	11	1	1.7E-05	nad1-215	CU	14	1	3.74E-07	exon	nad1	reverse
180011	NA	NA	NA	NA	NA	nad1-122	AU	13	0.77	0.00049132	exon	nad1	reverse
180818	180818-F	GA	26	0.31	0.01189	NA	NA	NA	NA	NA	intergenic	NA	NA
180828	180828-F	GA	27	1	1.44E-14	NA	NA	NA	NA	NA	intergenic	NA	NA

183582	183582-F	CU	5	1	0.02381	NA	NA	NA	NA	NA	intergenic	NA	NA
183614	183614-F	GA	5	1	0.02381	NA	NA	NA	NA	NA	intergenic	NA	NA
183618	183618-F	CU	5	1	0.02381	NA	NA	NA	NA	NA	intergenic	NA	NA
183622	183622-F	CU	5	1	0.02381	NA	NA	NA	NA	NA	intergenic	NA	NA
183648	183648-F	GA	5	1	0.02381	NA	NA	NA	NA	NA	intergenic	NA	NA
183653	183653-F	AC	5	1	0.02381	NA	NA	NA	NA	NA	intergenic	NA	NA
183686	183686-F	AG	7	1	0.002331	183686-R	UC	6	1	0.00757576	intergenic	NA	NA
183714	183714-F	AG	7	1	0.002331	183714-R	UC	6	1	0.00757576	intergenic	NA	NA
183715	183715-F	CU	7	1	0.002331	183715-R	GA	6	1	0.00757576	intergenic	NA	NA
183716	183716-F	UC	7	1	0.002331	183716-R	AG	6	1	0.00757576	intergenic	NA	NA
183733	183733-F	GA	7	1	0.002331	183733-R	CU	6	1	0.00757576	intergenic	NA	NA
183763	183763-F	CU	6	1	0.007576	NA	NA	NA	NA	NA	intergenic	NA	NA
185956	185956-F	CU	12	0.75	0.00138	NA	NA	NA	NA	NA	intergenic	NA	NA
186404	NA	NA	NA	NA	NA	186404-R	AU	8	1	0.0006993	intergenic	NA	NA
188596	188596-F	AU	11	0.64	0.011868	NA	NA	NA	NA	NA	intergenic	NA	NA
193812	NA	NA	NA	NA	NA	193812-R	AU	12	0.92	5.36E-05	intergenic	NA	NA
194566	194566-F	CU	28	0.29	0.012399	NA	NA	NA	NA	NA	intergenic	NA	NA
204361	204361-F	CU	55	0.35	3.49E-06	NA	NA	NA	NA	NA	intergenic	NA	NA
204858	nad5-1466	CU	10	1	5.95E-05	NA	NA	NA	NA	NA	exon	nad5	forward
204918	nad5-1526	CU	10	1	5.95E-05	nad5-1526-as	GA	9	1	0.00020568	exon	nad5	forward
204936	nad5-1544	CU	10	1	5.95E-05	nad5-1544-as	GA	9	1	0.00020568	exon	nad5	forward
204948	NA	NA	NA	NA	NA	nad5-1556-as	GA	9	1	0.00020568	exon	nad5	forward
204978	nad5-1586	CU	6	1	0.007576	nad5-1586-as	GA	8	1	0.0006993	exon	nad5	forward
205925	NA	NA	NA	NA	NA	205925-R	GA	29	0.24	0.0258529	intergenic	NA	NA
206270	206270-F	AU	10	0.9	0.000547	NA	NA	NA	NA	NA	intergenic	NA	NA
206271	206271-F	CU	10	0.9	0.000547	NA	NA	NA	NA	NA	intergenic	NA	NA

206303	nad5-1871	CU	13	1	1.35E-06	NA	NA	NA	NA	NA	exon	nad5	forward	
206324	nad5-1892	CU	11	1	1.7E-05	nad5-1892-as	GA	8	1	0.0006993	exon	nad5	forward	
206326	nad5-1894	CU	13	1	1.35E-06	nad5-1894-as	GA	8	1	0.0006993	exon	nad5	forward	
206366	nad5-1934	CU	10	1	5.95E-05	nad5-1934-as	GA	36	1	8.36E-20	exon	nad5	forward	
206389	NA	NA	NA	NA	NA	nad5-1957-as	GA	37	1	2.18E-20	exon	nad5	forward	
206408	NA	NA	NA	NA	NA	nad5-1976-as	GA	37	0.43	2.15E-05	exon	nad5	forward	
206412	NA	NA	NA	NA	NA	nad5-1980-as	AG	37	0.19	0.02783672	exon	nad5	forward	
208918	208918-F	AU	23	0.87	5.9E-09	NA	NA	NA	NA	NA	intergenic	NA	NA	
208919	208919-F	GU	35	0.34	0.000646	NA	NA	NA	NA	NA	intergenic	NA	NA	
209070	NA	NA	NA	NA	NA	209070-R	UA	31	0.58	1.53E-06	intergenic	NA	NA	
209071	NA	NA	NA	NA	NA	209071-R	UC	32	0.28	0.00635145	intergenic	NA	NA	
211446	211446-F	CG	17	0.53	0.0033	NA	NA	NA	NA	NA	intergenic	NA	NA	
211449	211449-F	UC	17	0.94	1.24E-07	NA	NA	NA	NA	NA	intergenic	NA	NA	
211451	211451-F	GA	17	1	2.83E-08	NA	NA	NA	NA	NA	intergenic	NA	NA	
211457	211457-F	AC	28	1	1.44E-14	211457-R	UG	5	1	0.02380952	intergenic	NA	NA	Yes
215230	215230-F	AU	9	1	0.000206	NA	NA	NA	NA	NA	intergenic	NA	NA	
215231	215231-F	AG	9	1	0.000206	NA	NA	NA	NA	NA	intergenic	NA	NA	
217868	NA	NA	NA	NA	NA	217868-R	CU	5	1	0.02380952	intergenic	NA	NA	
224344	NA	NA	NA	NA	NA	224344-R	UG	9	0.78	0.00761004	intergenic	NA	NA	
224346	NA	NA	NA	NA	NA	224346-R	CU	9	0.78	0.00761004	intergenic	NA	NA	
224349	NA	NA	NA	NA	NA	224349-R	GU	9	0.78	0.00761004	intergenic	NA	NA	
224350	NA	NA	NA	NA	NA	224350-R	AG	14	0.86	3.44e-005	intergenic	NA	NA	
224352	NA	NA	NA	NA	NA	224352-R	UG	16	0.88	3.42E-06	intergenic	NA	NA	
224443	NA	NA	NA	NA	NA	224443-R	CU	17	0.41	0.01954847	intergenic	NA	NA	
225265	orf151-165-as	CU	8	1	0.000699	NA	NA	NA	NA	NA	exon	orf151	reverse	
225483	NA	NA	NA	NA	NA	225483-R	GU	7	1	0.002331	intergenic	NA	NA	

235588	NA	NA	NA	NA	NA	235588-R	UC	16	0.88	3.42E-06	intergenic	NA	NA	
235589	NA	NA	NA	NA	NA	235589-R	UA	16	0.88	3.42E-06	intergenic	NA	NA	
237150	NA	NA	NA	NA	NA	237150-R	AC	8	1	0.0006993	intergenic	NA	NA	
243473	243473-F	GU	7	1	0.002331	NA	NA	NA	NA	NA	intergenic	NA	NA	
243474	243474-F	UA	7	1	0.002331	NA	NA	NA	NA	NA	intergenic	NA	NA	
243475	243475-F	UA	7	1	0.002331	NA	NA	NA	NA	NA	intergenic	NA	NA	
245623	245623-F	CU	14	1	1.35E-06	NA	NA	NA	NA	NA	intergenic	NA	NA	
245644	245644-F	CA	96	1	2.69E-55	245644-R	GU	38	1	5.66E-21	intergenic	NA	NA	Yes
245649	245649-F	GA	134	0.99	3.05E-75	245649-R	CU	41	1	9.89E-23	intergenic	NA	NA	Yes
245676	245676-F	CU	201	1	4.9E-118	245676-R	GA	50	1	5.06E-28	intergenic	NA	NA	Yes
245700	245700-F	CU	343	1	3.5E-203	245700-R	GA	117	1	8.20E-68	intergenic	NA	NA	
246132	246132-F	UG	34	0.24	0.013591	246132-R	AG	582	0.14	1.79E-24	intergenic	NA	NA	
266013	nad1-919-as	GA	19	0.84	6.46E-07	nad1-919	CU	9	1	0.00020568	exon	nad1	reverse	
266043	nad1-889-as	GA	17	0.82	6.3E-06	nad1-889	CU	14	1	3.74E-07	exon	nad1	reverse	
266118	nad1-814-as	GA	11	1	1.7E-05	nad1-814	CU	11	0.91	0.00017294	exon	nad1	reverse	
266201	NA	NA	NA	NA	NA	nad1-731	CU	5	1	0.02380952	exon	nad1	reverse	
267190	rps13-218-as	CU	10	0.7	0.009883	NA	NA	NA	NA	NA	exon	rps13	reverse	
267308	NA	NA	NA	NA	NA	rps13-100	CU	18	1	2.09E-09	exon	rps13	reverse	
267352	NA	NA	NA	NA	NA	rps13-56	CU	21	1	4.09E-11	exon	rps13	reverse	
267468	267468-F	CU	10	0.9	0.000547	NA	NA	NA	NA	NA	intergenic	NA	NA	
267520	267520-F	GA	16	0.69	0.000318	NA	NA	NA	NA	NA	intergenic	NA	NA	
273421	NA	NA	NA	NA	NA	nad2-455	CU	5	1	0.02380952	exon	nad2	reverse	
273490	nad2-386-as	GA	5	1	0.02381	NA	NA	NA	NA	NA	exon	nad2	reverse	
273517	nad2-359-as	GA	5	1	0.02381	nad2-359	CU	6	1	0.00757576	exon	nad2	reverse	
273551	NA	NA	NA	NA	NA	nad2-325	CU	6	1	0.00757576	exon	nad2	reverse	
273557	NA	NA	NA	NA	NA	nad2-319	CU	6	1	0.00757576	exon	nad2	reverse	

273562	NA	NA	NA	NA	NA	nad2-314	CU	6	1	0.00757576	exon	nad2	reverse	
287942	ccmFc-397-as	GA	8	0.88	0.005051	NA	NA	NA	NA	NA	exon	ccmFc	reverse	
288029	NA	NA	NA	NA	NA	ccmFc-310	CU	7	1	0.002331	exon	ccmFc	reverse	
291613	NA	NA	NA	NA	NA	291613-R	AG	40	0.4	2.57E-05	intergenic	NA	NA	
291966	nad4L-158	CU	10	1	5.95E-05	NA	NA	NA	NA	NA	exon	nad4L	forward	
291987	nad4L-179	CU	14	1	3.74E-07	NA	NA	NA	NA	NA	exon	nad4L	forward	
291996	nad4L-188	CU	18	1	2.09E-09	NA	NA	NA	NA	NA	exon	nad4L	forward	
292005	nad4L-197	CU	19	1	5.66E-10	NA	NA	NA	NA	NA	exon	nad4L	forward	
292089	nad4L-281	CU	17	0.94	1.24E-07	nad4L-281-as	GA	5	1	0.02380952	exon	nad4L	forward	
292338	atp4-59	CU	12	1	4.81E-06	atp4-59-as	GA	16	1	2.83E-08	exon	atp4	forward	
292350	atp4-71	CU	13	1	1.35E-06	atp4-71-as	GA	20	1	1.52E-10	exon	atp4	forward	
292368	atp4-89	CU	13	1	1.35E-06	atp4-89-as	GA	11	1	1.701e-005	exon	atp4	forward	
292397	NA	NA	NA	NA	NA	atp4-118-as	GA	15	1	1.03E-07	exon	atp4	forward	
292674	atp4-395	CU	28	1	3.79E-15	NA	NA	NA	NA	NA	exon	atp4	forward	
292686	atp4-407	CU	30	1	2.62E-16	NA	NA	NA	NA	NA	exon	atp4	forward	
292695	atp4-416	CU	33	0.97	1.51E-16	NA	NA	NA	NA	NA	exon	atp4	forward	
293748	293748-F	AU	7	1	0.002331	NA	NA	NA	NA	NA	intergenic	NA	NA	Yes
293790	293790-F	CU	16	1	2.83E-08	NA	NA	NA	NA	NA	intergenic	NA	NA	
301471	NA	NA	NA	NA	NA	orf104c-173	CG	5	1	0.02380952	exon	orf104c	reverse	
304442	NA	NA	NA	NA	NA	orf100b-276-as	AU	7	1	0.002331	exon	orf100b	forward	
308383	NA	NA	NA	NA	NA	308383-R	CA	49	0.16	0.01534786	intergenic	NA	NA	
308419	NA	NA	NA	NA	NA	308419-R	CU	80	0.11	0.00888218	intergenic	NA	NA	
308737	308737-F	CU	2437	0.17	1.8E-126	NA	NA	NA	NA	NA	intergenic	NA	NA	
308738	308738-F	AU	2650	0.12	1.87E-86	NA	NA	NA	NA	NA	intergenic	NA	NA	
308741	308741-F	CU	2782	0.11	1.11E-88	NA	NA	NA	NA	NA	intergenic	NA	NA	
309021	NA	NA	NA	NA	NA	309021-R	GU	450	0.18	7.39E-26	intergenic	NA	NA	

309161	NA	NA	NA	NA	NA	309161-R	AU	227	0.24	2.17E-16	intergenic	NA	NA
318159	318159-F	UC	77	0.16	0.001146	NA	NA	NA	NA	NA	intergenic	NA	NA
318160	318160-F	GA	76	0.14	0.002293	NA	NA	NA	NA	NA	intergenic	NA	NA
318223	nad4-1424-as	GA	100	0.66	4.01E-26	nad4-1424	CU	27	0.48	0.00017306	exon	nad4	reverse
318239	nad4-1408-as	GA	86	0.79	1.04E-29	nad4-1408	CU	47	0.66	5.44E-12	exon	nad4	reverse
318261	318261-F	GA	24	0.92	2.15E-10	318261-R	CU	58	0.91	5.70E-26	intron	nad4	reverse
318375	NA	NA	NA	NA	NA	orf111c-324	GU	13	0.77	0.00049132	exon	orf111c	reverse
318445	orf111c-254-as	CU	16	0.44	0.018626	NA	NA	NA	NA	NA	exon	orf111c	reverse
318471	orf111c-228-as	GU	31	0.48	3.52E-05	NA	NA	NA	NA	NA	exon	orf111c	reverse
318613	orf111c-86-as	CU	16	0.75	8.54E-05	NA	NA	NA	NA	NA	exon	orf111c	reverse
318644	NA	NA	NA	NA	NA	orf111c-55	CU	15	1	1.03E-07	exon	orf111c	reverse
320409	320409-F	CG	14	0.64	0.002212	NA	NA	NA	NA	NA	intron	nad4	reverse
320410	320410-F	CG	15	0.87	1.09E-05	NA	NA	NA	NA	NA	intron	nad4	reverse
320423	320423-F	UG	17	0.76	2.96E-05	NA	NA	NA	NA	NA	intron	nad4	reverse
320445	320445-F	CU	21	0.57	0.00025	NA	NA	NA	NA	NA	intron	nad4	reverse
320469	320469-F	CU	22	0.73	2.43E-06	NA	NA	NA	NA	NA	intron	nad4	reverse
320470	320470-F	CG	22	0.73	2.43E-06	NA	NA	NA	NA	NA	intron	nad4	reverse
321120	NA	NA	NA	NA	NA	nad4-1364	CU	11	1	1.701e-005	exon	nad4	reverse
321138	nad4-1346-as	GA	29	1	9.98E-16	nad4-1346	CU	14	1	3.74E-07	exon	nad4	reverse
321186	nad4-1298-as	GA	28	1	3.79E-15	nad4-1298	CU	21	1	4.09E-11	exon	nad4	reverse
321321	NA	NA	NA	NA	NA	nad4-1163	CU	6	1	0.00757576	exon	nad4	reverse
321342	nad4-1142-as	GA	10	1	5.95E-05	nad4-1142	CU	5	1	0.02380952	exon	nad4	reverse
321361	nad4-1123-as	GA	10	1	5.95E-05	NA	NA	NA	NA	NA	exon	nad4	reverse
321364	nad4-1120-as	GA	11	1	1.7E-05	NA	NA	NA	NA	NA	exon	nad4	reverse
321384	nad4-1100-as	GA	10	1	5.95E-05	NA	NA	NA	NA	NA	exon	nad4	reverse
321477	nad4-1007-as	GA	9	0.78	0.00761	nad4-1007	CU	6	1	0.00757576	exon	nad4	reverse

321483	nad4-1001-as	GA	9	1	0.000206	nad4-1001	CU	6	1	0.00757576	exon	nad4	reverse
321487	nad4-997-as	GA	9	0.78	0.00761	NA	NA	NA	NA	NA	exon	nad4	reverse
322312	322312-F	AG	15	0.8	5.79E-05	NA	NA	NA	NA	NA	intron	nad4	reverse
322313	322313-F	CU	15	0.73	0.000242	NA	NA	NA	NA	NA	intron	nad4	reverse
324209	nad4-878-as	GA	9	0.67	0.024887	nad4-878	CU	7	0.86	0.01456876	exon	nad4	reverse
324239	nad4-848-as	GA	11	0.73	0.003759	nad4-848	CU	18	0.72	4.143e-005	exon	nad4	reverse
324240	NA	NA	NA	NA	NA	nad4-847	CU	18	0.72	4.143e-005	exon	nad4	reverse
324329	NA	NA	NA	NA	NA	nad4-758	CU	20	0.9	2.91E-08	exon	nad4	reverse
324488	nad4-599-as	GA	14	0.93	4.91E-06	NA	NA	NA	NA	NA	exon	nad4	reverse
324767	324767-F	CG	53	0.27	0.000407	NA	NA	NA	NA	NA	intron	nad4	reverse
324972	NA	NA	NA	NA	NA	324972-R	CU	97	0.12	0.00245727	intron	nad4	reverse
325192	325192-F	UA	44	0.35	7.02E-05	NA	NA	NA	NA	NA	intron	nad4	reverse
325194	325194-F	CU	44	0.18	0.01489	NA	NA	NA	NA	NA	intron	nad4	reverse
325209	325209-F	GA	45	0.24	0.001773	NA	NA	NA	NA	NA	intron	nad4	reverse
325849	325849-F	AU	65	0.17	0.00414	NA	NA	NA	NA	NA	intron	nad4	reverse
325909	NA	NA	NA	NA	NA	325909-R	GU	12	0.58	0.01359537	intron	nad4	reverse
326042	NA	NA	NA	NA	NA	nad4-440	CU	22	0.86	1.94E-08	exon	nad4	reverse
326054	NA	NA	NA	NA	NA	nad4-428	CU	23	0.78	1.88E-07	exon	nad4	reverse
326055	NA	NA	NA	NA	NA	nad4-427	CU	24	0.92	2.15E-10	exon	nad4	reverse
326058	NA	NA	NA	NA	NA	nad4-424	CU	21	0.71	6.97E-06	exon	nad4	reverse
326141	nad4-341-as	UA	21	0.52	0.000696	NA	NA	NA	NA	NA	exon	nad4	reverse
326187	nad4-295-as	GA	35	0.29	0.003162	NA	NA	NA	NA	NA	exon	nad4	reverse
326294	NA	NA	NA	NA	NA	nad4-188	CU	40	0.9	4.26E-17	exon	nad4	reverse
326306	NA	NA	NA	NA	NA	nad4-176	CU	40	0.17	0.02837934	exon	nad4	reverse
326325	NA	NA	NA	NA	NA	nad4-157	CU	34	0.85	5.32E-13	exon	nad4	reverse
326333	NA	NA	NA	NA	NA	nad4-149	CU	33	0.85	1.76E-12	exon	nad4	reverse

326382	NA	NA	NA	NA	NA	nad4-100	AU	13	0.85	0.00010634	exon	nad4	reverse	
327995	327995-F	CA	6	1	0.007576	NA	NA	NA	NA	NA	intergenic	NA	NA	
328094	328094-F	GA	5	1	0.02381	328094-R	CU	6	1	0.00757576	intergenic	NA	NA	
329702	329702-F	UC	8	1	0.000699	NA	NA	NA	NA	NA	intergenic	NA	NA	
329712	329712-F	AG	9	1	0.000206	NA	NA	NA	NA	NA	tRNA	trnS-GGA	forward	Yes
329726	329726-F	UC	9	1	0.000206	NA	NA	NA	NA	NA	tRNA	trnS-GGA	forward	Yes
329744	329744-F	UC	9	1	0.000206	NA	NA	NA	NA	NA	tRNA	trnS-GGA	forward	Yes
329793	329793-F	UC	7	1	0.002331	NA	NA	NA	NA	NA	intergenic	NA	NA	
329795	329795-F	AC	9	1	0.000206	NA	NA	NA	NA	NA	intergenic	NA	NA	
331623	NA	NA	NA	NA	NA	331623-R	CG	15	0.93	1.46E-06	intergenic	NA	NA	
331624	NA	NA	NA	NA	NA	331624-R	UC	14	1	3.74E-07	intergenic	NA	NA	
332277	NA	NA	NA	NA	NA	332277-R	CU	946	0.13	6.35E-36	rRNA	rrn26	reverse	
332283	NA	NA	NA	NA	NA	332283-R	GU	759	0.13	1.20E-29	rRNA	rrn26	reverse	
332576	NA	NA	NA	NA	NA	332576-R	CG	3787	0.11	8.93E-133	rRNA	rrn26	reverse	
332751	NA	NA	NA	NA	NA	332751-R	AU	1721	0.1	7.53E-51	rRNA	rrn26	reverse	
332807	332807-F	AU	282	0.37	2.55E-34	NA	NA	NA	NA	NA	rRNA	rrn26	reverse	
333051	333051-F	CU	1512	0.11	1.43E-44	NA	NA	NA	NA	NA	rRNA	rrn26	reverse	
333877	333877-F	UA	691	0.11	2.9E-23	NA	NA	NA	NA	NA	rRNA	rrn26	reverse	
334003	334003-F	AG	518	0.11	7.4E-16	NA	NA	NA	NA	NA	rRNA	rrn26	reverse	
334355	334355-F	CU	1114	0.11	4.97E-35	NA	NA	NA	NA	NA	rRNA	rrn26	reverse	
334515	NA	NA	NA	NA	NA	334515-R	CU	4061	0.11	5.93E-130	rRNA	rrn26	reverse	
334621	NA	NA	NA	NA	NA	334621-R	AU	6002	0.12	1.91E-205	rRNA	rrn26	reverse	
334631	NA	NA	NA	NA	NA	334631-R	AU	6324	0.16	0.00E+00	rRNA	rrn26	reverse	
334696	334696-F	CU	2884	0.14	4.6E-113	NA	NA	NA	NA	NA	rRNA	rrn26	reverse	
334721	NA	NA	NA	NA	NA	334721-R	CU	5180	0.16	8.45E-243	rRNA	rrn26	reverse	
334969	NA	NA	NA	NA	NA	334969-R	UG	281	0.33	5.95E-31	intergenic	NA	NA	

335248	NA	NA	NA	NA	NA	335248-R	GU	150	0.1	0.00018788	intergenic	NA	NA
335335	335335-F	CU	179	0.1	2.54E-05	NA	NA	NA	NA	NA	intergenic	NA	NA
335346	NA	NA	NA	NA	NA	335346-R	GU	105	0.12	0.00065192	intergenic	NA	NA
335347	NA	NA	NA	NA	NA	335347-R	CU	90	0.2	1.63E-05	intergenic	NA	NA
335479	335479-F	CU	65	0.69	5.23E-18	335479-R	GA	33	0.79	5.80E-11	intergenic	NA	NA
335480	335480-F	AC	58	0.69	6.43E-16	335480-R	UG	32	0.78	1.81E-10	intergenic	NA	NA
335482	335482-F	GU	58	0.31	9.33E-06	NA	NA	NA	NA	NA	intergenic	NA	NA
335485	335485-F	GA	52	0.62	4.75E-12	335485-R	CU	28	0.75	1.57E-08	intergenic	NA	NA
335486	335486-F	GA	49	0.61	2.98E-11	335486-R	CU	26	0.73	1.38E-07	intergenic	NA	NA
335488	335488-F	CG	7	1	0.002331	NA	NA	NA	NA	NA	intergenic	NA	NA
335491	335491-F	GC	6	1	0.007576	NA	NA	NA	NA	NA	intergenic	NA	NA
342250	342250-F	AG	12	1	1.7E-05	NA	NA	NA	NA	NA	intergenic	NA	NA
342252	342252-F	UA	13	0.92	1.63E-05	NA	NA	NA	NA	NA	intergenic	NA	NA
342256	342256-F	GU	31	0.93	1.14E-13	NA	NA	NA	NA	NA	intergenic	NA	NA
342289	342289-F	CU	57	1	3.75E-32	342289-R	GA	27	0.96	3.75E-13	intergenic	NA	NA
342317	342317-F	UC	56	0.98	8.03E-30	342317-R	AG	146	0.99	5.74E-83	intergenic	NA	NA
342396	NA	NA	NA	NA	NA	orf281-71-as	CU	6	1	0.02380952	exon	orf281	forward
342411	orf281-86	CU	10	1	5.95E-05	NA	NA	NA	NA	NA	exon	orf281	forward
342436	orf281-111	CU	10	1	5.95E-05	NA	NA	NA	NA	NA	exon	orf281	forward
342440	orf281-115	AG	10	1	5.95E-05	NA	NA	NA	NA	NA	exon	orf281	forward
342472	orf281-147	GA	10	1	5.95E-05	NA	NA	NA	NA	NA	exon	orf281	forward
343698	cox1-242	CU	59	1	2.46E-33	NA	NA	NA	NA	NA	exon	cox1	forward
343710	cox1-254	CU	75	0.97	2.27E-39	NA	NA	NA	NA	NA	exon	cox1	forward
343721	cox1-265	CU	81	0.11	0.008904	NA	NA	NA	NA	NA	exon	cox1	forward
343908	cox1-452	CU	20	1	1.52E-10	cox1-452-as	GA	8	1	0.0006993	exon	cox1	forward
343971	cox1-515	CU	27	1	1.44E-14	cox1-515-as	GA	7	1	0.002331	exon	cox1	forward

344007	cox1-551	CU	16	1	2.83E-08	cox1-551-as	GA	29	1	9.98E-16	exon	cox1	forward
344046	cox1-590	CU	7	1	0.002331	cox1-590-as	GA	33	1	4.71E-18	exon	cox1	forward
344338	NA	NA	NA	NA	NA	orf304-75-as	UC	18	0.44	0.00888289	exon	orf304	forward
344358	NA	NA	NA	NA	NA	orf304-95-as	CU	21	0.52	0.00069641	exon	orf304	forward
344360	NA	NA	NA	NA	NA	orf304-97-as	UC	21	0.52	0.00069641	exon	orf304	forward
344372	NA	NA	NA	NA	NA	orf304-109-as	UA	19	0.53	0.00152142	exon	orf304	forward
344441	orf304-178	AC	14	1	3.74E-07	NA	NA	NA	NA	NA	exon	orf304	forward
344453	orf304-190	CA	14	1	3.74E-07	NA	NA	NA	NA	NA	exon	orf304	forward
344456	orf304-193	AC	14	1	3.74E-07	orf304-193-as	UG	20	1	1.52E-10	exon	orf304	forward
344502	orf304-239	CA	12	1	4.81E-06	orf304-239-as	GU	20	1	1.52E-10	exon	orf304	forward
344503	orf304-240	AG	12	1	4.81E-06	orf304-240-as	UC	20	1	1.52E-10	exon	orf304	forward
344530	orf304-267	UC	11	1	5.95E-05	orf304-267-as	AG	20	1	1.52E-10	exon	orf304	forward
344551	NA	NA	NA	NA	NA	orf304-288-as	UC	23	0.91	2.54E-09	exon	orf304	forward
344618	NA	NA	NA	NA	NA	orf304-355-as	UC	13	0.62	0.00558352	exon	orf304	forward
344647	orf304-384	AG	9	0.89	0.001687	orf304-384-as	UC	13	0.54	0.01510297	exon	orf304	forward
344650	orf304-387	AG	10	0.8	0.002739	NA	NA	NA	NA	NA	exon	orf304	forward
344676	orf304-413	UA	13	1	1.35E-06	orf304-413-as	AU	9	1	0.00020568	exon	orf304	forward
344756	orf304-493	AG	20	1	1.52E-10	orf304-493-as	UC	9	1	0.00020568	exon	orf304	forward
344777	orf304-514	CG	19	1	5.66E-10	orf304-514-as	GC	15	1	1.03E-07	exon	orf304	forward
344782	orf304-519	AG	19	0.42	0.009376	orf304-519-as	UC	14	0.64	0.00221205	exon	orf304	forward
344783	orf304-520	CG	19	0.53	0.001521	NA	NA	NA	NA	NA	exon	orf304	forward
344806	orf304-543	CA	18	1	2.09E-09	orf304-543-as	GU	7	1	0.002331	exon	orf304	forward
344807	orf304-544	AG	18	0.5	0.003615	NA	NA	NA	NA	NA	exon	orf304	forward
344876	orf304-613	AG	15	1	1.03E-07	orf304-613-as	UC	15	1	1.03E-07	exon	orf304	forward
344880	orf304-617	AG	15	1	1.03E-07	orf304-617-as	UC	21	1	4.09E-11	exon	orf304	forward
344912	orf304-649	CA	17	1	7.71E-09	orf304-649-as	GU	18	1	2.09E-09	exon	orf304	forward

344956	orf304-693	UA	16	1	2.83E-08	orf304-693-as	AU	10	1	5.95E-05	exon	orf304	forward	
344978	orf304-715	CG	8	1	0.002331	orf304-715-as	GC	5	1	0.02380952	exon	orf304	forward	
344993	orf304-730	GA	11	1	1.7E-05	orf304-730-as	CU	8	1	0.0006993	exon	orf304	forward	
345019	orf304-756	CU	18	1	2.09E-09	orf304-756-as	GA	7	1	0.002331	exon	orf304	forward	
345020	orf304-757	AG	16	1	2.83E-08	orf304-757-as	UC	7	1	0.002331	exon	orf304	forward	
345037	orf304-774	CU	16	1	2.83E-08	orf304-774-as	GA	7	1	0.002331	exon	orf304	forward	
345043	orf304-780	UC	16	0.69	0.000318	NA	NA	NA	NA	NA	exon	orf304	forward	
345061	orf304-798	AU	15	0.67	0.00085	NA	NA	NA	NA	NA	exon	orf304	forward	
345092	orf304-829	AG	10	1	5.95E-05	NA	NA	NA	NA	NA	exon	orf304	forward	
345171	cox1-754	CG	5	1	0.02381	NA	NA	NA	NA	NA	exon	cox1	forward	
345207	cox1-790	UC	15	0.53	0.007096	NA	NA	NA	NA	NA	exon	cox1	forward	
345472	NA	NA	NA	NA	NA	cox1-1055-as	AU	21	0.33	0.02241266	exon	cox1	forward	
345822	cox1-1405	CU	21	1	4.09E-11	cox1-1405-as	GA	47	1	2.95E-26	exon	cox1	forward	
345830	NA	NA	NA	NA	NA	cox1-1413-as	GA	47	0.21	0.00377719	exon	cox1	forward	
345850	cox1-1433	CU	30	0.9	1.18E-12	cox1-1433-as	GA	49	0.96	2.31E-24	exon	cox1	forward	
345906	cox1-1489	CU	27	0.89	4.71E-11	cox1-1489-as	GA	57	0.95	1.16E-27	exon	cox1	forward	
345911	NA	NA	NA	NA	NA	cox1-1494-as	GA	61	0.13	0.01615009	exon	cox1	forward	
345916	cox1-1499	CU	25	0.88	5.35E-10	NA	NA	NA	NA	NA	exon	cox1	forward	
345917	NA	NA	NA	NA	NA	cox1-1500-as	UC	34	0.21	0.02720007	exon	cox1	forward	
345969	NA	NA	NA	NA	NA	cox1-1552-as	CA	31	0.26	0.01304855	exon	cox1	forward	
345994	NA	NA	NA	NA	NA	cox1-1577-as	AG	24	0.42	0.00219366	exon	cox1	forward	
346452	orf317-192	GU	62	0.39	5.92E-08	NA	NA	NA	NA	NA	exon	orf317	forward	
347384	NA	NA	NA	NA	NA	347384-R	CU	26	0.68	1.52E-06	intergenic	NA	NA	
370518	370518-F	UC	25	0.96	4.95E-12	NA	NA	NA	NA	NA	intergenic	NA	NA	Yes
370536	370536-F	CG	29	0.96	1.03E-13	NA	NA	NA	NA	NA	intergenic	NA	NA	Yes
370778	NA	NA	NA	NA	NA	370778-R	GA	6	1	0.00757576	intergenic	NA	NA	

370814	NA	NA	NA	NA	NA	370814-R	UC	12	0.92	5.36E-05	intergenic	NA	NA		
370818	NA	NA	NA	NA	NA	370818-R	UC	12	0.75	0.00137973	intergenic	NA	NA		
370834	NA	NA	NA	NA	NA	370834-R	UC	14	0.93	4.91E-06	intergenic	NA	NA		
370972	370972-F	UG	5	1	0.02381	NA	NA	NA	NA	NA	intergenic	NA	NA		
371077	371077-F	AU	5	1	0.02381	NA	NA	NA	NA	NA	intergenic	NA	NA		
371211	371211-F	CU	35	0.8	5.85E-12	NA	NA	NA	NA	NA	intergenic	NA	NA		Yes
371218	371218-F	UC	26	0.31	0.01189	NA	NA	NA	NA	NA	intergenic	NA	NA		
371219	371219-F	AU	25	0.28	0.024399	NA	NA	NA	NA	NA	intergenic	NA	NA		
371221	371221-F	GU	17	0.65	0.000395	NA	NA	NA	NA	NA	intergenic	NA	NA		
371501	NA	NA	NA	NA	NA	371501-R	UC	18	1	2.09E-09	intergenic	NA	NA		
371503	NA	NA	NA	NA	NA	371503-R	GU	18	1	2.09E-09	intergenic	NA	NA		Yes
371515	NA	NA	NA	NA	NA	371515-R	UC	18	1	2.09E-09	intergenic	NA	NA		
371517	NA	NA	NA	NA	NA	371517-R	AG	18	1	2.09E-09	intergenic	NA	NA		
371523	NA	NA	NA	NA	NA	371523-R	GA	18	1	2.09E-09	intergenic	NA	NA		
371554	NA	NA	NA	NA	NA	371554-R	CA	11	0.78	0.00761004	intergenic	NA	NA		
375613	NA	NA	NA	NA	NA	375613-R	AG	6	1	0.00757576	intergenic	NA	NA		
378330	378330-F	CU	48	1	7.61E-27	NA	NA	NA	NA	NA	intergenic	NA	NA		
378331	378331-F	UC	48	0.52	7.37E-09	NA	NA	NA	NA	NA	intergenic	NA	NA		
378332	378332-F	UG	48	0.52	7.37E-09	NA	NA	NA	NA	NA	intergenic	NA	NA		
378709	NA	NA	NA	NA	NA	378709-R	CU	6243	0.12	3.42E-206	intergenic	NA	NA		
378712	NA	NA	NA	NA	NA	378712-R	CU	6131	0.2	0	intergenic	NA	NA		
378766	378766-F	AU	56	0.16	0.008126	NA	NA	NA	NA	NA	intergenic	NA	NA		
378786	NA	NA	NA	NA	NA	378786-R	AG	368	0.26	2.37E-29	intergenic	NA	NA		
378956	378956-F	AC	105	1	1.17E-60	NA	NA	NA	NA	NA	intergenic	NA	NA		
378958	378958-F	GU	91	1	2.54E-52	NA	NA	NA	NA	NA	intergenic	NA	NA		
378960	378960-F	CU	51	1	1.3E-28	NA	NA	NA	NA	NA	intergenic	NA	NA		

378961	378961-F	UA	50	1	5.05E-28	NA	NA	NA	NA	NA	intergenic	NA	NA	
378962	378962-F	UG	25	1	2.06E-13	NA	NA	NA	NA	NA	intergenic	NA	NA	
384671	NA	NA	NA	NA	NA	384671-R	UC	10	1	5.95E-05	intergenic	NA	NA	Yes
385810	385810-F	CU	6	1	0.007576	NA	NA	NA	NA	NA	intergenic	NA	NA	
388881	388881-F	AU	25	0.68	1.52E-06	NA	NA	NA	NA	NA	intergenic	NA	NA	
389029	NA	NA	NA	NA	NA	389029-R	CG	33	0.52	5.77E-06	intergenic	NA	NA	
395101	rps12-338-as	CU	29	0.39	0.001131	NA	NA	NA	NA	NA	exon	rps12	reverse	
395155	rps12-284-as	GA	36	1	8.36E-20	rps12-284	CU	14	1	3.74E-07	exon	rps12	reverse	
395218	rps12-221-as	GA	21	1	4.09E-11	rps12-221	CU	22	1	1.09E-11	exon	rps12	reverse	
395233	rps12-206-as	CA	50	0.23	0.001848	NA	NA	NA	NA	NA	exon	rps12	reverse	
395243	rps12-196-as	GA	44	1	1.71E-24	rps12-196	CU	26	1	5.44E-14	exon	rps12	reverse	
395280	rps12-159-as	GA	42	0.98	1.05E-21	rps12-159	CU	29	0.9	4.05E-12	exon	rps12	reverse	
395302	NA	NA	NA	NA	NA	rps12-137	AU	33	0.21	0.02696262	exon	rps12	reverse	
395335	NA	NA	NA	NA	NA	rps12-104	CU	47	1	2.95E-26	exon	rps12	reverse	
395344	rps12-95-as	CU	23	0.91	7.4E-10	NA	NA	NA	NA	NA	exon	rps12	reverse	
395358	rps12-81-as	AG	95	0.11	0.004766	NA	NA	NA	NA	NA	exon	rps12	reverse	
395403	NA	NA	NA	NA	NA	rps12-36	AU	30	0.37	0.00123293	exon	rps12	reverse	
395454	395454-F	CU	110	0.15	8.12E-05	NA	NA	NA	NA	NA	intergenic	NA	NA	
395461	395461-F	CU	280	0.12	7.84E-10	NA	NA	NA	NA	NA	intergenic	NA	NA	
395495	nad3-349-as	GA	351	0.98	1.5E-195	nad3-349	CU	113	1	1.99E-65	exon	nad3	reverse	
395500	nad3-344-as	GA	346	0.99	3.8E-198	nad3-344	CU	112	1	7.87E-65	exon	nad3	reverse	
395527	nad3-317-as	GA	323	1	1.1E-188	nad3-317	CU	141	0.99	3.80E-78	exon	nad3	reverse	
395569	nad3-275-as	GA	56	1	1.46E-31	nad3-275	CU	132	0.98	3.51E-71	exon	nad3	reverse	
395578	nad3-266-as	GA	15	1	1.03E-07	nad3-266	CU	115	0.97	9.48E-60	exon	nad3	reverse	
395593	NA	NA	NA	NA	NA	nad3-251	CU	120	0.96	2.87E-61	exon	nad3	reverse	
395597	NA	NA	NA	NA	NA	nad3-247	CU	124	0.92	1.70E-57	exon	nad3	reverse	

395613	NA	NA	NA	NA	NA	nad3-231	CU	90	0.5	5.16E-16	exon	nad3	reverse	
395614	nad3-230-as	GA	21	0.95	8.21E-10	nad3-230	CU	85	0.96	9.62E-44	exon	nad3	reverse	
395629	nad3-215-as	GA	20	0.95	2.91E-09	nad3-215	CU	53	0.96	1.19E-26	exon	nad3	reverse	
395635	nad3-209-as	GA	21	0.95	8.21E-10	nad3-209	CU	53	1	8.61E-30	exon	nad3	reverse	
395636	nad3-208-as	GA	21	0.95	8.21E-10	nad3-208	CU	53	1	8.61E-30	exon	nad3	reverse	
395698	nad3-146-as	GA	21	0.95	8.21E-10	nad3-146	CU	38	0.97	2.10E-19	exon	nad3	reverse	
395720	nad3-124-as	GA	7	1	0.002331	nad3-124	CU	21	0.95	8.21E-10	exon	nad3	reverse	
395764	nad3-80-as	GA	11	1	1.7E-05	nad3-80	CU	27	1	1.44E-14	exon	nad3	reverse	
395765	nad3-79-as	GA	12	1	4.81E-06	nad3-79	CU	27	1	1.44E-14	exon	nad3	reverse	
395782	nad3-62-as	GA	9	1	0.000206	nad3-62	CU	25	1	2.06E-13	exon	nad3	reverse	
395800	nad3-44-as	GA	5	1	0.02381	nad3-44	CU	26	1	5.44E-14	exon	nad3	reverse	
406188	406188-F	GU	6	1	0.007576	NA	NA	NA	NA	NA	intergenic	NA	NA	
406190	406190-F	CG	6	1	0.007576	NA	NA	NA	NA	NA	intergenic	NA	NA	
406191	406191-F	UC	6	1	0.007576	NA	NA	NA	NA	NA	intergenic	NA	NA	
406249	406249-F	UC	11	0.82	0.000953	406249-R	AG	9	1	0.00020568	intergenic	NA	NA	Yes
406280	406280-F	AC	10	0.8	0.002739	406280-R	UG	12	0.83	0.0003221	intergenic	NA	NA	Yes
406286	406286-F	CU	11	0.82	0.000953	406286-R	GA	12	0.83	0.0003221	intergenic	NA	NA	Yes
406319	NA	NA	NA	NA	NA	406319-R	UG	11	0.82	0.00095261	intergenic	NA	NA	Yes
406324	NA	NA	NA	NA	NA	406324-R	GA	9	0.78	0.00761004	intergenic	NA	NA	Yes
406336	NA	NA	NA	NA	NA	406336-R	GA	8	0.75	0.02027972	intergenic	NA	NA	Yes
411855	rpl5-35	CU	6	1	0.007576	NA	NA	NA	NA	NA	exon	rpl5	forward	
411867	rpl5-47	CU	6	1	0.007576	NA	NA	NA	NA	NA	exon	rpl5	forward	
411980	NA	NA	NA	NA	NA	rpl5-160-as	GA	7	1	0.002331	exon	rpl5	forward	
412234	rpl5-414	CU	90	0.62	1.57E-21	rpl5-414-as	GA	24	0.38	0.00509751	exon	rpl5	forward	
412255	rpl5-435	CU	82	0.7	5.11E-23	NA	NA	NA	NA	NA	exon	rpl5	forward	
412261	rpl5-441	CU	81	1	2.24E-46	rpl5-441-as	GA	34	1	1.23E-18	exon	rpl5	forward	

412329	NA	NA	NA	NA	NA	rpl5-509-as	GA	89	1	3.93E-51	exon	rpl5	forward
412331	NA	NA	NA	NA	NA	rpl5-511-as	GA	90	0.14	0.0006132	exon	rpl5	forward
412332	NA	NA	NA	NA	NA	rpl5-512-as	GA	90	1	10.00E-52	exon	rpl5	forward
412357	NA	NA	NA	NA	NA	rpl5-537-as	AG	77	0.14	0.00230339	exon	rpl5	forward
412373	NA	NA	NA	NA	NA	rpl5-553-as	AG	57	0.19	0.00399172	exon	rpl5	forward
412374	NA	NA	NA	NA	NA	rpl5-554-as	UC	56	0.18	0.00408341	exon	rpl5	forward
412375	NA	NA	NA	NA	NA	rpl5-555-as	UC	55	0.23	0.00092918	exon	rpl5	forward
420824	orf214-400-as	CA	57	0.2	0.002014	NA	NA	NA	NA	NA	exon	orf214	reverse
420825	orf214-399-as	AU	210	0.2	1.06E-11	NA	NA	NA	NA	NA	exon	orf214	reverse
420826	orf214-398-as	UA	474	0.1	8.45E-13	NA	NA	NA	NA	NA	exon	orf214	reverse
420827	orf214-397-as	CU	591	0.13	3.09E-21	NA	NA	NA	NA	NA	exon	orf214	reverse
420919	orf214-305-as	GA	621	0.68	2.2E-173	orf214-305	CU	543	0.71	7.98E-164	exon	orf214	reverse
421209	orf214-15-as	AU	74	0.14	0.004415	NA	NA	NA	NA	NA	exon	orf214	reverse
421266	atp9-212-as	GA	76	1	2.09E-43	atp9-212	CU	14	1	3.74E-07	exon	atp9	reverse
426294	NA	NA	NA	NA	NA	426294-R	GA	7	1	0.002331	intergenic	NA	NA
427622	nad7-77	CU	17	1	7.71E-09	nad7-77-as	GA	17	0.94	1.24E-07	exon	nad7	forward
427682	nad7-137	CU	5	1	0.02381	nad7-137-as	GA	12	1	4.81E-06	exon	nad7	forward
427689	NA	NA	NA	NA	NA	427689-R	CU	12	1	1.701e-005	intron	nad7	forward
427690	NA	NA	NA	NA	NA	427690-R	AU	12	1	4.81E-06	intron	nad7	forward
427692	NA	NA	NA	NA	NA	427692-R	GC	12	1	4.81E-06	intron	nad7	forward
428182	NA	NA	NA	NA	NA	orf103b-283-as	CA	60	0.17	0.00419266	exon	orf103b	forward
428248	NA	NA	NA	NA	NA	428248-R	CU	14	0.79	0.0001705	intron	nad7	forward
428608	nad7-200	CU	8	1	0.000699	NA	NA	NA	NA	NA	exon	nad7	forward
428620	nad7-212	AU	10	0.78	0.00761	NA	NA	NA	NA	NA	exon	nad7	forward
428948	428948-F	AU	18	0.5	0.003615	NA	NA	NA	NA	NA	intron	nad7	forward
429001	NA	NA	NA	NA	NA	429001-R	CU	20	0.5	0.00167103	intron	nad7	forward

429004	NA	NA	NA	NA	NA	429004-R	CG	21	0.52	0.00069641	intron	nad7	forward
429186	NA	NA	NA	NA	NA	429186-R	GU	39	0.23	0.00707329	intron	nad7	forward
429311	NA	NA	NA	NA	NA	429311-R	UC	50	0.16	0.01542891	intron	nad7	forward
429312	NA	NA	NA	NA	NA	429312-R	UG	50	0.16	0.01542891	intron	nad7	forward
429920	429920-F	GA	8	1	0.000699	429920-R	CU	13	0.62	0.00558352	intron	nad7	forward
430080	NA	NA	NA	NA	NA	nad7-347-as	AG	15	0.73	0.00024198	exon	nad7	forward
430188	nad7-455	GU	62	0.23	0.000238	NA	NA	NA	NA	NA	exon	nad7	forward
430189	nad7-456	GA	59	0.66	3.28E-15	nad7-456-as	CU	16	1	2.83E-08	exon	nad7	forward
430264	nad7-531	CU	33	0.52	5.77E-06	NA	NA	NA	NA	NA	exon	nad7	forward
430280	NA	NA	NA	NA	NA	nad7-547-as	AC	36	0.31	0.00145103	exon	nad7	forward
430281	NA	NA	NA	NA	NA	nad7-548-as	GU	35	0.34	0.0006464	exon	nad7	forward
430282	NA	NA	NA	NA	NA	nad7-549-as	AU	36	0.31	0.00145103	exon	nad7	forward
430283	NA	NA	NA	NA	NA	nad7-550-as	AC	35	0.32	0.0014111	exon	nad7	forward
430284	NA	NA	NA	NA	NA	nad7-551-as	GU	37	0.3	0.00152602	exon	nad7	forward
430285	NA	NA	NA	NA	NA	nad7-552-as	AU	35	0.31	0.00145103	exon	nad7	forward
430311	NA	NA	NA	NA	NA	nad7-578-as	GA	23	1	2.92E-12	exon	nad7	forward
430457	nad7-724	CU	20	1	1.52E-10	nad7-724-as	GA	11	1	1.701e-005	exon	nad7	forward
430472	nad7-739	CU	20	1	1.52E-10	nad7-739-as	GA	7	0.86	0.01456876	exon	nad7	forward
430473	nad7-740	CU	20	1	1.52E-10	nad7-740-as	GA	6	1	0.00757576	exon	nad7	forward
431083	NA	NA	NA	NA	NA	431083-R	AG	43	0.53	1.98E-07	intron	nad7	forward
431873	431873-F	CU	14	1	3.74E-07	431873-R	GA	15	1	1.03E-07	intron	nad7	forward
432125	nad7-1050	CU	24	0.92	2.15E-10	nad7-1050-as	GA	21	1	4.09E-11	exon	nad7	forward
432132	nad7-1057	CU	17	0.71	0.000116	nad7-1057-as	GA	19	1	5.66E-10	exon	nad7	forward
432178	nad7-1103	CU	8	0.88	0.005051	nad7-1103-as	GA	22	1	1.09E-11	exon	nad7	forward
432199	NA	NA	NA	NA	NA	nad7-1124-as	GA	25	0.88	5.35E-10	exon	nad7	forward
432241	NA	NA	NA	NA	NA	nad7-1166-as	GA	22	0.91	2.54E-09	exon	nad7	forward

434162	orf108b-201	GA	8	1	0.000699	NA	NA	NA	NA	NA	exon	orf108b	forward
434175	orf108b-214	CU	8	1	0.000699	NA	NA	NA	NA	NA	exon	orf108b	forward
434198	orf108b-237	GA	8	1	0.000699	NA	NA	NA	NA	NA	exon	orf108b	forward
434203	orf108b-242	CU	8	1	0.000699	NA	NA	NA	NA	NA	exon	orf108b	forward
434240	orf108b-279	CG	8	1	0.000699	orf108b-279-as	GC	8	1	0.0006993	exon	orf108b	forward
434255	orf108b-294	AU	8	1	0.000699	orf108b-294-as	UA	8	1	0.0006993	exon	orf108b	forward
434256	orf108b-295	AG	8	1	0.000699	orf108b-295-as	UC	8	1	0.0006993	exon	orf108b	forward
434279	NA	NA	NA	NA	NA	orf108b-318-as	GA	8	1	0.0006993	exon	orf108b	forward
434280	NA	NA	NA	NA	NA	orf108b-319-as	CU	8	1	0.0006993	exon	orf108b	forward
434296	NA	NA	NA	NA	NA	434296-R	GA	8	1	0.0006993	intergenic	NA	NA
434300	NA	NA	NA	NA	NA	434300-R	UG	8	1	0.0006993	intergenic	NA	NA
435089	435089-F	UC	103	0.13	0.000633	NA	NA	NA	NA	NA	intergenic	NA	NA
435237	435237-F	AU	251	0.12	4.99E-08	NA	NA	NA	NA	NA	intergenic	NA	NA
435240	435240-F	UC	854	0.12	1.26E-27	NA	NA	NA	NA	NA	intergenic	NA	NA
435241	435241-F	CU	1510	0.16	3.57E-69	NA	NA	NA	NA	NA	intergenic	NA	NA
447122	NA	NA	NA	NA	NA	447122-R	CU	8	1	0.0006993	intergenic	NA	NA
450885	nad1-536-as	GA	9	1	0.000206	NA	NA	NA	NA	NA	exon	nad1	reverse
450921	nad1-500-as	GA	12	1	4.81E-06	NA	NA	NA	NA	NA	exon	nad1	reverse
450928	nad1-493-as	GA	11	1	1.7E-05	NA	NA	NA	NA	NA	exon	nad1	reverse
450931	nad1-490-as	GA	11	1	1.7E-05	NA	NA	NA	NA	NA	exon	nad1	reverse
452265	NA	NA	NA	NA	NA	nad1-401	CU	6	1	0.00757576	exon	nad1	reverse
453020	NA	NA	NA	NA	NA	453020-R	CU	6	1	0.00757576	intergenic	NA	NA
460734	460734-F	AU	21	1	4.09E-11	NA	NA	NA	NA	NA	intergenic	NA	NA
468194	NA	NA	NA	NA	NA	orf456-1352	UG	6	1	0.00757576	exon	orf456	reverse
468217	orf456-1329-as	AG	12	1	4.81E-06	orf456-1329	UC	10	1	5.95E-05	exon	orf456	reverse
468259	orf456-1287-as	AG	18	1	2.09E-09	orf456-1287	UC	49	1	1.96E-27	exon	orf456	reverse

468286	orf456-1260-as	CU	22	1	1.09E-11	orf456-1260	GA	73	1	1.26E-41	exon	orf456	reverse
468310	orf456-1236-as	GA	30	0.3	0.006091	orf456-1236	CU	73	0.45	2.32E-11	exon	orf456	reverse
468316	orf456-1230-as	UC	18	1	2.09E-09	orf456-1230	AG	80	1	8.80E-46	exon	orf456	reverse
468361	orf456-1185-as	GA	15	1	1.03E-07	orf456-1185	CU	56	1	1.46E-31	exon	orf456	reverse
468364	orf456-1182-as	GC	15	1	1.03E-07	orf456-1182	CG	53	1	8.61E-30	exon	orf456	reverse
468449	orf456-1097-as	AU	47	1	2.95E-26	orf456-1097	UA	20	1	1.52E-10	exon	orf456	reverse
468454	orf456-1092-as	GC	46	1	1.14E-25	orf456-1092	CG	21	0.81	3.59E-07	exon	orf456	reverse
468456	orf456-1090-as	GA	47	1	2.95E-26	orf456-1090	CU	19	1	5.66E-10	exon	orf456	reverse
468474	orf456-1072-as	GA	50	1	5.05E-28	orf456-1072	CU	18	1	2.09E-09	exon	orf456	reverse
468511	orf456-1035-as	AU	26	1	5.44E-14	orf456-1035	UA	23	1	2.92E-12	exon	orf456	reverse
468523	orf456-1023-as	AG	16	1	2.83E-08	orf456-1023	UC	23	1	2.92E-12	exon	orf456	reverse
468526	orf456-1020-as	UC	17	0.94	1.24E-07	orf456-1020	AG	24	1	7.75E-13	exon	orf456	reverse
468541	orf456-1005-as	UA	100	1	1.12E-57	orf456-1005	AU	18	1	2.09E-09	exon	orf456	reverse
468562	orf456-984-as	AG	93	1	1.64E-53	orf456-984	UC	19	1	5.66E-10	exon	orf456	reverse
468578	orf456-968-as	CU	87	1	6.08E-50	orf456-968	GA	22	1	1.09E-11	exon	orf456	reverse
468603	orf456-943-as	UG	82	1	5.71E-47	orf456-943	AC	8	1	0.0006993	exon	orf456	reverse
468627	orf456-919-as	CU	104	0.98	2.47E-56	orf456-919	GA	54	1	2.21E-30	exon	orf456	reverse
468649	orf456-897-as	GA	74	1	3.21E-42	orf456-897	CU	69	1	2.96E-39	exon	orf456	reverse
468655	orf456-891-as	CU	76	1	2.09E-43	orf456-891	GA	72	1	4.93E-41	exon	orf456	reverse
468684	orf456-862-as	GU	84	1	3.7E-48	orf456-862	CA	84	1	3.70E-48	exon	orf456	reverse
468685	orf456-861-as	UC	85	1	9.4E-49	orf456-861	AG	88	1	1.55E-50	exon	orf456	reverse
468707	orf456-839-as	UG	56	1	1.46E-31	orf456-839	AC	75	1	8.19E-43	exon	orf456	reverse
468721	orf456-825-as	UC	29	1	9.98E-16	orf456-825	AG	60	1	6.31E-34	exon	orf456	reverse
468748	orf456-798-as	UC	16	1	2.83E-08	orf456-798	AG	47	1	2.95E-26	exon	orf456	reverse
468751	orf456-795-as	AG	16	1	2.83E-08	orf456-795	UC	51	1	1.30E-28	exon	orf456	reverse
468760	NA	NA	NA	NA	NA	orf456-786	UG	54	1	2.21E-30	exon	orf456	reverse

468787	orf456-759-as	UC	861	1	0	orf456-759	AG	29	0.97	2.80E-14	exon	orf456	reverse
468805	orf456-741-as	CU	896	0.27	2.9E-79	orf456-741	GA	69	0.45	1.22E-10	exon	orf456	reverse
468817	orf456-729-as	AG	884	0.99	0	orf456-729	UC	132	0.99	1.20E-74	exon	orf456	reverse
468827	orf456-719-as	CU	889	0.99	0	orf456-719	GA	157	0.99	1.64E-89	exon	orf456	reverse
468838	orf456-708-as	UA	893	0.99	0	orf456-708	AU	290	0.99	9.31E-167	exon	orf456	reverse
468862	orf456-684-as	GA	426	0.98	3.1E-233	orf456-684	CU	538	1	3.955e-315	exon	orf456	reverse
468888	orf456-658-as	GC	67	0.22	0.000119	NA	NA	NA	NA	NA	exon	orf456	reverse
468892	orf456-654-as	AG	56	0.73	7.81E-17	orf456-654	UC	825	1	0	exon	orf456	reverse
468910	orf456-636-as	AG	26	0.77	3.15E-08	orf456-636	UC	921	1	0	exon	orf456	reverse
468915	orf456-631-as	UG	26	0.77	3.15E-08	NA	NA	NA	NA	NA	exon	orf456	reverse
468916	orf456-630-as	AG	26	0.77	3.15E-08	orf456-630	UC	965	1	0	exon	orf456	reverse
468940	orf456-606-as	GU	27	0.78	1.03E-08	orf456-606	CA	718	1	0	exon	orf456	reverse
469051	orf456-495-as	AC	11	1	1.7E-05	orf456-495	UG	56	0.98	8.03E-30	exon	orf456	reverse
469072	orf456-474-as	GA	21	1	4.09E-11	orf456-474	CU	37	0.97	7.85E-19	exon	orf456	reverse
469099	orf456-447-as	GC	18	1	2.09E-09	orf456-447	CG	13	1	1.35E-06	exon	orf456	reverse
469112	orf456-434-as	GC	14	1	3.74E-07	orf456-434	CG	7	1	0.002331	exon	orf456	reverse
469132	orf456-414-as	CU	28	1	1.44E-14	orf456-414	GA	12	1	4.81E-06	exon	orf456	reverse
469135	orf456-411-as	UC	27	0.96	3.75E-13	orf456-411	AG	12	1	4.81E-06	exon	orf456	reverse
469141	orf456-405-as	CU	36	0.97	2.93E-18	orf456-405	GA	14	1	3.74E-07	exon	orf456	reverse
469152	orf456-394-as	AG	46	1	1.14E-25	orf456-394	UC	20	1	1.52E-10	exon	orf456	reverse
469171	orf456-375-as	GA	36	0.92	6.5E-16	orf456-375	CU	57	1	3.75E-32	exon	orf456	reverse
469189	orf456-357-as	UA	35	0.91	2.29E-15	orf456-357	AU	69	1	2.96E-39	exon	orf456	reverse
469194	orf456-352-as	CA	35	0.23	0.013752	NA	NA	NA	NA	NA	exon	orf456	reverse
469246	orf456-300-as	UA	27	0.89	4.71E-11	orf456-300	AU	68	1	1.16E-38	exon	orf456	reverse
469260	orf456-286-as	UC	23	0.91	7.4E-10	NA	NA	NA	NA	NA	exon	orf456	reverse
469297	orf456-249-as	UA	20	1	1.52E-10	orf456-249	AU	24	0.88	1.78E-09	exon	orf456	reverse

469303	orf456-243-as	CU	21	1	4.09E-11	orf456-243	GA	24	0.88	1.78E-09	exon	orf456	reverse
469318	orf456-228-as	CU	5	1	0.02381	orf456-228	GA	28	0.89	1.39E-11	exon	orf456	reverse
469323	orf456-223-as	UC	6	1	0.007576	orf456-223	AG	28	0.89	1.39E-11	exon	orf456	reverse
469327	orf456-219-as	CU	6	1	0.007576	orf456-219	GA	28	0.89	1.39E-11	exon	orf456	reverse
469330	orf456-216-as	AG	6	1	0.007576	orf456-216	UC	29	0.86	3.13E-11	exon	orf456	reverse
469356	orf456-190-as	GC	129	1	5.66E-75	orf456-190	CG	57	1	3.75E-32	exon	orf456	reverse
469361	orf456-185-as	AU	138	1	2.39E-80	orf456-185	UA	67	1	4.54E-38	exon	orf456	reverse
469372	orf456-174-as	CU	131	1	3.62E-76	orf456-174	GA	69	1	2.96E-39	exon	orf456	reverse
469423	orf456-123-as	UA	131	1	3.62E-76	orf456-123	AU	72	1	4.93E-41	exon	orf456	reverse
469447	orf456-99-as	GU	44	1	1.71E-24	NA	NA	NA	NA	NA	exon	orf456	reverse
469461	orf456-85-as	AG	44	1	1.71E-24	orf456-85	UC	102	1	7.18E-59	exon	orf456	reverse
469464	orf456-82-as	CU	41	1	9.89E-23	orf456-82	GA	109	1	4.84E-63	exon	orf456	reverse
469471	orf456-75-as	AG	58	1	9.61E-33	orf456-75	UC	115	1	1.28E-66	exon	orf456	reverse
469489	orf456-57-as	AG	60	1	6.31E-34	orf456-57	UC	118	1	2.08E-68	exon	orf456	reverse
469534	orf456-12-as	CA	55	1	5.68E-31	orf456-12	GU	110	1	1.23E-63	exon	orf456	reverse
469537	orf456-9-as	UA	55	1	5.68E-31	orf456-9	AU	106	1	2.97E-61	exon	orf456	reverse
469590	469590-F	UA	152	1	1.03E-88	469590-R	AU	54	1	2.21E-30	intergenic	NA	NA
469629	469629-F	UC	154	1	6.54E-90	469629-R	AG	51	1	1.30E-28	intergenic	NA	NA
469668	469668-F	CU	138	1	2.39E-80	NA	NA	NA	NA	NA	intergenic	NA	NA
469693	469693-F	CU	49	1	1.96E-27	469693-R	GA	107	1	7.53E-62	intergenic	NA	NA
469699	469699-F	AU	48	1	7.61E-27	469699-R	UA	104	1	4.62E-60	intergenic	NA	NA
469702	469702-F	GA	49	1	1.96E-27	469702-R	CU	105	1	1.17E-60	intergenic	NA	NA
469717	469717-F	AG	141	1	3.85E-82	469717-R	UC	104	1	4.62E-60	intergenic	NA	NA
469721	469721-F	AG	141	1	3.85E-82	469721-R	UC	107	1	7.53E-62	intergenic	NA	NA
469755	469755-F	GA	137	1	9.44E-80	469755-R	CU	54	1	2.21E-30	intergenic	NA	NA
469756	469756-F	UC	137	1	9.44E-80	469756-R	AG	51	1	1.30E-28	intergenic	NA	NA

469774	469774-F	CU	98	1	1.73E-56	469774-R	GA	64	1	2.71E-36	intergenic	NA	NA
469810	469810-F	UG	184	1	7.4E-108	469810-R	AC	105	1	4.62E-60	intergenic	NA	NA
469861	469861-F	GA	98	1	1.73E-56	469861-R	CU	157	1	1.05E-91	intergenic	NA	NA
469897	469897-F	UC	52	1	3.35E-29	469897-R	AG	144	0.99	6.39E-80	intergenic	NA	NA
469909	469909-F	CU	12	1	4.81E-06	469909-R	GA	135	1	1.48E-78	intergenic	NA	NA
469918	469918-F	UC	9	1	0.000206	469918-R	AG	118	1	2.08E-68	intergenic	NA	NA
469924	469924-F	AU	8	1	0.000699	469924-R	UA	83	1	1.45E-47	intergenic	NA	NA
469933	469933-F	UC	12	0.92	5.36E-05	469933-R	AG	83	1	1.45E-47	intergenic	NA	NA
469950	469950-F	UA	11	1	1.7E-05	469950-R	AU	46	1	1.14E-25	intergenic	NA	NA
469972	469972-F	GA	11	1	1.7E-05	469972-R	CU	6	1	0.00757576	intergenic	NA	NA
470024	470024-F	AC	5	1	0.02381	NA	NA	NA	NA	NA	intergenic	NA	NA
470666	470666-F	AG	10	0.7	0.009883	NA	NA	NA	NA	NA	intergenic	NA	NA
470667	470667-F	CU	10	0.7	0.009883	NA	NA	NA	NA	NA	intergenic	NA	NA
472504	ccmFn-1519-as	GA	62	1	4.14E-35	ccmFn-1519	CU	10	1	5.95E-05	exon	ccmFn	reverse
472539	ccmFn-1484-as	GA	62	1	4.14E-35	ccmFn-1484	CU	20	0.95	2.91E-09	exon	ccmFn	reverse
472551	ccmFn-1472-as	GA	62	1	4.14E-35	ccmFn-1472	CU	25	0.96	4.95E-12	exon	ccmFn	reverse
472575	NA	NA	NA	NA	NA	ccmFn-1448	CU	54	0.93	8.12E-25	exon	ccmFn	reverse
472636	NA	NA	NA	NA	NA	ccmFn-1387	CU	56	1	1.46E-31	exon	ccmFn	reverse
472669	NA	NA	NA	NA	NA	ccmFn-1354	CU	36	1	8.36E-20	exon	ccmFn	reverse
472702	NA	NA	NA	NA	NA	ccmFn-1321	CU	7	1	0.002331	exon	ccmFn	reverse
472719	NA	NA	NA	NA	NA	ccmFn-1304	CU	6	1	0.00757576	exon	ccmFn	reverse
473214	NA	NA	NA	NA	NA	ccmFn-809	CU	12	1	4.81E-06	exon	ccmFn	reverse
473229	NA	NA	NA	NA	NA	ccmFn-794	CU	16	1	2.83E-08	exon	ccmFn	reverse
473241	NA	NA	NA	NA	NA	ccmFn-782	CU	19	1	5.66E-10	exon	ccmFn	reverse
473263	NA	NA	NA	NA	NA	ccmFn-760	CU	19	0.95	1.02E-08	exon	ccmFn	reverse
473301	NA	NA	NA	NA	NA	ccmFn-722	CU	9	1	0.00020568	exon	ccmFn	reverse

473310	NA	NA	NA	NA	NA	ccmFn-713	CU	7	1	0.002331	exon	ccmFn	reverse	
475035	475035-F	UG	14	0.64	0.002212	NA	NA	NA	NA	NA	intergenic	NA	NA	
476531	NA	NA	NA	NA	NA	476531-R	CG	8	1	0.0006993	intergenic	NA	NA	
483502	483502-F	AG	5	1	0.02381	NA	NA	NA	NA	NA	intergenic	NA	NA	
484293	484293-F	GA	19	0.37	0.021122	NA	NA	NA	NA	NA	intergenic	NA	NA	
484425	cox3-764-as	GA	11	0.82	0.000953	cox3-764	CU	14	0.93	4.91E-06	exon	cox3	reverse	
484435	cox3-754-as	GA	11	0.91	0.000173	cox3-754	CU	9	1	0.00020568	exon	cox3	reverse	
484767	cox3-422-as	GA	94	0.98	1.82E-50	cox3-422	CU	5	1	0.02380952	exon	cox3	reverse	
484770	cox3-419-as	GA	83	1	1.45E-47	cox3-419	CU	9	1	0.00020568	exon	cox3	reverse	
484849	cox3-340-as	CU	63	0.18	0.002115	NA	NA	NA	NA	NA	exon	cox3	reverse	
484850	cox3-339-as	UC	81	0.12	0.008812	NA	NA	NA	NA	NA	exon	cox3	reverse	
484854	cox3-335-as	GA	91	0.31	5.59E-09	NA	NA	NA	NA	NA	exon	cox3	reverse	
484875	cox3-314-as	GA	97	0.85	7.87E-38	cox3-314	CU	90	1	10.00E-52	exon	cox3	reverse	
484878	cox3-311-as	GA	97	0.84	5.44E-37	cox3-311	CU	84	0.99	3.07E-46	exon	cox3	reverse	
484885	cox3-304-as	GA	96	0.85	3.69E-38	cox3-304	CU	69	0.97	6.95E-36	exon	cox3	reverse	
484944	cox3-245-as	GA	88	0.88	1.52E-36	cox3-245	CU	59	0.8	2.11E-20	exon	cox3	reverse	
485015	NA	NA	NA	NA	NA	cox3-174	CU	76	0.14	0.0022929	exon	cox3	reverse	
485731	485731-F	UA	25	0.32	0.011608	NA	NA	NA	NA	NA	intergenic	NA	NA	
485786	NA	NA	NA	NA	NA	atp8-467	GC	42	0.81	9.45E-15	exon	atp8	reverse	
485842	NA	NA	NA	NA	NA	atp8-411	GA	71	0.35	3.68E-08	exon	atp8	reverse	
486195	NA	NA	NA	NA	NA	atp8-58	CU	6	1	0.00757576	exon	atp8	reverse	
486206	NA	NA	NA	NA	NA	atp8-47	CU	5	1	0.02380952	exon	atp8	reverse	
491146	ccmB-325	AG	7	0.86	0.014569	NA	NA	NA	NA	NA	exon	ccmB	forward	
491313	NA	NA	NA	NA	NA	ccmB-492-as	AG	7	0.86	0.01456876	exon	ccmB	forward	

Table S2 List of SNP sites and indels predicted on the mitogenome of *S. miltiorrhiza*.

CHROM	POS	REF	ALT	QUAL	INFO	FORMAT
NC_023209	4144	ATTTT	ATTT	99	INDEL;DP=558;AF1=1;CI95=1,1;DP4=0,0,264,253;MQ=50	PL:DP:SP:GT:GQ 255,255,0:517:0:1/1:99
NC_023209	7198	GAAA	GAA	99	INDEL;DP=441;AF1=1;CI95=1,1;DP4=1,2,11,24;MQ=50;PV4=1,1,1,4.1e-07	PL:DP:SP:GT:GQ 255,69,0:38:0:1/1:99
NC_023209	7201	A	G	99	DP=431;AF1=1;CI95=1,1;DP4=0,2,62,57;MQ=50;PV4=0.24,0.0014,0.4,1	PL:DP:SP:GT:GQ 170,255,0:121:6:1/1:99
NC_023209	8529	G	GA	99	INDEL;DP=485;AF1=1;CI95=1,1;DP4=1,0,34,35;MQ=50;PV4=1,1,0.42,1	PL:DP:SP:GT:GQ 255,192,0:70:0:1/1:99
NC_023209	21156	A	G	88	DP=998;AF1=0.5;CI95=0.5,0.5;DP4=327,294,164,178;MQ=48;PV4=0.18,7e-280,3.3e-228,9.7e-114	PL:DP:SP:GT:GQ 118,0,255:963:7:0/1:99
NC_023209	21188	C	T	99	DP=2364;AF1=0.5;CI95=0.5,0.5;DP4=317,305,836,847;MQ=47;PV4=0.61,0.42,0,1	PL:DP:SP:GT:GQ 255,0,158:2305:2:0/1:99
NC_023209	21349	G	A	66	DP=5160;AF1=0.5;CI95=0.5,0.5;DP4=2717,1684,412,294;MQ=49;PV4=0.088,1,0,1	PL:DP:SP:GT:GQ 96,0,255:5107:11:0/1:99
NC_023209	41862	AAAGGAAAT	A	65.5	INDEL;DP=4216;AF1=0.5;CI95=0.5,0.5;DP4=1095,2583,135,300;MQ=49;PV4=0.58,0,0,1	PL:DP:SP:GT:GQ 103,0,255:4113:2:0/1:99
NC_023209	43239	TTTGTTG	TTTG	99	INDEL;DP=1155;AF1=0.5;CI95=0.5,0.5;DP4=233,220,139,53;MQ=50;PV4=9.2e-07,6.6e-68,1,3e-17	PL:DP:SP:GT:GQ 183,0,255:645:60:0/1:99

NC_023209	43250	C	T	36	DP=678;AF1=0.5;CI95=0.5,0.5;DP4=229,245,144,59;MQ=50;PV4=5.7e-08,3e-152,0.11,7e-61	PL:DP:SP:GT:GQ	66,0,255:677:72:0/1:69
NC_023209	53343	AA	AATGGTGGATACCGTGTGTGA	99	INDEL;DP=744;AF1=1;CI95=1,1;DP4=0,0,405,292;MQ=50	PL:DP:SP:GT:GQ	255,255,0:697:0:1/1:99
NC_023209	66281	CTTTTTTT	CTTTTTTTT,CTTTTTTTTT	99	INDEL;DP=758;AF1=1;CI95=1,1;DP4=2,0,350,309;MQ=50;PV4=0.5,1,0.45,1	PL:DP:SP:GT:GQ	187,255,185,0,255,182:661:3:1/1:99
NC_023209	81097	C	CTAACCAAT	99	INDEL;DP=3401;AF1=0.5;CI95=0.5,0.5;DP4=431,398,993,1118;MQ=46;PV4=0.017,0,1.3e-65,1	PL:DP:SP:GT:GQ	255,0,255:2940:18:0/1:99
NC_023209	81098	CGGG	CAACCAATTGGG	99	INDEL;DP=3391;AF1=0.5;CI95=0.5,0.5;DP4=418,393,1098,1242;MQ=47;PV4=0.025,1,1.4e-54,1	PL:DP:SP:GT:GQ	255,0,203:3151:16:0/1:99
NC_023209	89945	ACT	ACCT	99	INDEL;DP=602;AF1=1;CI95=1,1;DP4=8,8,236,181;MQ=50;PV4=0.62,1,1,0.038	PL:DP:SP:GT:GQ	255,255,0:433:2:1/1:99
NC_023209	89947	TAAAAAAAAAA	TAAAAAAAAAAA,TAAAAAAAAAAAA	73.5	INDEL;DP=599;AF1=1;CI95=1,1;DP4=8,9,243,198;MQ=50;PV4=0.62,1,1,0.051	PL:DP:SP:GT:GQ	114,255,119,0,255,117:458:2:1/1:99
NC_023209	105666	TAAAAAAAAA	TAAAAAAAAAA,TAAAAAAAAAAA	99	INDEL;DP=453;AF1=1;CI95=1,1;DP4=5,9,146,217;MQ=50;PV4=0.79,1,0.37,1	PL:DP:SP:GT:GQ	147,255,154,0,255,143:377:1:1/1:99
NC_023209	126942	G	GT	27.5	INDEL;DP=244;AF1=0.5;CI95=0.5,0.5;DP4=8,11,11,4;MQ=45;PV4=0.091,4e-05,1,1	PL:DP:SP:GT:GQ	65,0,190:34:10:0/1:68
NC_023209	126943	GAA	GAAA	32.5	INDEL;DP=248;AF1=0.5;CI95=0.5,0.5;DP4=3,15,13,3;MQ=46;PV4=0.00039,1.1e-05,1,1	PL:DP:SP:GT:GQ	70,0,151:34:34:0/1:73
NC_023209	130276	CAAAAAAAAAA	CAAAAAAAAAAA,C	67.5	INDEL;DP=681;AF1=1;CI95=1,1;DP4=9	PL:DP:SP:GT:GQ	

			AAAAAAAAAAAA		,5,287,186;MQ=50;PV4=1,1,1,0.4	108,255,103,0,255,104:487:0:1/1:99
NC_023209	135885	AAAG	AAAGTGAATAAAG AAG	7.34	INDEL;DP=515;AF1=1;CI95=1,1;DP4=0,0,213,256;MQ=50	PL:DP:SP:GT:GQ 47,255,0:469:0:1/1:99
NC_023209	140256	C	T	99	DP=2718;AF1=1;CI95=0.5,1;DP4=247,224,1129,981;MQ=44;PV4=0.68,1,5e-100,1	PL:DP:SP:GT:GQ 230,18,0:2581:2:1/1:90
NC_023209	140345	C	G	99	DP=1136;AF1=0.5;CI95=0.5,0.5;DP4=255,251,227,275;MQ=44;PV4=0.1,3e-203,2e-174,9e-169	PL:DP:SP:GT:GQ 207,0,255:1008:10:0/1:99
NC_023209	150197	T	G	41	DP=4520;AF1=0.5;CI95=0.5,0.5;DP4=324,286,285,56;MQ=47;PV4=5e-22,6.6e-143,1.9e-41,1	PL:DP:SP:GT:GQ 71,0,255:951:213:0/1:74
NC_023209	150198	T	A	99	DP=4527;AF1=0.5;CI95=0.5,0.5;DP4=290,212,321,127;MQ=47;PV4=9.9e-06,0,3e-54,1	PL:DP:SP:GT:GQ 146,0,255:950:50:0/1:99
NC_023209	150204	A	G	44	DP=5211;AF1=1;CI95=1,1;DP4=307,223,2294,2143;MQ=44;PV4=0.0076,0,2.2e-88,5e-09	PL:DP:SP:GT:GQ 77,207,0:4967:21:1/1:99
NC_023209	150214	C	A	99	DP=5675;AF1=0.5;CI95=0.5,0.5;DP4=1502,1789,1252,977;MQ=45;PV4=1.8e-14,3.6e-22,3.2e-280,2.5e-31	PL:DP:SP:GT:GQ 255,0,255:5520:137:0/1:99
NC_023209	150222	G	A	34	DP=5532;AF1=0.5;CI95=0.5,0.5;DP4=1786,2153,798,640;MQ=45;PV4=4.8e-11,0,0,1.2e-247	PL:DP:SP:GT:GQ 64,0,255:5377:103:0/1:67
NC_023209	211457	A	C	99	DP=1558;AF1=0.5;CI95=0.5,0.5;DP4=339,321,166,570;MQ=47;PV4=3.4e-29,0,	PL:DP:SP:GT:GQ 189,0,255:1396:255:0/1:99

					2.8e-59,2e-208	
NC_023209	245644	C	A	99	DP=2607;AF1=0.5;CI95=0.5,0.5;DP4=395,345,778,965;MQ=42;PV4=7.6e-05,0.0023,0,2e-51	PL:DP:SP:GT:GQ 255,0,255:2483:41:0/1:99
NC_023209	245649	G	A	52	DP=2929;AF1=0.5;CI95=0.5,0.5;DP4=386,327,983,1109;MQ=42;PV4=0.0011,0,0,4.5e-27	PL:DP:SP:GT:GQ 82,0,159:2805:29:0/1:85
NC_023209	245676	C	T	44	DP=4773;AF1=1;CI95=1,1;DP4=407,338,1741,2128;MQ=45;PV4=1.5e-06,0,1e-170,1	PL:DP:SP:GT:GQ 77,176,0:4614:58:1/1:99
NC_023209	246480	C	T	99	DP=1507;AF1=0.5;CI95=0.5,0.5;DP4=335,413,211,470;MQ=49;PV4=8.8e-08,0,2e-15,7.3e-112	PL:DP:SP:GT:GQ 137,0,255:1429:71:0/1:99
NC_023209	293748	A	T	99	DP=1355;AF1=0.5;CI95=0.5,0.5;DP4=292,409,255,331;MQ=46;PV4=0.53,2.2e-322,3.2e-80,4.4e-180	PL:DP:SP:GT:GQ 182,0,255:1287:3:0/1:99
NC_023209	293996	C	T	99	DP=4288;AF1=1;CI95=1,1;DP4=217,274,1658,1995;MQ=45;PV4=0.63,1,1.4e-76,1	PL:DP:SP:GT:GQ 255,193,0:4144:2:1/1:99
NC_023209	294044	C	T	6.2	DP=1925;AF1=0.4999;CI95=0.5,0.5;DP4=249,236,700,673;MQ=45;PV4=0.92,0,1.5e-65,0.00031	PL:DP:SP:GT:GQ 35,0,154:1858:0:0/1:38
NC_023209	329124	C	T	99	DP=1137;AF1=0.5;CI95=0.5,0.5;DP4=259,196,341,332;MQ=44;PV4=0.045,3.3e-250,3e-100,1	PL:DP:SP:GT:GQ 206,0,255:1128:13:0/1:99
NC_023209	329169	T	C	99	DP=1131;AF1=0.5;CI95=0.5,0.5;DP4=2	PL:DP:SP:GT:GQ

					13,252,333,329;MQ=43;PV4=0.15,2.5e-292,2.2e-77,1	203,0,255:1127:8:0/1:99
NC_023209	329184	T	C	99	DP=1115;AF1=0.5;CI95=0.5,0.5;DP4=201,256,335,319;MQ=43;PV4=0.02,0,1.4e-77,1.4e-49	PL:DP:SP:GT:GQ 147,0,255:1111:17:0/1:99
NC_023209	329712	A	G	99	DP=1058;AF1=0.5;CI95=0.5,0.5;DP4=351,190,335,160;MQ=45;PV4=0.36,1.8e-289,1e-119,1.2e-09	PL:DP:SP:GT:GQ 172,0,255:1036:4:0/1:99
NC_023209	329726	T	C	99	DP=1056;AF1=0.5;CI95=0.5,0.5;DP4=341,195,337,180;MQ=45;PV4=0.61,0,3.5e-120,1	PL:DP:SP:GT:GQ 132,0,255:1053:2:0/1:99
NC_023209	329744	T	C	99	DP=1064;AF1=0.5;CI95=0.5,0.5;DP4=326,216,339,182;MQ=45;PV4=0.1,0,1.3e-120,1	PL:DP:SP:GT:GQ 176,0,255:1063:10:0/1:99
NC_023209	329871	C	T	52	DP=2451;AF1=0.5;CI95=0.5,0.5;DP4=337,293,670,1143;MQ=43;PV4=5.5e-13,0,5.6e-114,1	PL:DP:SP:GT:GQ 82,0,241:2443:123:0/1:85
NC_023209	329905	C	T	99	DP=2265;AF1=0.5;CI95=0.5,0.5;DP4=319,381,572,959;MQ=44;PV4=0.00028,0.12,2.7e-132,0.018	PL:DP:SP:GT:GQ 255,0,182:2231:36:0/1:99
NC_023209	329918	T	G	99	DP=1821;AF1=0.5;CI95=0.5,0.5;DP4=344,427,300,616;MQ=44;PV4=6.2e-07,0,6.8e-192,1	PL:DP:SP:GT:GQ 145,0,255:1687:62:0/1:99
NC_023209	329920	C	A	92	DP=1695;AF1=0.5;CI95=0.5,0.5;DP4=343,430,298,602;MQ=44;PV4=2.7e-06,0,2.8e-197,1	PL:DP:SP:GT:GQ 122,0,255:1673:56:0/1:99

NC_023209	370518	T	C	17.1	DP=3383;AF1=0.5001;CI95=0.5,0.5;DP4=296,333,1052,1582;MQ=47;PV4=0.0012,0,1e-76,1	PL:DP:SP:GT:GQ 47,0,34:3263:29:0/1:37
NC_023209	370536	C	G	12.3	DP=4136;AF1=0.5;CI95=0.5,0.5;DP4=283,357,1349,2101;MQ=47;PV4=0.016,0,3.9e-54,1	PL:DP:SP:GT:GQ 42,0,48:4090:18:0/1:44
NC_023209	370754	GATAGGATC	GATAGGATCCTATAGGATC	99	INDEL;DP=2980;AF1=0.5;CI95=0.5,0.5;DP4=154,213,941,819;MQ=49;PV4=7.2e-05,1,4.6e-11,1	PL:DP:SP:GT:GQ 255,0,165:2127:41:0/1:99
NC_023209	370971	C	CGTTAT	99	INDEL;DP=3593;AF1=1;CI95=1,1;DP4=102,79,1433,799;MQ=49;PV4=0.037,6.9e-50,1,1	PL:DP:SP:GT:GQ 255,255,0:2413:14:1/1:99
NC_023209	371034	TCCCTTAA	T	99	INDEL;DP=2986;AF1=0.5;CI95=0.5,0.5;DP4=517,367,391,203;MQ=45;PV4=0.0046,4.3e-75,1.9e-20,1	PL:DP:SP:GT:GQ 255,0,255:1478:23:0/1:99
NC_023209	371035	C	T	98	DP=1241;AF1=0.5;CI95=0.5,0.5;DP4=245,225,426,194;MQ=47;PV4=3e-08,0,2.1e-37,7.3e-113	PL:DP:SP:GT:GQ 128,0,255:1090:75:0/1:99
NC_023209	371082	T	G	99	DP=1382;AF1=0.5;CI95=0.5,0.5;DP4=354,354,443,177;MQ=47;PV4=1.2e-15,0,1,3e-17	PL:DP:SP:GT:GQ 129,0,255:1328:149:0/1:99
NC_023209	371209	ACCTAAG	A	99	INDEL;DP=3584;AF1=0.5;CI95=0.5,0.5;DP4=288,423,101,433;MQ=48;PV4=2e-16,3.5e-123,2.3e-31,1	PL:DP:SP:GT:GQ 255,0,255:1245:157:0/1:99
NC_023209	371210	CCTAAGA	C	99	INDEL;DP=3577;AF1=0.5;CI95=0.5,0.5;DP4=198,253,166,660;MQ=48;PV4=8.1	PL:DP:SP:GT:GQ 255,0,255:1277:181:0/1:99

					e-19,4.6e-165,2.9e-17,1	
NC_023209	371211	C	T	67	DP=732;AF1=0.5;CI95=0.5,0.5;DP4=207,253,81,165;MQ=49;PV4=0.0022,1.8e-219,1e-29,1.8e-75	PL:DP:SP:GT:GQ 97,0,255:706:27:0/1:99
NC_023209	371258	G	T	99	DP=3439;AF1=1;CI95=1,1;DP4=180,280,486,2364;MQ=40;PV4=1.5e-24,0.038,1.7e-175,0.049	PL:DP:SP:GT:GQ 232,63,0:3310:238:1/1:99
NC_023209	371281	C	T	99	DP=3304;AF1=1;CI95=1,1;DP4=199,271,587,2094;MQ=37;PV4=2.6e-19,0.47,0,0.021	PL:DP:SP:GT:GQ 208,53,0:3151:186:1/1:99
NC_023209	371346	C	T	99	DP=3181;AF1=1;CI95=1,1;DP4=250,252,673,1915;MQ=40;PV4=8.1e-25,1,3.6e-164,1	PL:DP:SP:GT:GQ 228,33,0:3090:241:1/1:99
NC_023209	371385	GACTTTTTACTT	GACTT	44.5	INDEL;DP=2094;AF1=0.5;CI95=0.5,0.5;DP4=253,234,48,172;MQ=45;PV4=2.1e-14,2.1e-247,1.2e-146,1	PL:DP:SP:GT:GQ 82,0,255:707:137:0/1:85
NC_023209	371503	C	A	5.46	DP=2963;AF1=0.4999;CI95=0.5,0.5;DP4=302,283,130,210;MQ=48;PV4=9.6e-05,4.8e-290,1.1e-67,1	PL:DP:SP:GT:GQ 34,0,255:925:40:0/1:37
NC_023209	371574	T	A	10.4	DP=2290;AF1=0.5;CI95=0.5,0.5;DP4=333,265,633,1053;MQ=47;PV4=2e-14,0,7.6e-40,4.4e-15	PL:DP:SP:GT:GQ 40,0,185:2284:137:0/1:43
NC_023209	372935	G	A	5.45	DP=6066;AF1=1;CI95=1,1;DP4=449,546,2392,2510;MQ=48;PV4=0.037,0,7.4e-80,1	PL:DP:SP:GT:GQ 37,45,0:5897:14:1/1:99
NC_023209	378823	GAGGTTCTA	G	18.5	INDEL;DP=5063;AF1=0.5;CI95=0.5,0.5;	PL:DP:SP:GT:GQ

					DP4=2976,1349,388,224;MQ=50;PV4=0.0082,2.5e-174,1,1	56,0,255:4937:21:0/1:59
NC_023209	384671	A	G	99	DP=531;AF1=1;CI95=1,1;DP4=3,2,242,251;MQ=50;PV4=0.68,1,0.18,1	PL:DP:SP:GT:GQ 255,255,0:498:2:1/1:99
NC_023209	406249	T	C	70	DP=3123;AF1=0.5;CI95=0.5,0.5;DP4=367,516,746,1346;MQ=44;PV4=0.0025,0,3.9e-294,1	PL:DP:SP:GT:GQ 100,0,252:2975:26:0/1:99
NC_023209	406280	A	C	99	DP=3704;AF1=0.5;CI95=0.5,0.5;DP4=457,472,975,1743;MQ=43;PV4=1.2e-12,0.0098,0,1	PL:DP:SP:GT:GQ 255,0,227:3647:119:0/1:99
NC_023209	406286	C	T	55	DP=3684;AF1=0.5;CI95=0.5,0.5;DP4=473,452,965,1728;MQ=43;PV4=4.4e-16,0,0,1	PL:DP:SP:GT:GQ 85,0,210:3618:154:0/1:88
NC_023209	406319	A	C	33	DP=2587;AF1=0.5;CI95=0.5,0.5;DP4=543,407,492,959;MQ=42;PV4=3e-29,0,0,1	PL:DP:SP:GT:GQ 63,0,255:2401:255:0/1:66
NC_023209	406324	C	T	99	DP=2534;AF1=0.5;CI95=0.5,0.5;DP4=572,412,492,956;MQ=42;PV4=5.6e-32,0,0,0.45	PL:DP:SP:GT:GQ 193,0,255:2432:255:0/1:99
NC_023209	406336	C	T	99	DP=2107;AF1=0.5;CI95=0.5,0.5;DP4=602,386,350,695;MQ=43;PV4=1.6e-35,0,0,2.2e-49	PL:DP:SP:GT:GQ 177,0,255:2033:255:0/1:99
NC_023209	406348	G	A	46	DP=1559;AF1=0.5;CI95=0.5,0.5;DP4=587,365,151,320;MQ=45;PV4=3.8e-26,8.2e-262,0,1.4e-158	PL:DP:SP:GT:GQ 76,0,255:1423:254:0/1:79
NC_023209	422730	ACTT	ACTTCTT	99	INDEL;DP=561;AF1=1;CI95=1,1;DP4=5	PL:DP:SP:GT:GQ 255,103,0:93:1:1/1:99

					,5,34,49;MQ=49;PV4=0.74,1,0.21,2.1e-08	
NC_023209	432717	TAAAAAAAAAA	TAAAAAAAAAAA,TAAAAAAAAAAAA	71.5	INDEL;DP=362;AF1=1;CI95=1,1;DP4=6,4,152,105;MQ=50;PV4=1,1,0.27,1	PL:DP:SP:GT:GQ 112,255,121,0,255,108:267:0:1/1:99
NC_023209	432720	A	AN	74.5	INDEL;DP=375;AF1=1;CI95=1,1;DP4=6,4,148,104;MQ=50;PV4=1,1,0.26,1	PL:DP:SP:GT:GQ 115,255,0:262:0:1/1:99
NC_023209	434680	A	G	11.3	DP=731;AF1=0.5;CI95=0.5,0.5;DP4=274,216,76,164;MQ=44;PV4=6.3e-10,6.5e-138,5.4e-232,1	PL:DP:SP:GT:GQ 41,0,255:730:92:0/1:44
NC_023209	434683	A	C	67	DP=821;AF1=0.5;CI95=0.5,0.5;DP4=274,218,74,192;MQ=43;PV4=1.5e-13,2.5e-70,5.7e-234,1	PL:DP:SP:GT:GQ 97,0,255:758:128:0/1:99
NC_023209	434709	T	G	66	DP=1292;AF1=0.5;CI95=0.5,0.5;DP4=257,193,378,324;MQ=38;PV4=0.3,0,0,1	PL:DP:SP:GT:GQ 96,0,255:1152:5:0/1:99
NC_023209	434722	T	C	99	DP=1278;AF1=0.5;CI95=0.5,0.5;DP4=275,182,401,391;MQ=37;PV4=0.0012,4.4e-22,0,1	PL:DP:SP:GT:GQ 217,0,255:1249:29:0/1:99
NC_023209	434727	T	C	99	DP=1254;AF1=0.5;CI95=0.5,0.5;DP4=261,171,416,405;MQ=37;PV4=0.001,0.0005,0,1	PL:DP:SP:GT:GQ 247,0,255:1253:30:0/1:99
NC_023209	434731	T	C	99	DP=1241;AF1=0.5;CI95=0.5,0.5;DP4=261,160,413,402;MQ=37;PV4=0.00018,3.1e-321,0,1	PL:DP:SP:GT:GQ 146,0,255:1236:37:0/1:99
NC_023209	434753	A	G	95	DP=1918;AF1=0.5;CI95=0.5,0.5;DP4=265,178,856,532;MQ=37;PV4=0.5,0,5.8e-305,1.9e-07	PL:DP:SP:GT:GQ 125,0,206:1831:3:0/1:99

NC_023209	434789	A	T	23	DP=2385;AF1=0.5;CI95=0.5,0.5;DP4=268,196,1091,722;MQ=42;PV4=0.37,0,1.3e-194,1	PL:DP:SP:GT:GQ 53,0,79:2277:4:0/1:56
NC_023209	434792	A	G	99	DP=2236;AF1=0.5;CI95=0.5,0.5;DP4=266,197,1056,706;MQ=42;PV4=0.34,4.5e-07,1.2e-213,1	PL:DP:SP:GT:GQ 240,0,90:2225:5:0/1:93
NC_023209	434810	G	T	89	DP=2320;AF1=0.5;CI95=0.5,0.5;DP4=234,207,410,358;MQ=44;PV4=0.95,0,4.8e-129,1	PL:DP:SP:GT:GQ 119,0,255:1209:0:0/1:99
NC_023209	436398	TTTCTAAATT	TTTCTAAATTCTAAATT	99	INDEL;DP=4812;AF1=0.5;CI95=0.5,0.5;DP4=288,279,1829,1724;MQ=50;PV4=0.79,1,0.21,1	PL:DP:SP:GT:GQ 255,0,174:4120:1:0/1:99
NC_023209	436401	CTAAATT	CTAAATTATAAATT	99	INDEL;DP=5168;AF1=0.5;CI95=0.5,0.5;DP4=552,486,1727,1619;MQ=49;PV4=0.39,1,1,1	PL:DP:SP:GT:GQ 255,0,255:4384:4:0/1:99
NC_023209	444313	T	C	99	DP=800;AF1=1;CI95=1,1;DP4=1,1,428,330;MQ=42;PV4=1,4.9e-18,1,0.16	PL:DP:SP:GT:GQ 255,255,0:760:0:1/1:99
NC_023209	472132	CAAAAAAAAAA	CAAAAAAAAAAAA,CAAAAAAAAAAAAA	99	INDEL;DP=596;AF1=1;CI95=1,1;DP4=7,4,225,148;MQ=50;PV4=1,1,1,0.3	PL:DP:SP:GT:GQ 232,255,173,0,255,169:384:0:1/1:99
NC_023209	472141	AA	AACA	14.6	INDEL;DP=653;AF1=0.5;CI95=0.5,0.5;DP4=73,54,67,28;MQ=47;PV4=0.05,2.2e-54,1,1	PL:DP:SP:GT:GQ 52,0,143:222:13:0/1:55
NC_023209	472142	A	AAC	99	INDEL;DP=660;AF1=0.5;CI95=0.5,0.5;DP4=81,54,93,24;MQ=48;PV4=0.001,2.8e-25,1,1	PL:DP:SP:GT:GQ 155,0,255:252:30:0/1:99
NC_023209	484277	CTTTTTTTTT	CTTTTTTTTTT,CTT	99	INDEL;DP=623;AF1=1;CI95=1,1;DP4=3	PL:DP:SP:GT:GQ

			TTTTTTTTT		,4,193,271;MQ=50;PV4=1,1,0.38,0.4	150,255,153,0,255,149:471:0:1/1:99
NC_023209	491516	ACC	AC	99	INDEL;DP=559;AF1=1;CI95=1,1;DP4=0,4,31,50;MQ=50;PV4=0.29,1,1,0.018	PL:DP:SP:GT:GQ 255,174,0:85:5:1/1:99
NC_023209	491517	C	CT	99	INDEL;DP=546;AF1=0.5;CI95=0.5,0.5;DP4=2,12,6,11;MQ=50;PV4=0.24,1,1,0.18	PL:DP:SP:GT:GQ 161,0,69:31:6:0/1:72
NC_023209	491518	C	T	99	DP=557;AF1=1;CI95=1,1;DP4=0,2,27,126;MQ=50;PV4=1,1,1,1	PL:DP:SP:GT:GQ 192,255,0:155:0:1/1:99

Table S3 Tissue distribution of RNA editing sites on the mitogenome of *S. miltiorrhiza*.

Table S4 Tissue expression patterns of RNA editing sites (A) Only two categories are considered. “2” : RNA editing sites predicted; “0”: RNA editing sites were not predicted in this tissue because it did not pass the threshold or there were no reads covering the regions; (B) Three categories are considered. “2” : RNA editing sites predicted; “1”: RNA editing sites were not predicted because it did not passing the threshold but there was at least one read covering the regions ; “0” RNA editing sites were not predicted because there were no read covering the regions.

(A)

Expression Patterns			Numbers	Strand
Flower	Leaf	Root		
0	0	2	87	Positive
0	2	0	87	Positive
0	2	2	19	Positive
2	0	0	126	Positive
2	0	2	36	Positive
2	2	0	56	Positive
2	2	2	89	Positive
0	0	2	86	Negative
0	2	0	92	Negative
0	2	2	21	Negative
2	0	0	124	Negative
2	0	2	29	Negative

2	2	0	50	Negative
2	2	2	97	Negative

(B)

**Expression Patterns**				
**Flower**	**Leaf**	**Root**	**Numbers**	**Strand**
0	0	1	18	Positive
0	0	2	20	Positive
0	1	0	60	Positive
0	1	1	31	Positive
0	1	2	15	Positive
0	2	0	16	Positive
0	2	1	11	Positive
1	0	0	15	Positive
1	0	1	10	Positive
1	0	2	6	Positive
1	1	0	44	Positive
1	1	1	108	Positive
1	1	2	46	Positive
1	2	0	17	Positive
1	2	1	43	Positive
1	2	2	19	Positive
2	0	0	24	Positive
2	0	1	14	Positive
2	0	2	1	Positive
2	1	0	27	Positive
2	1	1	61	Positive
2	1	2	35	Positive
2	2	0	13	Positive
2	2	1	43	Positive
2	2	2	89	Positive
0	0	0	85	Negative
0	0	1	20	Negative
0	0	2	23	Negative
0	1	0	61	Negative
0	1	1	22	Negative
0	1	2	7	Negative
0	2	0	24	Negative
0	2	1	10	Negative
0	2	2	6	Negative

1	0	0	14	Negative
1	0	1	10	Negative
1	0	2	5	Negative
1	1	0	50	Negative
1	1	1	110	Negative
1	1	2	51	Negative
1	2	0	25	Negative
1	2	1	33	Negative
1	2	2	15	Negative
2	0	0	24	Negative
2	0	1	15	Negative
2	0	2	1	Negative
2	1	0	27	Negative
2	1	1	58	Negative
2	1	2	28	Negative
2	2	0	16	Negative
2	2	1	34	Negative
2	2	2	97	Negative

Table S5 List of 225 “C to U” RNA editing sites in the CDS regions on the mitogenome of *S. miltiorrhiza*.

Classification of Genes	Editing site[a]	Genomic position	Codon[b]	Amino acid change	Editing frequency
Complex I	nad1-215	179918	TCC	S72F[d]	1
	nad1-265	179868	CGG	R89W[d]	1
	nad1-307	179826	CCG	P103S	0.73
	nad1-308	179825	CCG	P103L	0.82
	nad1-376	179757	CGG	R126W[d]	1
	nad1-401	452265	TCT	S134F[d]	1
	nad1-889	266043	CGG	R297W[d]	1
	nad1-919	266013	CGG	R307W[d]	1
	nad1-814	266118	CTC	L272L[c]	0.91
	nad1-731	266201	TCT	S244F[d]	1
	nad2-967	70439	CTA	L323L[c]	0.7
	nad2-455	273421	TCG	S152L[d]	1
	nad2-359	273517	TCG	S152L[d]	1
	nad2-325	273551	CGC	R109C	1
	nad2-319	273557	CCT	P107S	1
	nad2-314	273562	CCA	P105L	1
	nad3-344	395500	TCG	S115L[d]	1
	nad3-349	395495	CGG	R117W[d]	1
	nad3-317	395527	TCT	S106F[d]	0.99
	nad3-275	395569	TCT	S 92F[d]	0.98
	nad3-266	395578	CCC	P89L	0.97
	nad3-251	395593	CCC	P84L	0.96
	nad3-247	395597	CCT	P83S	0.92
	nad3-231	395613	TCC	S77S[c]	0.5
	nad3-230	395614	TCC	S77L[d]	0.96
	nad3-215	395629	CCG	P72L	0.96
	nad3-209	395635	CCT	P70L	1
	nad3-208	395636	CCT	P70S	1
	nad3-146	395698	TCC	S49L[d]	0.97
	nad3-124	395720	CAC	H42Y[d]	0.95
	nad3-80	395764	CCA	P27L	1
	nad3-79	395765	CCA	P27S	1
	nad3-62	395782	CCA	P21L	1
	nad3-44	395800	TCG	S15L[d]	1
	nad4-1424	318223	CCG	P475L	0.48
	nad4-1408	318239	CAC	H470Y[d]	0.48
	nad4-1364	321120	TCC	S455L[d]	1

nad4-1346	321138	CCA	P447L	1
nad4-1298	321186	GCG	A433V	1
nad4-1163	321321	TCA	S388L[d]	1
nad4-1142	321342	TCC	S381L[d]	1
nad4-1007	321477	TCA	S336L[d]	1
nad4-1001	321483	CCG	P334L	1
nad4-878	324209	TCG	S293L[d]	0.86
nad4-848	324239	CCA	P283L	0.72
nad4-847	324240	CCA	P283S	0.72
nad4-758	324329	CCT	P253L	0.9
nad4-440	326042	CCA	P147L	0.86
nad4-428	326054	CCC	P143L	0.78
nad4-427	326055	CCC	P143S	0.92
nad4-424	326058	CTT	L142F	0.71
nad4-188	326294	TCT	S63F[d]	0.9
nad4-176	326306	TCT	S59F[d]	0.17
nad4-157	326325	CCG	R53W[d]	0.85
nad4-149	326333	CCT	P50L	0.85
nad4L-158	291966	TCG	S53L[d]	1
nad4L-179	291987	TCA	S60L[d]	1
nad4L-188	291996	TCA	S63L[d]	1
nad4L-197	292005	TCA	S66L[d]	1
nad4L-281	292089	TCT	S94F[d]	0.94
nad5-359	35588	TCT	S120F[d]	0.88
nad5-374	35603	CCA	P125L	1
nad5-398	35627	TCT	S133F[d]	1
nad5-539	35768	CCT	P180L	1
nad5-548	35777	TCG	S183L[d]	1
nad5-608	35837	GCC	A203V	1
nad5-676	35905	CTT	L226F	1
nad5-713	35942	TCG	S238L[d]	1
nad5-725	35954	TCA	S242L[d]	1
nad5-835	36064	CCG	P279S	1
nad5-1310	36539	TCA	S437L[d]	0.64
nad5-1466	204858	CCC	P489L	1
nad5-1526	204918	ACC	T509I[d]	1
nad5-1544	204936	CCG	P515L	1
nad5-1586	204978	TCC	S529L[d]	1
nad5-1871	206303	TCA	S624L[d]	1
nad5-1892	206324	TCT	S631F[d]	1
nad5-1894	206326	CGT	R632C	1
nad5-1934	206366	TCG	S644L[d]	1
nad6-26	49648	CCT	P9L	0.92
nad6-88	49710	CCC	P30S	0.98

	nad6-89	49711	CCC	P30L	0.98
	nad6-90	49712	CCC	P30P[c]	0.27
	nad6-95	49717	CCA	P32L	0.98
	nad6-103	49725	CGC	R35C	0.98
	nad6-161	49783	CCA	P54L	0.9
	nad6-169	49791	CAT	H57Y[d]	0.88
	nad6-191	49813	TCA	S64L[d]	0.88
	nad6-463	50085	CCT	P155S	0.99
	nad7-77	427622	TCA	S26L[d]	1
	nad7-137	427682	TCA	S46L[d]	1
	nad7-200	428608	TCT	S67F[d]	1
	nad7-531	430264	TCC	S177S[c]	0.52
	nad7-724	430457	CAT	H242Y[d]	1
	nad7-739	430472	CCT	P247S	1
	nad7-740	430473	CCT	P247L	1
	nad7-1050	432125	CCC	P350P[c]	0.92
	nad7-1057	432132	CGT	R353C	0.71
	nad7-1103	432178	TCT	S368F[d]	0.88
	nad9-478	43785	CCA	P160S	0.33
	nad9-398	43865	TCA	S133L[d]	0.83
	nad9-368	43895	TCC	S123L[d]	1
	nad9-113	44150	CCA	P38L	1
	nad9-92	44171	TCT	S31F[d]	1
Complex III	Cob-1160	19318	ACG	T387M	1
	Cob-1084	19394	CCT	P362S	1
	Cob-1015	19463	CGC	R339C	1
	Cob-982	19496	CAC	H328Y[d]	0.89
	Cob-358	20120	CGG	R120W[d]	1
	Cob-325	20153	CAT	H109Y[d]	1
	Cob-298	20180	CAC	H100Y[d]	1
ComplexIV	cox1-242	343698	TCT	S81F[d]	1
	cox1-254	343710	TCT	S85F[d]	0.96
	cox1-265	343721	CTG	L89L[c]	0.11
	cox1-452	343908	TCT	S151F[d]	1
	cox1-515	343971	TCC	S172F[d]	1
	cox1-551	344007	TCA	S184L[d]	1
	cox1-590	344046	CCA	P197L	1
	cox1-1405	345822	CGT	R469C	1
	cox1-1433	345850	TCA	S478L[d]	0.9
	cox1-1489	345906	CCA	P497S	0.89
	cox1-1499	345916	CCA	P500L	0.88
	cox2-745	27294	CGG	R249W[d]	1
	cox2-724	27315	CCT	P242S	1
	cox2-632	30484	TCG	S211L[d]	1

	cox2-557	30559	CCT	P186L	0.83
	cox2-544	30572	CCT	P182S	1
	cox2-476	30640	TCA	S159L[d]	1
	cox2-71	31045	TCT	S24F[d]	1
	cox3-764	484425	CCA	P255 L	0.93
	cox3-754	484435	CGG	R252W[d]	1
	cox3-422	484767	CCT	P141L	1
	cox3-419	484770	CCC	P140L	1
	cox3-314	484875	TCT	S105F[d]	1
	cox3-311	484878	TCT	S104F[d]	0.99
	cox3-304	484885	CGG	R102W[d]	0.97
	cox3-245	484944	CCT	P82L	0.8
	cox3-174	485015	TTC	F58F[c]	0.14
Complex V	atp4-59	292338	TCT	S20F[d]	1
	atp4-71	292350	TCA	S24L[d]	1
	atp4-89	292368	TCA	S30L[d]	1
	atp4-395	292674	TCA	S132L[d]	1
	atp4-407	292686	CCA	P136L	1
	atp4-416	292695	ACT	T139I[d]	0.97
	atp8-58	486195	CTC	L20L[c]	1
	atp8-47	486206	TCA	S16L[d]	1
	atp9-212	421266	TCA	S71L[d]	1
Ribosomal proteins S	rps10-2	128591	ACG	T1M	1
	rps12-284	395155	TCC	S95L[d]	1
	rps12-221	395218	TCG	S74L[d]	1
	rps12-196	395243	CAC	H65Y[d]	1
	rps12-159	395280	GCC	A53A[c]	0.9
	rps12-104	395335	CCG	P35L	1
	rps13-56	267352	TCA	S19L[d]	1
	rps13-100	267308	CGT	R34C	1
	rps3-701	169698	TCG	S234L[d]	0.92
	rps3-1472	168927	TCA	S491L[d]	0.81
	rps3-1525	168874	CGT	R509C	0.81
	rps3-1558	168841	CCT	P520S	0.99
	rps7-175	156024	CTA	L59L[c]	0.14
Ribosomal proteins L	rpl16-146	168662	ACT	T49I[d]	0.99
	rpl16-449	168359	TCG	S150L[d]	1
	rpl23-71	144814	TCT	S24F[d]	0.76
	rpl23-89	144796	TCA	S30L[d]	0.86
	rpl5-35	411855	TCA	S12L[d]	1
	rpl5-47	411867	CCG	P16L	1
	rpl5-414	412234	TCC	S138S[c]	0.62

	rpl5-435	412255	TTC	F145F[c]	0.7
	rpl5-441	412261	ATC	I147I[c]	1
Cytochrome biosynthesis	ccmFc-310	288029	CGT	R104C	1
	ccmFn-1519	472504	CCC	P507S	1
	ccmFn-1484	472539	TCA	S495L[d]	0.95
	ccmFn-1472	472551	CCA	P491L	0.96
	ccmFn-1448	472575	TCC	S483F[d]	0.93
	ccmFn-1387	472636	CGG	R463W[d]	1
	ccmFn-1354	472669	CGG	R452W[d]	1
	ccmFn-1321	472702	CTA	H440Y[d]	1
	ccmFn-1304	472719	CCA	P435L	1
	ccmFn-809	473214	TCA	S270L[d]	1
	ccmFn-794	473229	CCA	P265L	1
	ccmFn-782	473241	TCA	S261L[d]	1
	ccmFn-760	473263	CGT	R254C	0.95
	ccmFn-722	473301	TCA	S241L[d]	1
	ccmFn- 713	473310	CCT	P238L	1
	ccmC-400	138815	CTT	L134F	0.9
	ccmC-399	138816	TTC	L133F	0.91
	ccmC-331	138884	CGG	R111W[d]	0.86
	ccmC-184	139031	CGG	R62W[d]	1
	ccmC-179	139036	GCG	A60V	1
	ccmC-161	139054	CCC	P54L	1
Other	matR-1744	6086	CTC	H582Y[d]	1
	matR-1708	6122	CGC	R570C	1
	matR-1688	6142	CCT	P563L	1
	matR-1667	6163	TCC	S556L[d]	1
	matR-43	7787	CCC	P15S	1
	matR-32	7798	TCC	S11F[d]	1
	mttB-695	9269	TCT	S232 F[d]	0.56
	mttB-687	9277	TTC	L229 F	0.71
	mttB-682	9282	CGT	R228C	0.96
	mttB-631	9333	CTC	L211L[c]	0.81
	mttB-625	9339	CCG	P209S	0.97
	mttB-593	9371	TCC	S198L[d]	0.88
	mttB-569	9395	CCA	P190L	1
	mttB-394	9570	CGG	R132W[d]	0.75
	mttB-391	9573	CCC	P131S	0.83
	mttB-388	9576	CTT	L130F	0.75
	mttB-361	9603	CGC	R121C	0.75
	mttB-346	9618	CAT	H116Y[d]	0.9
	mttB-343	9621	CTC	L115L[c]	0.75
	orf108b-214	434175	CTT	L72F	1

orf108b-242	434203	GCA	A81V	1
orf111c-55	318644	CAT	H19Y[d]	1
orf214-305	420919	CCC	P102L	0.71
orf281-86	342411	TCG	S29L[d]	1
orf281-111	342436	TTC	L37F	1
orf304-756	345019_	CTC	L252L[c]	1
orf304- 774	345037	GAC	D258D[c]	1
orf456-1236	468310	CGC	R412R[c]	0.45
orf456-1185	468361	TCC	S395S[c]	1
orf456-1090	468456	CTC	L364L[c]	1
orf456-1072	468474	CAT	H358Y[d]	1
orf456-897	468649	AGC	S299S[c]	1
orf456-684	468862	GCC	A228A[c]	1
orf456-474	469072	TGC	C158C[c]	0.97
orf456-375	469171	AAC	N125N[c]	1

[a]The name of each editing site is the combination of the gene name and its position number on the CDS joined with"-", with the translation initiation site being position 1. [b]The edited base "C" in each codon is underlined. [c]RNA editing events that do not change the encoded amino acids. [d]Editing events increasing the hydrophobicity of the encoded amino acids.

.

Table S6 NUMTs identified in *S. miltiorrhiza*.

Query id	% identity	Alignment length	Mismatches	Gap openings	Query start	Query end	Subject start	Subject end	e-value	bit score
tig1072	94.01	167	2	2	230	388	145037	145203	5E-65	246
tig10945	91.7	253	14	4	7	255	402540	402291	5E-95	344
tig11023	89.67	271	15	9	12461	12728	89160	88900	3E-91	333
tig1107	96.86	159	5	0	4731	4889	151666	151508	2E-71	267
tig11264	97.29	221	0	6	4	221	89912	89695	6E-103	370
tig11569	94.19	155	9	0	7269	7423	144963	145117	1E-62	237
tig11940	96.59	205	6	1	8536	8739	442998	442794	4E-93	339
tig11941	90	220	11	4	5197	5414	442772	442562	1E-73	274
tig12051	96.18	157	1	1	1835	1986	139300	139144	4E-67	252
tig12118	93.27	208	7	7	1	201	54497	54704	1E-81	300
tig12162	88.96	317	19	6	3939	4241	283623	283937	4E-105	377
tig12848	90.07	282	18	8	3770	4043	498731	498452	1E-98	357
tig12952	95.9	122	5	0	2464	2585	148157	148036	1E-50	198
tig13193	77.78	540	63	22	16413	16903	143238	143769	5E-75	279
tig1320	92.98	285	18	2	34	317	412780	412497	9E-116	414
tig13226	97.89	190	4	0	14004	14193	451031	450842	4E-90	329
tig13560	97.54	285	7	0	12817	13101	164802	165086	1E-137	488
tig13671	99.26	135	0	1	995	1128	57017	57151	2E-64	243
tig14030	97.63	211	4	1	1226	1435	201869	202079	2E-100	361
tig14197	91.78	146	12	0	1094	1239	173353	173208	2E-52	204

tig14256	95.86	169	6	1	552	720	173825	173658	7E-73	272
tig14356	99.52	209	0	1	961	1169	82009	81802	9E-106	379
tig14357	96.92	325	9	1	1167	1490	80878	80554	1E-155	544
tig14361	98.48	263	2	2	2103	2363	68742	68480	9E-131	462
tig14364	98.77	244	2	1	188	430	65341	65098	2E-122	433
tig14410	99.03	103	1	0	1269	1371	145564	145666	4E-47	185
tig1453	89.77	264	22	1	16683	16946	372526	372268	4E-91	333
tig1455	92.57	296	12	3	46	331	375836	375541	4E-117	416
tig14572	95.61	592	26	0	2	593	126178	125587	0	950
tig14644	85.1	255	18	11	13792	14026	42765	42511	1E-63	243
tig14681	99.29	283	2	0	43	325	337022	337304	3E-146	512
tig14753	99.41	169	0	1	1	168	450368	450200	1E-83	305
tig14769	98.37	369	6	0	41005	41373	330019	330387	0	649
tig14772	98.77	162	0	2	4142	4301	1673	1834	7E-78	287
tig14864	95.48	155	6	1	1162	1315	188778	188932	1E-65	246
tig14996	92.62	149	10	1	1267	1414	277247	277395	2E-55	213
tig15104	85.26	190	28	0	8420	8609	420766	420577	5E-50	196
tig15171	92.66	109	5	2	366	474	170952	171057	4E-38	154
tig15197	91	100	4	1	7293	7387	312313	312214	4E-30	130
tig15213	99.11	224	0	2	1	224	54681	54460	8E-113	401
tig15219	86.42	162	12	3	11970	12124	169765	169923	1E-41	169
tig15682	98.21	168	1	2	12	178	8770	8936	3E-79	292
tig15688	82.34	1314	185	30	4699	6004	237850	239124	0	1098
tig15793	99.22	129	1	0	1019	1147	177900	177772	3E-62	233
tig16090	95.48	177	2	2	5561	5731	197829	197653	8E-75	278
tig16165	98.65	148	0	2	10140	10287	476751	476896	1E-69	261

tig16214	98.73	158	2	0	588	745	328538	328381	3E-75	281
tig16215	87.67	219	20	6	1009	1225	263688	263475	5E-66	248
tig16231	91.01	356	24	3	12326	12679	149523	149174	1E-133	473
tig16232	98.9	181	1	1	79	258	148454	148274	1E-87	322
tig16335	90.44	136	11	2	2358	2491	157579	157444	3E-45	178
tig16579	96.12	232	8	1	1164	1394	293743	293974	2E-105	377
tig16650	96.74	184	2	1	17350	17533	323306	323127	5E-82	303
tig17018	98.51	268	2	1	336	603	80357	80622	4E-133	472
tig1711	92.38	105	7	1	3820	3923	102102	101998	2E-35	148
tig17151	92.55	161	12	0	7868	8028	153970	153810	6E-61	231
tig17215	93.49	169	11	0	36572	36740	317948	317780	3E-66	252
tig1725	93.27	104	7	0	31837	31940	275512	275615	6E-37	154
tig17383	88.68	106	7	4	9849	9954	147217	147317	1E-28	124
tig17384	93.33	390	17	2	1	390	147335	147715	6E-162	568
tig17583	87.21	383	26	7	35853	36235	188294	188653	3E-115	414
tig17725	99.03	103	1	0	409	511	489051	488949	6E-48	185
tig17728	93.7	238	14	1	1512	1749	91030	90794	1E-98	355
tig17737	94.67	169	1	2	20321	20489	464795	464635	9E-68	255
tig17776	98.69	153	2	0	6812	6964	185176	185024	3E-73	272
tig18148	98.9	272	0	3	1755	2024	399084	399354	4E-137	483
tig18190	96.72	122	4	0	17289	17410	3202	3081	3E-52	204
tig18347	94.42	233	12	1	10589	10821	465768	465999	1E-98	357
tig18353	86.55	1041	97	18	5146	6181	158857	157855	0	1107
tig18354	87.36	831	81	6	4	817	157426	156603	0	931
tig1842	93.01	143	10	0	418	560	459054	458912	7E-55	209
tig18728	97.6	125	3	0	1127	1251	318525	318649	7E-56	215

tig18984	84.4	109	10	6	1	102	292326	292218	8E-21	100
tig1899	98.52	270	3	1	2211	2479	152813	153082	1E-134	475
tig19045	92.88	393	22	4	4281	4668	155399	155790	1E-161	566
tig19065	92.55	188	12	2	15178	15363	148566	148379	9E-72	268
tig19125	91	100	2	1	449	548	315615	315707	3E-29	128
tig19335	94.31	123	7	0	9460	9582	136657	136779	8E-48	189
tig19837	92.74	317	20	3	978	1291	274848	275164	9E-129	455
tig20229	94.31	123	5	2	13783	13903	443941	444063	3E-47	187
tig20230	95.88	194	7	1	702	894	167927	167734	7E-86	313
tig20244	93.2	309	14	2	2440	2748	95458	95157	3E-126	448
tig20575	94.89	137	6	1	18	154	93982	94117	1E-55	213
tig20664	96.33	109	4	0	8007	8115	158557	158449	2E-45	180
tig2073	95.28	106	3	2	986	1089	320512	320407	3E-41	167
tig20741	97.31	223	0	6	1	223	61382	61598	5E-103	374
tig20797	90.09	111	5	3	3827	3936	213897	213792	2E-33	139
tig20816	88	225	15	3	8009	8231	433976	434190	6E-68	255
tig20846	96.58	146	5	0	7586	7731	64259	64114	3E-64	243
tig20848	97.44	195	3	1	475	667	61057	60863	1E-91	331
tig21253	94.95	475	11	2	2923	3388	147740	147270	0	732
tig21254	97.75	311	7	0	386	696	146274	145964	6E-153	536
tig21260	88.82	161	18	0	16588	16748	400376	400216	2E-50	198
tig21273	93.27	431	14	3	855	1270	228255	228685	6E-179	621
tig21416	96.5	257	9	0	72	328	151625	151369	7E-120	425
tig21443	96.4	111	4	0	15259	15369	95333	95223	4E-46	183
tig21444	98.05	205	4	0	3132	3336	461570	461774	5E-99	357
tig21445	85.52	905	108	10	349	1243	75823	74932	0	924

tig21534	91.23	114	10	0	141	254	148803	148690	6E-38	156
tig21562	94.85	136	5	2	11902	12036	201285	201151	2E-54	211
tig21707	94.27	157	9	0	23717	23873	321381	321225	5E-63	241
tig21724	99.37	317	0	2	1	315	285623	285307	2E-163	573
tig21913	94.52	146	5	1	1092	1237	277537	277395	4E-58	222
tig2211	95.22	335	11	3	537	866	37927	37593	8E-150	525
tig2220	93.52	494	29	2	3744	4235	125927	126419	0	732
tig22222	89.17	240	18	4	8007	8244	370991	370758	5E-79	292
tig2229	93.79	177	11	0	658	834	330473	330297	3E-72	267
tig22315	88.26	298	33	2	1391	1687	102703	102407	9E-98	355
tig22456	99.33	298	0	2	1	298	169384	169679	4E-153	538
tig22470	99.01	302	0	3	860	1159	60095	59795	4E-154	538
tig22472	98.68	227	3	0	1672	1898	57522	57296	3E-113	403
tig22479	86.54	104	13	1	1322	1424	41634	41531	2E-25	113
tig22597	92.81	153	11	0	1262	1414	310	462	1E-58	222
tig22678	97.85	186	4	0	3416	3601	107910	107725	6E-88	322
tig22678	97.85	186	4	0	3416	3601	308765	308580	6E-88	322
tig22720	91.3	115	2	5	13012	13122	71303	71413	4E-36	150
tig22748	95.16	124	6	0	8174	8297	295483	295606	3E-50	196
tig22983	98.6	285	4	0	12484	12768	93237	93521	6E-143	505
tig23185	93.22	118	8	0	4673	4790	323912	323795	7E-44	174
tig23255	99.42	173	0	1	5815	5987	117952	118123	2E-85	313
tig23312	96.55	348	12	0	35389	35736	80361	80708	3E-164	577
tig23376	85.27	292	23	10	1483	1770	63597	63872	7E-77	283
tig23420	96.47	340	10	2	9155	9493	156864	156526	8E-160	560
tig23421	96.24	372	10	4	931	1301	155834	155466	2E-173	606

tig23512	94.06	202	2	10	19782	19973	358445	358244	1E-80	298
tig23555	89.32	103	11	0	6972	7074	159819	159921	8E-30	130
tig23566	93.24	222	10	2	843	1059	70205	69984	8E-89	322
tig23567	94.59	222	7	3	4	221	170353	170573	2E-92	339
tig2372	90.23	133	13	0	904	1036	382965	382833	2E-44	174
tig2410	91.84	515	23	6	5653	6149	89372	89885	0	701
tig24319	76.24	181	22	12	4926	5086	499236	499057	3E-14	76.8
tig24413	86.43	199	14	4	215	412	43412	43598	4E-54	206
tig24516	92.57	269	11	3	1895	2159	132723	132986	1E-104	377
tig24516	92.57	269	11	3	1895	2159	219001	219264	1E-104	377
tig24633	97.71	131	3	0	551	681	338177	338047	1E-59	226
tig24853	95.54	202	8	1	1491	1691	78163	77962	2E-88	322
tig24877	95.06	263	9	4	1238	1497	470050	469789	1E-115	411
tig24878	91.61	918	67	3	7	918	468914	468001	0	1260
tig25030	96.69	302	8	2	7653	7952	198680	198379	6E-142	501
tig2526	97.93	242	0	1	1	242	32442	32206	5E-116	414
tig25449	94.32	352	18	2	1334	1683	188749	188398	7E-154	538
tig25674	96.92	130	4	0	8319	8448	292176	292047	5E-57	219
tig25718	91.84	282	18	1	5504	5785	165679	165403	8E-108	388
tig25763	99.19	369	0	3	8507	8872	298182	298550	0	662
tig26001	97.74	133	1	2	14798	14929	322331	322200	3E-59	228
tig26256	96.9	129	4	0	1086	1214	73349	73221	4E-57	217
tig26377	99.52	207	1	0	2005	2211	41219	41013	3E-105	377
tig26378	99.09	220	1	1	1	220	357368	357150	3E-110	394
tig26635	97.87	141	2	1	1	140	129161	129021	5E-64	243
tig26644	98.19	276	5	0	75	350	427283	427558	2E-136	483

tig26653	93.09	217	10	2	13288	13499	370755	370971	7E-85	313
tig26867	97.48	159	4	0	3392	3550	321159	321317	6E-73	272
tig2698	94.93	217	8	1	14263	14476	125876	126092	2E-92	337
tig27383	92.96	142	10	0	19643	19784	294552	294411	3E-53	207
tig2761	98.83	171	2	0	22413	22583	449962	449792	1E-82	305
tig28014	96.3	108	2	2	5246	5351	368561	368668	1E-43	176
tig2836	96.88	160	5	0	1345	1504	84747	84588	5E-72	268
tig28362	99.56	227	1	0	1	227	129549	129323	3E-115	414
tig28461	96.84	158	3	1	2330	2485	226393	226550	1E-70	263
tig28469	97.7	174	0	4	1001	1171	417006	417178	3E-81	296
tig28521	89.68	126	7	5	1514	1633	272765	272890	1E-38	156
tig28600	80.54	257	20	13	911	1146	405795	406042	2E-43	171
tig28732	87.13	513	40	8	2	490	485756	486266	1E-159	558
tig2937	78.43	102	12	3	388	479	143888	143787	1E-08	58.4
tig29473	94.32	176	5	3	449	624	450560	450390	1E-70	265
tig29553	96.67	150	3	1	33732	33881	457788	457641	3E-65	248
tig29559	91.52	1132	60	13	2080	3178	126835	125707	0	1526
tig29798	91.95	149	3	2	4835	4974	203388	203536	1E-51	200
tig30012	98.38	185	3	0	14581	14765	158997	159181	6E-89	326
tig30094	97.28	405	11	0	10087	10491	1685	1281	0	688
tig30127	93.33	135	8	1	4534	4667	320394	320260	4E-51	198
tig30166	97.51	362	8	1	8398	8758	194429	194790	4E-177	617
tig30263	89.72	107	7	1	22156	22262	211601	211499	9E-31	134
tig30507	97.47	277	7	0	1	277	30497	30773	2E-134	473
tig30520	85.65	230	33	0	145	374	485602	485373	4E-65	243
tig30636	98.09	262	0	4	13334	13590	318948	319209	8E-127	451

tig3069	92.59	216	16	0	4614	4829	183297	183082	2E-84	311
tig30815	92.8	236	17	0	1645	1880	168712	168947	6E-95	342
tig30821	94.02	117	6	1	355	471	136063	135948	5E-44	176
tig3083	94.21	121	7	0	1212	1332	6577	6697	1E-47	185
tig3083	95.69	116	4	1	997	1112	164055	164169	1E-47	185
tig31332	94.23	104	6	0	306	409	192255	192358	9E-40	159
tig314	98.78	328	2	2	1	327	285770	285444	3E-167	582
tig31417	98.3	176	3	0	2489	2664	432138	432313	1E-84	309
tig31749	95.45	286	12	1	2412	2696	458986	459271	6E-128	455
tig32270	99.21	126	1	0	10464	10589	446336	446211	2E-59	228
tig32287	96.65	328	6	4	1	326	474605	474929	2E-154	540
tig32484	92.86	112	8	0	920	1031	386296	386185	5E-40	163
tig325	99.39	328	2	0	791	1118	471370	471697	1E-170	595
tig32507	93.52	216	13	1	1169	1383	457370	457585	6E-87	320
tig32543	96.09	307	12	0	995	1301	267289	266983	7E-143	501
tig32587	94.51	164	9	0	398	561	57020	56857	2E-67	254
tig329	99.5	4031	3	9	1	4025	304744	308763	0	7317
tig33052	90.85	164	15	0	742	905	459354	459191	4E-58	220
tig33087	99.11	112	1	0	29270	29381	27213	27102	2E-51	202
tig33239	95.45	110	5	0	7481	7590	32870	32979	5E-44	176
tig334	98.18	274	2	1	3181	3451	336300	336573	1E-134	475
tig33621	89.31	159	13	2	2182	2336	330172	330330	2E-50	196
tig33681	98.76	161	1	1	1	161	130666	130825	4E-77	285
tig33708	88.1	126	15	0	14502	14627	42963	43088	5E-36	150
tig33754	94.59	185	9	1	325	509	311341	311524	1E-77	285
tig33771	91.67	120	7	2	2426	2542	156843	156962	7E-41	163

tig33845	96.06	203	7	1	21076	21277	48783	48985	6E-90	329
tig33876	97.72	219	5	0	4260	4478	470828	471046	2E-104	377
tig33948	95.41	109	5	0	7561	7669	139095	139203	1E-43	174
tig340	99.57	8108	8	10	1	8082	363057	371163	0	14754
tig341	94.58	203	0	4	394	596	100142	99951	2E-83	303
tig3442	97.12	347	10	0	12788	13134	200113	200459	2E-167	586
tig34464	93.18	381	21	3	6388	6764	381123	380744	3E-158	555
tig3475	98.32	297	5	0	5009	5305	156639	156343	2E-148	521
tig3476	99.21	252	2	0	5613	5864	421440	421691	9E-128	455
tig348	97.13	244	6	1	379	621	394884	395127	3E-114	411
tig35051	84.16	101	16	0	2701	2801	148873	148973	2E-20	99
tig3506	98.39	124	2	0	20229	20352	173473	173596	1E-56	219
tig35173	99.33	297	1	1	1	297	447801	448096	1E-153	536
tig3518	97.55	286	7	0	333	618	71354	71069	1E-139	490
tig3519	99.64	1373	0	2	1	1368	210116	211488	0	2503
tig3522	98.23	283	5	0	331	613	131989	132271	4E-141	496
tig3522	98.23	283	5	0	331	613	218267	218549	4E-141	496
tig35257	99.42	345	0	2	5613	5956	8758	9101	2E-179	625
tig35341	99.5	199	1	0	503	701	172944	172746	3E-101	363
tig35517	95.43	219	2	4	9915	10131	175689	175477	6E-94	342
tig35565	98.73	237	2	1	1	236	440588	440352	2E-117	420
tig35665	92.17	166	11	1	1797	1960	193149	192984	1E-61	233
tig3590	99.62	265	0	1	1548	1812	40350	40613	1E-136	483
tig36252	99.14	233	0	2	1	233	260789	261019	4E-117	418
tig3629	95.22	314	7	3	3066	3375	491950	491641	2E-138	490
tig36330	99.19	124	1	0	330	453	123979	123856	2E-59	224

tig36340	94.44	162	9	0	13369	13530	403807	403968	3E-66	250
tig36383	97	100	2	1	16418	16517	41256	41354	6E-41	167
tig365	92.66	218	11	3	1918	2130	396796	397013	7E-85	309
tig3652	95.65	161	7	0	1182	1342	459383	459223	6E-70	259
tig36592	92.31	182	12	1	875	1054	213777	213596	2E-68	257
tig36610	99.06	425	3	1	1	424	381270	381694	0	761
tig36711	89	100	7	2	2808	2906	286925	286829	1E-27	121
tig36725	95.24	105	5	0	774	878	124340	124444	2E-41	167
tig36729	97.12	104	3	0	418	521	474111	474008	2E-44	176
tig368	92.31	182	13	1	267	448	464247	464427	1E-69	257
tig36879	98	150	3	0	1038	1187	421254	421403	3E-70	261
tig37038	91.95	149	9	2	6134	6282	209385	209240	3E-53	206
tig37162	99.5	199	1	0	518	716	130683	130485	3E-101	363
tig3723	91.46	574	27	13	872	1433	287750	288313	0	769
tig37310	90.85	142	6	3	1	139	453399	453262	1E-46	183
tig37369	92.12	330	16	1	6854	7183	210320	210001	9E-129	457
tig37507	97.37	152	4	0	5140	5291	34154	34305	2E-69	259
tig37522	97.72	219	5	0	349	567	470828	471046	1E-104	377
tig37875	99.38	323	2	0	1	323	214548	214226	3E-167	586
tig38145	96.58	234	8	0	6366	6599	331705	331938	5E-108	388
tig38383	99.3	143	1	0	9084	9226	333956	334098	3E-69	259
tig38386	96.98	398	8	2	1	395	158627	158231	0	665
tig38400	98.94	188	2	0	2105	2292	155320	155507	7E-93	337
tig38439	99.6	253	1	0	255	507	457746	457494	3E-130	462
tig38522	92.27	220	14	2	8897	9113	107805	107586	3E-84	309
tig38522	92.27	220	14	2	8897	9113	308660	308441	3E-84	309

tig38696	95.35	215	7	1	27911	28122	132986	132772	1E-92	339
tig38696	95.35	215	7	1	27911	28122	219264	219050	1E-92	339
tig38838	97.32	261	2	5	15913	16171	421198	420941	7E-123	438
tig39510	98.08	156	3	0	4357	4512	76153	75998	2E-73	272
tig39732	95.04	121	6	0	12276	12396	210841	210961	4E-48	191
tig39796	92.73	110	7	1	3898	4007	205227	205119	2E-38	158
tig40010	93.16	117	8	0	2877	2993	67670	67786	2E-43	172
tig40106	99.41	339	0	2	5895	6232	278578	278241	4E-176	614
tig40142	90.6	500	31	5	1135	1629	358765	358277	0	649
tig40144	99.29	140	0	1	652	791	48659	48797	6E-68	252
tig40144	99.29	140	0	1	652	791	346280	346418	6E-68	252
tig40492	99.15	234	2	0	292	525	285887	285654	6E-119	422
tig40495	96.57	233	7	1	7800	8032	217258	217027	5E-107	385
tig40583	99.3	142	1	0	1	142	126271	126412	2E-69	257
tig4099	97.14	105	3	0	5177	5281	15855	15751	6E-45	178
tig41043	94.44	144	6	1	931	1074	3315	3174	8E-58	220
tig41102	94.44	252	12	2	337	587	392531	392781	4E-108	387
tig41146	88.48	217	16	4	1185	1393	468867	468652	2E-68	254
tig41869	98.17	328	2	4	7844	8170	376185	375861	1E-162	569
tig41898	94.3	193	10	1	441	632	35975	36167	2E-80	294
tig42449	98.81	168	2	0	427	594	430868	431035	2E-82	300
tig42746	97.04	1015	15	3	17835	18834	147152	146138	0	1694
tig42843	98.86	175	2	0	357	531	229820	229994	3E-85	313
tig43041	97.54	285	7	0	528	812	22684	22400	3E-138	488
tig43206	99.59	243	1	0	6310	6552	412952	412710	7E-125	444
tig43718	98.19	166	3	0	1	166	100127	100292	1E-78	291

tig43740	97.04	372	11	0	1	372	358552	358181	0	627
tig43742	99.56	227	1	0	17529	17755	93198	93424	1E-115	414
tig43930	94.67	244	13	0	1326	1569	183549	183792	3E-105	379
tig44173	96.8	281	3	6	6146	6424	418151	417875	4E-131	464
tig44468	91.39	267	19	3	6435	6699	147342	147078	3E-100	363
tig44603	87.94	1119	84	18	241	1333	125462	124369	0	1271
tig44749	99	200	2	0	2099	2298	418185	417986	9E-100	359
tig45073	88.79	116	7	2	857	966	15033	14918	2E-32	137
tig45111	89.63	164	17	0	21414	21577	334182	334019	8E-54	209
tig45429	99.24	132	1	0	7645	7776	270127	270258	4E-63	239
tig4590	93.07	361	25	0	5354	5714	392518	392158	3E-150	529
tig45937	99.12	227	1	1	859	1085	883	658	1E-114	407
tig46272	98.97	292	3	0	4	295	330613	330904	6E-149	523
tig46425	99.21	254	2	0	423	676	131301	131048	1E-129	459
tig46425	99.21	254	2	0	423	676	217579	217326	1E-129	459
tig46518	88.11	244	10	7	904	1135	44351	44587	5E-74	272
tig46519	89.9	208	9	6	24	219	44380	44587	2E-69	257
tig46637	93.97	116	6	1	9610	9724	151401	151286	3E-43	174
tig46781	98.43	191	3	0	459	649	48018	47828	2E-92	337
tig46825	98.84	173	1	1	19643	19814	200377	200549	2E-83	307
tig46876	93.02	215	7	3	817	1031	342287	342493	2E-84	307
tig47305	98.31	177	3	0	1152	1328	140733	140557	2E-85	311
tig47305	98.31	177	3	0	1152	1328	493761	493585	2E-85	311
tig47412	87.35	253	17	5	3020	3257	135682	135934	4E-74	276
tig47785	99.63	272	1	0	379	650	382609	382880	7E-142	497
tig47786	97.38	191	3	2	1	190	384938	385127	2E-88	324

tig47818	99.31	288	0	2	17904	18189	347587	347300	3E-147	520
tig47876	98.72	156	1	1	1	155	71934	71779	4E-75	276
tig481	93.9	164	9	1	1186	1348	430891	430728	3E-65	246
tig48280	99.61	259	1	0	6023	6281	462675	462417	7E-134	473
tig48672	99.15	236	0	2	1	236	292100	291867	7E-119	424
tig48838	95.1	286	11	2	1659	1942	367236	366952	2E-126	448
tig48887	99.15	351	2	1	1372	1721	163700	163350	0	630
tig48976	98.47	261	4	0	1	261	137709	137969	1E-129	460
tig49013	96.06	127	5	0	1	127	268039	268165	4E-54	207
tig4902	95.9	244	3	7	23217	23456	392011	392251	1E-107	388
tig49060	96.64	119	4	0	631	749	445691	445573	7E-52	198
tig49327	95.43	197	9	0	16054	16250	320873	321069	1E-85	315
tig49541	98.49	199	3	0	22318	22516	496350	496548	2E-96	351
tig49552	99.37	317	2	0	357	673	492406	492722	5E-164	575
tig49859	94.29	105	5	1	132	235	38954	39058	4E-39	159
tig50069	96.49	114	4	0	7730	7843	197393	197506	4E-48	189
tig50070	97.35	151	4	0	1	151	70114	70264	3E-69	257
tig50897	89.74	117	7	2	6566	6677	76469	76353	9E-35	145
tig50902	96.83	126	2	2	6134	6258	137702	137578	2E-54	209
tig50968	97.93	145	0	3	1	142	412501	412645	1E-65	248
tig51095	98.51	134	2	0	1732	1865	87127	86994	2E-62	237
tig515	95.24	105	5	0	854	958	40725	40829	5E-42	167
tig5167	91.67	168	7	1	1958	2125	54574	54734	4E-59	226
tig51680	98.05	154	3	0	259	412	303513	303666	6E-73	268
tig52131	98.43	255	4	0	2155	2409	68653	68399	2E-126	449
tig5252	91.84	147	8	1	6587	6733	272680	272538	5E-52	202

tig52613	97.32	149	4	0	1059	1207	258661	258513	4E-68	254
tig52855	99.64	279	1	0	1	279	13423	13145	3E-145	510
tig52856	92.39	197	0	4	3960	4155	363947	364129	8E-72	267
tig52947	84	100	9	7	1693	1788	93287	93191	2E-18	89.8
tig53001	92.31	208	12	1	1847	2054	149086	149289	3E-79	292
tig53002	95.24	126	6	0	174	299	153917	154042	8E-52	200
tig53025	96.4	250	9	0	1085	1334	78861	79110	4E-116	412
tig53290	88.81	134	3	3	618	743	178711	178582	1E-37	154
tig53391	92.74	427	25	4	1107	1531	177426	177848	7E-176	612
tig53893	99.21	378	3	0	2580	2957	216860	217237	0	682
tig54050	98.79	248	3	0	673	920	26815	27062	3E-125	442
tig54222	98.34	181	0	3	1	178	396728	396908	7E-87	315
tig54261	99.47	190	1	0	1	190	364030	363841	3E-95	346
tig54463	92.02	188	8	2	2221	2403	363749	363934	4E-69	257
tig549	99.64	281	1	0	773	1053	169663	169943	7E-147	514
tig54994	94.23	104	2	3	1836	1939	429679	429580	1E-38	156
tig55087	94.69	113	6	0	100	212	428382	428270	9E-45	176
tig55121	98.86	176	0	2	1	176	428386	428213	2E-85	313
tig55317	98.67	226	0	3	8113	8335	179455	179680	6E-111	398
tig55440	98.77	243	2	1	8521	8762	273831	273589	6E-121	431
tig55800	99.18	243	1	1	1	243	6228	5987	1E-122	436
tig55925	98.28	174	3	0	1	174	319948	319775	4E-83	305
tig5624	97.69	216	5	0	28921	29136	488509	488294	2E-102	372
tig56457	93.09	217	4	1	7267	7483	79892	80097	9E-84	307
tig5672	97.02	336	10	0	1	336	342034	342369	9E-162	566
tig56779	97.56	246	6	0	1	246	226734	226489	3E-118	422

tig5679	93.29	298	17	2	17187	17481	144388	144685	3E-122	436
tig56855	92.38	105	7	1	3856	3960	275512	275615	6E-36	148
tig5696	98.21	168	1	2	202	368	8770	8936	2E-79	292
tig56961	98.88	179	1	1	392	570	228194	228371	6E-88	318
tig57207	99.38	162	1	0	348	509	285869	286030	1E-80	294
tig57686	90.45	157	6	3	382	536	175106	174957	1E-51	198
tig5780	87.5	152	19	0	2505	2656	22311	22160	6E-44	176
tig58128	94.56	147	7	1	5959	6105	179612	179757	2E-59	226
tig58131	98.96	386	0	4	1	383	420277	420661	0	688
tig59078	98.67	376	4	1	1	375	88819	89194	0	665
tig5913	97.93	145	3	0	515	659	82464	82608	6E-68	252
tig59641	96.38	138	2	3	1	135	449163	449300	6E-59	224
tig5983	92.8	125	6	3	7477	7598	290430	290306	1E-44	178
tig5983	92.8	125	6	3	7477	7598	353892	354016	1E-44	178
tig60117	98.55	138	2	0	21	158	68892	69029	2E-65	244
tig60142	96.95	164	3	2	1	163	105941	106103	6E-74	274
tig60174	98.25	229	1	3	4484	4709	335815	336043	3E-111	398
tig602	89.83	118	0	8	3	114	139357	139468	1E-33	141
tig6036	93.7	127	3	1	11904	12030	421439	421560	7E-47	185
tig60385	95.07	203	3	7	1	198	122977	123177	1E-85	313
tig60589	97	100	3	0	938	1037	8796	8697	1E-42	169
tig60818	88.49	139	6	5	151	282	148114	147979	5E-40	159
tig60875	98.5	133	2	0	3773	3905	162359	162227	2E-62	235
tig61398	89.42	189	14	2	814	1000	421553	421369	2E-62	233
tig61399	92	125	10	0	4	128	421430	421306	7E-45	176
tig61502	90.38	156	15	0	1363	1518	37535	37380	1E-53	206

tig61615	98.15	108	2	0	1717	1824	326254	326361	1E-48	189
tig61691	95.99	324	13	0	1289	1612	421235	421558	2E-150	527
tig61693	94.84	213	11	0	1428	1640	444497	444285	4E-92	333
tig61710	99.53	215	0	1	1	215	265675	265462	3E-109	390
tig61773	95.69	394	17	0	1514	1907	466206	465813	0	634
tig61807	99.03	103	1	0	1419	1521	193337	193439	1E-47	185
tig61843	99.26	271	0	2	1706	1975	264505	264774	8E-139	488
tig61905	82.43	239	17	10	75	299	173189	173416	1E-47	185
tig61921	96.89	193	1	4	1657	1846	32370	32180	1E-87	318
tig61999	97.33	225	5	1	1524	1747	281952	282176	1E-106	381
tig62131	96.69	121	4	0	1207	1327	328404	328524	9E-53	202
tig6224	87.74	106	13	0	9505	9610	433699	433594	2E-28	124
tig6225	91.67	132	10	1	541	672	460374	460244	4E-46	182
tig62291	97.12	139	3	1	878	1015	4350	4488	3E-62	233
tig62375	89.43	123	12	1	721	842	92592	92714	2E-38	154
tig62399	96.46	113	3	1	981	1092	464670	464558	8E-48	185
tig62431	97.73	176	3	1	49	223	432313	432138	8E-83	302
tig62454	99.36	156	1	0	913	1068	60339	60494	3E-77	283
tig62459	97.63	253	4	1	56	308	58271	58021	3E-122	433
tig62465	98.18	385	6	1	41	424	480360	479976	0	671
tig62494	99.58	239	0	1	6	243	277423	277661	7E-123	435
tig62548	99.56	228	1	0	238	465	167316	167543	3E-117	416
tig62607	99.01	302	0	3	1042	1341	387659	387959	5E-154	538
tig62959	97.6	125	3	0	427	551	234152	234276	9E-57	215
tig62974	96.58	117	0	4	1115	1228	400401	400286	2E-49	191
tig63007	88.08	193	23	0	669	861	420577	420769	3E-61	230

tig63061	99.52	207	0	1	1	206	304421	304627	4E-105	375
tig63245	99.28	278	2	0	1	278	49095	48818	2E-143	503
tig63375	99.12	114	1	0	268	381	135616	135503	5E-54	206
tig6343	99.35	155	0	1	1	154	272297	272143	4E-75	279
tig63503	94.51	237	9	4	889	1123	237619	237853	3E-101	363
tig63596	98.53	273	0	4	842	1110	40767	41039	3E-136	479
tig63683	99.46	185	0	1	910	1094	57694	57511	6E-93	335
tig64326	98.94	283	1	2	743	1024	215455	215736	4E-144	505
tig6602	94.5	109	6	0	317	425	274059	274167	1E-42	169
tig6679	98.98	393	1	3	1	391	384441	384832	0	701
tig6921	98.23	282	4	1	4033	4313	334097	334378	5E-139	492
tig6972	99.42	342	2	0	1	342	161529	161188	7E-179	621
tig7009	99.27	275	2	0	1244	1518	361966	361692	2E-141	497
tig7046	98.02	253	5	0	1318	1570	70177	70429	3E-124	440
tig7055	98.6	143	0	2	2	143	430205	430346	6E-68	252
tig7057	89.34	441	27	7	1493	1931	434425	434003	1E-152	536
tig7058	91.51	648	40	8	268	907	209333	209973	0	878
tig7332	99.1	111	1	0	120	230	278011	277901	1E-51	200
tig7452	85.59	111	10	2	2875	2979	15033	14923	8E-25	111
tig7491	90.28	144	3	7	676	814	313898	314035	1E-44	178
tig7542	96.84	190	6	0	6806	6995	172826	172637	5E-87	318
tig7543	98.75	320	3	1	5268	5586	29893	30212	3E-162	568
tig7546	94.63	298	16	0	79	376	177234	177531	3E-131	462
tig7548	98.96	193	2	0	579	771	25343	25151	8E-96	346
tig7554	97.14	385	5	5	1	379	142918	142534	0	645
tig7554	97.14	385	5	5	1	379	495946	495562	0	645

tig7629	99.04	208	0	2	1	207	269724	269930	2E-103	372
tig7647	96.92	227	5	2	3086	3312	55246	55022	2E-105	379
tig8016	84	150	5	8	284	432	281859	281990	2E-29	126
tig8044	90.86	197	16	2	13307	13502	34459	34264	4E-70	263
tig8118	97.44	313	8	0	6672	6984	191334	191646	4E-152	534
tig8119	96.6	353	8	1	60	408	191334	191686	4E-166	582
tig8336	93.55	124	7	1	1115	1237	404225	404348	4E-46	183
tig8431	98.48	132	2	0	673	804	207015	206884	4E-62	233
tig8730	94.59	111	1	1	14784	14894	428827	428722	3E-41	167
tig8731	97.17	106	1	1	38	141	428827	428722	2E-44	178
tig8845	97.74	265	6	0	8040	8304	496736	496472	1E-128	457
tig8889	93.85	179	9	2	18657	18834	51914	52091	1E-71	268
tig890	99.33	150	1	0	2306	2455	204005	203856	1E-73	272
tig8992	98.77	163	0	2	3616	3778	261114	261274	2E-78	289
tig9027	79.83	233	21	9	147	353	14966	15198	3E-35	147
tig9056	99.56	226	0	1	8053	8278	345630	345406	2E-114	411
tig9429	84.91	212	20	7	5319	5527	83066	82864	5E-52	204
tig9445	97.27	110	0	3	1	109	2035	2142	7E-46	183
tig9469	98.31	178	3	0	8343	8520	203412	203589	2E-85	313
tig9585	96.15	156	1	1	1138	1288	89832	89987	3E-66	250
tig9687	97.32	112	3	0	2809	2920	271442	271553	2E-48	191
tig9744	90.21	143	1	1	10869	10998	87856	87998	3E-43	174
tig9747	81.82	253	26	12	183	416	333967	333716	7E-50	195
tig9817	98.85	262	3	0	18190	18451	468994	468733	1E-131	468
tig9820	88.06	134	7	5	13403	13535	23534	23409	9E-36	150
tig9826	95.8	262	11	0	982	1243	344333	344594	1E-119	424

tig9827	92.77	318	17	3	1	317	344937	345249	3E-128	455
tig9833	94	300	18	0	35	334	176421	176720	1E-128	455
tig985	99.62	265	1	0	592	856	111695	111959	6E-138	484
tig9851	88.93	244	8	7	631	862	44587	44351	2E-77	283
tig9863	91.43	502	26	7	5005	5496	488559	488065	0	673
tig991	89.2	324	9	10	671	983	130583	130891	8E-107	381

Table S7 Comparison of RNA-editing sites identified in the NUMT and the mitogenome (see the excel file).

Table S8 List of 115 Symmetrical RNA editing sites in the CDS regions on the mitogenome of *S. miltiorrhiza*.

RNA Editing Site Names	Genomic Position	Type of REE[a] on SS[b]	Frequency of REE on SS	Type of REE on AS[c]	Frequency of REE on AS
matR-1744	6086	CT	1	GA	1
matR-1708	6122	CT	1	GA	1
mttB-687	9277	CT	0.71	GA	0.75
mttB-682	9282	CT	0.96	GA	1
mttB-394	9570	CT	0.75	GA	0.79
mttB-391	9573	CT	0.83	GA	0.77
mttB-388	9576	CT	0.75	GA	0.77
cob-1084	19394	CT	1	GA	1
cob-358	20120	CT	1	GA	1
cox2-745	27294	CT	1	GA	0.91
cox2-724	27315	CT	1	GA	0.9
cox2-632	30484	CT	1	GA	0.9
nad5-359	35588	CT	1	GA	1
nad5-374	35603	CT	1	GA	1

nad5-398	35627	CT	1	GA	1
nad5-539	35768	CT	1	GA	0.54
nad5-608	35837	CT	1	GA	0.79
nad5-676	35905	CT	1	GA	0.89
nad5-835[d]	36064	CT	0.93	GA	0.96
nad5-1310	36539	CT	0.64	GA	1
nad9-113	44150	CT	1	GA	0.86
nad6-161	49783	CT	0.9	GA	0.98
nad6-169	49791	CT	0.88	GA	0.98
nad6-191	49813	CT	0.88	GA	1
rpl23-71	144796	CT	0.86	GA	0.84
rpl23-89	144814	CT	0.76	GA	1
rpl16-146	168359	CT	1	GA	0.98
rpl16-449	168662	CT	0.99	GA	0.98
rps3-1558[d]	168841	CT	0.99	GA	0.85
rps3-1525	168874	CT	0.81	GA	0.67
nad1-376	179757	CT	1	GA	1
nad1-308	179825	CT	0.82	GA	1
nad1-307	179826	CT	0.73	GA	1
nad1-265	179868	CT	1	GA	1
nad5-1526	204918	CT	1	GA	1
nad5-1544	204936	CT	1	GA	1
nad5-1586	204978	CT	1	GA	1
nad5-1892	206324	CT	1	GA	1
nad5-1894	206326	CT	1	GA	1
nad5-1934	206366	CT	1	GA	1

nad1-919	266013	CT	1	GA	0.84
nad1-889	266043	CT	1	GA	0.82
nad1-814	266118	CT	0.91	GA	1
nad2-359	273517	CT	1	GA	1
nad4L-281	292089	CT	0.94	GA	1
atp4-59	292338	CT	1	GA	1
atp4-71	292350	CT	1	GA	1
atp4-89	292368	CT	1	GA	1
nad4-1424	318223	CT	0.48	GA	0.66
nad4-1408	318239	CT	0.66	GA	0.79
nad4-1346	321138	CT	1	GA	1
nad4-1298	321186	CT	1	GA	1
nad4-1142	321342	CT	1	GA	1
nad4-1007	321477	CT	1	GA	0.78
nad4-1001	321483	CT	1	GA	1
nad4-878	324209	CT	0.86	GA	0.67
nad4- 848	324239	CT	0.72	GA	0.73
cox1-452	343908	CT	1	GA	1
cox1-515	343971	CT	1	GA	1
cox1-551	344007	CT	1	GA	1
cox1-590	344046	CT	1	GA	1
orf304-756	345019	CT	1	GA	1
orf304-774	345037	CT	1	GA	1
cox1-1405	345822	CT	1	GA	1
cox1-1433	345850	CT	0.9	GA	0.96
cox1-1489	345906	CT	0.89	GA	0.95

rps12-284	395155	CT	1	GA	1
rps12-221	395218	CT	1	GA	1
rps12-196	395243	CT	1	GA	1
rps12-159	395280	CT	0.9	GA	0.98
nad3-349	395495	CT	1	GA	0.98
nad3-344	395500	CT	1	GA	0.99
nad3-317	395527	CT	0.99	GA	1
nad3-275	395569	CT	0.98	GA	1
nad3-266	395578	CT	0.97	GA	1
nad3-230	395614	CT	0.96	GA	0.95
nad3-215	395629	CT	0.96	GA	0.95
nad3-209	395635	CT	1	GA	0.95
nad3-208	395636	CT	1	GA	0.95
nad3-146	395698	CT	0.97	GA	0.95
nad3-124	395720	CT	0.95	GA	1
nad3-80	395764	CT	1	GA	1
nad3-79	395765	CT	1	GA	1
nad3-62	395782	CT	1	GA	1
nad3-44	395800	CT	1	GA	1
rpl5-414	412234	CT	0.62	GA	0.38
orf214-305	420919	CT	0.71	GA	0.68
atp9-212	421266	CT	1	GA	1
nad7-77	427622	CT	1	GA	0.94
nad7-137	427682	CT	1	GA	1
nad7-724	430457	CT	1	GA	1
nad7-739	430472	CT	1	GA	0.86

nad7-740	430473	CT	1	GA	1
nad7-1050	432125	CT	0.92	GA	1
nad7-1057	432132	CT	0.71	GA	1
nad7-1103	432178	CT	0.88	GA	1
orf456-1236	468310	CT	0.45	GA	0.3
orf456-1185	468361	CT	1	GA	1
orf456-1090	468456	CT	1	GA	1
orf456-1072	468474	CT	1	GA	1
orf456-897	468649	CT	1	GA	1
orf456-684	468862	CT	1	GA	0.98
orf456-474	469072	CT	0.97	GA	1
orf456-375	469171	CT	1	GA	0.92
ccmFn-1519	472504	CT	1	GA	1
ccmFn-1484	472539	CT	0.95	GA	1
ccmFn-1472	472551	CT	0.96	GA	1
cox3-764	484425	CT	0.93	GA	0.82
cox3-754	484435	CT	1	GA	0.91
cox3-422	484767	CT	1	GA	0.98
cox3-419	484770	CT	1	GA	1
cox3-314	484875	CT	1	GA	0.85
cox3-311	484878	CT	0.99	GA	0.84
cox3-304	484885	CT	0.97	GA	0.85
cox3-245	484944	CT	0.8	GA	0.88

[a]REE: RNA editing events; [b]SS: Sense strand; [c]AS: antisense strand. [d]Validated by Strand-specific RT-PCR method.

Table S9 RNA-editing sites found among homologous CDS from the mitogenomes of *A. thaliana*, *O. sativa* and *S. miltiorrhiza* (see the excel file).

Table S10 List of symmetrical RNA editing events found in the CDS regions on the mitogenome of *A. thaliana*.

RNA editing site	Genomic Position	Type of REEs on SS	Frequency of REEs on SS	Type of REEs on AS	Frequency of REEs on AS
nad5-1958	20623	CT	0.71	GA	0.92
nad5-1918	20663	CT	0.44	GA	0.93
nad5-1916	20665	CT	0.65	GA	0.98
nad5-1895	20686	CT	0.44	GA	0.71
nad5-1731	21824	CT	0.21	GA	0.19
nad5-1665	21890	CT	0.15	GA	0.11
nad5-1550	22005	CT	0.99	GA	0.97
nad9-439	23797	CT	0.98	GA	0.97
nad9-398	23838	CT	0.98	GA	0.99
nad9-298	23938	CT	0.92	GA	0.88
nad9-167	24069	CT	0.93	GA	0.97
rpl16-506	25110	CT	0.97	GA	0.96
rpl16-440	25176	CT	0.97	GA	1

rpl16-209	25407	CT	0.97	GA	0.94
rps3-1598	25555	CT	0.89	GA	0.91
rps3-1571	25582	CT	0.89	GA	0.8
rps3-1534	25619	CT	0.72	GA	0.54
rps3-1470	25683	CT	0.85	GA	0.8
rps3-1344	25809	CT	0.4	GA	0.32
rps3-887	26266	CT	0.82	GA	0.83
rps3-603	26550	CT	0.82	GA	0.64
rps3-64	28670	CT	0.61	GA	0.38
ccb206-16	30478	CT	0.82	GA	0.49
ccb206-28	30490	CT	0.75	GA	0.51
ccb206-71	30533	CT	0.63	GA	0.75
ccb206-80	30542	CT	0.42	GA	0.69
ccb206-304	30766	CT	0.9	GA	0.47
ccb206-338	30800	CT	0.88	GA	0.42
ccb206-367	30829	CT	0.86	GA	0.5
ccb206-379	30841	CT	0.84	GA	0.55
ccb206-380	30842	CT	0.82	GA	0.55
ccb206-406	30868	CT	0.28	GA	0.22
ccb206-424	30886	CT	0.66	GA	0.86
ccb206-428	30890	CT	0.66	GA	0.86
ccb206-467	30929	CT	0.52	GA	0.9
ccb206-475	30937	CT	0.54	GA	0.9
ccb206-476	30938	CT	0.54	GA	0.9
ccb206-485	30947	CT	0.46	GA	0.89
cox2-742	40543	CT	0.94	GA	0.99

cox2-721	40564	CT	0.96	GA	0.98
cox2-581	42048	CT	0.97	GA	0.98
cox2-557	42072	CT	1	GA	0.99
cox2-379	42250	CT	0.97	GA	0.97
cox2-278	42351	CT	0.98	GA	0.92
cox2-253	42376	CT	0.97	GA	0.95
cox2-138	42491	CT	0.78	GA	0.63
cox2-27	42602	CT	0.77	GA	0.78
cox2-25	42604	CT	0.62	GA	0.61
ccb452-1327	51325	CT	0.71	GA	0.9
ccb452-1280	51372	CT	0.52	GA	0.41
ccb452-1246	51406	CT	0.51	GA	0.51
ccb452-1215	51437	CT	0.47	GA	0.43
ccb452-1172	51480	CT	0.87	GA	0.89
ccb452-561	53051	CT	0.38	GA	0.19
ccb452-415	53197	CT	0.57	GA	0.7
ccb452-406	53206	CT	0.52	GA	0.62
ccb452-378	53234	CT	0.12	GA	0.15
ccb452-334	53278	CT	0.79	GA	0.57
ccb452-333	53279	CT	0.69	GA	0.46
ccb452-175	53437	CT	0.23	GA	0.31
ccb452-160	53452	CT	0.31	GA	0.42
ccb452-155	53457	CT	0.61	GA	0.6
ccb452-146	53466	CT	0.72	GA	0.66
ccb452-123	53489	CT	0.59	GA	0.44
ccb452-122	53490	CT	0.76	GA	0.65

rpl5-35	57808	CT	0.79	GA	0.74
rpl5-58	57831	CT	0.5	GA	0.31
rpl5-59	57832	CT	0.81	GA	0.6
rpl5-64	57837	CT	0.5	GA	0.56
rpl5-92	57865	CT	0.49	GA	0.63
rpl5-169	57942	CT	0.82	GA	0.81
rpl5-317	58090	CT	0.93	GA	0.8
rpl5-329	58102	CT	0.97	GA	0.9
rpl5-512	58285	CT	0.84	GA	0.88
cob-118	60352	CT	0.98	GA	0.98
cob-286	60520	CT	0.99	GA	0.98
cob-325	60559	CT	0.97	GA	0.95
cob-568	60802	CT	0.99	GA	0.99
cob-610	60844	CT	0.21	GA	0.12
cob-924	61158	CT	0.14	GA	0.17
cob-1160	61394	CT	0.32	GA	0.32
nad6-463	76797	CT	0.96	GA	0.93
nad6-446	76814	CT	0.99	GA	1
nad6-191	77069	CT	0.96	GA	0.99
nad6-161	77099	CT	0.91	GA	0.99
nad6-89	77171	CT	0.99	GA	0.96
nad6-88	77172	CT	0.99	GA	0.98
nad6-53	77207	CT	0.96	GA	0.9
nad6-26	77234	CT	0.99	GA	0.94
nad2-461	79857	CT	0.96	GA	0.98
nad2-394	79924	CT	0.98	GA	0.95

nad2-389	79929	CT	0.96	GA	0.93
nad2-344	79974	CT	0.95	GA	0.93
nad2-341	79977	CT	0.98	GA	0.96
nad2-90	81208	CT	0.69	GA	0.8
nad2-89	81209	CT	0.95	GA	0.97
nad2-28	81270	CT	0.64	GA	0.67
nad2-19	81279	CT	0.1	GA	0.13
rps4-1052	82065	CT	0.6	GA	0.78
rps4-1043	82074	CT	0.54	GA	0.67
rps4-1042	82075	CT	0.73	GA	0.81
rps4-992	82125	CT	0.92	GA	0.9
rps4-967	82150	CT	0.85	GA	0.78
rps4-956	82161	CT	0.94	GA	0.91
rps4-524	82593	CT	0.8	GA	0.8
rps4-377	82740	CT	0.8	GA	0.65
rps4-332	82785	CT	0.78	GA	0.64
rps4-308	82809	CT	0.77	GA	0.57
rps4-299	82818	CT	0.87	GA	0.68
rps4-235	82882	CT	0.62	GA	0.65
rps4-226	82891	CT	0.75	GA	0.71
rps4-175	82942	CT	0.71	GA	0.65
rps4-88	83029	CT	0.79	GA	0.55
rps4-77	83040	CT	0.76	GA	0.45
atp6-1-475	112224	CT	1	GA	0.98
orfB-10	129918	CT	0.31	GA	0.32
orfB-389	130297	CT	0.1	GA	0.18

nad7-24	132094	CT	0.97	GA	0.87
nad7-38	132108	CT	0.92	GA	0.8
nad7-77	132147	CT	0.98	GA	1
nad7-137	132207	CT	0.89	GA	1
nad7-200	133233	CT	0.9	GA	0.87
nad7-213	134309	CT	0.9	GA	0.85
nad7-244	134340	CT	0.99	GA	0.97
nad7-251	134347	CT	0.97	GA	0.94
nad7-335	134431	CT	0.96	GA	0.98
nad7-344	134440	CT	0.86	GA	0.88
nad7-698	135847	CT	0.79	GA	0.43
nad7-724	135873	CT	0.83	GA	0.63
nad7-734	135883	CT	0.91	GA	0.81
nad7-739	135888	CT	0.82	GA	0.62
nad7-769	135918	CT	0.9	GA	0.87
nad7-789	135938	CT	0.16	GA	0.11
nad7-795	135944	CT	0.55	GA	0.54
nad7-963	137931	CT	0.38	GA	0.77
nad7-1050	138018	CT	0.15	GA	0.1
nad7-1088	138056	CT	0.94	GA	0.84
nad7-1103	138071	CT	0.96	GA	0.9
nad7-1124	138092	CT	0.97	GA	0.85
nad7-1137	138105	CT	0.6	GA	0.61
nad5-1318	140852	CT	0.12	GA	0.11
nad5-1275	140895	CT	1	GA	1
nad5-1273	140897	CT	1	GA	1

nad5-875	141295	CT	0.95	GA	0.98
nad5-835	141335	CT	0.97	GA	0.97
nad5-801	141369	CT	0.34	GA	0.45
nad5-725	141445	CT	0.99	GA	0.93
nad5-676	141494	CT	0.98	GA	0.99
nad5-629	141541	CT	0.94	GA	0.99
nad5-609	141561	CT	0.86	GA	0.84
nad5-608	141562	CT	0.94	GA	0.97
nad5-598	141572	CT	0.96	GA	0.98
nad5-553	141617	CT	0.97	GA	0.94
nad5-548	141622	CT	0.97	GA	0.95
nad5-494	141676	CT	0.98	GA	0.97
nad5-398	141772	CT	0.98	GA	0.99
nad5-374	141796	CT	0.94	GA	0.99
nad5-358	141812	CT	0.97	GA	1
nad5-272	141898	CT	0.96	GA	0.9
nad1-937	143260	CT	0.94	GA	0.97
nad1-898	143299	CT	0.95	GA	0.98
nad1-823	143374	CT	0.97	GA	0.9
nad1-725	143472	CT	0.86	GA	0.97
matR-1895	144418	CT	0.94	GA	0.96
matR-1807	144506	CT	0.81	GA	0.82
matR-1771	144542	CT	0.8	GA	0.82
matR-1751	144562	CT	0.91	GA	0.89
matR-1731	144582	CT	0.71	GA	0.85
matR-1730	144583	CT	0.94	GA	0.95

matR-1596	144717	CT	0.57	GA	0.48
matR-1593	144720	CT	0.62	GA	0.63
matR-1408	144905	CT	0.14	GA	0.15
matR-461	145852	CT	0.95	GA	0.94
matR-374	145939	CT	0.87	GA	0.88
matR-214	146099	CT	0.18	GA	0.17
rpl2-212	154955	CT	0.47	GA	0.41
rpl2-711	155454	CT	0.14	GA	0.12
orfX-59	157549	CT	0.82	GA	0.95
orfX-145	157635	CT	0.77	GA	0.64
orfX-161	157651	CT	0.71	GA	0.62
orfX-164	157654	CT	0.71	GA	0.58
orfX-406	157896	CT	0.41	GA	0.4
orfX-407	157897	CT	0.38	GA	0.45
orfX-412	157902	CT	0.37	GA	0.39
orfX-440	157930	CT	0.44	GA	0.78
orfX-474	157964	CT	0.45	GA	0.42
orfX-505	157995	CT	0.85	GA	0.83
orfX-530	158020	CT	0.84	GA	0.75
orfX-538	158028	CT	0.86	GA	0.81
orfX-581	158071	CT	0.58	GA	0.56
orfX-586	158076	CT	0.25	GA	0.27
orfX-643	158133	CT	0.91	GA	0.62
orfX-649	158139	CT	0.83	GA	0.51
orfX-666	158156	CT	0.54	GA	0.53
orfX-693	158183	CT	0.69	GA	0.81

orfX-705	158195	CT	0.53	GA	0.52
orfX-746	158236	CT	0.73	GA	0.88
nad4-29	161721	CT	0.9	GA	0.96
nad4-74	161766	CT	0.98	GA	0.96
nad4-84	161776	CT	0.24	GA	0.26
nad4-158	161850	CT	0.97	GA	0.98
nad4-164	161856	CT	0.85	GA	0.89
nad4-166	161858	CT	0.85	GA	0.92
nad4-197	161889	CT	0.91	GA	0.97
nad4-362	162054	CT	0.99	GA	0.94
nad4-376	162068	CT	0.99	GA	0.93
nad4-403	162095	CT	0.99	GA	0.92
nad4-436	162128	CT	1	GA	0.95
nad4-437	162129	CT	0.98	GA	0.9
nad4-608	163771	CT	0.98	GA	0.99
nad4-767	163930	CT	0.98	GA	0.99
nad4-784	163947	CT	0.98	GA	0.99
nad4-836	163999	CT	0.91	GA	0.92
nad4-1033	167277	CT	0.99	GA	0.98
nad4-1101	167345	CT	0.89	GA	0.58
nad4-1131	167375	CT	0.34	GA	0.21
nad4-1148	167392	CT	0.98	GA	0.99
nad4-1172	167416	CT	1	GA	0.99
nad4-1194	167438	CT	0.12	GA	0.11
nad4-1206	167450	CT	0.27	GA	0.32
nad4-1355	167599	CT	1	GA	1

nad4-1373	167617	CT	0.99	GA	0.98
nad4-1405	169591	CT	0.46	GA	0.8
nad4-1417	169603	CT	0.8	GA	0.94
nad4-1433	169619	CT	0.91	GA	1
orf25-395	188268	CT	0.95	GA	0.97
orf25-382	188281	CT	0.36	GA	0.37
orf25-251	188412	CT	1	GA	0.97
orf25-250	188413	CT	0.88	GA	0.76
orf25-248	188415	CT	1	GA	0.98
orf25-138	188525	CT	0.72	GA	0.89
orf25-89	188574	CT	0.93	GA	0.99
nad4L-197	189035	CT	0.88	GA	0.96
nad4L-188	189044	CT	0.92	GA	0.97
nad4L-131	189101	CT	0.94	GA	0.85
nad4L-110	189122	CT	0.94	GA	0.87
nad4L-100	189132	CT	0.94	GA	0.8
nad4L-95	189137	CT	0.98	GA	0.85
nad4L-86	189146	CT	0.97	GA	0.86
nad4L-41	189191	CT	0.92	GA	0.96
nad4L-21	189211	CT	0.22	GA	0.19
cox3-112	218391	CT	0.98	GA	0.96
cox3-245	218524	CT	0.99	GA	0.98
cox3-257	218536	CT	0.93	GA	0.93
cox3-311	218590	CT	0.96	GA	0.96
cox3-314	218593	CT	0.97	GA	0.98
cox3-413	218692	CT	0.95	GA	0.96

cox3-422	218701	CT	0.98	GA	0.98
ccb382-955	232088	CT	0.86	GA	0.82
ccb382-806	232237	CT	0.88	GA	0.98
ccb382-791	232252	CT	0.71	GA	0.79
ccb382-779	232264	CT	0.9	GA	0.96
ccb382-710	232333	CT	0.98	GA	0.92
ccb382-709	232334	CT	0.49	GA	0.39
ccb382-289	232754	CT	0.83	GA	0.87
ccb382-269	232774	CT	0.92	GA	0.93
ccb382-262	232781	CT	0.92	GA	0.93
ccb382-157	232886	CT	0.92	GA	0.92
ccb382-143	232900	CT	1	GA	1
ccb382-104	232939	CT	1	GA	1
ccb382-44	232999	CT	0.92	GA	0.84
ccb256-619	240140	CT	0.61	GA	0.92
ccb256-618	240141	CT	0.39	GA	0.79
ccb256-614	240145	CT	0.66	GA	0.84
ccb256-608	240151	CT	0.86	GA	0.84
ccb256-575	240184	CT	0.9	GA	0.75
ccb256-568	240191	CT	0.86	GA	0.75
ccb256-548	240211	CT	0.88	GA	0.7
ccb256-521	240238	CT	0.93	GA	0.61
ccb256-184	240575	CT	0.53	GA	0.6
ccb256-133	240626	CT	0.82	GA	0.84
ccb203-391	257086	CT	0.69	GA	0.91
ccb203-356	257121	CT	0.74	GA	0.9

ccb203-344	257133	CT	1	GA	0.91
ccb203-320	257157	CT	0.93	GA	0.65
ccb203-277	257200	CT	0.93	GA	0.75
ccb203-226	257251	CT	0.9	GA	0.8
ccb203-208	257269	CT	0.52	GA	0.65
ccb203-176	257301	CT	0.69	GA	0.66
ccb203-93	257384	CT	0.35	GA	0.31
ccb203-65	257412	CT	0.94	GA	0.9
rps12-285	260317	CT	0.95	GA	0.94
rps12-284	260318	CT	0.96	GA	0.97
rps12-269	260333	CT	0.96	GA	0.96
rps12-221	260381	CT	0.97	GA	0.89
rps12-196	260406	CT	0.94	GA	0.95
rps12-104	260498	CT	0.91	GA	0.85
rps12-84	260518	CT	0.92	GA	0.85
nad3-352	260655	CT	0.88	GA	0.84
nad3-347	260660	CT	0.88	GA	0.87
nad3-250	260757	CT	0.9	GA	0.92
nad3-149	260858	CT	0.81	GA	0.82
nad3-83	260924	CT	0.91	GA	0.89
nad3-64	260943	CT	0.84	GA	0.85
nad3-26	260981	CT	0.86	GA	0.86
nad3-8	260999	CT	0.86	GA	0.61
atp9-167	279061	CT	0.99	GA	0.99
nad1-635	287942	CT	0.62	GA	0.62
nad1-580	287997	CT	0.98	GA	0.94

nad1-571	288006	CT	0.9	GA	0.83
nad1-536	288041	CT	0.93	GA	0.94
nad1-500	288077	CT	0.67	GA	0.4
nad1-493	288084	CT	0.93	GA	0.77
nad1-492	288085	CT	0.88	GA	0.77
nad1-490	288087	CT	0.94	GA	0.85
atp6-2-367	297503	CT	0.89	GA	0.98
atp6-2-145	297725	CT	0.17	GA	0.16
atp1-1425	302265	CT	0.17	GA	0.13
atp1-1415	302275	CT	0.97	GA	0.98
nad1-308	318083	CT	0.9	GA	0.93
nad1-307	318084	CT	0.94	GA	0.95
nad1-167	318224	CT	0.97	GA	0.92
nad1-40	318351	CT	0.22	GA	0.22
nad1-2	318389	CT	0.59	GA	0.62
nad2-1436	327954	CT	0.7	GA	0.88
nad2-1433	327957	CT	0.87	GA	0.95
nad2-1309	329737	CT	0.79	GA	0.76
nad2-1280	329766	CT	0.93	GA	0.87
nad2-1279	329767	CT	0.8	GA	0.81
nad2-1160	329886	CT	0.98	GA	0.98
nad2-995	330051	CT	0.97	GA	0.95
nad2-991	330055	CT	0.97	GA	0.95
nad2-961	330085	CT	0.98	GA	0.99
nad2-953	330093	CT	0.99	GA	0.99
nad2-842	330204	CT	0.93	GA	0.99

nad2-821	330225	CT	0.98	GA	1
nad2-695	332989	CT	0.92	GA	0.85

Table S11 List of symmetrical RNA editing events found in the CDS regions on the mitogenome of *Oryza sativa* variety nipponbare.

RNA editing site	Genomic Position	Type of REEs on SS	Frequency of REEs on SS	Type of REEs on AS	Frequency of REEs on AS
nad1-2	11025	CT	0.87	GA	0.91
nad1-215	11238	CT	0.97	GA	1
nad1-307	11330	CT	0.77	GA	0.95
nad1-308	11331	CT	0.75	GA	0.97
cox3-809	17260	CT	0.99	GA	1
cox3-799	17270	CT	0.99	GA	1
cox3-572	17497	CT	0.97	GA	0.99
cox3-467	17602	CT	0.99	GA	0.98
cox3-458	17611	CT	0.99	GA	0.99
cox3-359	17710	CT	0.98	GA	0.98
cox3-356	17713	CT	0.99	GA	0.99
cox3-334	17735	CT	0.98	GA	0.98
cox3-302	17767	CT	0.95	GA	0.93
cox3-290	17779	CT	0.99	GA	0.99
orf25-419	18577	CT	0.99	GA	0.97
orf25-398	18598	CT	0.98	GA	0.96
orf25-121	18875	CT	0.99	GA	1

orf25-92	18904	CT	0.99	GA	1
orf25-79	18917	CT	1	GA	1
orf25-74	18922	CT	1	GA	1
orf25-62	18934	CT	1	GA	1
orf25-59	18937	CT	1	GA	0.87
orf25-363	19809	CT	0.35	GA	0.22
rps7-332	46680	CT	0.98	GA	0.97
rps7-141	46871	CT	0.3	GA	0.32
orfB-60	53483	CT	0.12	GA	0.12
orfB-123	53546	CT	0.97	GA	0.98
orfB-442	53865	CT	0.19	GA	0.34
nad6-569	59568	CT	0.95	GA	0.97
nad6-567	59570	CT	1	GA	1
nad6-562	59575	CT	1	GA	1
nad6-476	59661	CT	1	GA	0.96
nad6-446	59691	CT	1	GA	1
nad6-191	59946	CT	1	GA	1
nad6-89	60048	CT	0.98	GA	0.98
nad6-88	60049	CT	0.98	GA	0.98
nad6-26	60111	CT	0.98	GA	0.98
nad6-25	60112	CT	0.97	GA	0.96
ccmC-665	60468	CT	0.95	GA	0.92
ccmC-624	60509	CT	0.9	GA	0.79
ccmC-619	60514	CT	0.93	GA	0.68
ccmC-614	60519	CT	0.96	GA	0.65
ccmC-608	60525	CT	0.95	GA	0.79

ccmC-575	60558	CT	0.84	GA	0.93
ccmC-568	60565	CT	0.9	GA	0.97
ccmC-548	60585	CT	0.85	GA	0.94
ccmC-521	60612	CT	0.8	GA	0.8
ccmC-400	60733	CT	0.83	GA	0.97
ccmC-331	60802	CT	0.96	GA	0.98
ccmC-299	60834	CT	0.96	GA	0.98
ccmC-236	60897	CT	0.99	GA	0.98
ccmC-184	60949	CT	1	GA	0.91
ccmC-179	60954	CT	1	GA	0.95
ccmC-115	61018	CT	0.94	GA	0.96
ccmC-103	61030	CT	0.95	GA	1
ccmC-76	61057	CT	0.98	GA	1
orf183-422	61497	CT	1	GA	0.91
orfX-700	78416	CT	0.94	GA	0.99
orfX-694	78422	CT	0.93	GA	0.99
orfX-687	78429	CT	0.87	GA	0.93
orfX-637	78479	CT	0.97	GA	0.96
orfX-581	78535	CT	0.98	GA	0.99
orfX-580	78536	CT	0.12	GA	0.12
orfX-575	78541	CT	0.98	GA	1
orfX-532	78584	CT	0.98	GA	0.94
orfX-468	78648	CT	0.58	GA	0.61
orfX-464	78652	CT	0.92	GA	0.78
orfX-434	78682	CT	0.9	GA	0.8
orfX-406	78710	CT	0.88	GA	0.79

orfX-403	78713	CT	0.7	GA	0.68
orfX-401	78715	CT	0.8	GA	0.68
orfX-373	78743	CT	0.95	GA	0.85
orfX-371	78745	CT	0.92	GA	0.78
orfX-358	78758	CT	0.9	GA	0.75
orfX-355	78761	CT	0.95	GA	0.75
orfX-308	78808	CT	0.71	GA	0.62
orfX-289	78827	CT	0.9	GA	0.9
orfX-251	78865	CT	0.73	GA	0.96
orfX-229	78887	CT	0.79	GA	0.84
orfX-215	78901	CT	1	GA	0.81
orfX-205	78911	CT	1	GA	0.88
orfX-158	78958	CT	0.94	GA	0.84
orfX-157	78959	CT	0.54	GA	0.41
orfX-155	78961	CT	0.85	GA	0.61
orfX-149	78967	CT	0.9	GA	0.78
orfX-127	78989	CT	0.85	GA	0.73
orfX-102	79014	CT	0.82	GA	0.64
orfX-91	79025	CT	0.89	GA	0.65
orfX-86	79030	CT	0.86	GA	0.57
orfX-64	79052	CT	0.88	GA	0.69
orfX-53	79063	CT	0.87	GA	0.59
nad7-1103	84968	CT	1	GA	0.98
nad7-836	86919	CT	1	GA	1
nad7-739	87016	CT	1	GA	1
nad7-734	87021	CT	1	GA	1

nad7-724	87031	CT	1	GA	0.96
nad7-698	87057	CT	1	GA	1
nad7-679	88072	CT	0.61	GA	0.88
nad7-578	88173	CT	1	GA	1
nad7-534	88217	CT	0.28	GA	0.24
nad7-533	88218	CT	0.99	GA	0.98
nad7-531	88220	CT	0.13	GA	0.14
nad7-383	88368	CT	0.99	GA	0.99
nad7-344	88407	CT	0.99	GA	1
nad7-335	88416	CT	0.99	GA	1
nad7-315	88436	CT	0.35	GA	0.39
nad7-251	88500	CT	0.98	GA	0.71
nad7-244	88507	CT	0.98	GA	0.81
nad7-224	88527	CT	0.95	GA	0.9
nad7-211	89852	CT	0.75	GA	0.79
nad7-137	90737	CT	0.93	GA	0.94
nad7-99	90775	CT	0.95	GA	0.92
nad7-83	90791	CT	0.98	GA	0.95
nad7-77	90797	CT	0.98	GA	0.97
nad7-45	90829	CT	0.97	GA	0.93
nad7-44	90830	CT	0.97	GA	0.95
nad7-38	90836	CT	0.98	GA	0.97
rps3-58	170893	CT	0.26	GA	0.27
rps3-64	170899	CT	0.4	GA	0.46
rps3-69	170904	CT	0.32	GA	0.35
rps3-170	172852	CT	0.26	GA	0.34

rps3-502	173184	CT	0.68	GA	0.76
rps3-643	173325	CT	0.77	GA	0.79
rps3-814	173496	CT	0.16	GA	0.18
rps3-955	173637	CT	0.85	GA	0.94
rps3-1285	173967	CT	0.4	GA	0.28
rps3-1512	174194	CT	0.81	GA	0.83
rps3-1543	174225	CT	1	GA	0.95
rps3-1557	174239	CT	1	GA	0.95
rpl16-225	174371	CT	0.93	GA	0.96
rpl16-227	174373	CT	0.93	GA	0.9
rpl16-228	174374	CT	0.85	GA	0.92
rpl16-287	174433	CT	0.95	GA	0.95
rpl16-355	174501	CT	1	GA	1
rpl16-444	174590	CT	0.57	GA	0.77
rpl16-458	174604	CT	0.98	GA	1
rpl16-524	174670	CT	0.88	GA	0.9
rpl16-530	174676	CT	0.86	GA	0.95
nad3-172	175573	CT	0.59	GA	0.57
nad3-173	175574	CT	0.75	GA	0.66
nad3-190	175591	CT	0.51	GA	0.44
nad3-191	175592	CT	0.6	GA	0.67
nad3-257	175658	CT	0.81	GA	0.77
nad3-296	175697	CT	0.8	GA	0.89
nad3-301	175702	CT	0.8	GA	0.94
nad3-319	175720	CT	0.88	GA	0.87
nad3-320	175721	CT	0.88	GA	0.9

nad3-358	175759	CT	0.91	GA	0.61
nad3-362	175763	CT	0.95	GA	0.62
nad3-455	175856	CT	0.81	GA	0.86
nad3-460	175861	CT	0.76	GA	0.83
rps12-71	175984	CT	0.88	GA	0.97
rps12-196	176109	CT	0.75	GA	0.9
rps12-221	176134	CT	0.63	GA	0.69
rps12-269	176182	CT	0.73	GA	0.71
rps12-284	176197	CT	1	GA	0.7
rps2-776	184863	CT	0.82	GA	0.72
rps2-769	184870	CT	0.87	GA	0.69
rps2-745	184894	CT	0.81	GA	0.68
rps2-674	184965	CT	0.73	GA	0.88
rps2-479	185160	CT	0.8	GA	0.82
rps2-425	185214	CT	0.8	GA	0.79
rps2-404	185235	CT	0.88	GA	0.8
rps2-203	185436	CT	0.2	GA	0.17
rps2-158	185481	CT	0.41	GA	0.38
rps2-12	185627	CT	0.22	GA	0.29
nad4-44	198428	CT	0.98	GA	0.88
nad4-154	198538	CT	0.96	GA	0.98
nad4-164	198548	CT	0.95	GA	0.98
nad4-166	198550	CT	0.91	GA	0.97
nad4-197	198581	CT	0.94	GA	1
nad4-317	198701	CT	1	GA	0.97
nad4-362	198746	CT	0.99	GA	0.98

nad4-376	198760	CT	0.99	GA	1
nad4-401	198785	CT	0.95	GA	0.9
nad4-416	198800	CT	0.94	GA	0.88
nad4-433	198817	CT	0.83	GA	0.81
nad4-437	198821	CT	0.94	GA	0.97
nad4-449	198833	CT	0.86	GA	0.88
nad4-1417	207426	CT	0.56	GA	0.79
cox2-7	212419	CT	0.14	GA	0.1
cox2-14	212426	CT	0.98	GA	0.99
cox2-30	212442	CT	0.85	GA	0.89
cox2-33	212445	CT	0.94	GA	0.97
cox2-77	212489	CT	0.98	GA	0.98
cox2-144	212556	CT	0.71	GA	0.68
cox2-162	212574	CT	0.1	GA	0.11
cox2-167	212579	CT	0.98	GA	0.97
cox2-169	212581	CT	0.98	GA	0.97
cox2-449	214136	CT	0.94	GA	0.96
cox2-466	214153	CT	0.94	GA	0.95
cox2-467	214154	CT	0.94	GA	0.95
cox2-482	214169	CT	0.96	GA	0.96
cox2-550	214237	CT	0.99	GA	0.98
cox2-563	214250	CT	0.98	GA	0.99
cox2-638	214325	CT	0.95	GA	0.94
cox2-704	214391	CT	0.95	GA	0.94
atp6-1003	225273	CT	0.58	GA	0.95
atp6-965	225311	CT	0.71	GA	0.95

atp6-956	225320	CT	0.84	GA	0.95
atp6-949	225327	CT	0.74	GA	0.94
atp6-812	225464	CT	1	GA	0.98
atp6-770	225506	CT	0.98	GA	1
atp6-555	225721	CT	0.85	GA	0.87
atp6-554	225722	CT	0.98	GA	0.89
atp6-547	225729	CT	0.98	GA	0.92
atp6-539	225737	CT	0.98	GA	0.91
atp6-521	225755	CT	0.91	GA	0.76
atp6-514	225762	CT	0.97	GA	0.85
atp6-410	225866	CT	0.92	GA	0.91
atp6-368	225908	CT	0.94	GA	0.98
atp6-341	225935	CT	0.97	GA	0.96
atp6-13	226263	CT	0.84	GA	0.83
nad5-1918	231615	CT	0.97	GA	0.97
nad5-1916	231617	CT	0.97	GA	0.98
nad5-1901	231632	CT	0.74	GA	0.72
nad5-1900	231633	CT	0.96	GA	0.97
nad5-1895	231638	CT	0.95	GA	0.98
nad5-1611	232848	CT	0.1	GA	0.17
nad5-1591	232868	CT	0.13	GA	0.14
nad5-1589	232870	CT	0.99	GA	1
nad5-1580	232879	CT	0.99	GA	1
nad5-1567	232892	CT	0.13	GA	0.15
nad5-1550	232909	CT	0.99	GA	0.99
nad5-1490	232969	CT	1	GA	0.99

nad1-635	239915	CT	0.98	GA	0.83
nad1-577	239973	CT	1	GA	1
nad1-555	239995	CT	1	GA	0.97
nad1-537	240013	CT	0.7	GA	0.59
nad1-536	240014	CT	1	GA	0.97
nad1-500	240050	CT	0.86	GA	0.97
rps13-287	242555	CT	0.98	GA	0.91
rps13-256	242586	CT	0.98	GA	0.91
rps13-100	242742	CT	0.76	GA	0.79
rps13-56	242786	CT	0.94	GA	0.67
rps4-1027	248612	CT	1	GA	0.91
rps4-1022	248617	CT	1	GA	0.91
rps4-962	248677	CT	0.9	GA	0.94
rps4-926	248713	CT	0.94	GA	1
rps4-897	248742	CT	1	GA	0.93
rps4-858	248781	CT	0.8	GA	0.57
rps4-290	249349	CT	0.97	GA	0.96
rps4-278	249361	CT	1	GA	1
rps4-49	249590	CT	0.97	GA	1
rps4-38	249601	CT	0.97	GA	0.97
rps11-354	250243	CT	0.54	GA	0.42
atp9-81	263221	CT	0.86	GA	0.88
atp9-82	263222	CT	0.98	GA	0.99
atp9-205	263345	CT	0.97	GA	0.96
atp9-212	263352	CT	0.99	GA	0.98
rps19-76	297760	CT	0.26	GA	0.33

rps19-116	297800	CT	0.94	GA	0.96
rps19-138	297822	CT	0.78	GA	0.82
rps19-163	297847	CT	0.88	GA	0.87
rps19-164	297848	CT	0.97	GA	0.99
nad4L-2	298830	CT	0.61	GA	0.64
nad4L-8	298836	CT	0.33	GA	0.43
nad4L-188	299016	CT	0.96	GA	0.96
nad4L-197	299025	CT	0.97	GA	0.96
cob-1124	303918	CT	0.97	GA	0.96
cob-1098	303944	CT	0.29	GA	0.26
cob-982	304060	CT	0.88	GA	0.86
cob-908	304134	CT	0.96	GA	0.92
cob-853	304189	CT	0.98	GA	0.97
cob-725	304317	CT	0.97	GA	0.98
cob-680	304362	CT	0.98	GA	0.59
cob-568	304474	CT	0.92	GA	0.9
cob-419	304623	CT	0.99	GA	0.95
cob-325	304717	CT	0.9	GA	0.96
cob-298	304744	CT	0.91	GA	0.96
cob-286	304756	CT	0.92	GA	0.95
nad1-953	315061	CT	0.97	GA	0.97
nad1-939	315075	CT	0.15	GA	0.12
nad1-937	315077	CT	0.98	GA	0.98
nad1-898	315116	CT	0.99	GA	0.97
nad1-823	315191	CT	0.98	GA	1
nad1-779	315235	CT	0.97	GA	0.98

nad1-743	315271	CT	0.99	GA	1
nad1-734	315280	CT	0.97	GA	1
matR-1800	316049	CT	0.16	GA	0.17
matR-1766	316083	CT	0.9	GA	0.9
matR-1745	316104	CT	0.81	GA	0.89
rps1-505	321382	CT	0.9	GA	0.91
rps1-377	321510	CT	0.98	GA	0.95
ccmFn-1772	322084	CT	0.12	GA	0.12
ccmFn-1549	322307	CT	1	GA	0.96
ccmFn-1514	322342	CT	1	GA	1
ccmFn-1501	322355	CT	1	GA	1
ccmFn-1466	322390	CT	1	GA	0.88
ccmFn-1454	322402	CT	1	GA	0.77
ccmFn-1453	322403	CT	0.76	GA	0.36
ccmFn-1450	322406	CT	0.95	GA	0.72
ccmFn-1430	322426	CT	0.91	GA	0.7
ccmFn-1336	322520	CT	0.53	GA	1
ccmFn-1318	322538	CT	0.7	GA	1
ccmFn-1286	322570	CT	1	GA	1
ccmFn-1258	322598	CT	1	GA	0.97
ccmFn-943	322913	CT	1	GA	1
ccmFn-794	323062	CT	1	GA	0.96
ccmFn-779	323077	CT	1	GA	0.98
ccmFn-767	323089	CT	0.93	GA	0.96
ccmFn-745	323111	CT	1	GA	0.97
ccmFn-707	323149	CT	0.98	GA	0.97

ccmFn-698	323158	CT	0.94	GA	0.94
ccmFn-550	323306	CT	0.89	GA	0.94
ccmFn-469	323387	CT	0.31	GA	0.38
ccmFn-388	323468	CT	0.1	GA	0.13
ccmFn-372	323484	CT	0.97	GA	0.97
ccmFn-365	323491	CT	0.97	GA	0.97
ccmFn-356	323500	CT	0.99	GA	0.99
ccmFn-142	323714	CT	0.75	GA	0.77
ccmFn-137	323719	CT	0.83	GA	0.79
ccmFn-121	323735	CT	0.76	GA	0.74
ccmFn-98	323758	CT	0.74	GA	0.54
ccmFn-38	323818	CT	0.75	GA	0.87
ccmFc-38	330634	CT	0.79	GA	0.81
ccmFc-50	330646	CT	0.81	GA	0.71
ccmFc-52	330648	CT	0.84	GA	0.73
ccmFc-146	330742	CT	0.7	GA	0.76
ccmFc-301	330897	CT	0.77	GA	0.92
ccmFc-325	330921	CT	0.53	GA	0.54
ccmFc-369	330965	CT	0.27	GA	0.27
ccmFc-397	330993	CT	0.77	GA	0.81
ccmFc-543	331139	CT	0.62	GA	0.68
ccmFc-900	332500	CT	0.58	GA	0.73
ccmFc-960	332560	CT	0.96	GA	0.92
ccmFc-1130	332730	CT	0.93	GA	0.82
cox1-15	338586	CT	0.8	GA	0.53
cox1-1403	339974	CT	0.95	GA	1

cox1-1406	339977	CT	0.94	GA	0.98
cox1-1434	340005	CT	0.91	GA	0.93
cox1-1490	340061	CT	0.96	GA	0.95
atp1-1490	353868	CT	0.99	GA	1
atp1-1499	353877	CT	0.99	GA	1
nad9-14	361806	CT	0.98	GA	0.98
nad9-92	361884	CT	0.97	GA	0.99
nad9-111	361903	CT	0.2	GA	0.19
nad9-113	361905	CT	0.96	GA	0.99
nad9-167	361959	CT	0.98	GA	0.98
nad9-190	361982	CT	0.98	GA	0.98
nad9-223	362015	CT	0.99	GA	0.98
nad9-298	362090	CT	0.99	GA	0.98
nad9-311	362103	CT	1	GA	0.98
nad9-328	362120	CT	1	GA	0.99
nad9-356	362148	CT	1	GA	0.99
nad9-398	362190	CT	0.99	GA	0.99
nad2-621	363905	CT	1	GA	0.9
nad2-788	366469	CT	0.95	GA	0.96
nad2-809	366490	CT	0.83	GA	0.97
nad2-1148	366829	CT	1	GA	1
nad2-1397	368480	CT	0.98	GA	0.94
nad2-1400	368483	CT	0.98	GA	0.95
nad2-1403	368486	CT	0.98	GA	0.95
nad2-1408	368491	CT	0.97	GA	1
nad2-1409	368492	CT	0.97	GA	0.99

nad2-1416	368499	CT	1	GA	0.94
ccmB-28	391782	CT	1	GA	0.97
ccmB-43	391797	CT	0.97	GA	0.97
ccmB-304	392058	CT	0.88	GA	0.77
ccmB-338	392092	CT	0.89	GA	0.92
ccmB-367	392121	CT	0.83	GA	0.84
ccmB-428	392182	CT	0.9	GA	0.87
ccmB-467	392221	CT	0.88	GA	0.8
ccmB-475	392229	CT	0.8	GA	0.66
ccmB-476	392230	CT	0.8	GA	0.66
ccmB-485	392239	CT	0.83	GA	0.68
ccmB-596	392350	CT	0.96	GA	0.96
nad2-523	403809	CT	0.88	GA	0.86
nad2-26	405516	CT	0.94	GA	0.9

Table S12 Symmetric RNA-editing sites found among homologous CDS from the mitogenomes of *A. thaliana*, *O. sativa* and *S. miltiorrhiza* (see the excel file).

Table S13 Primers used for the validation of selected RNA editing sites.

Gene	Primer sequence (5'-3')
cox2	Forward: CAGAAGATGATCCAGAATTGGGTCA
	Reverse: GTTCCACAAATCTCACTGCACTGAC
nad1	Forward: CCGCCTATCCTAGATCTTCCC
	Reverse: GGGAGCCATTGAAAGGTGAC
nad5[a]	Forward: GCACAGATAGGATCGCATACTTGG
	Reverse: CGCATATCTTGCTCATCCGACA
nad6	Forward: CCAACCCAAAGAAATACGACCTCTC
	Reverse: GATCGTGAGTGGGTCTGTCGTC
rpl16[a]	Forward: GGAGGAAGCTACTCCATTTTTACG
	Reverse: GGACACACGAGCAATCCAACC
rps3[a]	Forward: GCTTCTCCGATTGCTCAAGACA
	Reverse: CCACACTTTGACACCTAAGATTC
cox3	Forward: GCGCGATGTTCTACGTGAATCC
	Reverse: GCGAGTATAGCATGATGAGCCCA
orf214[a]	Forward: GGGGCGTGCTATATCCAAAAC
	Reverse: TCTACAATGGCGCTTTAGGTGTTTC
orf456	Forward: GGGCCATCTGCTGAAATGTCA
	Reverse: GCAGATTCACACCAAATGCTTCC
rpl2	Forward: GCAATGGAACCGTAGACAGTCAAG
	Reverse: CAAAGGTAGGGCATTTCCCATTT

[a]These primers are also used in the experiments to validate symmetrical RNA editing sites.
